# Molecular Phylogeny and Biogeographic Diversification of Linnaeoideae (Caprifoliaceae s. l.) Disjunctly Distributed in Eurasia, North America and Mexico

**DOI:** 10.1371/journal.pone.0116485

**Published:** 2015-03-10

**Authors:** Hua-Feng Wang, Sven Landrein, Wen-Pan Dong, Ze-Long Nie, Katsuhiko Kondo, Tsuneo Funamoto, Jun Wen, Shi-Liang Zhou

**Affiliations:** 1 State Key Laboratory of Systematic and Evolutionary Botany, Institute of Botany, Chinese Academy of Sciences, Beijing 100093, China; 2 Royal Botanic Gardens, Kew, Richmond, Surrey, TW9 3AB, United Kingdom; 3 College of Life Sciences, University of Chinese Academy of Sciences, Beijing 100049, China; 4 Key Laboratory of Plant Resources Conservation and Utilization, College of Biology and Environmental Sciences, Jishou University, Jishou, Hunan 416000, China; 5 Laboratory of Plant Genetics and Breeding Science, Department of Agriculture, Faculty of Agriculture, Tokyo University of Agriculture, 1737 Funako, Atsugi City, Kanagawa Prefecture 243–0034, Japan; 6 Biological Institute, Fundamental Education and Research Centre of Pharmaceutical Sciences, Showa Pharmaceutical University, 3-chome, Higashi-Tamagawagakuen, Machida City, Tokyo 194–8543, Japan; 7 Department of Botany, National Museum of Natural History, MRC 166, Smithsonian Institution, Washington, DC 20013–7012, United States of America; 8 Beijing Urban Ecosystem Research Station, State Key Laboratory of Urban and Regional Ecology, Research Center for Eco-Environmental Sciences, Chinese Academy of Sciences, 100085 Beijing, China; Field Museum of Natural History, UNITED STATES

## Abstract

Linnaeoideae is a small subfamily of erect or creeping shrubs to small trees in Caprifoliaceae that exhibits a wide disjunct distribution in Eurasia, North America and Mexico. Most taxa of the subfamily occur in eastern Asia and Mexico but the monospecific genus *Linnaea* has a circumboreal to north temperate distribution. In this study, we conducted phylogenetic and biogeographic analyses for Linnaeoideae and its close relatives based on sequences of the nuclear ribosomal ITS and nine plastid (*rbc*L, *trn*S-G, *mat*K, *trn*L-F, *ndh*A, *trn*D-*psb*M, *pet*B-D, *trn*L-*rpl*32 and *trn*H-*psb*A) markers. Our results support that Linnaeoideae is monophyletic, consisting of four eastern Asian lineages (*Abelia*, *Diabelia*, *Dipelta* and *Kolkwitzia*), the Mexican *Vesalea*, and *Linnaea*. The Mexican *Vesalea* was formerly placed in *Abelia*, but it did not form a clade with the eastern Asian *Abelia*; instead *Vesalea* and *Linnaea* are sisters. The divergence time between the eastern Asian lineages and the Mexican *Vesalea* plus the *Linnaea* clade was dated to be 50.86 Ma, with a 95% highest posterior density of 42.8 Ma (middle Eocene) to 60.19 Ma (early Paleocene) using the Bayesian relaxed clock estimation. Reconstructed ancestral areas indicated that the common ancestor of *Linnaea* plus *Vesalea* may have been widespread in eastern Asia and Mexico or originated in eastern Asia during the Eocene and likely migrated across continents in the Northern Hemisphere via the North Atlantic Land Bridges or the Bering Land Bridge. The Qinling Mountains of eastern Asia are the modern-day center of diversity of *Kolkwitzia-Dipelta-Diabelia* clade. The *Diabelia*clade became highly diversified in Japan and eastern China. Populations of *Diabelia serrata* in Japan and eastern China were found to be genetically identical in this study, suggesting a recent disjunction across the East China Sea, following the last glacial event.

## Introduction

**Fig 1:**
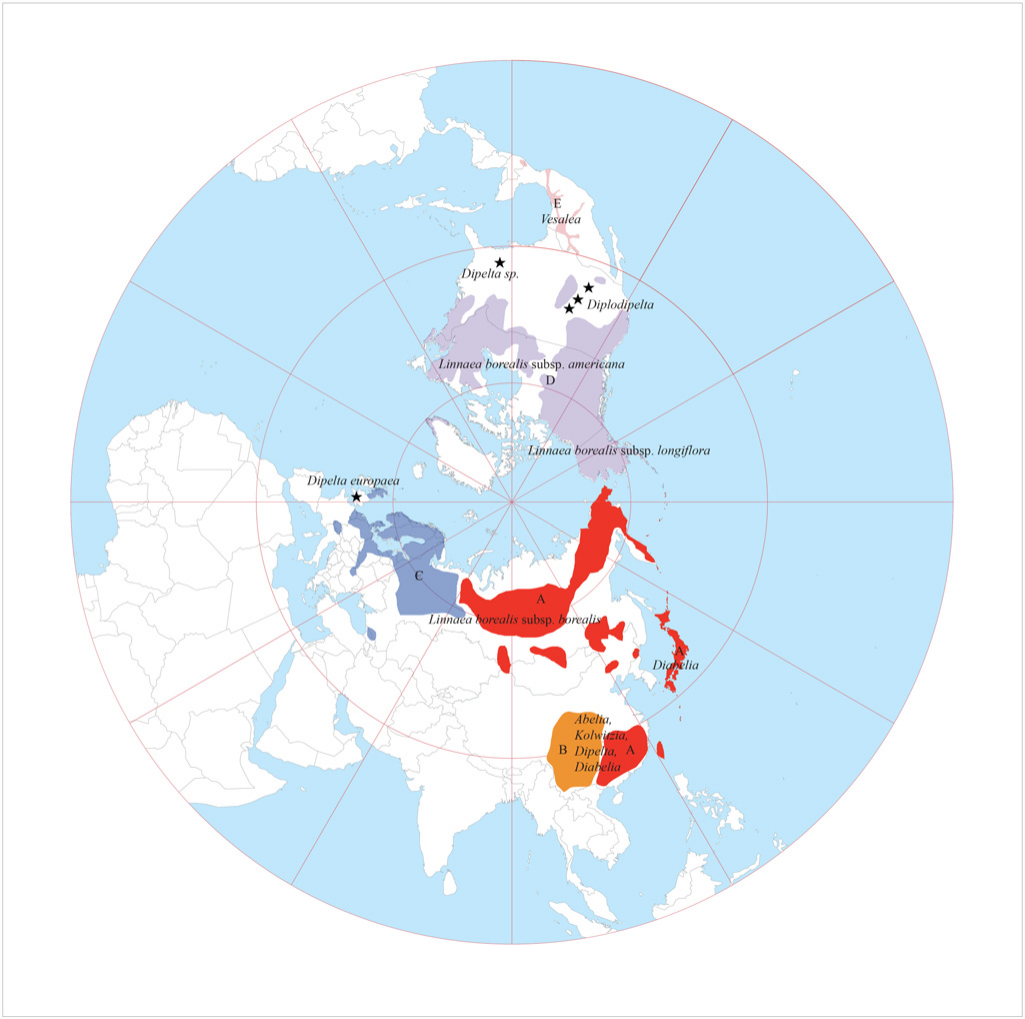
Distribution of extant Linnaeoideae species and known fossils showing intercontinental disjunctions in the Northern Hemisphere.

Intercontinental disjunct distributions of plants have fascinated botanists for centuries. The East Asian—North American plant disjunctions have attracted much attention in the last two decades [1–9]. The disjunct patterns have also been utilized to understand the histories of plant spreading between continents as well as allopatric speciation [5,6,10]. Fossil, molecular and geological data all suggest that the disjunctions between East Asia and North America originated many times in multiple areas throughout the Tertiary [4,5,9,11,12]. However, these studies have primarily focused on distributions restricted to the Northern Hemisphere, north of Mexico [13]. Fewer studies explored the evolution of the intercontinental disjunct pattern involving lineages distributed in Central America, such as in Mexico [14–16]. Linnaeoideae is such an example with taxa distributed in Eurasia and North America including Mexico [14] (Fig. 1).

Linnaeoideae (Dumortier) P.F. Stevens is a subfamily of Caprifoliaceae s.l. (including Caprifolioideae, Diervilloideae, Dipsacoideae, Linnaeoideae, Morinoideae, Valerianoideae and *Zabelia*) [17,95]. This re-circumscribed family of Caprifoliaceae s.l. has been shown to be monophyletic in several recent studies [18–22]. The nomenclature of the clade is also subject to debate; we herein use the subfamily rank Linnaeoideae, although some authors used the tribe rank Linnaeeae Dumortier [21,25,28,38,73].

Improvements from successive molecular studies have provided important insights into the phylogeny of Linnaeoideae. (1) Based on *rbc*L, ITS sequences and morphological data from 45 samples (not including *Diabelia*, *Vesalea* and *Zabelia*), Donoghue et al. [19] found that Linnaeoideae was monophyletic and more closely related to Dipsacoideae than to Caprifolioideae. (2) Based on *ndh*F and *trn*L-F from 26 samples (not including *Diabelia*, *Vesalea* and *Zabelia*), Zhang et al. [20] supported the classification of Caprifoliaceae s.l. as in Donoghue et al. [19]. (3) Based on *ndh*F, *trn*L-F, *trn*L, *mat*K and *rbc*L*-atp*B regions from 30 samples (not including *Diabelia*, *Vesalea* and *Zabelia*), Bell et al.[18] supported that Linnaeoideae was monophyletic. (4) Based on nuclear and plastid sequence data (ITS, *trn*K, *mat*K, *atp*B*-rbc*L and *trn*L-F) from 51 samples (including all six genera), Jacobs et al. [23] were able to resolve a greater number of monophyletic subgroups within Linnaeoideae, now including *Abelia*, *Dipelta*, *Kolkwitzia*, *Linnaea* as well as *Vesalea*. They also questioned the position of *Zabelia* (Rehder) Makino and a sister relationship with Morinoideae or Valerianoideae was proposed but with low support [23]. (5) Using five plastid regions (*rbc*L, *ndh*F, *trn*L intron, *trn*L-F spacer and *mat*K) from 31 samples (including all six genera), Landrein et al. [38] identified a new monophyletic group designated at the generic level as *Diabelia*.

Christenhusz [29] argued for the broadest sense of *Linnaea* L. to include all members of Linnaeoideae except *Zabelia*. Major morphological differences within Linnaeoideae as well as a need to keep the number of nomenclatural changes to a minimum would direct to keep the monophyletic clades distinct. The redefined Linnaeoideae here includes six genera: *Abelia* R.Br., *Diabelia* Landrein, *Dipelta* Maxim., *Kolkwitzia* Graebn., *Linnaea* Dumortier and *Vesalea* M. Martens & Galeotti (Table 1). The subfamily is characterized by achenes topped with persistent sepals, three to four locular ovaries with only one or two fertile locules, a distinct epicalyx, and four stamens.

**Table 1:** The classification systems of taxa in Linnaeoideae.

Fukuoka [96]	Donoghue [97]	Takhtajan [98]	Kim [73]	Landrein et al. [38]	Christenhusz [29]
*Symphoricarpos*	*Symphoricarpos*	*Symphoricarpos*,*Heptacodium*			
*Linnaea*	*Linnaea*	*Linnaea*	*Linnaea*	*Linnaea*	*Linnaea (incl*. *Abelia*, *Dipelta*, *Kolkwitzia*, *Vesalea and Diabelia)*
*Dipelta*	*Dipelta*	*Dipelta*	*Dipelta*	*Dipelta*	
*Kolkwitzia*	*Kolkwitzia*	*Kolkwitzia*	*Kolkwitzia*	*Kolkwitzia*	
*Abelia*	*Abelia* (incl. *Zabelia*)	*Abelia* (incl. *Zabelia*)	*Abelia*	*Abelia*	
				*Diabelia*,	
			*Vesalea*	*Vesalea*,	
*Zabelia*			*Zabelia*	*Zabelia*	*Zabelia*

Linnaeoideae exhibits a wide intercontinental disjunct distribution in Eurasia, North America and Mexico (Fig. 1, and Figs. 6–8 of Tang and Li [30]). *Abelia*, *Dipelta* and *Kolkwitzia* (Fig. 8 of Tang and Li [30]) are endemic to China except for *A*. *chinensis*, which reaches the Ryukyu Islands, Japan. *Diabelia* is restricted to Japan with one locality found on the eastern coast of China [31,32]. *Vesalea* is endemic to Mexico. *Linnaea* has a circumboreal distribution with relic populations found in Japan (e.g. Iwate, Nagano, Yamanashi), Northeast China, Central Asia, the Caucasus, the Alps, the Appalachian and the Rocky Mountains (Fig. 6 of Tang and Li [30]).

The phylogeny and biogeography of Linnaeoideae remain poorly understood, and the unique disjunct distribution in East Asia and Mexico deserve far more attention. We thus conducted a phylogenetic study on Linnaeoideae using one nuclear marker (ITS) and nine chloroplast markers (*rbc*L, *trn*S-G, *mat*K, *trn*L-F, *ndh*A, *trn*D*-psb*M, *pet*B-D, *trn*L*-rpl*32 and *trn*H*-psb*A) with a nearly complete taxon sampling, including all genera, and most species, except *Dipelta wenxianensis*. Our objectives are to: (1) reconstruct the phylogeny of Linnaeoideae; (2) estimate the divergence times of the major lineages of Linnaeoideae using a fossil-calibrated molecular clock method; and (3) explore the evolution of the unique biogeographic distributions in Linnaeoideae, emphasizing on the intercontinental disjunctions in Eurasia, Mexico, and North America.

## Materials and Methods

### Ethics Statement

Linnaeoideae are not included in any Eurasian, North American or Mexican official list of threatened plants. No special permits were required for this study. The field studies did not involve endangered or protected species and the specific location of our study was provided in Table 2. Herbarium voucher specimens were deposited in the Institute of Botany, Beijing (PE) and Kew (K). The sequences determined in this study were listed in Table 2 (GenBank accession numbers: KP297477-KP297801). All sequence data have been deposited in GenBank under accession numbers KP297477-KP297801.

**Table 2:** Sampling information and the GenBank accession numbers of sequences used in this study.

	Taxon	Locality	Voucher	ITS	*trn*L-trnF	*mat*K	*rbc*L	*trn*S-trnG	*ndh*A	*psb*M-*trn*D	*pe*tB-petD	*trn*L*-rpl*32	*trn*H*-psb*A	References
Ingroup	*Dipelta yunnanensis* Franchet	China, Yunnan, Deqin	S. L. Zhou 465 (PE)	-	KP297750	KP297510	KP297686	KP297785	KP297558	KP297653	-	KP297723	KP297620	This study
Ingroup	*Abelia* × *grandiflora* (Rovelli exAndré) Rehder	China, Zhejiang, Hangzhou Botanical Garden	*S*.*L*. *Zhou 470* (PE)	KP297483	KP297774	KP297534	KP297667	KP297774	KP297548	KP297642	KP297576	KP297726	KP297609	This study
Ingroup	*Abelia* × *grandiflora* (Rovelli ex André) Rehder	Japan, Yamanashi	*T*. *Funamota 471* (PE)	KP297484	KP297775	KP297516	KP297668	KP297775	KP297549	KP297643	KP297577	KP297727	KP297610	This study
Ingroup	*Abelia chinensis* R. Br.	China, Beijing, Institute of Botany	*S*.*L*. *Zhou 486* (PE)	KP297477	KP297733	KP297533	KP297670	KP297768	KP297542	KP297636	KP297570	KP297718	KP297603	This study
Ingroup	*Abelia chinensis* R. Br.	China, Chongqing, Wanxian	*J*. *Wen 487* (PE)	KP297478	KP297734	KP297513	KP297671	KP297769	KP297543	KP297637	KP297571	KP297717	KP297604	This study
Ingroup	*Abelia engleriana* (Graebn.) Rehder	China, Chongqing, Kaixian, Xuebaoshan mountain	*S*. *L*. *Zhou and J*. *Wen 494* (PE)	KP297479	KP297735	KP297514	KP297673	KP297770	KP297544	KP297638	KP297572	KP297716	KP297605	This study
Ingroup	*Abelia engleriana* (Graebn.) Rehder	China, Chongqing, Kaixian, Xuebaoshan mountain	*S*. *L*. *Zhou and J*. *Wen* 495 (PE)	KP297480	KP297736	KP297515	KP297674	KP297771	KP297545	KP297639	KP297573	KP297715	KP297606	This study
Ingroup	*Abelia macrotera* (Graebn. et Buchw.) Rehder.	China, Chongqing, Nanchuan, Jinfoshan	*S*. *L*. *Zhou 508* (PE)	KP297485	KP297741	KP297517	KP297675	KP297776	KP297551	KP297645	KP297579	KP297712	KP297611	This study
Ingroup	*Abelia macrotera* (Graebn. et Buchw.) Rehder.	China, Chongqing, Nanchuan, Jinfoshan	*S*. *L*. *Zhou 521* (PE)	KP297486	KP297742	KP297518	KP297676	KP297777	KP297552	KP297646	KP297580	KP297711	KP297612	This study
Ingroup	*Abelia myrtilloides* Rehder	China, Sichuan, Wenchuan county	*S*. *L*. *Zhou 522* (PE)	KP297487	KP297743	-	-	KP297778	KP297553	-	KP297581	KP297700	KP297613	This study
Ingroup	*Abelia parvifolia* Hemsl.	China, Yunnan, Kunming	*S*. *L*. *Zhou 523* (PE)	KP297488	KP297744	KP297532	KP297677	KP297779	KP297554	KP297647	KP297582	KP297714	KP297614	This study
Ingroup	*Diabelia serrata* (Siebold and Zucc.) Landrein	Japan, Yamanashi	*T*. *Funamota 524* (PE)	KP297489	KP297745	KP297519	KP297678	KP297780	KP297555	KP297649	KP297584	KP297701	KP297616	This study
Ingroup	*Diabelia serrata* (Siebold and Zucc.) Landrein	China, Zhejiang, Yongjia Sihaishan mountain	*S*. *L*. *Zhou 525* (PE)	KP297490	KP297746	KP297520	KP297679	KP297781	KP297556	KP297650	KP297585	KP297702	KP297617	This study
Ingroup	*Diabelia tetrasepala* (Hara et Kurosawa) Landrein	Japan, Yamanashi	*T*. *Funamota 526* (PE)	KP297492	KP297748	KP297522	KP297680	KP297783	-	KP297652	KP297587	KP297703	KP297619	This study
Ingroup	*Abelia uniflora* R. Brown	China, Fujian, Wuyishan	*S*. *L*. *Zhou 533* (PE)	KP297509	KP297767	KP297541	KP297681	KP297801	-	KP297648	KP297583	KP297704	KP297615	This study
Ingroup	*Abelia × grandiflora (Rovelli exAndré) Rehder*	Japan, Yamanashi	*T*. *Funamota 534* (PE)	KP297498	KP297756	KP297540	KP297682	KP297790	KP297563	KP297657	KP297578	KP297710	KP297624	This study
Ingroup	*Kolkwitzia amabilis Graebner*	China, Beijing, Institute of Botany	*S*.*L*. *Zhou 537 (PE)*	EU240666	KP297752	KP297524	KP297683	KP297786	KP297559	KP297654	KP297589	KP297713	KP297621	This study
Ingroup	*Diabelia spathulata* (Siebold and Zucc.) Landrein	Japan, Yamanashi	*T*. *Funamota 559* (PE)	KP297491	KP297747	KP297521	KP297687	KP297782	KP297557	KP297651	KP297586	KP297724	KP297618	Bad DNA
Ingroup	*Abelia forrestii* (Diels) W.W.Sm	China, Yunnan, Zhongdian, Hutiaoxia	*S*. *L*. *Zhou AB04* (PE)	KP297481	KP297737	KP297535	KP297688	KP297772	KP297546	KP297640	KP297574	KP297705	KP297607	This study
Ingroup	*Linnaea borealis* subsp. *borealis* L.	China, Heilongjiang, Tahe county	*W*. *C*. *Hou AB10 (PE)*	KP297496	KP297754	KP297512	KP297689	KP297788	KP297561	KP297655	KP297591	KP297699	KP297622	This study
Ingroup	*Vesalea grandifolia* (Villarreal) H.F. Wang and Landrein	Mexico, Queretaro, La lagunita de San Diego	*S*. *Landrein (K) # 438*(K)	KP297503	KP297761	KP297531	KP297690	KP297795	KP297565	KP297662	KP297597	KP297706	KP297628	This study
Ingroup	*Vesalea floribunda* M.Martens and Galeotti	Mexico, Veracruz, Tlacotiopa	*S*. *Landrein # 2656* (K)	-	KP297760	KP297530	KP297691	KP297794	-	KP297661	KP297596	KP297719	KP297627	This study
Ingroup	*Abelia forrestii* (Diels) W.W.Sm	China, Yunnan, Nujiang	*S*. *Landrein #2051* (K)	KP297482	KP297738	KP297529	KP297692	KP297773	KP297547	KP297641	KP297575	KP297720	KP297608	This study
Ingroup	*Vesalea coriacea var*. coriacea (Hemsl.) T.Kim and B.Sun ex Landrein	Mexico, San Luis Potosi	*S*. *Landrein # 406* (K)	KP297500	KP297758	KP297527	KP297693	KP297792	-	KP297659	KP297594	KP297721	KP297625	This study
Ingroup	*Vesalea occidentalis* (Villarreal) H.F. Wang and Landrein	Mexico, Durango, Reserva la Michilia	*S*. *Landrein # 500* (K)	KP297505	KP297763	KP297528	KP297694	KP297797	KP297567	KP297664	KP297599	KP297722	KP297629	This study
Ingroup	*Vesalea mexicana* (Villarreal) H.F. Wang and Landrein	Mexico, Oaxaca, 10 km NE of Chicahuaxtla	*Breedlove*, *D*. *E*. *# 2232* (K)	KP297504	KP297762	KP297537	KP297695	KP297796	KP297566	KP297663	KP297598	KP297707	-	This study
Ingroup	*Vesalea coriacea var*. *subcoriacea* (Hemsl.) T.Kim and B.Sun ex Landrein	Mexico, Nuevo Leon, road from Los Lirios to Cola de Caballo ‘San Isidro Canyon’	*Fairey*, *J*. *# s*.*n*. *(K)*	KP297501	KP297759	KP297536	KP297696	KP297793	-	KP297660	KP297595	KP297708	KP297626	This study
Ingroup	*Linnaea borealis* subsp. *borealis* L.	Finland, Turku, Hallinen	*M*. *Chritenhusz 6026* (H)	KP297497	KP297755	KP297539	KP297697	KP297789	KP297562	KP297656	KP297592	KP297709	KP297623	This study
Ingroup	*Linnaea borealis* subsp.*longiflora* (Torr.) Hultén	Cultivated in Kew	*S*. *Landrein 25460* (K)	KP297498	KP297756	KP297540	KP297698	KP297790	KP297563	KP297657	KP297593	KP297710	KP297635	This study
Ingroup	*Dipelta floribunda* Maxim.	China, Gansu	*Pyck 1978–4099* (KU)	GU168628	GU168700	GU168647	HQ680740	-	-	-	-	-	-	Jacobs et al. 2010;Landrein et al. 2012
Ingroup	*Linnaea borealis* subsp.*americana* (Forbes) Hultén ex Clausen	Door County, Wisconsin	Donoghue, 1990, voucher lacking	AY236181	GU168706	HQ693930	HQ680732	-	-	-	-	-	-	Bell et al. 2004;Jacobs et al. 2010; Landrein el al 2012
Ingroup	*Dipelta elegans* Batalin	China, Gansu	*Z*.*L*. *Liu* 223 (Northeast University)	KC464764	KC464769	-	KC464765	-	-	-	-	-	-	Liu et al. 2013
Outgroup	*Acanthocalyx alba* (Hand.-Mazz.) M. Cannon	China, Yunnan, Jisha	*Boufford et al*. *28401* (A)	AY236183	-	AF446913	AF446943	-	-	-	-	-	-	Bell 2004; Zhang et al. 2003; Bell et al. 2002
Outgroup	*Cryptothladia chinensis* (Pai) M. Cannon	China, Qinghai, Dari	*Boufford et al*. *27846* (A)	AY236184	AF366925	AF446914	AF446944	-	-	-	-	-	-	Bell 2004; Zhang et al. 2003; Bell et al. 2002
Outgroup	*Morina longifolia* Wallich ex DC.	Cult. Bergius Bot. Gard., Sweden;	*Eriksson s*.*n*., *2 Nov*. 1999 (SBT)	AY236185	AF446975	AF446915	AF446945	-	-	-	-	-	-	Jacobs et al. 2010; Bell et al. 2002; Bell 2004; Bremer et al. 2002
Outgroup	*Zabelia buddleioides* (W.W.Sm.) Hisauti and Hara	China, Yunnan, Zhongdian, Hutiaoxia	*S*. *L*. *Zhou 485* (PE)	KP297507	KP297765	KP297525	KP297669	KP297799	-	KP297665	KP297601	KP297728	BOP012222	This study
Outgroup	*Zabelia dielsii* (Graebner) Makino	China, Shanxi, Jishan county, Xishezhen	*S*. *L*. *Zhou 491* (PE)	KP297508	KP297766	KP297526	KP297672	KP297800	KP297569	KP297666	KP297602	KP297731	BOP012228	This study
Outgroup	*Heptacodium miconioides* Rehder	China, Zhejiang, Haizhou Botanical Garden	*S*.*L*. *Zhou 536* (PE)	-	KP297751	-	-	-	-	-	-	-	BOP012292	This study
Outgroup	*Weigela florida* (Bunge) A. DC	Japan, Yamanashi	*T*. *Funamota 540*.*1* (PE)	KP297506	KP297764	KP297538	KP297684	KP297798	KP297568	-	KP297600	KP297730	BOP012296	This study
Outgroup	*Symphoricarpos sinensis* Rehder	China, Yunnan, Kunming institute of Botany	*S*. *L*. *Zhou 542* (PE)	KP297499	KP297757	KP297511	KP297685	KP297791	KP297564	KP297658	-	KP297732	BOP012300	This study
Outgroup	*Leycesteria formosa* Wallich	China, Yunnan, Lijiang Yuhu	*S*. *L*. *Zhou 543* (PE)	KP297495	KP297753	KP297510	KP297686	KP297787	KP297560	-	KP297590	KP297729	BOP012301	This study
Outgroup	*Lonicera involucrata* (Richardson) Banks ex Spreng.	National Botanic Garden Belgium	*Pyck 53–6481*,Belgium,	EU265584	GU168629	GU168650	-	EU265358	-	-	-	-	-	Jacobs et al. 2010; Theis et al. 2008
Outgroup	*Leycesteria crocothyrsos* Airy Shaw	National Botanic Garden Belgium	*Pyck 1992–1691*, Belgium	AF265277	GU168704	FJ745393	-	EU265328	-	-	-	-	-	Theis, et al. 2008, unpublished; Gould and Donoghue, 2000, unpublished; Jacobs et al. 2010
Outgroup	*Triosteum perfoliatum L*.	USA, Southern Indiana	-	AF265291	GU168717	GQ284972	AJ420871	EU265335	-	-	-	-	-	Gould and Donoghue,unpublished; Jacobs et al. 2010; Bell, 2010; Donoghue et al. 2001; Theis et al. 2008

### Sampling

The chloroplast fragments were chosen amongst the core DNA barcodes for land plants and also from the most variable plastid regions previously used in Caprifoliaceae [39]. *Abelia* is the most taxa-rich genus in Linnaeoideae. Rehder [26] recognized 13 species; Hu [37] accepted five species; Yang and Landrein [28] accepted three species and a species complex which includes all species with two sepals. In order to test relationships among taxa of the two-sepal group, we distinguished five names in this publication (*A*. *macrotera*, *A*. *myrtilloides*, *A*. *engleriana*, *A*. *uniflora* and *A*. *parvifolia*: specimens were selected and identified by S. L. Zhou). A total of 32 accessions of Linnaeoideae representing seven species of *Abelia*, five species of *Vesalea* [35,36,74], three species of *Dipelta*, three subspecies of *Linnaea* and the only species of *Kolkwitzia* were collected from China, Finland, Japan, and Mexico; 12 accessions representing ten outgroup genera were also added (Table 2). The voucher information and GenBank accession numbers are given in Table 2.

Based on previous analyses [19,24,40,70], we included *Heptacodium*, *Leycesteria*, *Lonicera*, *Symphoricarpos* and *Triosteum* in Caprifolioideae, *Morina* and *Acanthocalyx* in Morinoideae, *Weigela* in Diervilloideae plus unplaced *Zabelia* as outgroups for this study.

### DNA extraction, amplification and sequencing

Total DNA was extracted from silica gel-dried leaf tissue using the modified Cetyltrimethyl Ammonium Bromide (mCTAB) method [41]. Approximately 20 mg of dried plant tissue was used per extraction. DNA fragments were amplified and sequenced using the primers suggested by Olmstead and Palmer [42] for *rbc*L, Sun et al. [43] for *mat*K, Taberlet et al. [44] for *trn*L-F, Shaw et al. [45] for *trn*S-G, and Sun et al. [46] for ITS. Primers for *ndh*A, *trn*D*-psb*M, *pet*B-D, *trn*L*-rpl*32 and *trn*H*-psb*A are from Dong et al. [39]. Each polymerase chain reaction amplification was carried out in a 25 μL volume with the following reagents: Taq polymerase buffer, 10–50 ng total genomic DNA, 2.0 μM MgCl_2_, 0.4 μM each of both forward and reverse primers, 0.25 μM each dNTP, and 2 units of Taq DNA polymerase (Takara Biotechnology Co., Dalian, China). The thermal cycling conditions were 3 min at 94°C, followed by 35 cycles of 30 s at 94°C, 40 s at 52°C and 1.5 min at 72°C, with a final extension of 10 min at 72°C. The obtained PCR products were purified with PEG8000 and sequenced using ABI Prism BigDye Terminator Cycle Sequencing Kits v. 3.1 on an ABI 3730xl DNA Analyzer (Life Technologies, 5791 Van Allen Way, Carlsbad, California 92008, USA) following the manufacturer’s instructions.

### Phylogenetic analyses

The sequences were edited and assembled using Sequencher v. 4.7 (Gene Codes Corporation, Ann Arbor, Michigan, USA). The resulting sequences were combined with those downloaded from GenBank, aligned using Clustal W implemented in Mega version 6.0 software [47] and manually adjusted using Se-Al 2.0 [48]. Prior to concatenating the dataset of each marker, incongruence length difference (ILD) tests were performed on all ten datasets. The datasets were finally concatenated using SequenceMatrix [49].

Phylogenetic analyses were performed using PAUP* v4b10 [50] for maximum parsimony (MP), RAxML [51] for maximum likelihood (ML) analyses, and MrBayes 3.2.2 [52] for Bayesian inference (BI). The MP analyses used heuristic searches with 1,000 random addition sequence replicates, tree bisection reconnection (TBR) branch swapping, and MULTREES on. All character states were treated as unordered and equally weighted with gaps treated as missing data. To evaluate the relative robustness of clades in the MP trees, the bootstrap analysis [99] was performed with 1 000 replicates using the same options as above except that a maximum of 100 trees were saved per replicate.

MrModeltest 3.7 [100] was run for each of the data sets to determine a model of sequence evolution. The models chosen under the Akaike information criterion (AIC) were used in the ML and BI analyses [108] (see the last row of Table 3). For the ML analyses, ten independent runs were conducted using automatic termination following 20 000 generations without a significant (lnL increase of 0.01) topology change. To estimate the support for each node, 1 000 bootstrap replicates were performed with automatic termination at 10 000 generations, All final runs were performed on the CIPRS Science Gateway (http://www.phylo.org/portal2/) [53].

**Table 3:** MP analysis statistics with 1000 replications of internal transcribed spacer (ITS) and nine plastid regions.

		ITS	*mat*K	*rbc*L	*trn*L-F	*trn*S-G	*ndh*A	*pet*B-D	*psb*A*-trn*H	*psb*M-*trn*D	*trn*L-*rpl*32	Plastid
Best tree length (L)	Ingroup	86	26	89	42	145	37	46	73	37	370	511
	All taxa	442	171	165	143	254	257	106	322	180	370	1 898
Length of aligned matrices (Bp)	Ingroup	637	779	532	857	898	1 110	1 204	806	1 186	1 032	8 404
	All taxa	638	779	610	858	929	1 164	1 204	843	1 213	1 158	8 758
Nucleotide diversity (π)	Ingroup	0.019 2	0.005 4	0.009	0.008	0.010 6	0.008	0.01	0.02	0.006	0.02	-
	All taxa	0.045 4	0.024 9	0.012	0.019	0.027	0.015	0.01	0.02	0.01	0.03	-
Number of constant characters	Ingroup	572	754	572	817	830	1079	1171	761	1149	948	7249
	All taxa	401	635	535	732	716	955	1122	622	1052	885	7300
Number of potentially parsimony-Informative characters (Nc)	Ingroup	40	16	14	21	40	21	20	31	19	53	230
	All taxa	135	81	25	80	89	66	35	77	41	122	596
Percentage of potentially parsimony-informative sites	Ingroup	6.27	2.05	2.3	2.45	4.31	1.89	1.66	3.85	1.6	5.14	3.01
	All taxa	21.19	10.4	4.7	9.33	9.91	5.67	2.91	9.13	3.38	10.5	6.81
Consistency index (CI)	Ingroup	0.814	1	0.528	0.976	0.703	0.838	0.74	0.7	1	0.85	0.81
	All taxa	0.72	0.89	0.5	0.93	0.87	0.87	0.81	0.77	0.89	0.87	0.86
Retention index (RI)	Ingroup	0.91	1	0.48	0.99	0.7	0.94	0.86	0.83	1	0.94	0.91
	All taxa	0.79	0.93	0.34	0.97	0.92	0.89	0.84	0.82	0.94	0.9	0.88
Model selected by AIC		GTR+G	GTR+G	HKY+G	GTR+G	HKY+G	GTR+G	HKY+G	HKY+G	GTR+G	GTR+G	-

A partitioned Bayesian analysis of the plastid dataset was also implemented by applying the previously determined models to each data partition [109]. For BI 40 million generations were run with four chains, each starting with a random tree. Trees were sampled every 1 000 generations. Posterior probabilities (PP) were calculated from the majority consensus of all the sampled trees. When the standard deviation of the split frequencies (SDSF) permanently fell below 0.01, the trees sampled during the burn-in phase were discarded. All final runs were performed on the CIPRS Science Gateway (http://www.phylo.org/portal2/) [53].

### Estimation of divergence times

Seven *Abelia*-like fruit fossils were reported from Late Oligocene to Middle Eocene [54]. The most reliable character to distinguish *Abelia* fossils is the shape of fruits. In the extant *Abelia*, the typical fruit is an oblong achene crowned with 2~5 persistent sepals. Crane [55] thought only *A*. *trialata*, *A*. *quadrialata*, *A*. *quinquealata* and one *Abelia* sp. were probably correctly determined, while three additional *Abelia*-like fossils were wrongly identified. Fruits of *A*. *quadrialata* and *A*. *trialata* have been found to have hypogynous rather than epigynous fruits [56]. Moreover, even if *Abelia*-like fossils are correctly determined, it is difficult to identify which genera they belong to. Due to this uncertainty, we did not use the *Abelia*-like fossils. Manchester and Donoghue [57] also discounted the *Abelia*-like fossils for their study.

Our tree was calibrated with three points. First, seeds of *Weigela* are known from the Miocene and Pliocene in Poland [101], the Miocene in Mammoth Mountain, Eastern Russia, the Oligocene and Miocene of Western Siberia [102,103], and the Miocene in Denmark [104]. Therefore, we set lognormal prior of the divergence between *Weigela* and its sister *Diervilla* at 23 Ma with mean = 0, SD = 1.0, offset = 23 Ma.

**Fig 2:**
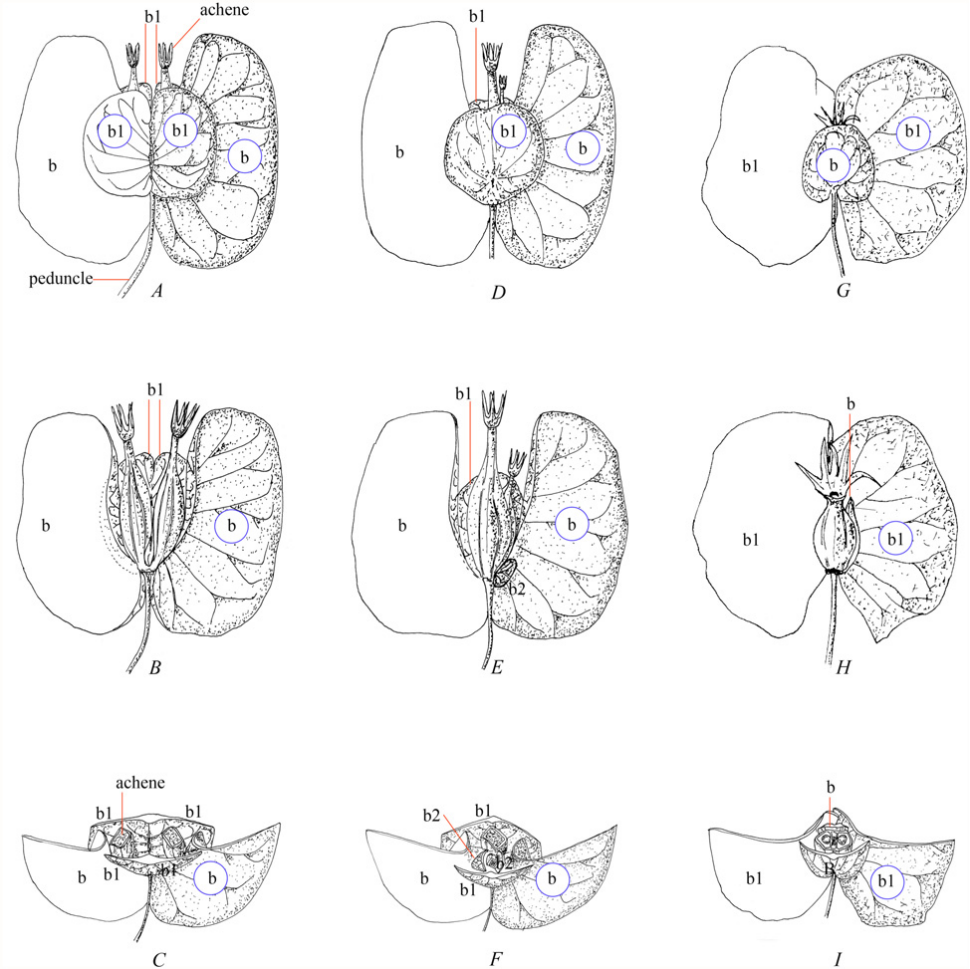
Theoretical reconstruction of *Diplodipelta* and *Dipelta* infructescences made of single or paired achenes and winged epicalyx bracts (according to phylogenetic data and inflorescence evolution theory) [25,57,80]. A-C, hypothetical reconstruction of infructescence with achenes of similar size. A, infructescence; B, one pair of fused prophyll-1 removed; C, cross section. D-F, hypothetical reconstruction of infructescence with achenes of different sizes. D, infructescence; E, one pair of fused prophyll-1 removed; F, cross section. G-I, hypothetical reconstruction of *Dipelta europaea* fossil infructescence. G, infructescence; H, one bract removed; I. cross section. b: bract; b1: bracteole-1; b2: bracteole-2.

Second, Manchester and Donoghue [57] described the fossil genus *Diplodipelta* from the late Eocene Florissant flora of Colorado (36–35 Ma), and from the Ruby, and Mormon Creek floras of Montana. The infructescence is made of two achene-like fruits of similar size enclosed by three bracts; two are wing-like, fused to the peduncle at base and the third one is hypothesized to be folded transversely and enveloping the two achenes. In order to interpret the *Diplodipelta* fossils we present two theoretical morphologies that could correspond to extant inflorescences in Linnaeoideae [25]. (1) The paired achenes could be of similar size (maturing simultaneously) and the infructescence similar to the extant genus *Diabelia* (Fig. 2A-C). (2) The paired achenes could be of different size (maturing consecutively) and the infructescence similar to the extant genus *Kolkwitzia* (Fig. 2D-F).

In both cases the epicalyx is theoretically formed of six prophylls of different origins (bracts, bracteole-1 and bracteole-2; see Landrein el al. [25] for terminology).

As shown by Manchester and Donoghue [57], it seems that paired achenes of similar sizes are the most plausible morphology. Nevertheless, this configuration does not allow the bracteole-1 (b1) to be larger than the bracts (b) like the situation in extant *Dipelta* species (Fig. 2G-I), and a fusion of the bracteole-1 in two pairs has to be hypothesized (this fusion can also be observed in extant species of the genus *Heptacodium* but not in Linnaeoideae). If *Diplodipelta* is related to the genus *Dipelta*, it is hard to explain why the bracts have reduced in size whereas the bracteole-1 became wing-like. The fact that the bracts of *Diplodipelta* are fused at the base to the peduncle could form an explanation. We thus differ from Manchester and Donoghue’s [57] reconstruction which only shows three bracts; the median wing in Fig. 8 p710 showing both sides is interpreted as a single folded bract which is slightly peltate at base and cordate at apex. Although we have not examined critical specimens of the fossil (e.g., Manchester and Donoghue’s Figs. 8, 10, 11 showing the connection between the front and back sides of the same specimen), our reinterpreted bract configuration of four bracteoles-1 fused in two pairs, slightly peltate on one side and cordate on the other side, seems to resemble extant Linnaeoideae fruit morphology (the bracteole-1 of *D*. *floribunda* is often either peltate or cordate).

When taking into account new phylogenetic results and inflorescence ontogenetic data, *Diplodipelta* infructescences could not be dissociated from Linnaeoideae but the fossil genus could also be sister to *Diabelia* as well as *Dipelta*. The stratigraphic record of *Diplodipelta*, together with the occurrence of genuine *Dipelta* fruits in the late Eocene of England and Mississippi [58], indicates that the divergence of these genera occurred during or prior to late Eocene [57]. We therefore consider the split of *Diplodipelta*, *Dipelta* and *Diabelia* fossils at about 36–35 Ma and set the stem of *Dipelta* with lognormal mean = 0, SD = 1.0, offset = 36 Ma.

Third, Caprifoliaceae is a family within eudicots, the oldest fossils of eudicots were recorded at about 125 Ma with their distinctive tricolpate pollen [75,105–107]. Bell and Donoghue [76] suggested that the Dipsacales originated by the mid-Cretaceous, well before previous age estimates for eudicots. They estimated the Dipsacales node to be 102–110 Ma. In this study the Dipsacales node (the root of our tree) was constrained to 103 Ma, with a normal prior, mean = 103 Ma, SD = 5, despite the lack of fossil evidence.

The estimation of divergence times was obtained using a Yule process speciation prior and an uncorrelated lognormal (UCLN) model of rate change with a relaxed clock [59]. The analyses were run for 30 million generations with parameters sampled every 1 000 generations. Trace files were loaded into Tracer v.1.5 [60] to look for an effective sampling size (ESS), and to examine the posterior distributions of all parameters and their associated statistics including 95% highest posterior density (HPD) intervals. Initially to optimize efficiency in BEAST, we undertook several trial runs of 10–20 million generations and analyzed the results using Tracer v.1.5 [60]. These results were then used to determine the number of generations necessary to achieve the desired ESS of at least 200 and to optimize the operator settings for our abovementioned final analysis. The program Tree Annotator v. 1.8.0 [60] was used to summarize the set of post burn-in trees and their parameters (burn-in set to 4 000), to produce a maximum clade credibility (MCC) chronogram showing mean divergence time estimates with 95% HPD intervals. FigTree v.1.3.1 [61] was used for visualization of the resulting divergence times.

### Biogeographic analyses

As with many other genera endemic to China [62], the species diversity of Linnaeoideae is high in western China along the Qinghai-Tibetan plateau and low in eastern China. Five areas of endemism were defined to cover the distribution ranges of Linnaeoideae and its relatives as follows (Fig. 1) based on the distribution of taxa in the subfamily: (A), eastern and northern Asia: Japan, eastern China (Anhui, Fujian, Guangdong, Henan, Hunan, Hubei, Jiangsu, Jiangxi, Shanxi, Taiwan, and Zhejiang provinces), Korea, the Far East of Russia, and the adjacent regions; (B), central and western China (Chongqing, Gansu, Guangxi, Guizhou, Shaanxi, Sichuan, and Yunnan provinces); (C), Europe; (D), North America; and (E), Mexico.

The distribution of each species was assigned to at least one of these regions. The ancestral distributions were inferred using a likelihood approach under the dispersal-extinction-cladogenesis (DEC) model implemented in Lagrange [68]. Python scripts were generated using the online Lagrange configurator (http://www.reelab.net/lagrange/configurator). The MCC tree from BEAST analysis was used as the input tree. The probability of dispersal between areas was modeled as equal, and all values in the dispersal constraint matrix were set to 1.

Many recent studies have incorporated fossils into biogeographic reconstruction [e.g., 63–67]. The ancestral distribution was thus optimized with Lagrange using the phylogeny of the extant species inferred from the combined data set with and without reliable *Dipelta* and *Diplodipelta* fossil taxa.

(i) The fossil of *Dipelta europaea* was found in southern England in the late Eocene to early Oligocene [80]. We incorporated the age 32.8 Ma as the time of occurrence of *D*. *europaea* in Europe into the BEAST tree.
(ii) The fossil *Dipelta* sp. was reported from the Eocene of Mississippi [58]. The age 33.5 Ma indicating the occurrence of *Dipelta* sp. in North America was incorporated into the BEAST tree.
(iii) *Diplodipelta* fossil was described by Manchester and Donoghue [57] from the late Eocene Florissant flora of Colorado. The age 37 Ma was incorporated to indicate the position of *Diplodipelta* as sister to the *Diabelia*—*Dipelta* clade in the BEAST tree.

Given that the fossil of *Diplodipelta* may be sister to *Diabelia* or *Dipelta*, we made two alternative estimations: (1) sister relationship between *Diplodipelta* and *Dipelta* in the BEAST tree, and (2) sister relationship between *Diplodipelta* and *Diabelia* in the BEAST tree.

## Results

### Sequence variability within Linnaeoideae

Length of aligned matrices, number of constant characters and potentially parsimony-informative characters, as well as consistency and retention indices of the nine chloroplast regions and the nuclear ribosomal ITS are summarized in Table 3. Of the nine chloroplast regions, *trn*L*-rpl*32 is the most variable fragment with a π (nucleotide diversity) value of 0.020 35, while *psb*M*-trn*H is the least variable fragment with a π value of 0.005 4. The concatenated length of the nine chloroplast regions reached 7641 bp with 230 parsimony-informative characters. ITS is also very variable in Linnaeoideae with π = 0.019 2 and 40 parsimony-informative characters.

### Phylogenetic relationships

**Fig 3:**
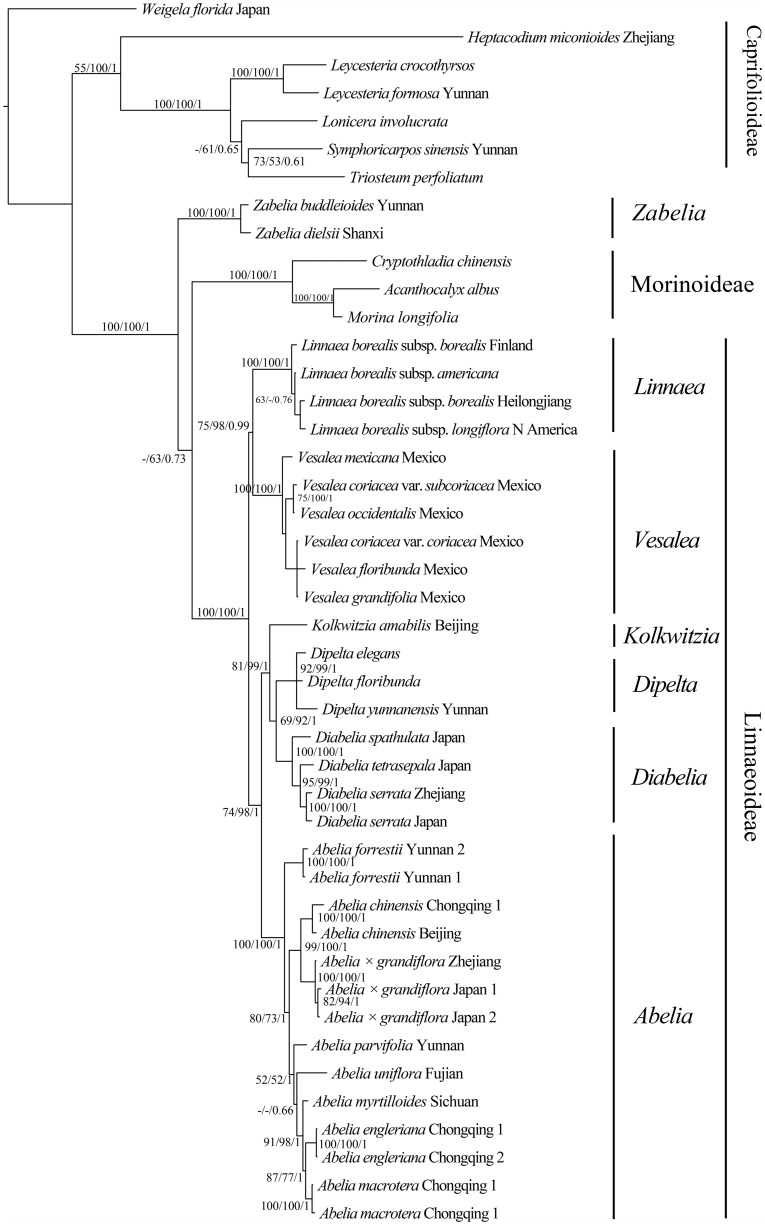
Bayesian tree of Linnaeoideae and outgroups based on the combined *rbc*L, *trn*S-G, *mat*K, *trn*L-F, *ndh*A, *trn*D*-psb*M, *pet*B-D, *trn*L*-rp*L32 and *trn*H-*psb*A sequence data. MP (first) and ML (middle) bootstrap branch support and Bayesian posterior probabilities (last) are indicated above a cut-off value of 50 and 0.5, respectively.-indicates bootstrap value < 50%.

The concatenated plastid markers resolved the tree topologies well at generic level while polytomies existed on all single marker trees. The tree topologies based on different markers were similar. One exception is the tree based on *trn*L-F which showed sistership between *Zabelia* and *Morina* + *Cryptothladia*, while other markers suggested a more basal position of *Zabelia*. ILD tests showed that ITS is incongruent with the nine plastid markers at a significant level (*p* = 0.01). Thus, we only concatenated all nine plastid markers to build better resolved phylogenetic trees using MP, ML and BI methods (Fig. 3, S1 Fig., and S1 Dataset).

The monophyly of Linnaeoideae is strongly supported (PB = 100, LB = 100, PP = 1; Fig. 3). Within the subfamily the monophyly of each of the narrowly circumscribed genera is also strongly supported. *Zabelia* is shown to be a sister group to Morinoideae (PB = 100, LB = 100, PP = 1; Fig. 3) in agreement with previous study from Jacobs et al. [23]. The resolution within Mexican species of *Vesalea* and Chinese *Abelia* species is low.

### Divergence times of major lineages

**Fig 4:**
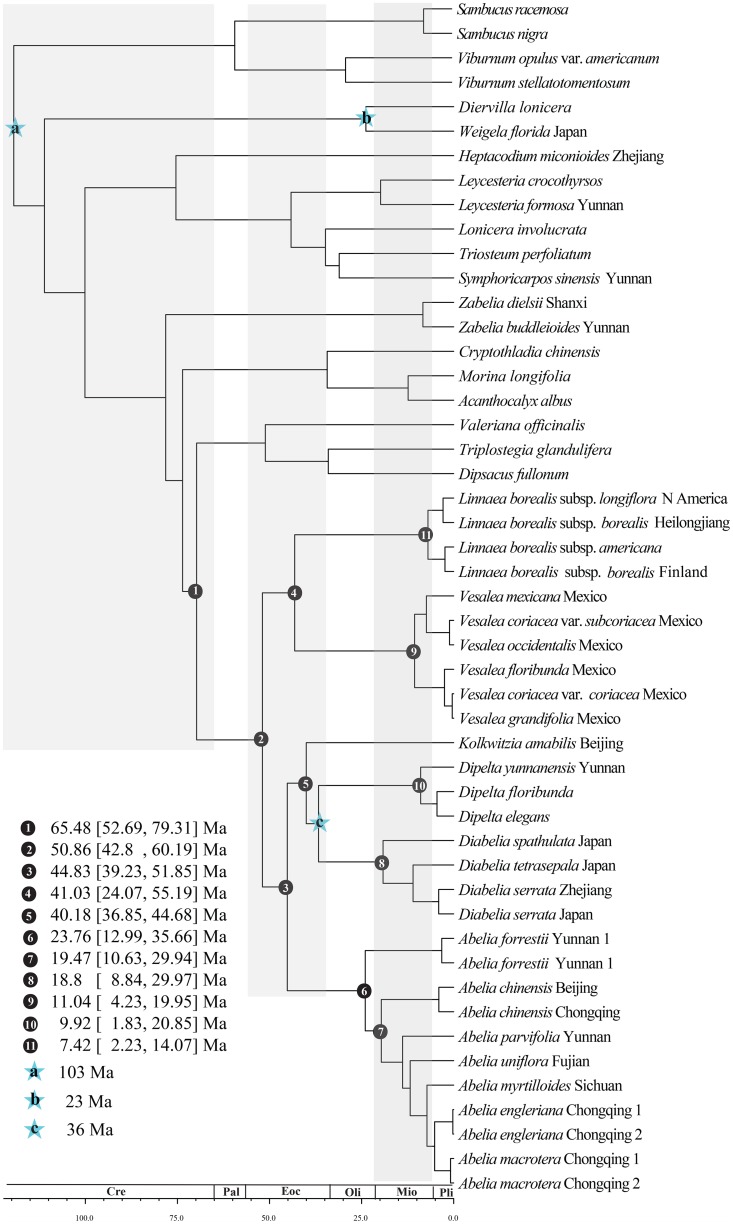
Chronogram of Linnaeoideae and outgroups based on nine plastid sequence combined data estimated from BEAST. Calibration points are indicated by stars.

The inferred divergence times of Linnaeoideae and its lower ranks are shown in Fig. 4. The crown group of Linnaeoideae was estimated at 50.86 (95% HPD 43.39–63.23) Ma from the Paleocene to early Eocene. Almost all genera of Linnaeoideae had diverged in the Eocene, but the divergences of extant species are inferred to have occurred mostly in the Miocene and Pliocene. According to our estimates, *Linnaea* split from *Vesalea* at around 41.03 (95% HPD 24.07–55.19) Ma in the middle Eocene, and *Kolkwitzia* split from *Dipelta* at 40.18 (95% HPD 36.85–44.68) Ma. The crown group of *Abelia* was dated at 23.76 (95% HPD 12.99–35.66) Ma, and the divergence of *Abelia chinensis* with other *Abelia* species was dated at 19.47 (95% HPD 10.63–29.94) Ma. The crown groups of *Vesalea*, *Dipelta* and *Linnaea* were estimated at 11.04 (95% HPD 4.23–19.95) Ma, 9.92 (95% HPD 1.83–20.85) Ma and 7.42 (95% HPD 2.23–14.07) Ma, respectively (Fig. 4).

### Biogeography of Linnaeoideae

**Fig 5:**
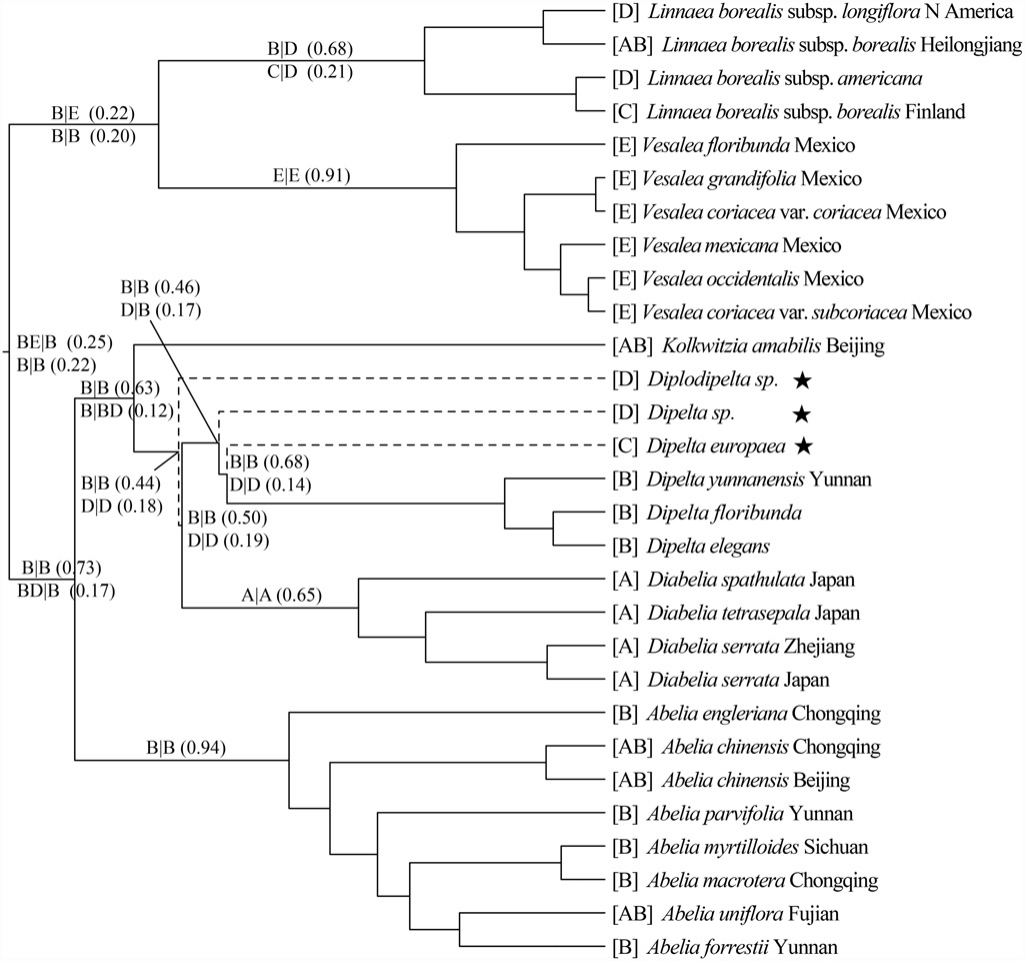
Ancestral area reconstruction of Linnaeoideae using Lagrange including three fossils, in which *Diplodipelta* was placed as sister to the *Dipelta*—*Diabelia* clade. The tree was based on a 50% majority-rule BI consensus tree. For the Lagrange results, a slash indicates the split of areas into two daughter lineages, i.e., left/right, where “up” and “down” are the ranges inherited by each descendant branch. The values in brackets represent relative probabilities.

Given *Diplodipelta* might be the common ancestor of *Dipelta* or *Diabelia*, we placed *Diplodipelta* as sister to the *Dipelta—Diabelia* clade. In this scenario, our Lagrange analyses reconstructed the ancestral area of the Linnaeoideae in western China as well as central and western China plus Mexico (BE|B with 0.25 relative probability, Fig. 5). Without incorporating fossils, our Lagrange analysis reconstructed the ancestral area of Linnaeoideae in western China as well as central and western China plus Mexico (BE|B with 0.52 relative probability, S2 Fig.).

However, we could not exclude *Diplodipelta* from being at the lower nodes of the BEAST tree. Therefore, *Diplodipelta* was placed alternatively as sister to *Dipelta* or *Diabelia*. When placed with *Dipelta*, our Lagrange analysis reconstructed the ancestral area of the Linnaeoideae in western China as well as central and western China plus Mexico (BE|B with 0.30 relative probability, S3 Fig.); when placed instead with *Diabelia*, the Lagrange analysis reconstructed the ancestral area of the Linnaeoideae in western China as well as central and western China plus Mexico (BE|B with 0.32 relative probability, S4 Fig.).

## Discussion

### Phylogenetic relationships in Linnaeoideae

Rehder [26] divided taxa now recognized in Linnaeoideae into two major groups, sect. *Abelia* and sect. *Zabelia*. *Abelia* sect. *Zabelia* was later raised to a generic level in 1948 by Makino [27]. The generic name *Zabelia* was however, rarely used in later publications and recently Yang and Landrein [28] used it for the Flora of China. Recent molecular studies have shown that *Zabelia* is closer to Morinoideae or Valerianoideae than to Linnaeoideae [23]. This study also shows *Zabelia* is sister to the rest of Linnaeoideae (PB = 100, LB = 100, PP = 1; Fig. 3).

When *Zabelia* is excluded from the analyses, *Abelia* s.l. is still polyphyletic [25,38]. The removal of the species belonging to *Vesalea* M. Martens and Galeotti (= *Abelia* ser. *Vesalea* Zabel) and *Diabelia* Landrein (= *Abelia* ser. *Serrata* Graebner) left *Abelia* as a monophyletic genus.

Six monophyletic groups: *Abelia*, *Diabelia*, *Dipelta*, *Kolkwitzia*, *Linnaea*, and *Vesalea* are well supported in this study and the recently established genera *Diabelia* and *Vesalea* are also supported [38,69] (Fig. 3). *Diabelia* and *Vesalea* were formerly treated as part of the genus *Abelia* [23,73]. The strong morphological similarities among *Abelia*, *Diabelia* and *Vesalea* are probably due to convergent evolution, a common phenomenon among the mesic forest elements of the eastern Asian—North American disjunct plants [5, 6, 13]. Some distinct characters can nevertheless be observed concerning inflorescence architecture, corolla shape, and nectaries (Table 4). *Abelia*, *Diabelia* and *Vesalea* share three synapomorphies: accrescent calyx, reduced episepals (except in *V*. *floribunda* var. *foliacea*) and ovaries flattened dorsi-ventrally. *Kolkwitzia* and *Dipelta* also have ovaries flattened dorsi-ventrally but they have a well developed epicalyx and reduced calyx but this is clearly linked to dispersal mechanisms (Table 4).

**Table 4:** Diagnostic morphological characters of the genera in Linnaeoideae.

Taxon	Inflorescence	Flower arrangements and opening	Nectary	Corolla	Calyx	Epicalyx	Distribution
***Abelia***	Axillary and terminal on long shoots, many flowered	Single or paired and opening consecutively	Glandular hairs dense at base of corolla tube	Bilabiate to infundifuliform	2 or 5 sepals	4 or 6 small and non accrescent	China-Japan (S islands)
***Diabelia***	Terminal on short shoots reduced to two flowers	Paired and terminal, opening simultaneously	Glandular hairs dense at base of corolla tube, sometimes cushion like.	Bilabiate	2, 3, 4 or 5 sepals	6 small and non accrescent	Japan- E China (Zhejiang)
***Dipelta***	Terminal thyrses on short shoots	Single	Glandular hairs dense at base of corolla tube	Bilabiate	5 sepals	4 with 2 large accrescent bracts becoming wing-like	China
***Kolkwitzia***	Terminal thyrses on short shoots	Single or paired and opening consecutively	Glandular hairs dense at base of corolla tube	Bilabiate,	5 sepals	4 or 6 accrescent, becoming inflated and sclerified.	China
***Linnaea***	Raceme-like on short shoot, reduced to one pair of flowers.	Single	Glandular hairs more or less dense at base of the corolla tube	Campanulate to infundibuliform	5 sepals deciduous	4 with 2 large accrescent bracts and covered with large stalked glandular hairs.	Widely distributed in alpine and cold regions of the N Hemisphere
***Vesalea***	Raceme-like on short shoots, few flowered.	Single or paired and opening consecutively	Glandular hairs spread along one to three lines in between the filaments.	Tubular to infundibilform and bilabiate	5 sepals	4 or 6, small to large and non accrescent.	Mexico
***Zabelia***	Congested terminal thyrse of 1–3 flowered sessile cymes	Paired and terminal, opening simultaneously or in 3-flowered cymes.	Glandular hairs spread along one to three lines in between the filaments.	Hypocrateriform	4 or 5 sepals	6 small to large and non accrescent, sometimes leaf-like.	Afghanistan, China, NW India, Japan, Korea, Kazakhstan, Kyrgyzstan, Nepal, Far East Russia

The clade formed by the circumboreal *Linnaea* and the Mexican *Vesalea* seems surprising at first because *Linnaea* has very distinct morphological features like the creeping habit, paired flowers and specialized epicalyx bracts. Nevertheless *Vesalea* and *Linnaea* share several morphological synapomorphies (Table 4): (1) raceme-like inflorescences with few flowers, forming on short shoots and appearing in the spring; (2) nectary which is not forming a bulge at the base of the corolla tube but a zone of dense glandular hairs in between the abaxial filaments, and (3) similar creeping habit between *Linnaea* and *Vesalea floribunda* except in dry conditions.

Divergent characters could be explained by an adaptation to their different environments. The two genera vicariously occupy the Rocky Mountains and the Sierra Madre Oriental, with extant populations being only separated by a few hundred kilometers.

All six genera have distinct morphological characters that have been described in detail by some taxonomists [71,72]. However, there are species problems within *Abelia* and *Vesalea*. The 5-sepaled species of *Abelia* (*A*. *chinensis* and *A*. *forrestii*, with the former occupying eastern and southern China, and the latter restricted to a small area in northwestern Yunnan and southwestern Sichuan) are well resolved, but the 2-sepaled species (*A*. *macrotera*, *A*. *myrtilloides*, *A*. *engleriana*, *A*. *uniflora* and *A*. *parvifolia*) are poorly delimited. Yang and Landrein [28] treated the latter group as the *A*. *uniflora* species complex. This study suggests that most of the 2-sepaled taxa of the genus are very closely related. Similarly to the 2-sepaled *Abelia* species, the species in *Vesalea*, which are 5-sepaled, are poorly resolved. Five species, *V*. *floribunda*, *V*. *coriacea*, *V*. *grandifolia*, *V*. *mexicana* and *V*. *occidentalis*, have been considered to occur in Mexico [74]. Many species within *Vesalea* are also difficult to separate morphologically.

Linnaeoideae is a group of shrubby or small tree species producing achenes; these fruits are adapted for wind dispersal in the genera *Abelia*, *Diabelia*, *Dipelta* and *Vesalea*. Achenes of *Diabelia serrata* and *Abelia uniflora* only possess two sepals instead of five. In *Dipelta* the wings originate from the epicalyx and two large bracts are present allowing for wind dispersal. In *Kolkwitzia* the spiny achenes are surrounded by corky episepals [25] and the fruits are called Hedge-Hog in Chinese, suggesting possible animal dispersal by clinging to animal fur. Finally in *Linnaea* the calyx is deciduous in fruit but two of the episepals are covered by large and numerous sticky glandular hairs. Fruit dispersal adaptations, number of sepals and episepals do not seem good indicators of systematic relationships and this could be due to convergence.

### Biogeography of Linnaeoideae

**Dipelta *and* Diplodipelta *distribution***: The crown group for *Diabelia*, *Dipelta* and *Kolkwitzia* was dated at 40.18 (95% HPD 36.85–44.68) Ma in the middle Eocene (Fig. 4). The Lagrange analyses inferred that *Dipelta* originated in central and western China (B) in the Eocene (Fig. 5). As discussed previously, the Linnaeoideae fruits and *Dipelta* in particular are adapted to wind dispersal; though it is not known how far they can travel, long distance dispersal events cannot be ascertained. In *Kolkwitzia* the achenes are possibly carried away in animal fur but long distance dispersal has not been tested. Starting in the Miocene, there was a distinct climatic cooling period across the high-latitude areas of the Northern Hemisphere, which may have resulted in a reduction of the distribution of forests [93]. *Dipelta* once reached southern England as well as the Mississippi region in the late Eocene, as evidenced from the fossil species *D*. *europaea* and *D*. sp., respectively [58,80]. The genus is now restricted to central and western China (B). Evidence for an early North American origin can be inferred from the fossil genus *Diplodipelta* with its *Dipelta*-like infructescences that existed in the late Eocene of western North America [57]. *Diplodipelta* may represent a sister group of *Diabelia* and *Dipelta*. *Dipelta* is inferred to have been more broadly distributed in the Miocene (although no Miocene fossil occurrences are known), with occurrences in Europe and North America, although the genus is restricted to western China today. The Tertiary disjunct distribution of *Dipelta* between Europe and North America may be explained by extinctions in large parts of its former ranges. Extinction events could have extirpated the old stem relatives that diverged prior to the extant crown radiation, leaving a phylogeny that includes only extant taxa with long stems and species-rich crowns [94]. A remarkably long “temporal gap” occurs between the *Dipelta* stem and the beginning of the extant radiation in the early Miocene (Fig. 3).

The lack of fossils from paleobotanically rich deposits of Asia might mean that the ancestral area was not in Asia, but in Europe and/or North America. The lack of DNA from extirpated populations of Europe and North America may have given a false impression that the area of modern diversity is the area of origin. *Diplodipelta* would also possibly have occurred in the lower node of the BEAST tree, therefore, in this study, we discuss all possible scenarios on the likely phylogenetic position of *Diplodipelta*.

Reconstruction of ancestral areas with Lagrange including fossils (i.e., *Diplodipelta* in three different positions of the BEAST tree) and without the fossils showed the same ancestral area for Linnaeoideae (c.f. Fig. 5, S2 Fig., S3 Fig., and S4 Fig.), which suggested an ancestral distribution and early diversification of Linnaeoideae in central and western China as well as central and western China plus Mexico, and subsequent dispersal into eastern Asia, Europe as well as into North America and Mexico.

The incorporation of fossils had little impact on the ancestral area of Linnaeoideae in this study (c.f. Fig. 5, S2 Fig., S3 Fig., and S4 Fig.). This may be due to the fact that these fossils are deeply nested within a clade which is now only found in central and western China, while the *Vesalea* plus *Linnaea* clade did not incorporate any fossil. This resulted in the same ancestral area for the four scenarios (Fig. 5, S2 Fig., S3 Fig., and S4 Fig.). Nevertheless, all Lagrange analyses had a comparatively low probability (less than 0.60) and did not clearly show the origin place for the subfamily (BE|B). A broader phylogenetic framework is also needed for Linnaeoideae and its close relatives.

The North Atlantic Land Bridges (NALB) [77] and the Bering Land Bridge (BLB) [78] have been hypothesized to have played important roles for the spreading of many intercontinental disjunct taxa of the Northern Hemisphere in the Tertiary [5,9,11–13]. NALB existed from the late Cretaceous to early Tertiary, which is an important migration channel for thermophilic plants in the Northern Hemisphere [11,79]. Similarly, BLB provided a stepping-stone migration route for high-latitude distributed (69–75°N) temperate plants from the Eocene to the present except for several temperature decreasing periods [5,12]. The NALB existed from the late Cretaceous to early Tertiary [12,79], and our dating and biogeographic results as well as the fossil records are consistent with a hypothesis of the migration of the *Diplodipelta*-*Dipelta*-*Kolkwitzia*-*Diabelia* clade from Eurasia to North America via NALB.

### *The circumboreal distribution of* Linnaea

*Linnaea borealis* is divided into three subspecies, subsp. *borealis* in Europe, Asia and Alaska; subsp. *longiflora* (Torr.) Piper & Beattie along the Pacific coast of western North America from Alaska to California, and subsp. *americana* (J. Forbes) Hultén in the rest of Canada and USA as well as Greenland [33,34]. The wide disjunct distribution of the monotypic genus *Linnaea* most likely represents an example of migration from Eurasia to North America via Beringia. *Linnaea borealis* is the only Linnaeoideae showing a continuous intercontinental extant distribution with populations along the Bering Strait islands, Chukotka and the Alaska Peninsula. *Linnaea borealis* is clearly the most cold-resistant species in Linnaeoideae and could have survived the conditions in the Bering Land Bridge area in the late Tertiary. Smith [81] concluded that the Caprifolieae clade originated within Asia and migrated around the Northern Hemisphere during the Cenozoic, including several migrations through the BLB. The Beringian route was also reported as a possible hypothesis for the disjunction between the East Asian *Weigela middendorffiana* and the North American *Diervilla* [82].

### Vesalea *and* Linnaea

Our BEAST and biogeographic analyses suggest that the *Linnaea-Vesalea* clade originated in central and western China (B) and Mexico (E) at 41.03 (95% HPD 24.07–55.19) Ma in the middle Eocene (Fig. 4). The Lagrange analysis supports dispersal from central and western China (B) to Mexico (E) as the explanation of the intercontinental disjunction between *Linnaea-Vesalea* and the rest of Linnaeoideae.

*Vesalea* and *Abelia* are both thermophilic genera, but *Linnaea* is well adapted to cold conditions. Two alternative hypotheses regarding the migration of the clade are consistent with our results. Unfortunately the lack of well preserved fossil for *Vesalea* as well as *Abelia* does not allow us to strongly favor one or the other.

First, since the genera of Linnaeoideae originated in the Eocene, migration through BLB seems likely. Tiffney and Manchester [63] argued that BLB may be too cold for the thermophilic plants in the late Tertiary. The Pleistocene glaciations disrupted gene flow and drove thermophilic species southward widening their genetic divergences. A migration of the *Linnaea-Vesalea* common ancestor through the BLB and subsequent radiation of *Vesalea* in Mexico cannot be excluded, as it allows both conditions to be met (cold resistant and thermophilic).

Second, the NALB might be a more likely route for the migration of the Mexican *Vesalea* or its common ancestor. *Vesalea* species were presumably more commonly distributed than its present range prior to the Pleistocene glaciations, and its perennial growth habit may have allowed it to survive the subsequent millennia locally within this former range in various high-elevation or otherwise cool and moist habitats in the highlands, like a few other Northern Hemisphere disjunct plants, such as *Aralia* L. [83], *Liquidambar* L. [84], *Platanus* L. [15], and *Toxicodendron* Mill. [85]. Many thermophilic disjunct plants of the Northern Hemisphere have been attributed to fragmentation of a once continuous belt of mixed mesophytic broadleaf-evergreen vegetation, i.e., the boreotropical flora [12,13,93] in the Northern Hemisphere. Remnants of the boreotropical floristic elements occur today in East Asia and eastern North America. The lineages that once grew in other areas became extinct by the late Eocene period due to a combination of climatic and geologic changes [5,13,41,93].

### Diabelia *diversification in the Sino-Japanese Floristic Region*

*Diabelia* is a widespread genus in Japan and is only recorded in one locality of Zhejiang province of East China [31,32]. The Sino-Japanese Floristic Region (SJRF) is a major region of plant diversity mostly composed of temperate deciduous forest in eastern China, Korea and Japan [86]. *Kolkwitzia*, *Dipelta* and *Diabelia* form a well-supported clade (81/99/1) (Fig. 3); their stem group was dated at 45.79 (95% HPD 39.04–53.52) Ma, and their crown group at 40.53 Ma in the Middle Eocene. The genera *Kolkwitzia* and *Dipelta* are endemic Chinese floristic elements and most diverse in the Qinling Mountains. *Diabelia* is a component of the Sino-Japanese floristic region and most diverse in Eastern China and Japan. About 63.8% of the genera of the Qinling range also occur in Japan [92]. *Kolkwitzia*, *Dipelta* and *Diabelia* could therefore represent a typical element of this flora which has also shown a slow decline and extinction of the genus *Diabelia* in East China. *Diabelia serrata* (collected in Zhejiang) and the same species collected in Japan could not be differentiated with the molecular markers we employed (100/100/1). It is therefore likely that *Diabelia* in China represents a relatively recent refugium following the last glacial event.

During glaciations in the Quaternary, the East China Sea level was lowered and a continuous belt of forests connected the now disjunct populations of East China, South Japan and Korea [87]. The East China Sea Land Bridge [90, 91] may have allowed dispersal and gene exchange between woodland species of East China, Korea and Japan. Examples such as *Cercidiphyllum japonicum* [88] and *Kalopanax septemlobus* [89] have been documented. However the East China Sea Land Bridge may also have acted as a ‘filter’ during the last glacial event for certain species and produced genetic differentiation among populations in South Japan, South Korea and East China. This is the case of many rare species such as *Platycrater arguta* [90] and *Kirengeshoma palmata* [91]. Despite those results we were not able to reproduce this scenario with the species *Diabelia spathulata* also growing in Zhejiang. Further phylogeographic studies using population genetic data may allow discovering whether a possible admixture, isolation or ‘filter’ event occurred in this species.

### Nomenclature

The following new combinations are made in light of the phylogenetic results;

*Vesalea occidentalis* (Villarreal) H.F. Wang & Landrein, comb. nov.

Basionym,—*Abelia occidentalis* Villarreal, Brittonia 49 (1), 84. 1997

Holotype,—Mexico. DURANGO, Mpio. Suchil, Reserva de la Michilia, Cienega Los Caballos, *Villarreal-Quintanilla*, *J*.*A*. *(with Carranza, N.A.) 8180* (MEXU).

*Vesalea grandifolia* (Villarreal) H.F. Wang & Landrein, comb. nov.

Basionym,—*Abelia grandifolia* Villarreal, Brittonia 52(2), 174. 2000

Holotype,—Mexico. QUERÉTARO, Mpio. de Jalpan, Cerro Grande, 13 June 1991, *Servin*, *B*. *1101* (CAS).

*Vesalea mexicana* (Villarreal) H.F. Wang & Landrein, comb. nov.

Basionym,—*Abelia mexicana* Villarreal, Brittonia 52 (2), 172. 2000

Holotype,—Mexico. OAXACA, Mpio. San Sebastian Tecomaxtlahuaca, *Calzada*, *J*.*I*. *21100* (MEXU).

*Vesalea coriacea* Hemsl. var. *subcoriacea* (Villarreal) H.F. Wang & Landrein, comb. nov.

Basionym,—*Abelia coriacea* Hemsl. var. *subcoriacea* Villarreal, Acta Bot. Mex. 102, 115, 2013

Holotype,—Mexico. COAHUILA, Mpio. Sierra Mojada, Sierra Mojada, Near Esmeralda, above San Salvador Mine, *Stewart*, *R*.*M*. *1081* (MEXU).

## Supporting Information

S1 Dataset Data matrix. The aligned sequence data as presented in nexus format.
(DOC)

S1 Fig Bayesian tree of Linnaeoideae and outgroups based on the internal transcribed spacer (ITS) sequence data. (PDF)

S2 Fig Ancestral area reconstruction of Linnaeoideae using Lagrange without fossil information. (PDF)

S3 Fig Ancestral area reconstruction of Linnaeoideae using Lagrange including three fossils, in which *Diplodipelta* was placed as sister to *Dipelta*. (PDF)

S4 Fig Ancestral area reconstruction of Linnaeoideae using Lagrange including three fossils, in which *Diplodipelta* was placed as sister to *Diabelia*. (PDF)
