# Supplementary material for: Molecular Phylogeny and Biogeographic Diversification of Linnaeoideae (Caprifoliaceae s. l.) Disjunctly Distributed in Eurasia, North America and Mexico
Source: PLoS One. 2015 Mar 10;10(3):e0116485. doi: 10.1371/journal.pone.0116485 (PMC4355296; doi:10.1371/journal.pone.0116485)
Supplement: S1 Dataset — The aligned sequence data as presented in nexus format. (DOC) [file pone.0116485.s001.doc]

#NEXUS

[Written by SequenceMatrix 1.7.8 on Tue Jun 17 22:37:19 CST 2014]

BEGIN DATA;

DIMENSIONS NTAX=44 NCHAR=8541;

FORMAT DATATYPE=DNA GAP=- MISSING=? ;

MATRIX

Abelia_chinensis_BOP012223 CCTGAGCCAAATCCAGTTTTACGA----AAACAAGGGTTCAGAAAGCTAAAATC-AAAAAGGATAGGTGCAGAGACTCAATGGAAGCTGTTCTAACAAATGGAGTTGACTGTGTTGTGTTGGTAGAAAGAATCCTTCCATAGAAACTTCAGAAAGGATAAACGTATAAACATGGATATACGCATTGAAATACTAT-ATACTCTACCAAATGATTAATGACGATCCGAATCTGTATT--TA-----TATATATCAAAATGGGAGAATGGTTGTGAAGTGATTCCATATTGAAGAAAGAATCGAATATTTATTGATCAAATCATTCACTCCATAGTCTGATAGATC-TTTTGAAGAACTGATTAATTGGACGAGAATAAAGATAGAGTCCCATTCTACATGTCAATACTGGCAACAATGAAATTTATAGTAAAAGGAAAATCCGTCGACTTTAGAAATCGTGAGGGTTCAAGTCCCTCTATCCCCAAAAAACCCATATGGACTCCCTAATTATTTATCCTCTCCTT-TTATCC-TTTTTTGTTAGCGGTTAAAAATTCG------TTATATTTCTCATTCACCCTACTCTTTTACAAAGAGATCTGAGCGGAAATGTTTTTCTCTTATC----ACAAGTCTTGTGATCTAAG----ATAA-TACGTGTACAAATGAACATCTTTGAGTAAGGAATCCCCATTTAAATGATTCATGGTCAA------TGTCATTATTCATACTGAAACTTACAAAGTCTTCCTT-TTGAAGATCCAAGAAATTCCAGGACCTGGATAATACTTTGTAAGACCCTTTCAATTGACA-TAGACCCGAGTTATCTAGC--AAAATGAGGATGCAGCGGTATTTATGTTAATGCACTTCCCAATGATACGTAAACAAGGCATTTCTGGTCCTTTA--------TAGAAAAGATCTATCATAGATATTTGTAATCTATCATTTATCGCTTGGGGGAGGAACAATAGTATTTCATTGCTACAAGTATGGATTATTGAGAATAATAAGACATGTATTTGGATATTTCCCTTGAACTCCGCAATCTTTTTGATTTGACATGGATAGTTGAAGGGAATTTTCCGAAGAAAAAATGGATTAGATTATGGGAGTGTGTGACTTGAACTATTGATTGGTCTGTGCAGATATATGCCTTTCGCTATCTGCCACATTGGAATTCACAACCAAATGTGTCTTTGTTCCAACCATTGCGTAAGCCC----------CATACAGAGGATAGGCTGGTTCACTTGAAGAGAATCTTTTCTATGATCAGATCCGAATCATGTCGTACATGAGCAGGTTCCGTAAGATCCAGTAG-----AATAAGTG-AACTAGATAACCCATAATCGAGATTATGGGTTATCTAG-TTCACTTACATACGATTGAATAGTATGGAAATGCATTCATTTCCTATGCATTGACACGATCTATGATACTATCGGAGTGAAACGGGGGATCTAAGGAAGAACAGAGGCTAGGCTATATTAGTAACAAGTAAACCC-----TTTGTGTGTCTGTCAAAAGTCTCCAAGTATTTTGGGGATAAACACCGATCCTAAGGTCTGAGACGACCCAGAAAGCATT--TGATCCTATCATGATCCACCTTGTAAGCCTACTTGGGTATTGAGTATTTACTTGTAAGAACCAAAATTTTTGCGCTGGATAGTTGCAACTCCGGAAAAAGAAATCC------------------AGTCAAATTTTTC------TTTTAAT--------AAAATCATTCATATTATCATA-----TATGTGTGGATCTAGATAACATATAGATTTTATATGGATTCCTTATGGTTCTTTTTCTTTTTGCTCGAGCCGTATGATGAAAAATTATCATGTCCGGTTCCTTCGGGGGATGGAT-----CTATAAAAATTCACCTATCCCAATAACAAAAAAACCTGACCTGAATGATCCTGTATTAAGAGCTAAATTGGCTAAAGGTATGGGTCATAATTATTACGGAGAGCCCGCATGGCCCAATGATCTTTTATATATTTTTCCAGTAGAGATTG-----------------------AAG-----AAACCAATGCAATATTTTTTAGGAAAGATAAAACTGGATGAATTCAAATTCGTTCAAATGGG-----------------------ATTGGAAGGTTCCTTATTTTCATTTAGGGTGTTCGTTTTATTTCTTCCCTTAGGACTTTGGTGTAGTTTATGCTCTCTCTCCTGGAATCG-AATTGTTGT-AACTGGACGCTTCTATCCT-CTAGCTAGTA--GGGATAGAAC-----AAAAAAAATATTTTCA------TTTTTTAATGAATTTTTTCTCATTTATCCGATTTATCAAATTTGAAACAAAAAGATACATTTTTTCAATGAACACA----------AAAAAATCCTAAAGTT---------ATACTATACAAAGGGTTGTCAAAATGGAATCAATTAGTTTCACCAATTCCTTC-----------------------ATTTTTACTAATGATCTTATA----TATGCCC-------TTCTATAGATATCTATAGAG----------------ATAGAGAAACCC----------------------------ATTTCTCT-------TATTATA-----GATAAATAGGTTGATGGGGAAAATAAGACCCCGC-------CCTCGAAATGATAAAATCTACTAAAAAGAAAGGTAAAACTTTGTATCTTGTCTTTA--------------------TTTTCAAAAAAA--------TTATTTTTTTAATTTAGAAATTTAGTAAACAGAAGCATTTTTATTCTACTTCCATTCCCTATTGTTTTCGGCC-------AATGAATAGGGAATGGGAATTATTCATTTTATTTTTAGATTAACAATGAAATCAGAC---------AGTCAAATCAATTGGGATTATTCTAACGTTTTATGA--------CTTATTTGTTTGTCGTACAAAAAAACTTTTTACATTTCCGGTAGGTCGTACAAAAAAAC-TTTTTGAATTCCCGGT-ACAAAGGCCATCTCCCCCAATTGAACAAGAGAATAATGACTATGTTACATTACACATGAAGTAA-----GAAAAAAGTC--TTG-CTTTCTCTTTCTTTATGATATAG-------ATATGTACAACTTTGACCAGCAATTTCATTTAGATC-TAAGTAAGGGCTCGAAAGAT-CCAATAGACAAAT-------------ATAAAGAAAAATAAAGAAGACCC-----CTTTGATTTTGTTCCCTTTATTCCCACGGCCTGGCCTGGTCAATACCTAGCCGGGCC----TTTTTTTGTTCCAACAAATCCTAGCTAAAAGAATTTAGCTG-------------CTTTGAACACA---------------AAAATGCTTGCTATTAAAGCAGC-------------------AATAAAAAGATGAGGGGTTATTTCCATTCTTACTTATTATTCCAT------------------TCTTACTTATTATATA--TTATA---------------------------TATATATATAAATTATATAAAATCAAAGTATCCTTTCTTATT-----ATTCCTTCTTCCCTTTTGAGTTACTTGACGACCTTACGGGAAT--ATAAAATGAAACTG-----TGGGTTCTTA---AATAATAATGAATGCATTTTTCTGTTATGATTTCAGTGGTTTTAGTGAGCCATATCTATCAAAATCCCCCCAGCAAAAGAAAAAATAGAACTT--GTTATTTC----------------ATTTAGTTATTTAAAAGAGCCC-----TCCTTTCCGGAATCTCATTAAATCGAAATCCCCCGCGAA-AAACGT-CGACACTCTCATTTTCATGA-----TTATGATC-CTA--T-CTTTATTACGCCTAATTTCCTCGGTTCGACAAAAAGTTCATTTGTATATAATAAGTATTAG-TATATCTAATACCTTACCCAGCCCATTTGGAAATTTTGGTTCAAACTCTTCGCTACTGGGTAAAAGATGCCCCCTCTTTGCATTTATTACGATTCTTTCTTCACGACTATCGGCATTGGACTAGTCTTTTTATTCCAAAGAAAGCCAGTTCTTTTTTTTCAAAACGAAAG------------------CAAAGATTATTCTTCTTCCTATATAATTCTCATGTATGTGAATACGAATCCATCTTCGTCTTTCTCCGTAACCAATCTTCTCATTTACAATCAACATCTTCTGGAGCCCTTCTTGAACGAATATATTTCTATGAAAAAATAGAACATCTTGTCGAAGTCTTTGCTAAA-GATTTTCAAGGCAATCTATGGTTGTTCAAGGATCCTTTCATGCATTATGTTAGGTATCAAGGAAAGTCAATTCTCGCTTCAAAGGAGAACTTTCTTTTGATGCGTAAATGGAAATATTACTTTGTACGTTTCTGGCAATGTCATTTTTACCAGTGGTTTCAACCAGGAAGGATCTATATAAACCAATTATCCAAACATTCCCTCGACCTTCTGGGCTATCTTTCAAGTGTGCGGCTAAATCCTTTAACGATACGCAGTCAAATGCTAGAAAATTCATTTCTAATTGATAATGCTGTTAAGAAGTTCGATACCATTGTTCCAATTATTCCTCTGATTGGATCATTGGTTAAAGCGAAATTTTGTAACGTATTAGGGCATCCTGTTAGTAAGGTAGTTTGGGTAGATTTATCAGATTTAAGACAT-AAAATTTACCCTACTTCTTTATCTTTC-TAGGAAGGGCCTTTCATGAGTTGAATAGAGATTTTCA-TTTTTTATTCATCATTCGGGTTGATGAACTAAA-CCAGATAGTTATATGAGTGAAAGAAACAGCTTATAAATTTGCAGTAAAAAGATTGAGTCTCATTTT-CTATGTAC-AAGAG-TTAAGTGAAAGTAACCATAAACATTA-GAAACGGTTTACCCCAAGATTGGTTAATTAGTGATCATGGCTTGAAGCGGGTGC-AAAAGATCAACTATATGGGG-TTTTTACTATCTATTACCATACATGTATTACCCTAACGGGCGATTAGCAAAAAGAGGTGGATAGTTAGGAACACCAAAGTACACAAAGGATTCGTAATAGAGATTATGTAAGTTATTCAACAGAATTTTTCTGTGCATAA-AAGGAATTCTGATTGGGACTTTAAGTTGGTAGAAATGATGAAGAAGTACTCCCCCTGATTCCGATCCAGAGTATACTCCTATCCACCGATTAAGTAAATAACTATCAAGAACGAAGTAATCCTTT-ACTTT------GTTTAAAGTCCCTTTTTCTGAGAAAGGAGAATAGGAACGAAAAAAATC-------AAATAG-AAAGA-ATAGAATTGCACTAG-----AAAGAAAGAGATCTTTTTTTATTCTTTCTTTCC-------------TCTATTTAGAAAGATAGAATTCTTGTCATCATTCGTGAACTAATGCGATGCCTAATTGTTTTTCGTAATCGAAAATGCTAGGTTG--------AAATATCTATGAATA-TTGCTACAAGAAAGA-TTTTATTGAAAA-TTGAAGTTATCACTCAAC-AAAGAAAAATAAAAA-TTATT-----AAAAGATAAGATCAA-TTCCG-AAGCGCTTTA-TTTTCAATATAGCAGACAGAATTCCATTGTCTAATTCGGG--ACTTTACAGTAGATTTTGATTCTATCTATCCTACGAATATAT-------------------CAAGATAAATAATAGCGAACTGGTCCTTAGA-TTTATTTGTGA-CCTTTGAGGAGCCAGTATGAGATGAAAATCTCATGTACGGTATTTAGATTA---------------------------------------------------------------------------------------------------------------------------------------------------------------------------------CTAAA------AATATTTAGTATTTTTCTTT-AA-----ATTTATTAAAA--ATATGTAAGTCAA--------------------------------------------------------------------------------TGTGAAAT-AAAAAAGGAGCAATAATCCCCTTCTTGTTCTATCAAG-AGGGCGATATTGCTCCTTTTTT-ATTTCTTTCAAATACTCGTATACACTAAGGCCGGGTCTTATCCATTTATAGATGGAGCTTCAAGAGCAGCTAGGTCTAGAGGGAAGTTATGAGCATTACGTTCATGCATAACTTCCATACCAAGGTTAGCGCGGTTAATGATATCAGCCCAAGTATTAATTACACGACCTTGACTATCAACTACAGATTGGTTGAAATTAAAACCATTTAGGTTGAAAGCCATAGTGCTGATACCTAAAGCAGTGAACCAGATACCTACTACAGGCCAAGCAGCTAGGAAGAAATGTAAAGAACGAGAGTTGTTGAAACTAGCATATTGGAAGATCAATCGGCCAAAATAACCATGAGCAGCTACAATATTATAAGTTTCTTCTTCTTGA-CCGAATC---TGTAACCTTCATTAGCAATAAATGCAAGAATATTTAC-TTCCATAATCTCATCGTTTTTTTACTTCAAAATAACT-CGGGATTTAATCCCATAGAGATAATAAATCTTTCGCCTGTCAATTCA----ATGAATTACCTCTCGATGATC-TTGAAATCGGATCAATATCATGAATAACAATATCTGAGCTATCAAATCAATTCGTCGTCGAGAATTGAATAGTATAACATAGAAAGATCTTT-TATCCATACCG------AATCCAA------------------------AATTTCTTTATTTATCAATCATTCTTTTCTGTTCTTTCTTTATTTACAACCTATCTTAGGTCCTCCTTGTACAATCATCGGATAAAGTATCGTCTGACCGCCCGTCCGTTTCCATTAGTCACAAACGCCCAACAAACAATAGAAGCGAAGTGGAAAAAGAAATAAGTTACGTTCTAAACTCCG----TTTTTTTAATGATCTAGTTTTCTTGGAAGACAAAGAAGTGTGATAAAGAGGAGTTCCGGGATAAAGGATGGAATATTCCATCAAACTAACTATTTGAGTTTGGGTTTTGTTCGTTYTTCGACGGG--CCCT-------AAAAAAAAAATAG--AAAAAAATAGGAAGGAAAAAT-GATTTATTCCCC-GCTACTTGTTAAGCTAAAAAAGGGGTGGGATCTTTGATTGATCTTTATTTTTCTTTTA-CCCCCCCTTCCTT---------------------------------------------------------------------------------------------------------------------------------------------------------------------------------------------------------------------------------------------------------------------------------------------------------------AACGCTCTCAATAATTGTACTATTCTACATATGTCTTTCTCCTACCAATCAGTATTATTTGAAATAATGAAAATTCCCCTATTTGTTTGATGAGAAGTGC----GAAATGCCAAAGGAAA--GAAAAAAGAACCCCCTT-GGAAATG-AAATTCTGCTCCCCGTGCCCCCTTTAACAGAAAAGGGAAGATTACAAATTGACTTATTATACTCCTGACTATGAAACCAAAGATACTGATATCTTGGCAGCATT-CCGAGTAACTCCTCAACCTGGAGTTCCACCTGAAGAAGCAGGGGCCGCGGTAGCTGCCGAATCTTCAACTGGTACATGGACAACTGTGTGGACCGATGGACTTACCAGCCTTGATCGTTACAAAGGGCGATGCTACCACATCGAGCCCGTTGCTGGAGAAGAAAATCAATATATTGCTTATGTAGCTTACCCATTAGACCTTTTTGAAGAAGGTTCTGTTACAAACATGTTTACTTCTATTGTGGGTAATGTATTTGGGTTCAAAGCCCTGCGCGCTCTACGTCTGGAAGATCTGCGAATCCCTGTCGCTTATACTAAAACTTTCCAAGGCCCGCCTCATGGCATCCAAGTTGAGAGAGATAAATTGAACAAGTATGGTCGCCCCCTGTTGGGATGTACTATTAAACCTAAATTGGGGTTATCTGCTAAAAACTATGGTAGAGCGGTTTATGAATGTCTACGTGGTGGACTTGATTTTACCAAAGATGATGAGAA--CGT--GAACTCCCAACCATTTATGCGTT---GGAGAGATCGATTC

Abelia_chinensis_BOP012224 CCTGAGCCAAATCCAGTTTTACGA----AAACAAGGGTTCAGAAAGCTAAAATC-AAAAAGGATAGGTGCAGAGACTCAATGGAAGCTGTTCTAACAAATGGAGTTGACTGTGTTGTGTTGGTAGAAAGAATCCTTCCATAGAAACTTCAGAAAGGATAAACGTATAAACATGGATATACGCATTGAAATACTAT-ATACTCTACCAAATGATTAATGACGATCCGAATCTGTATT--TA-----TATATATCAAAATGGGAGAATGGTTGTGAAGTGATTCCATATTGAAGAAAGAATCGAATATTTATTGATCAAATCATTCACTCCATAGTCTGATAGATC-TTTTGAAGAACTGATTAATTGGACGAGAATAAAGATAGAGTCCCATTCTACATGTCAATACTGGCAACAATGAAATTTATAGTAAAAGGAAAATCCGTCGACTTTAGAAATCGTGAGGGTTCAAGTCCCTCTATCCCCAAAAAACCCATATGGACTCCCTAATTATTTATCCTCTCCTT-TTATCC-TTTTTTGTTAGCGGTTAAAAATTCG------TTATCTTTCTCATTCACCCTACTCTTTTACAAAGAGATCTGAGCGGAAATGTTTTTCTCTTATC----ACAAGTCTTGTGATCTAAG----ATAA-TACGTGTACAAATGAACATCTTTGAGTAAGGAATCCCCATTTAAATGATTCATGGTCAA------TGTCATTATTCATACTGAAACTTACAAAGTCTTCCTT-TTGAAGATCCAAGAAATTCCAGGACCTGGATAAGACTTTGTAAGACCCTTTCAATTGACA-TAGACCCGAGTTATCTAGC--AAAATGAGGATGCAGCGGTATTTATGTTAATGCACTTCCCAATGATACGTAAACAAGGCATTTCTGGTCCTTTA--------TAGAAAAGATCTATCATAGATATTTGTAATCTATCATTTATCGCTTGGGGGAGGAACAATAGTATTTCATTGCTACAAGTATGGATTATTGAGAATAATAAGACATGTATTTGGATATTTCCCTTGAACTCCGCAATCTTTTTGATTTGACATGGATAGTTGAAGGGAATTTTCCGAAGAAAAAATGGATTAGATTATGGGAGTGTGTGACTTGAACTATTGATTGGTCTGTGCAGATATATGCCTTTCGCTATCTGCCACATTGGAATTCACAACCAAATGTGTCTTTGTTCCAACCATTGCGTAAGCCC----------CATACAGAGGATAGGCTGGTTCACTTGAAGAGAATCTTTTCTATGATCAGATCCGAATCATGTCGTACATGAGCAGGTTCCGTAAGATCCAGTAG-----AATAAGTG-AACTAGATAACCCATAATCTCGATTATGGGTTATCTAG-TTCACTTACATACGATTGAATAGTATGGAAATGCATTCATTTCCTATGCATTGACACGATCTATGATACTATCGGAGTGAAACGGGGGATCTAAGGAAGAACAGAGGCTAGGCTATATTAGTAACAAGTAAACCC-----TTTGTGTGTCTGTCAAAAGTCTCCAAGTATTTTGGGGATAAACACCGATCCTAAGGTCTGAGACGACCCAGAAAGCATT--TGATCCTATCATGATCCACCTTGTAAGCCTACTTGGGTATTGAGTATTTACTTGTAAGAACCAAAATTTTTGCGCTGGATAGTTGCAACTCCGGAAAAAGAAATCC------------------AGTCAAATTTTTC------TTTTAAT--------AAAATCATTCATATTATCATA-----TATGTGTGGATCTAGATAACATATAGATTTTATATGGATTCCTTATGGTTCTTTTTCTTTTTGCTCGAGCCGTATGATGAAAAATTATCATGTCCGGTTCCTTCGGGGGATGGAT-----CTATAAAAATTCACCTATCCCAATAACAAAAAAACCTGACCTGAATGATCCTGTATTAAGAGCTAAATTGGCTAAAGGTATGGGTCATAATTATTACGGAGAGCCCGCATGGCCCAATGATCTTTTATATATTTTTCCAGTAGAGATTG-----------------------AAG-----AAACCAATGCAATATTTTTTAGGAAAGATAAAACTGGATGAATTCAAATTCGTTCAAATGGG-----------------------ATTGGAAGGTTCCTTATTTTCATTTAGGGTGTTCGTTTTATTTCTTCCCTTAGGACTTTGGTGTAGTTTATGCTCTCTCTCCTGGAATCG-AATTGTTGT-AACTGGACGCTTCTATCCT-CTAGCTAGTA--GGGATAGAAC-----AAAAAAAATATTTTCA------TTTTTTAATGAATTTTTTCTCATTTATCCGATTTATCAAATTTGAAACAAAAAGATACATTTTTTCAATGAACACA----------AAAAAATCCTAAAGTTATACTATACATACTATACAAAGGGTTGTCAAAATGGAATCAATTAGTTTCACCAATTCCTTC-----------------------ATTTTTACTAATGATCTTATA----TATGCCC-------TTCTATAGATATATATAGAG----------------ATAGAGAAACCC----------------------------ATTTTTCT-------TATTATA-----GATAAATAGGTTGATGGGGAAAATAAGACCCCGC-------CCTCGAAATGATAAAATCTACTAAAAAGAAAGGTAAAACTTTGTATCTTGTCTTTA--------------------TTTTCAAAAAAATTATTA--TTTTTTTTTTAATTTAGAAATTTAGTAAACAGAAGCATTTTTATTCTACTTCCATTCCCTATTGTTTTCGGCC-------AATGAATAGGGAATGGGAATTATTCATTTTATTTTTAGATTAACAATGAAATCAGAC---------AGTCAAATCAATTGGGATTATTCTAACGTTTTATGA--------CTTATTTGTTTGTCGTACAAAAAAACTTTTTACATTTCCGGTAGGTCGTACAAAAAAAC-TTTTTGAATTCCCGGT-ACAAAGGCCATCTCCCCCAATTGAACAAGAGAATAATGACTATGTTACATTACACATGAAGTAA-----GAAAAAAGTC--TTG-CTTTCTCTTTCTTTATGATATAG-------ATATGTACAACTTTGACCAGAAATTTCATTTAGATC-TAAGTAAGGGCTCGAAAGAT-CCAATAGACAAAT-------------ATAAAGAAAAATAAAGAAGACCC-----CTTTGATTTTGTTCCCTTTATTCCCACGGCCTGGCCTGGTCAATACCTAGCCGGGCC----TTTTTTTGTTCCAACAAATCCTAGCTAAAAGAATTTAGCTG-------------CTTTGAACACA---------------AAAATGCTTGCTATTAAAGCAGC-------------------AATAAAAAGATGAGGGGTTATTTCCATTCTTACTTATTATTCCAT------------------TCTTACTTATTATATACTTATTATA-------------------------TATATATATAAATTATATAAAATCAAAGTATCCTTTCTTATT-----ATTCCTTCTTCCCTTTTGAGTTACTTGACGACCTTACGGGAAT--ATAAAATGAAACTG-----TGGGTTCTTA---AATAATAATGAATGCATTTTTCTGTTATGATTTCAGTGGTTTTAGTGAGCCATATCTATCAAAATCCCCCCAGCAAAAGAAAAAATAGAACTT--GTTATTTC----------------ATTTAGTTATTTAAAAGAGCCC-----TCCTTTCCGGAATCTCATTAAATCGAAATCCCCCGCGAA-AAACGT-CGACACTCTCATTTTCATGA-----TTATGATC-CTA--T-CTTTATTACGCCTAATTCCTCGGTT-CGACAAAAAGTTCATTTGTATATAATAAGTATTAG-TATATCTAATACCTTACCCAGCCCATTTGGAAATTTTGGTTCAAACTCTTCGCTACTGGGTAAAAGATGCCCCCTCTTTGCATTTATTACGATTCTTTCTTCACGACTATCGGCATTGGACTAGTCTTTTTATTCCAAAGAAAGCCAGTTCTTTTTTTTCAAAACGAAAG------------------CAAAGATTATTCTTCTTCCTATATAATTCTCATGTATGTGAATACGAATCCATCTTCGTCTTTCTCCGTAACCAATCTTCTCATTTACAATCAACATCTTCTGGAGCCCTTCTTGAACGAATATATTTCTATGAAAAAATAGAACATCTTGTCGAAGTCTTTGCTAAA-GATTTTCAAGGCAATCTATGGTTGTTCAAGGATCCTTTCATGCATTATGTTAGGTATCAAGGAAAGTCAATTCTCGCTTCAAAGGAGAACTTTCTTTTGATGCGTAAATGGAAATATTACTTTGTACGTTTCTGGCAATGTCATTTTTACCAGTGGTTTCAACCAGGAAGGATCTATATAAACCAATTATCCAAACATTCCCTCGACCTTCTGGGCTATCTTTCAAGTGTGCGGCTAAATCCTTTAACGATACGCAGTCAAATGCTAGAAAATTCATTTCTAATTGATAATGCTGTTAAGAAGTTCGATACCATTGTTCCAATTATTCCTCTGATTGGATCATTGGTTAAAGCGAAATTTTGTAACGTATTAGGGCATCCTGTTAGTAAGGTAGTTTGGGTAGATTTATCAGATTTAAGACAT-AAAATTTACCCTACTTCTTTATCTTTC-TAGGAAGGGCCTTTCATGAGTTGAATAGAGATTTTCA-TTTTTTATTCATCATTCGGGTTGATGAACTAAA-CCAGATAGTTATATGAGTGAAAGAAACAGCTTATAAATTTGCAGTAAAAAGATTGAGTCTCATTTT-CTATGTAC-AAGAG-TTAAGTGAAAGTAACCATAAACATTA-GAAACGGTTTACCCCAAGATTGGTTAATTAGTGATCATGGCTTGAAGCGGGTGC-AAAAGATCAACTATATGGGG-TTTTTACTATCTATTACCATACATGTATTACCCTAACGGGCGATTAGCAAAAAGAGGTGGATAGTTAGGAACACCAAAGTACACAAAGGATTCGTAATAGAGATTATGTAAGTTATTCAACAGAATTTTTCTGTGCATAA-AAGGAATTCTGATTGGGACTTTAAGTTGGTAGAAATGATGAAGAAGTACTCCCCCTGATTCCGATCCAGAGTATACTCCTATCCACCGATTAAGTAAATAACTATCAAGAACGAAGTAATCCTTT-ACTTT------GTTTAAAGTCCCTTTTTCTGAGAAAGGAGAATAGGAACGAAAAAAATC-------AAATAG-AAAGA-ATAGAATTGCACTAG-----AAAGAAAGAGATCTTTTTTTATTCTTTCTTTCC-------------TCTATTTAGAAAGATAGAATTCTTGTCATCATTCGTGAACTAATGCGATGCCTAATTGTTTTTCGTAATCGAAAATGCTAGGTTG--------AAATATCTATGAATA-TTGCTACAAGAAAGA-TTTTATTGAAAA-TTGAAGTTATCACTCAAC-AAAGAAAAATAAAAA-TTATT-----AAAAGATAAGATCAA-TTCCG-AAGCGCTTTA-TTTTCAATATAGCAGACAGAATTCCATTGTCTAATTCGGG--ACTTTACAGTAGATTTTGATTCTATCTATCCTACGAATATAT-------------------CAAGATAAATAATAGCGAACTGGTCCTTAGA-TTTATTTGTGA-CCTTTGAGGAGCCAGTATGAGATGAAAATCTCATGTACGGTATTTAGATTA---------------------------------------------------------------------------------------------------------------------------------------------------------------------------------CTAAA------AATATTTAGTATTTTTCTTT-AA-----ATTTATTAAAA--ATATGTAAGTCAA--------------------------------------------------------------------------------TGTGAAAT-AAAAAAGGAGCAATAATCCCCTTCTTGTTCTATCAAG-AGGGCGATATTGCTCCTTTTTT-ATTTCTTTCAAATACTCGTATACACTAAGGCCGGGTCTTATCCATTTATAGATGGAGCTTCAAGAGCAGCTAGGTCTAGAGGGAAGTTATGAGCATTACGTTCATGCATAACTTCCATACCAAGGTTAGCGCGGTTAATGATATCAGCCCAAGTATTAATTACACGACCTTGACTATCAACTACAGATTGGTTGAAATTAAAACCATTTAGGTTGAAAGCCATAGTGCTGATACCTAAAGCAGTGAACCAGATACCTACTACAGGCCAAGCAGCTAGGAAGAAATGTAAAGAACGAGAGTTGTTGAAACTAGCATATTGGAAGATCAATCGGCCAAAATAACCATGAGCAGCTACAATATTATAAGTTTCTTCTTCTTGA-CCGAATC---TGTAACCTTCATTAGCAATAAATGCAAGAATATTTAC-TTCCATAATCTCATCGTTTTTTTACTTCAAAATAACT-CGGGATTTAATCCCATAGAGATAATAAATCTTTCGCCTGTCAATTCA----ATGAATTACCTCTCGATGATC-TTGAAATCGGATCAATATCATGAATAACAATATCTGAGCTATCAAATCAATTCGTCGTCGAGAATTGAATAGTATAACATAGAAAGATCTTT-TATCCATACCG------AATCCAA------------------------AATTTCTTTATTTATCAATCATTCTTTTCTGTTCTTTCTTTATTTACAACCTATCTTAGGTCCTCCTTGTACAATCATCGGATAAAGTATCGTCTGACCGCCCGTCCGTTTCCATTAGTCACAAACGCCCAACAAACAATAGAAGCGAAGTGGAAAAAGAAATAAGTTACGTTCTAAACTCCG----TTTTTTTAATGATCTAGTTTTCTTGGAAGACAAAGAAGTGTGATAAAGAGGAGTTCCGGGATAAAGGATGGAATATTCCATCAAACTAACTATTTGAGTTTGGGTTTTGTTCGTTYTTCGACGGG--CCCT-------AAAAAAAAAATAG--AAAAAAATAGGAAGGAAAAAT-GATTTATTCCCC-GCTACTTGTTAAGCTAAAAAAGGGGTGGGATCTTTGATTGATCTTTATTTTTCTTTTA-CCCCCCCTTCCTT---------------------------------------------------------------------------------------------------------------------------------------------------------------------------------------------------------------------------------------------------------------------------------------------------------------AACGCTCTCAATAATTGTACTATTCTACATATGTCTTTCTCCTACCAATCAGTATTATTTGAAATAATGAAAATTCCCCTATTTGTTTGATGAGAAGTGC----GAAATGCCAAAGGAAA--GAAAAAAGAACCCCCTT-GGAAATG-AAATTCTGCTCCCCGTGCCCCCTTTAACAGAAAAGGGAAGATTACAAATTGACTTATTATACTCCTGACTATGAAACCAAAGATACTGATATCTTAGCAGCATT-CCGAGTAACTCCTCAACCTGGAGTTCCACCTGAAGAAGCAGGGGCCGCGGTAGCTGCCGAATCTTCAACTGGTACATGGACAACTGTGTGGACCGATGGACTTACCAGCCTTGATCGTTACAAAGGGCGATGCTACCACATCGAGCCCGTTGCTGGAGAAGAAAATCAATATATTGCTTATGTAGCTTACCCATTAGACCTTTTTGAAGAAGGTTCTGTTACAAACATGTTTACTTCTATTGTGGGTAATGTATTTGGGTTCAAAGCCCTGCGCGCTCTACGTCTGGAAGATCTGCGAATCCCTGTCGCTTATACTAAAACTTTCCAAGGCCCGCCTCATGGCATCCAAGTTGAGAGAGATAAATTGAACAAGTATGGTCGCCCCCTGTTGGGATGTACTATTAAACCTAAATTGGGGTTATCTGCTAAAAACTATGGTAGAGCGGTTTATGAATGTCTACGTGGTGGACTTGATTTTACCAAAGATGATGAGAA--CGT--GAACTCCCAACCATTTATGCGTT---GGAGAGATCGATTC

Abelia_engleriana_BOP012231 CCTGAGCCAAATCCAGTTTTACGA----AAACAAGGGTTCAGAAAGCTAAAATC-AAAAAGGATAGGTGCAGAGACTCAATGGAAGCTGTTCTAACAAATGGAGTTGACTGTGTTGTGTTGGTAGAAAGAATCCTTCCATAGAAACTTCAGAAAGGATAAACGTATAAACATGGATATACGCATTGAAATACTAT-ATACTCTACCAAATGATTAATGACGACCCGAATCTGTATT--TA-----TATATATCAAAATGGGAGAATGGTTGTGAAGTGATTCCATATTGAAGAAAGAATCGAATATTTATTGATCAAATCATTCACTCCATAGTCTGATAGATC-TTTTGAAGAACTGATTAATTGGACGAGAATAAAGATAGAGTCCCATTCTACATGTCAATACCGGCAACAATGAAATTTATAGTAAAAGGAAAATCCGTCGACTTTAGAAATCGTGAGGGTTCAAGTCCCTCTATCCCCAAAAAACCCATATGGACTCCCTAATTATTTATCCTCTCCTT-TTATCC-TTTTTTGTTAGCGGTTAAAAATTCG------TTATCTTTCTCATTCACCCTACTCTTTTACAAAGAGATCTGAGCGGAAATGTTTTTCTCTTATC----ACAAGTCTTGTGATCTAAG----ATAA-TACGTGTACAAATGAACATCTTTGAGTAAGGAATCCCCATTTAAATGATTCATGGTCAA------TGTCATTATTCATACTGAAACTTACAAAGTCTTCCTT-TTGAAGATCCAAGAAATTCCAGGACCTGGATAAGACTTTGTAAGACCCTTTCAATTGACA-TAGACCCGAGTTATCTAGC--AAAATGAGGATGCAGCGGTATTTATGTTAATGCACTTCCCAATGATACGTAAACAAGGCATTTCTGGTCCTTTA--------TAGAGAAGATCTATCATAGATATTTGTAATCTATCATTTATCGCTTGGGGGAGGAACAATAGTATTTCATTGCTACAAGTATGGATTATTGAGAATAATAAGACATGTATTTGGATATTTCCCTTGAACTCCGCAATCTTTTTGATTTGACATGGATAGTTGAAGGGAATTTTCCGAAGAAAAAATGGATTAGATTATGGGAGTGTGTGACTTGAACTATTGATTGGTCTGTGCAGATATATGCCTTTCGCTATCTGCCACATTGGAATTCACAACCAAATGTGTCTTTGTTCCAACCATTGCGTAAGCCC----------CATACAGAGGATAGGCTGGTTCACTTGAAGAGAATCTTTTCTATGATCAGATCCGAATCATGTCGTACATGAGCAGGTTCCGTAAGATCCAGTAG-----AATAAGTG-AACTAGATAACCCATAATCTCGATTATGGGTTATCTAG-TTCACTTACATACGATTGAATAGTATGGAAATGCATTCATTTCCTATGCATTGACACGATCTATGATACTATCGGAGTGAAACAGGGGATCTAAGGAAGAACAGAGGCTAGGCTATATTAGTAACAAGTAAACCC-----TTTGTGTGTCTGTCAAAAGTCTCCAAGTATTTTGGGGATAAACACCGATCCTAAGGTCTGAGACGACCCAGAAAGCATT--TGATCCTATCATGATCCACCTTGTAAGCCTACTTGGGTATTGAGTATTTACTTGTAAGAACCAAAATTTTTGCGCTGGATAGTTGCAACTCCGGAAAAAGAAATCC------------------AGTCAAATTTTTC------TTTTAAT--------AAAATCATTCATATTATCATA-----TATGTGTGGATCTAGATAACATATAGATTTTATATGGATTCCTTATGGTTCTTTTTCTTTTTGCTCGAGCCGTATGATAAAAAATTATCATGTCCGGTTCCTTCGGGGGATGGAT-----CTATAAAAATTCACCTATCCCAATAACAAAAAAACCTGACCTGAATGATCCTGTATTAAGAGCTAAATTGGCTAAAGGTATGGGTCATAATTATTACGGAGAGCCCGCATGGCC----------------------------GAGATTG-----------------------AAG-----AAATCAATGCAATATTTTTTAGGAAAGATAAAACTGGATGAATTCAAATTCGTTCAAATGGG-----------------------ATTGGAAGGTTCCTTATTTTCATTTAGGGTGTTCGTTTTATTTCTTCCCTTAGGACTTTGGTGTAGTTTATGCTCTCTCTTCTGGAATCG-AATTGTTGT-AACTGGACGCTTCTATCCT-CTAGCTAGTA--GGGATAGAAC-----AAAAAAAATATTTTCA------TTTTTTAATGAATTTTTTCTCATTTATCCGATTTATCAAATTTGAAACAAAAAGATACATTTTTTCAATGAACACA----------AAAAAATCCTAAAGTT---------ATACTATACAAAGGGTTGTCAAAATGGAATCAATTAGTTTCACCAATTCCTTC-----------------------ATTTTTACTAATGATCTTATA----TATGCCC-------TTCTATAGATATATATAGAG----------------ATAGAGAAACCC----------------------------ATTTTTCT-------TATTATA-----GATAAATAGGTTGATGGGGAAAATAAGACCCCGC-------CCTCGAAATGATAAAATCTACTAAAAAGAAAGGTAAAACCTTGTATCTTGTCTTTA--------------------TTTTCAAAAAAA--------TTCTTTTTTTTATTTAGAAATTTAGTAAACAGAAGCATTTTTATTCTACTTCCATTCCCTATTGTTTTCGGCC-------AATGAATAGGGAATGGGAATTATTGATTTTATTTTTAGATTAACAATGAAATCAGAC---------AGTCAAATCAATTGGGATTATTCTAACGTTTTATGA--------CTTATTTGTTTGTCGTACAAAAAAACTTTTTACATTTCCGGTAGGTCGTACAAAAAGAC-TTTTTGAATTCCCGGT-ACAAAGGCCATCTCCCCCAATTGAACAAGAGAATAATGACTATGTTACATTACACATGAAGTAA-----GAAAAAAGTC--TTG-CTTTCTCTTTCTTTATGATATAG-------ATATGTACAACTTTGACCAGCAATTTCATTTAGATC-TAAGTAAGGGCTCGAAAGAT-CCAATAGACAAAT-------------ATAAAGAAAAATAAAGAAGACCC-----CTTTAATTTTGTTCCCTTTATTCCCACGGCCTGGCCTGGTCAATACCTAGCCGGGCC----TTTTTTTGTTCCAACAAATCCTAGCTAAAAGAATTTAGCTG-------------CTTTGAACACA---------------AAAATGCTTGCTATTAAAGCAGC-------------------AATAAAAAGATGAGGGGTTATTTCCATTCTTACTTAT--------------------------------------TATA--TATTATATTATTTATATTA-------------AATATATATAAATTATATAAAATCAAAGTATCCTTTCTTATT-----ATTCCTTCTTCCCTTTTGAGTTACTTGACGACCTTACGGGAAT--ATAAAATGAAACTG-----TGGGTTCTTA---AATAATAATGAATGCATTTTTCTGTTATGATTTCAGTGGTTTTAGTGAGCCATATCTATCAAAATCCCCCCAGCAAAAGAAAAAATAGAACTT--GTTATTTC----------------ATTTAGTTATTTAAAAGAGCCC-----TCCTTTCCGGAATCTCATTAAATCGAAATCCCCCGCGAA-AAACGT-CGACACTCTCATTTTCATGA-----TTATGATC-CTA--T-CTTTATTACGCCTAATTCCTCGGTT-CGACAAAAAGTTCATTTGTATATAATAAGTATTAG-TATATCTAATACCTTACCCAGCCCATTTGGAAATTTTGGTTCAAACTCTTCGCTACTGGGTAAAAGATGCCCCCTCTTTGCATTTATTACGATTCTTTCTTCACGACTATCGGCATTGGACTAGTCTTTTTATTCCAAAGAAAGCCAGTTCTTTTTTTTCAAAACGAAAG------------------CAAAGATTATTCTTCTTCCTATATAATTCTCATGTATGTGAATACGAATCCATCTTCGTCTTTCTCCGTAACCAATCTTCTCATTTACAATCAACATCTTCTGGAGCCCTTCTTGAACGAATATATTTCTATGAAAAAATAGAACATCTTGTCGAAGTCTTTGCTAAA-GATTTTCAAGGCAATCTATGGTTGTTCAAGGATCCTTTCATGCATTATGTTAGGTATCAAGGAAAGTCAATTCTCGCTTCAAAGGAGAACTTTCTTTTGATGCGTAAATGGAAATATTACTTTGTACGTTTCTGGCAATGTCATTTTTACCAGTGGTTTCAACCAGGAAGGATCTATATAAACCAATTATCCAAACATTCCCTCGACCTTCTGGGCTATCTTTCAAGTGTGCGGCTAAATCCTTTAACGATACGCAGTCAAATGCTAGAAAATTCATTTCTAATTGATAATGCTGTTAAGAAGTTCGATACCATTGTTCCAATTATTCCTCTGATTGGATCATTGGTTAAAGCGAAATTTTGTAACGTATTAGGGCATCCTGTTAGTAAGGTAGTTTGGGCAGATTTATCAGATTTAAGACAT-AAAATTTACCCTACTTCTTTATCTTTC-TAGGAAGGGCCTTTCATGAGTTGAATAGAGATTTTCA-TTTTTTATTCATCATTCGGGTTGATGAACTAAA-CCAGATAGTTATATGAGTGAAAGAAACAGCTTATAAATTTGCAGTAAAAAGATTGAGTCTCATTTT-CTATGTAC-AAGAG-TTAAGTGAAAGTAACCATAAACATTA-GAAACGGTTTACCCCAAGATTGGTCAATTAGTGATCATGGCTTGAAGCGGGTGC-AAAAGATCAACTATATGGGG--TTTTACTATCTATTACCATACATGTATTACCCTAACGGGCGATTAGCAAAAAGAGGTGGATAGTTAGGAACACCAAAGTACACAAAGGATTCGTAATAGAGATTATGTAAGTTATTCAACAGAATTTTTCTGTGCATAA-AAGGAATTCTGATTGGGACTTTAAGTTGGTAGAAATGATGAAGAAGTACTCCCCCTGATTCCGATCCAGAGTATACTCCTATCCACCGATTAAGTAAATAACTATCAAGAACGAAGTAATCCTTT-ACTTT------GTTTAAAGTCCCTTTTTCTGAGAAAGGAGAATAGGAACGAAAAAAATC-------AAATAG-AAAGA-ATAGAATTGCACTAGAAAGAAAAGAAAGAGATCTTTTTTTATTCTTTCTTTCC-------------TCTATTTAGAAAGATAGAATTCTTGTCATCATTCGTGAACTAATGCGATGCCTAATTGTTTTTCGTAATCGAAAATGCTAGGTTG--------AAATATCTATGAATA-TTGCTACAAGAAAGA-TTTTATTGAAAA-TTTAAGTTATCACTCAAC-AAAGAAAAATAAAAA-TTATT-----AAAAGATAAGATCAA-TTCCG-AAGCGCTTTA-TTTTCAATATAGCAGACAGAATTCCATTGTCTAATTCGGG--ACTTTACAGTAGATTTTGATTCTATCTATCCTACGAATATAT-------------------CAAGATAAATAATAGCGAACTGGTCCTTAGA-TTTATTTGTGA-CCTTTGAGGAGCC-GTATGAGATGAAAATCTCATGTACGGTATTTAGATTA---------------------------------------------------------------------------------------------------------------------------------------------------------------------------------CTAAA------AATATTTACGATTTTTCTTT-AA-----ATAAAAAAAAA--ATATGTAAGTCAA--------------------------------------------------------------------------------TGTGAAAT-AAAAAAGGAGCAATAATCCCCTTCTTGTTCTATCAAGAAGGGCGATATTGCTCCTTTTTT-ATTTCTTTCAAATACTCGTATACACTAAGGCCAGGTCTTATCCWTTTATAGATGGAGCTTCAAGAGCAGCTAGGTCTAGAGGGAAGTTATGAGCATTACGTTCATGCATAACTTCCATACCAAGGTTAGCGCGGTTAATGATATCAGCCCAAGTATTAATTACACGACCTTGACTATCAACTACAGATTGGTTGAAATTAAAACCATTTAGGTTGAAAGCCATAGTGCTGATACCTAAAGCAGTGAACCARATACCTACTACAGGCCAAGCAGCTAGGAARAAATGTAAAGAACGAGAGTTGTTGAAACTAGCATATTGRAARATCAATCGGCCAAAATAACCATGAGCAGCTACAATATTATAAGTTTCTTCTTCTTGA-CCGAATC---TGTAACCTTCATTAGCAATAAATGCAAGAATATTTACTTTCCATAATCTCATCGTTTTTTTACTTCAAAATAACT-CGGGATTTAATCCCATAGAGATAATAAATCTTTCGCCTGTCAATTCA----ATGAATTACCTCTCGATGATC-TTGAAATCGGATCAATATCATGAATAACAATATCTGAGCTATCAAATCAATTCGTCGTCGAGAATTGAATAGTATAACATAGAAAGATCTTT-TATCCATACCG------AATCCAA------------------------AATTTCTTTATTTATCAATCATTCTTTTCTGTTCTTTCTTTATTTACAACCTATCTTAGGTCCTCCTTGTACAATCATCGGATAAAGTATCGTCTGACCGCCCGTCCGTTTCCATTAGTCACAAACGCCCAACAAACAATAGAAGCGAAGTGGAAAAAGAAATAAGTTACGTTCTAAACTCCG----TTTTTTTAATGATCTAGTTTTCTTGGAAGACAAAGAAGTGTGATAAAGAGGAGTTCCGGGATAAAGGATGTAATATTCCATCAAACTAACTATTTGAGTTTGGGTTTTGTTCGTTYTTCGACGGG--CCCT------AAAAAAAAAAATAG-AAAAAAAATAGGAAGGAAAAAT-GATTTATTCCCCCGCTACTTGTTAAGCTAAAAAAGGGGTGGGATCTTTGATTGATCTTTATTTTTCTTTTA-CCCCCCCTTCCTT---------------------------------------------------------------------------------------------------------------------------------------------------------------------------------------------------------------------------------------------------------------------------------------------------------------AACGCTCTCAATAATTGTACTATTCTACATATGTCTTTCTCCTACCAATCAGTATTATTTGAAATAATGAAAATTCCCCTATTTGTTTGATGAGAAGTGC----GAAATGCCAAAGGAAA--GAAAAAAGAACCCCCTT-GGGAATGAAAATTCTGCTCCCCGTGCCCCCTTTAACGAGAAAAAGAAGATTACAAATTGACTTATTATACTCCTGACTATGAAACCAAAGATACTGATATCTTGGCAGCATT-CCGAGTAACTCCTCAACCTGGAGTTCCACCTGAAGAAGCAGGGGCCGCGGTAGCTGCCGAATCTTCAACTGGTACATGGACAACTGTGTGGACCGATGGACTTACCAGCCTTGATCGTTACAAAGGGCGATGCTACCACATCGAGCCCGTTGCTGGAGAAGAAAATCAATATATTGCTTATGTAGCTTACCCATTAGACCTTTTTGAAGAAGGTTCTGTTACTAACATGTTTACTTCTATTGTGGGTAATGTATTTGGGTTCAAAGCCCTGCGCGCTCTACGTCTGGAAGATCTGCGAATCCCTGTCGCTTATACTAAAACTTTCCAAGGCCCGCCTCATGGCATCCAAGTTGAGAGAGATAAATTGAACAAGTATGGTCGCCCCCTGTTGGGATGTACTATTAAACCTAAATTGGGGTTATCTGCTAAAAACTATGGTAGAGCGGTTTATGAATGTCTACGTGGTGGACTTGATTTTACCAAAGATGATGAGAA--CGT--GAACTCCCAACCATTTATGCGTT---GGAGAGATCGATTC

Abelia_engleriana_BOP012232 CCTGAGCCAAATCCAGTTTTACGA----AAACAAGGGTTCAGAAAGCTAAAATC-AAAAAGGATAGGTGCAGAGACTCAATGGAAGCTGTTCTAACAAATGGAGTTGACTGTGTTGTGTTGGTAGAAAGAATCCTTCCATAGAAACTTCAGAAAGGATAAACGTATAAACATGGATATACGCATTGAAATACTAT-ATACTCTACCAAATGATTAATGACGACCCGAATCTGTATT--TA-----TATATATCAAAATGGGAGAATGGTTGTGAAGTGATTCCATATTGAAGAAAGAATCGAATATTTATTGATCAAATCATTCACTCCATAGTCTGATAGATC-TTTTGAAGAACTGATTAATTGGACGAGAATAAAGATAGAGTCCCATTCTACATGTCAATACCGGCAACAATGAAATTTATAGTAAAAGGAAAATCCGTCGACTTTAGAAATCGTGAGGGTTCAAGTCCCTCTATCCCCAAAAAACCCATATGGACTCCCTAATTATTTATCCTCTCCTT-TTATCC-TTTTTTGTTAGCGGTTAAAAATTCG------TTATCTTTCTCATTCACCCTACTCTTTTACAAAGAGATCTGAGCGGAAATGTTTTTCTCTTATC----ACAAGTCTTGTGATCTAAG----ATAA-TACGTGTACAAATGAACATCTTTGAGTAAGGAATCCCCATTTAAATGATTCATGGTCAA------TGTCATTATTCATACTGAAACTTACAAAGTCTTCCTT-TTGAAGATCCAAGAAATTCCAGGACCTGGATAAGACTTTGTAAGACCCTTTCAATTGACA-TAGACCCGAGTTATCTAGC--AAAATGAGGATGCA-------------------------CAATGATACGTAAACAAGGCATTTCTGGTCCTTTA--------TAGAGAAGATCTATCATAGATATTTGTAATCTATCATTTATCGCTTGGGGGAGGAACAATAGTATTTCATTGCTACAAGTATGGATTATTGAGAATAATAAGACATGTATTTGGATATTTCCCTTGAACTCCGCAATCTTTTTGATTTGACATGGATAGTTGAAGGGAATTTTCCGAAGAAAAAATGGATTAGATTATGGGAGTGTGTGACTTGAACTATTGATTGGTCTGTGCAGATATATGCCTTTCGCTATCTGCCACATTGGAATTCACAACCAAATGTGTCTTTGTTCCAACCATTGCGTAAGCCC----------CATACAGAGGATAGGCTGGTTCACTTGAAGAGAATCTTTTCTATGATCAGATCCGAATCATGTCGTACATGAGCAGGTTCCGTAAGATCCAGTAG-----AATAAGTG-AACTAGATAACCCATAATCTCGATTATGGGTTATCTAG-TTCACTTACATACGATTGAATAGTATGGAAATGCATTCATTTCCTATGCATTGACACGATCTATGATACTATCGGAGTGAAACAGGGGATCTAAGGAAGAACAGAGGCTAGGCTATATTAGTAACAAGTAAACCC-----TTTGTGTGTCTGTCAAAAGTCTCCAAGTATTTTGGGGATAAACACCGATCCTAAGGTCTGAGACGACCCAGAAAGCATT--TGATCCTATCATGATCCACCTTGTAAGCCTACTTGGGTATTGAGTATTTACTTGTAAGAACCAAAATTTTTGCGCTGGATAGTTGCAACTCCGGAAAAAGAAATCC------------------AGTCAAATTTTTC------TTTTAAT--------AAAATCATTCATATTATCATA-----TATGTGTGGATCTAGATAACATATAGATTTTATATGGATTCCTTATGGTTCTTTTTCTTTTTGCTCGAGCCGTATGATAAAAAATTATCATGTCCGGTTCCTTCGGGGGATGGAT-----CTATAAAAATTCACCTATCCCAATAACAAAAAAACCTGACCTGAATGATCCTGTATTAAGAGCTAAATTGGCTAAAGGTATGGGTCATAATTATTACGGAGAGCCCGCATGGCCCAATGATCTTTTATATATTTTTCCAGTAGAGATTG-----------------------AAG-----AAATCAATGCAATATTTTTTAGGAAAGATAAAACTGGATGAATTCAAATTCGTTCAAATGGG-----------------------ATTGGAAGGTTCCTTATTTTCATTTAGGGTGTTCGTTTTATTTCTTCCCTTAGGACTTTGGTGTAGTTTATGCTCTCTCTTCTGGAATCG-AATTGTTGT-AACTGGACGCTTCTATCCT-CTAGCTAGTA--GGGATAGAAC-----AAAAAAAATATTTTCA------TTTTTTAATGAATTTTTTCTCATTTATCCGATTTATCAAATTTGAAACAAAAAGATACATTTTTTCAATGAACACA----------AAAAAATCCTAAAGTT---------ATACTATACAAAGGGTTGTCAAAATGGAATCAATTAGTTTCACCAATTCCTTC-----------------------ATTTTTACTAATGATCTTATA----TATGCCC-------TTCTATAGATATATATAGAG----------------ATAGAGAAACCC----------------------------ATTTTTCT-------TATTATA-----GATAAATAGGTTGATGGGGAAAATAAGACCCCGC-------CCTCGAAATGATAAAATCTACTAAAAAGAAAGGTAAAACCTTGTATCTTGTCTTTA--------------------TTTTCAAAAAAA--------TTCTTTTTTTTATTTAGAAATTTAGTAAACAGAAGCATTTTTATTCTACTTCCATTCCCTATTGTTTTCGGCC-------AATGAATAGGGAATGGGAATTATTGATTTTATTTTTAGATTAACAATGAAATCAGAC---------AGTCAAATCAATTGGGATTATTCTAACGTTTTATGA--------CTTATTTGTTTGTCGTACAAAAAAACTTTTTACATTTCCGGTAGGTCGTACAAAAAGAC-TTTTTGAATTCCCGGT-ACAAAGGCCATCTCCCCCAATTGAACAAGAGAATAATGACTATGTTACATTACACATGAAGTAA-----GAAAAAAGTC--TTG-CTTTCTCTTTCTTTATGATATAG-------ATATGTACAACTTTGACCAGCAATTTCATTTAGATC-TAAGTAAGGGCTCGAAAGAT-CCAATAGACAAAT-------------ATAAAGAAAAATAAAGAAGACCC-----CTTTAATTTTGTTCCCTTTATTCCCACGGCCTGGCCTGGTCAATACCTAGCCGGGCC----TTTTTTTGTTCCAACAAATCCTAGCTAAAAGAATTTAGCTG-------------CTTTGAACACA---------------AAAATGCTTGCTATTAAAGCAGC-------------------AATAAAAAGATGAGGGGTTATTTCCATTCTTACTTAT--------------------------------------TATA--TATTATATTATTTATATTA-------------AATATATATAAATTATATAAAATCAAAGTATCCTTTCTTATT-----ATTCCTTCTTCCCTTTTGAGTTACTTGACGACCTTACGGGAAT--ATAAAATGAAACTG-----TGGGTTCTTA---AATAATAATGAATGCATTTTTCTGTTATGATTTCAGTGGTTTTAGTGAGCCATATCTATCAAAATCCCCCCAGCAAAAGAAAAAATAGAACTT--GTTATTTC----------------ATTTAGTTATTTAAAAGAGCCC-----TCCTTTCCGGAATCTCATTAAATCGAAATCCCCCGCGAA-AAACGT-CGACACTCTCATTTTCATGA-----TTATGATC-CTA--T-CTTTATTACGCCTAATTCCTCGGTT-CGACAAAAAGTTCATTTGTATATAATAAGTATTAG-TATATCTAATACCTTACCCAGCCCATTTGGAAATTTTGGTTCAAACTCTTCGCTACTGGGTAAAAGATGCCCCCTCTTTGCATTTATTACGATTCTTTCTTCACGACTATCGGCATTGGACTAGTCTTTTTATTCCAAAGAAAGCCAGTTCTTTTTTTTCAAAACGAAAG------------------CAAAGATTATTCTTCTTCCTATATAATTCTCATGTATGTGAATACGAATCCATCTTCGTCTTTCTCCGTAACCAATCTTCTCATTTACAATCAACATCTTCTGGAGCCCTTCTTGAACGAATATATTTCTATGAAAAAATAGAACATCTTGTCGAAGTCTTTGCTAAA-GATTTTCAAGGCAATCTATGGTTGTTCAAGGATCCTTTCATGCATTATGTTAGGTATCAAGGAAAGTCAATTCTCGCTTCAAAGGAGAACTTTCTTTTGATGCGTAAATGGAAATATTACTTTGTACGTTTCTGGCAATGTCATTTTTACCAGTGGTTTCAACCAGGAAGGATCTATATAAACCAATTATCCAAACATTCCCTCGACCTTCTGGGCTATCTTTCAAGTGTGCGGCTAAATCCTTTAACGATACGCAGTCAAATGCTAGAAAATTCATTTCTAATTGATAATGCTGTTAAGAAGTTCGATACCATTGTTCCAATTATTCCTCTGATTGGATCATTGGTTAAAGCGAAATTTTGTAACGTATTAGGGCATCCTGTTAGTAAGGTAGTTTGGGCAGATTTATCAGATTTAAGACAT-AAAATTTACCCTACTTCTTTATCTTTC-TAGGAAGGGCCTTTCATGAGTTGAATAGAGATTTTCA-TTTTTTATTCATCATTCGGGTTGATGAACTAAA-CCAGATAGTTATATGAGTGAAAGAAACAGCTTATAAATTTGCAGTAAAAAGATTGAGTCTCATTTT-CTATGTAC-AAGAG-TTAAGTGAAAGTAACCATAAACATTA-GAAACGGTTTACCCCAAGATTGGTCAATTAGTGATCATGGCTTGAAGCGGGTGC-AAAAGATCAACTATATGGGG--TTTTACTATCTATTACCATACATGTATTACCCTAACGGGCGATTAGCAAAAAGAGGTGGATAGTTAGGAACACCAAAGTACACAAAGGATTCGTAATAGAGATTATGTAAGTTATTCAACAGAATTTTTCTGTGCATAA-AAGGAATTCTGATTGGGACTTTAAGTTGGTAGAAATGATGAAGAAGTACTCCCCCTGATTCCGATCCAGAGTATACTCCTATCCACCGATTAAGTAAATAACTATCAAGAACGAAGTAATCCTTT-ACTTT------GTTTAAAGTCCCTTTTTCTGAGAAAGGAGAATAGGAACGAAAAAAATC-------AAATAG-AAAGA-ATAGAATTGCACTAGAAAGAAAAGAAAGAGATCTTTTTTTATTCTTTCTTTCC-------------TCTATTTAGAAAGATAGAATTCTTGTCATCATTCGTGAACTAATGCGATGCCTAATTGTTTTTCGTAATCGAAAATGCTAGGTTG--------AAATATCTATGAATA-TTGCTACAAGAAAGA-TTTTATTGAAAA-TTTAAGTTATCACTCAAC-AAAGAAAAATAAAAA-TTATT-----AAAAGATAAGATCAA-TTCCG-AAGCGCTTTA-TTTTCAATATAGCAGACAGAATTCCATTGTCTAATTCGGG--ACTTWACAGTAGATTTTGATTCTATCTATCCTACGAATATAT-------------------CAAGATAAATAATAGCGAACTGGTCCTTAGA-TTTATTTGTGA-CCTTTGAGGAGCC-GTATGAGATGAAAATCTCATGTACGGTATTTAGATTA---------------------------------------------------------------------------------------------------------------------------------------------------------------------------------CTAAA------AATATTTACGATTTTTCTTT-AA-----ATAAAAAAAAA--ATATGTAAGTCAA--------------------------------------------------------------------------------TGTGAAAT-AAAAAAGGAGCAATAATCCCCTTCTTGTTCTATCAAGAAGGGCGATATTGCTCCTTTTTT-ATTTCTTTCAAATACTCGTATACACTAAGGCCAGGTCTTATCCWTTTATAGATGGAGCTTCAAGAGCAGCTAGGTCTAGAGGGAAGTTATGAGCATTACGTTCATGCATAACTTCCATACCAAGGTTAGCGCGGTTAATGATATCAGCCCAAGTATTAATTACACGACCTTGACTATCAACTACAGATTGGTTGAAATTAAAACCATTTAGGTTGAAAGCCATAGTGCTGATACCTAAAGCAGTGAACCARATACCTACTACAGGCCAAGCAGCTAGGAARAAATGTAAAGAACGAGAGTTGTTGAAACTAGCATATTGRAARATCAATCGGCCAAAATAACCATGAGCAGCTACAATATTATAAGTTTCTTCTTCTTGA-CCGAATC---TGTAACCTTCATTAGCAATAAATGCAAGAATATTTACTTTCCATAATCTCATCGTTTTTTTACTTCAAAATAACT-CGGGATTTAATCCCATAGAGATAATAAATCTTTCGCCTGTCAATTCA----ATGAATTACCTCTCGATGATC-TTGAAATCGGATCAATATCATGAATAACAATATCTGAGCTATCAAATCAATTCGTCGTCGAGAATTGAATAGTATAACATAGAAAGATCTTT-TATCCATACCG------AATCCAA------------------------AATTTCTTTATTTATCAATCATTCTTTTCTGTTCTTTCTTTATTTACAACCTATCTTAGGTCCTCCTTGTACAATCATCGGATAAAGTATCGTCTGACCGCCCGTCCGTTTCCATTAGTCACAAACGCCCAACAAACAATAGAAGCGAAGTGGAAAAAGAAATAAGTTACGTTCTAAACTCCG----TTTTTTTAATGATCTAGTTTTCTTGGAAGACAAAGAAGTGTGATAAAGAGGAGTTCCGGGATAAAGGATGTAATATTCCATCAAACTAACTATTTGAGTTTGGGTTTTGTTCGTTYTTCGACGGG--CCCT------AAAAAAAAAAATAG-AAAAAAAATAGGAAGGAAAAAT-GATTTATTCCCCCGCTACTTGTTAAGCTAAAAAAGGGGTGGGATCTTTGATTGATCTTTATTTTTCTTTTA-CCCCCCCTTCCTT---------------------------------------------------------------------------------------------------------------------------------------------------------------------------------------------------------------------------------------------------------------------------------------------------------------AACGCTCTCAATAATTGTACTATTCTACATATGTCTTTCTCCTACCAATCAGTATTATTTGAAATAATGAAAATTCCCCTATTTGTTTGATGAGAAGTGC----GAAATGCCAAAGGAAA--GAAAAAAGAACCCCCTT-GGGAATGAAAATTCTGCTCCCCGTGCCCCCTTTAACGAGAAAAAGAAGATTACAAATTGACTTATTATACTCCTGACTATGAAACCAAAGATACTGATATCTTGGCAGCATT-CCGAGTAACTCCTCAACCTGGAGTTCCACCTGAAGAAGCAGGGGCCGCGGTAGCTGCCGAATCTTCAACTGGTACATGGACAACTGTGTGGACCGATGGACTTACCAGCCTTGATCGTTACAAAGGGCGATGCTACCACATCGAGCCCGTTGCTGGAGAAGAAAATCAATATATTGCTTATGTAGCTTACCCATTAGACCTTTTTGAAGAAGGTTCTGTTACTAACATGTTTACTTCTATTGTGGGTAATGTATTTGGGTTCAAAGCCCTGCGCGCTCTACGTCTGGAAGATCTGCGAATCCCTGTCGCTTATACTAAAACTTTCCAAGGCCCGCCTCATGGCATCCAAGTTGAGAGAGATAAATTGAACAAGTATGGTCGCCCCCTGTTGGGATGTACTATTAAACCTAAATTGGGGTTATCTGCTAAAAACTATGGTAGAGCGGTTTATGAATGTCTACGTGGTGGACTTGATTTTACCAAAGATGATGAGAA--CGT--GAACTC----------------------------------

Abelia_forrestii_BOP012329 CCTGAGCCAAATCCAGTTTTACGA----AAATAAGGGTTCAGAAAGCTAAAATC-AAAAAGGATAGGTGCAGAGACTCAATGGAAGCTGTTCTAACAAATGGAGTTGACTGTGTTGTGTTGGTAGAAAGAATCCTTCCATAGAAACTTCAGAAAGGATAAACGTATAAACATGGATATACGCATTGAAATACTAT-ATACTCTACCAAATGATTAATGACGACCCGAATCTGTATT--TA-----TATATATCAAAATGGGAGAATGGTTGTGAAGTGATTCCATATTGAAGAAAGAATCGAATATTTATTGATCAAATCATTCACTCCATAGTCTGATAGATC-TTTTGAAGAGCTTATTAATTGGACGAGAATAAAGATAGAGTCCCATTCTACATGTCAATACCGGCAACAATGAAATTTATAGTAAAAGGAAAATCCGTCGACTTTAGAAATCGTGAGGGTTCAAGTCCCTCTATCCCCAAAAAACCCATAGGGACTCCCTAATTATTTATCCTCTCCTT-TTATCC-TTTTTTGTTAGCGGTTAAAAATTCG------TTATCTTTCTCATTCACCCTACTCTTTTACAAAGAGATCTGAGCGGAAATGTTTTTCTCTTATC----ACAAGTCTTGTGATCTAAG----ATAA-TACGTGTACAAATGAACATCTTTGAGTAAGGAATCCCCATTTAAATGATTCATGGTCAA------TGACATTATTCATACTGAAACTTACAAAGTCTTCCTT-TTGAAGATCCAAGAAATTCCAGGACCTGGCTAAGACTTTGTAAGACCCTTTCAATTGACA-TAGACCCGAGTTATCTAGT--AAAATGAGGATGCAGCGGTATTTATGTTAATGCACTTCCCAATGATACGTAAACAAGGCATTTCTGGTCCTTTA--------TAGAGAAGATCTATCATAGATATTTGTAATCTATCATTTATCGCTT-GGGGAGGAACAATAGTATTTCATTGCTACAAGTATGGATTATTGAGAATAATAAGACATGTATTTGGATATTTCCCTTGAACTCCGCAATCTTTTTGATTTGACATGGATAGTTGAAGGGAATTTTCCGAAGAAAAAATGGATTAGATTATGGGAGTGTGTGACTTGAACTATTGATTGGTCTGTGCAGATATATGCCTTTCGCTATCTGCCACATTGGAATTCACAACCAAATGTGTCTTTGTTCCAACCATTGCGTAAGCCC----------CATACAGAGGATAGGCTGGTTCACTTGAAGAGAATCTTTTCTATGATCAGATCCGAATCATGTCGTACATGAGCAGGTTCCGTAAGATCCAGTAG-----AATAAGTG-AACTAGATAACCCATAATCGAGATTATGGGTTATCTAG-TTCACTTACATACGATTGAATAGTATGGAAATGCATTCATTTCCTATGCATTGACACGATCTATGATACTATCGGAGTGAAACAGGGGATCTAAGGAAGAACAGAGGCTAGGCTATATTAGTAACAAGTAAACCC-----TTTGTGTGTCTGTCAAAAGTCTCCAAGTATTTTGGGGATAAACACCGATCCTAAGGTCTGAGACGACCCAGAAAGCATT--TGATCCTATCATGATCCACCTTGTAAGCCTACTTGGGTATTGAGTATTTACTTGTAAGAACCAAAATTTTTGCGCTGGATAGTTGCACCTCTGGAAAAAGAAATCC------------------AGTCAAATTTTTC------TTTTAAT--------AAAATCATTCATATTATCATA-----TATGTGTGGATCTAGATAACATATAGATTTTATATGGATTCCTTATGGTTCTTTTTCTTTTTGCTCGAGCCGTATGATGAAAAATTATCATGTCCGGTTCCTTCGGGGGATGGAT-----CTATAAAAATTCACCTATCCCAATAACAAAAAAACCTGACCTGAATGATCCTGTATTAAGAGCTAAATTGGCTAAAGGTATGGGTCATAATTATTACGGAGAGCCCGCATGGCCCAATGATCTTTTATATATTTTTCCAGTA-AGATTG-----------------------AAG-----AAATCAATGCAATATTTTTTAGGAAAGATAAAACTGGATGAATTCAAATTCGTTCAAATGGGATTGGAAGGTTCCTTATTTTCATATTGGAAGGTTCCTTATTTTCATTTAGGGTGTTCGTTTTATTTCTTCCCTTAGGACTTTGGTGTAGTTTATGCTCTCTCTCCTGGAATCG-AATTGTTGT-AACTGGACGCTTCTATCCT-CTAGCTAGTA--GGGATAGAAC------AAAAAAATATTTTCA------TTTTTTAATGAATTTTTTCTCATTTATCCGATTTATCAAATTTGAAACAAAAAGATACATTTTTTCAATGAACACA----------AAAAAATCCTAAAGTT---------ATACTATACAAAGGGTTGTCAAAATGGAATCAATTAGTTTCACCAATTCCTTC-----------------------ATTTTTACTAATGATCTTATA----TATGCCC-------TTCTATAGATATATATAGAG----------------ATAGAGAAACCC----------------------------ATTTTTCT-------TATTATA-----GATAAATAGGTTGATGGGGAAAATAAGACCCCGC-------CCTCGAAATGATAAAATCTACTAAAAAGAAAGGTAAAACCTTGTATCTTGTCTTTA--------------------TTTTCAAAAAA---------TTCTTTTTTTAATTTAGAAATTTAGTAAACAGAAGCATTTTTATTCTACTTCCATTCCCTATTGTTTTCGGCC-------AATGAATAGGGAATGGGAATTATTCATTTTATTTTTAGATTAACAATGAAATCAGAC---------AGTCAAATCAATTGGGATTATTCTAACGTTTTATGA--------CTTATTTGTTTGTCGTACAAAAAAACTTTTTACATTTCCGGTAGGTCGTACAAAAAAAC-TTTTTGAATTCCCGGT-ACAAAG------------------------------------------CATACACATGAAGTAA-----GAAAAAAGTC--TTG-CTTTCTCTTTCTTTATGATATAG-------ATATGTACAACTTTGACCAGCAATTTCATTTAGATC-TAAGTAAGGGCTCGAAAGAT-CCAATAGACAAAT-------------ATAAAGAAAAATAAAGAAGACCC-----CTTTGATTTTGTTCCCTTTATTCCCACGGCCTGGCCTGGTCAATACCTAGCCGGGCC----TTTTTTTGTTCCAACAAATCCTAGCTAAAAGAATTTAGCTG-------------CTTTGAACACA---------------AAAATGCTTGCTATTAAAGCAGC-------------------AATAAAAAGATGAGGGGTTATTTCCATTCTTACTTAT--------------------------------------TATATATATTATTATTATTTATATTATA----------TATATATATAAATTATATAAAATCAAAGTATCCTTTCTTATT-----ATTCCTTCTTCCCTTTTGAGTTACTTGACAACCTTACGGGAAT--ATAAAATGAAACTG-----TGGGTTCTTA---AATAATAATGAATGCATTTTTCTGTTATGATTTCAGTGGTTTTAGTGAGCCATATCTATCAAAATCCCCCCAGCAAAAGAAAAAATAGAACTT--GTTATTTC----------------ATTTAGTTATTTAAATGAGCCC-----TCCTTTCCGGAATCTCATTAAATCGAAATCCCCCGCGAA-AAACGT-CGACACTCTCATTTTCATGA-----TTATGATC-CTA--T-CTTTATTACGCTCAATTCCTTG--------------------------------GTATTAG-TATATCTAATACCTTACCCAGCCCATTTGGAAATTTTGGTTCAAACTCTTCGCTACTGGGTAAAAGATGCCCCCTCTTTGCATTTATTACGATTCTTTCTTCACGACTATCGGCATTGGACTAGTCTTTTTATTCCAAAGAAAGCCAGTTCTTTTTTTTCAAAACGAAAG------------------CAAAGATTATTCTTCTTCCTATATAATTCTCATGTATGTGAATACGAATCCATCTTCGTCTTTCTCCGTAACCAATCTTCTCATTTACAATCAACATCTTCTGGAGCCCTTCTTGAACGAATATATTTCTATGAAAAAATAGAACATCTTGTCGAAGTCTTTGCTAAA-GATTTTCAAGGCAATCTATGGTTGTTCAAGGATCCTTTCATGCATTATGTTAGGTATCAAGGAAAGTCAATTCTCGCTTCAA-GGAGAACTTTCTTTTGATGCGTAAATGGAAATATTACTTTGTACGTTTCTGGCAATGTCATTTTTACCAGTGGTTTCAACCAGGAAGGATCTATATAAACCAATTATCCAAACATTCCCTCGACCTTCTGGGCTATCTTTCAAGTGTGCGGCTAAATCCTTTAACGATACGCAGTCAAATGCTAGAAAATTCATTTCTAATTGATAATGCTGTTAAGAAGTTCGATACCATTGTTCCAATTATTCCTCTGATTGGATCATTGGTTAAAGCGAAATTTTGTAACGTATTAGGGCATCCTGTTAGTAAGGTAGTTTGGGCAGATTTATCAGATTTAAGACAT-AAAATTTACCCTACTTCTTTATCTTTC-TAGGAAGGGCCTTTCATGAGTTGAATAGAGATTTTCA-TTTTTTATTCATCATTCGGGTTGATGAACTAAA-CCAGATAGTTATATGAGTGAAAGAAACAGCTTATAAATTTGCAGTAAAAAGATTGAGTCTCATTTT-CTATGTAC-AAGAG-TTAAGTGAAAGTAACCATAAACATTA-GAAACAGTTTACCCCAAGATTGGTTAATTAGTGATCATGGCTTGAAGCGGGTGC-AAAAGATCAACTATATGGGG-TTTTTACTATCTATTACCATACATGTATTACCCTAACGGGCGATTAGCAAAAAGAGGTGGATAGTTAGGAACACCAAGGTACACAAAGGATTCGTAATAGAGATTATGTAAATTATTCAACAGAATTTTTCTGTGCATAA-AAGGAATTCTGATTGGGACTTTAAGTTGGTAGAAATGATGAAGAAGTACTCCCCCTGATTCCGATCCAGAGTATACTCCTATCCACCGATTAAGTAAATAACTATCAAGAACGAAGTAATCCTTT-ACTTT------GTTTAAAGTCCCTTTTTCTGAGAAAGGAGAATAGGAACGAAAAAAATC-------AAATAG-AAAGA-ATAGAATTGCACTAG-----AAAGAAAGAGATCTTTTTTTATTCTTTCTTTCC-------------TCTATTTAGAAAGATAGAATTCTTGTCATCATTCGTGAACTAATGCGATGCCTAATTGTTTTTCGTAATCGAAAATGCTAGGTTG--------AAATATCTATGAATA-TTGCTACAAGAAAGA-TTTTATTGAAAG-TTTAAGTTATCACTCAAC-AAAGAAAAATAAAAA-TTATT-----AAAAGATAAGATCAA-TTCCG-AAGCGCTTTA-TTTTCAATATAGCAGACAGAATTCCATTGTCTAATTCGGG--ACTTTACAGTAGATTTTGATTCTATCTATCCTACGAATATAT-------------------CAAGATAAATAATAGCGAACTGGTCCTTAGA-TTTATTTGTGA-CCTTTGAGGAGCC-GTATGAGATGAAAATCTCATGTACGGTATTTAGATTA---------------------------------------------------------------------------------------------------------------------------------------------------------------------------------CTAAA------AATATTTACRATTTTTCTTT-AA-----ATTTTAAAAAA--ATATGTAAGTCAA--------------------------------------------------------------------------------TGTGAAAT-AAAAAAGGAGCAATAATCCCCTTCTTGTTCTATCAAG-AGGGCGATATTGCTCCTTTTTT-ATTTCTTTCAAATACTCGTATACACTAAGGCCGGGTCTTATCCATTTATAGATGGAGCTTCAAGAGCAGCTAGGTCTAGAGGGAAGTTATGAGCATTACGTTCATGCATAACTTCCATACCAAGGTTAGCGCGGTTAATGATATCAGCCCAAGTATTAATTACACGACCTTGACTATCAACTACAGATTGGTTGAAATTAAAACCATTTAGGTTGAAAGCCATAGTGCTGATACCTAAAGCAGTGAACCARATACCTACTACAGGCCAAGCAGCTAGGAAGAAATGTAAAGAACGAGAGTTGTTGAAACTAGCATATTGGAAGATCAATCGGCCAAAATAACCATGAGCAGCTACAATATTATAAGTTTCTTCTTCTTGA-CCGAATC---TGTAACCTTCATTAGCAATAAATGCAAGAATATTTAC-TTCCATAATCTCATCGTTTTTTTACTTCAAAATAACT-CGGGATTTAATCCCATAGAGATAATAAATCTTTCGCCTGTCAATTCA----ATGAATTACCTCTCGATGATC-TTGAAATCGGATCAATATCATGAATAACAATATCTGAGCTATCAAATCAATTYGTCGTCGAGAATTGAATAGTATAACATAGAAAGATCTTT-TATCCATACCG------AATCCAA------------------------AATTTCTTTATTTATCAATCATTCTTTTCTGTTCTTTCTTTATTTACAACCTATYTTAGGTCCTCCTTGTACAATCATCGGATAAAGTATYGTCTGACCGCCCGTCCGTTTCCATTAGTCACAAACGCCCAACAAACAATAGAAGCGAAGTGGAAAAAGAAATAAGTTACGTTCGAAACTCCG----TTTTTTTAATGATCTAGTTTTCTTGGAAGACAAAGAAGTGTGATAAAGAGGAGTTCCGGGATAAAGGATGTAATATTCCATCAAACTAACTATTTGAGTTTGGGTTTTGTTCGTTYTTCGACGGG--CCCT------AAAAAAAAAAATAG-AAAAAAAATAGGAAGGAAAAAT-GATTTATTCCCCTGCTACTTGTTAAGCTAAAAAAGGGGTGGGATCTTTGATTGATCTTTATTTTTCTTTTA--CCCCCCTTCCTT---------------------------------------------------------------------------------------------------------------------------------------------------------------------------------------------------------------------------------------------------------------------------------------------------------------AACGCTCTCAATAATTGTACTATTCTACATATGTCTTTCTCCTACCAATCAGTATTATTTGAAATAATGAAAATGCCCCTATTTGTTTGATGAGAAGTGC----GAAATGCCAAAGGAAA--GAAAAAAGAACCCCCTT-GGGAATG-AAATTCTGCTCCCCGTGCCCCCTTTAACAGAAAAGGGAAGATTACAAATTGACTTATTATACTCCTGACTATGAAACCAAAGATACTGATATCTTGGCAGCATT-CCGAGTAACTCCTCAACCTGGAGTTCCACCTGAAGAAGCAGGGGCCGCGGTAGCTGCCGAATCTTCAACTGGTACATGGACAACTGTGTGGACCGATGGACTTACCAGCCTTGATCGTTACAAAGGGCGATGCTACCACATCGAGCCCGTTGCTGGAGAAGAAAATCAATATATTGCTTATGTAGCTTACCCATTAGACCTTTTTGAAGAAGGTTCTGTTACTAACATGTTTACTTCTATTGTGGGTAATGTATTTGGGTTCAAAGCCCTGCGCGCTCTACGTCTGGAAGATCTGCGAATCCCTGCCGCTTATACTAAAACTTTCCAAGGCCCGCCTCATGGCATCCAAGTTGAGAGAGATAAATTGAACAAGTATGGTCGCCCCCTGTTGGGATGTACTATTAAACCTAAATTGGGGTTATCTGCTAAAAACTATGGTAGAGCGGTTTATGAATGTCTACGTGGTGGACTTGATTTTACCAAAGATGATGAGAA--CGT--GAACTCCCAACCATTTATGCGTT---GGAGAGATCGATTC

Abelia_forrestii_BOP022784 CCTGAGCCAAATCCAGTTTTACGA----AAACAAGGGTTCAGAAAGCTAAAATC-AAAAAGGATAGGTGCAGAGACTCAATGGAAGCTGTTCTAACAAATGGAGTTGACTGTGTTGTGTTGGTAGAAAGAATCCTTCCATAGAAACTTCAGAAAGGATAAACGTATAAACATGGATATACGCATTGAAATACTAT-ATACTCTACCAAATGATTAATGACGACCCGAATCTGTATT--TA-----TATATATCAAAATGGGAGAATGGTTGTGAAGTGATTCCATATTGAAGAAAGAATCGAATATTTATTGATCAAATCATTCACTCCATAGTCTGATAGATC-TTTTGAAGAGCTTATTAATTGGACGAGAATAAAGATAGAGTCCCATTCTACATGTCAATACCGGCAACAATGAAATTTATAGTAAAAGGAAAATCCGTCGACTTTAGAAATCGTGAGGGTTCAAGTCCCTCTATCCCCAAAAAACCCATATGGACTCCCTAATTATTTATCCTCTCCTT-TTATCC-TTTTTTGTTATCGGTTAAAAATTCG------TTATCTTTCTCATTCACCCTACTCTTTTACAAAGAGATCTGAGCGGAAATGTTTTTCTCTTATC----ACAAGTCTTGTGATCTAAG----ATAA-TACGTGTACAAATGAACATCTTTGAGTAAGGAATCCCCATTTAAATGATTCATGGTCAA------TGACATTATTCATACTGAAACTTACAAAGTCTTCCTT-TTGAAGATCCAAGAAATTCCAGGACCTGGATAAGACTTTGTAAGACCCTTTCAATTGACA-TAGACCCGAGTTATCTAGT--AAAATGAGGATGCAGCGGTATTTATGTTAATGCACTTCCCAATGATACGTAAACAAGGCATTTCTGGTCCTTTA--------TAGAGAAGATCTATCATAGATATTTGTAATCTATCATTTATCGCTT-GGGGAGGAACAATAGTATTTCATTGCTACAAGTATGGATTATTGAGAATAATAAGACATGTATTTGGATATTTCCCTTGAACTCCGCAATCTTTTTGATTTGACATGGATAGTTGAAGGGAATTTTCCGAAGAAAAAATGGATTAGATTATGGGAGTGTGTGACTTGAACTATTGATTGGTCTGTGCAGATATATGCCTTTCGCTATCTGCCACATTGGAATTCACAACCAAATGTGTCTTTGTTCCAACCATTGCGTAAGCCC----------CATACAGAGGATAGGCTGGTTCACTTGAAGAGAATCTTTTCTATGATCAGATCCGAATCATGTCGTACATGAGCAGGTTCCGTAAGATCCAGTAG-----AATAAGTG-AACTAGATAACCCATAATCGAGATTATGGGTTATCTAG-TTCACTTACATACGATTGAATAGTATGGAAATGCATTCATTTCCTATGCATTGACACGATCTATGATACTATCGGAGTGAAACAGGGGATCTAAGGAAGAACAGAGGCTAGGCTATATTAGTAACAAGTAAACCC-----TTTGTGTGTCTGTCAAAAGTCTCCAAGTATTTTGGGGATAAACACCGATCCTAAGGTCTGAGACGACCCAGAAAGCATT--TGATCCTATCATGATCCACCTTGTAAGCCTACTTGGGTATTGAGTATTTACTTGTAAGAACCAAAATTTTTGCGCTGGATAGTTGCACCTCCGGAAAAAGAAATCC------------------AGTCAAATTTTTC------TTTTAAT--------AAAATCATTCATATTATCATA-----TATGTGTGGATCTAGATAACATATAGATTTTATATGGATTCCTTATGGTTCTTTTTCTTTTTGCTCGAGCCGTATGATGAAAAATTATCATGTCCGGTTCCTTCGGGGGATGGAT-----CTATAAAAATTCACCTATCCCAATAACAAAAAAACCTGACCTGAATGATCCTGTATTAAGAGCTAAATTGGCTAAAGGTATGGGTCATAATTATTACGGAGAGCCCGCATGGCCCAATGATCTTTTATATATTTTTCCAGTA-AGATTG-----------------------AAG-----AAATCAATGCAATATTTTTTAGGAAAGATAAAACTGGATGAATTCAAATTCGTTCAAATGGGATTGGAAGGTTCCTTATTTTCATATTGGAAGGTTCCTTATTTTCATTTAGGGTGTTCGTTTTATTTCTTCCCTTAGGACTTTGGTGTAGTTTATGCTCTCTCTCCTGGAATCG-AATTGTTGT-AACTGGACGCTTCTATCCT-CTAGCTAGTA--GGGATAGAAC------AAAAAAATATTTTCA------TTTTTTAATGAATTTTTTCTCATTTATCCGATTTATCAAATTTGAAACAAAAAGATACATTTTTTCAATGAACACA----------AAAAAATCCTAAAGTT---------ATACTATACAAAGGGTTGTCAAAATGGAATCAATTAGTTTCACCAATTCCTTC-----------------------ATTTTTACTAATGATCTTATA----TATGCCC-------TTCTATAGATATATATAGAG----------------ATAGAGAAACCC----------------------------ATTTTTCT-------TATTATA-----GATAAATAGGTTGATGGGGAAAATAAGACCCCGC-------CCTCGAAATGATAAAATCTACTAAAAAGAAAGGTAAAACCTTGTATCTTGTCTTTA--------------------TTTTCAAAAAA---------TTCTTTTTTTAATTTAGAAATTTAGTAAACAGAAGCATTTTTATTCTACTTCCATTCCCTATTGTTTTCGGCC-------AATGAATAGGGAATGGGAATTATTCATTTTATTTTTAGATTAACAATGAAATCAGAC---------AGTCAAATCAATTGGGATTATTCTAACGTTTTATGA--------CTTATTTGTTTGTCGTACAAAAAAACTTTTTACATTTCCGGTAGGTCGTACAAAAAAAC-TTTTTGAATTCCCGGT-ACAAAG------------------------------------------CATACACATGAAGTAA-----GAAAAAAGTC--TTG-CTTTCTCTTTCTTTATGATATAG-------ATATGTACAACTTTGACCAGCAATTTCATTTAGATC-TAAGTAAGGGCTCGAAAGAT-CCAATAGACAAAT-------------ATAAAGAAAAATAAAGAAGACCC-----CTTTGATTTTGTTCCCTTTATTCCCACGGCCTGGCCTGGTCAATACCTAGCCGGGCC----TTTTTTTGTTCCAACAAATCCTAGCTAAAAGAATTTAGCTG-------------CTTTGAACACA---------------AAAATGCTTGCTATTAAAGCAGC-------------------AATAAAAAGATGAGGGGTTATTTCCATTCTTACTTAT--------------------------------------TATATATATTATTATTATTTATATTATA----------TATATATATAAATTATATAAAATCAAAGTATCCTTTCTTATT-----ATTCCTTCTTCCCTTTTGAGTTACTTGACAACCTTACGGGAAT--ATAAAATGAAACTG-----TGGGTTCTTA---AATAATAATGAATGCATTTTTCTGTTATGATTTCAGTGGTTTTAGTGAGCCATATCTATCAAAATCCCCCCAGCAAAAGAAAAAATAGAACTT--GTTATTTC----------------ATTTAGTTATTTAAATGAGCCC-----TCCTTTCCGGAATCTCATTAAATCGAAATCCCCCGCGAA-AAACGT-CGACACTCTCATTTTCATGA-----TTATGATC-CTA--T-CTTTATTACGCTCAATTCCTTG--------------------------------GTATTAG-TATATCTAATACCTTACCCAGCCCATTTGGAAATTTTGGTTCAAACTCTTCGCTACTGGGTAAAAGATGCCCCCTCTTTGCATTTATTACGATTCTTTCTTCACGACTATCGGCATTGGACTAGTCTTTTTATTCCAAAGAAAGCCAGTTCTTTTTTTTCAAAACGAAAG------------------CAAAGATTATTCTTCTTCCTATATAATTCTCATGTATGTGAATACGAATCCATCTTCGTCTTTCTCCGTAACCAATCTTCTCATTTACAATCAACATCTTCTGGAGCCCTTCTTGAACGAATATATTTCTATGAAAAAATAGAACATCTTGTCGAAGTCTTTGCTAAA-GATTTTCAAGGCAATCTATGGTTGTTCAAGGATCCTTTCATGCATTATGTTAGGTATCAAGGAAAGTCAATTCTCGCTTCAAAGGAGAACTTTCTTTTGATGCGTAAATGGAAATATTACTTTGTACGTTTCTGGCAATGTCATTTTTACCAGTGGTTTCAACCAGGAAGGATCTATATAAACCAATTATCCAAACATTCCCTCGACCTTCTGGGCTATCTTTCAAGTGTGCGGCTAAATCCTTTAACGATACGCAGTCAAATGCTAGAAAATTCATTTCTAATTGATAATGCTGTTAAGAAGTTCGATACCATTGTTCCAATTATTCCTCTGATTGGATCATTGGTTAAAGCGAAATTTTGTAACGTATTAGGGCATCCTGTTAGTAAGGTAGTTTGGGCAGATT-------------------------------------------------------------------------GAGATTTTCA-TTTTTTATTCATCATTCGGGTTGATGAACTAAA-CCAGATAGTTATATGAGTGAAAGAAACAGCTTATAAATTTGCAGTAAAAAGATTGAGTCTCATTTT-CTATGTAC-AAGAG-TTAAGTGAAAGTAACCATAAACATTA-GAAACAGTTTACCCCAAGATTGGTTAATTAGTGATCATGGCTTGAAGCGGGTGC-AAAAGATCAACTATATGGGG-TTTTTACTATCTATTACCATACATGTATTACCCTAACGGGCGATTAGCAAAAAGAGGTGGATAGTTAGGAACACCAAGGTACACAAAGGATTCGTAATAGAGATTATGTAAATTATTCAACAGAATTTTTCTGTGCATAA-AAGGAATTCTGATTGGGACTTTAAGTTGGTAGAAATGATGAAGAAGTACTCCCCCTGATTCCGATCCAGAGTATACTCCTATCCACCGATTAAGTAAATAACTATCAAGAACGAAGTAATCCTTT-ACTTT------GTTTAAAGTCCCTTTTTCTGAGAAAGGAGAATAGGAACGAAAAAAATC-------AAATAG-AAAGA-ATAGAATTGCACTAG-----AAAGAAAGAGATCTTTTTTTATTCTTTCTTTCC-------------TCTATTTAGAAAGATAGAATTCTTGTCATCATTCGTGAACTAATGCGATGCCTAATTGTTTTTCGTAATCGAAAATGCTAGGTTGAAATATCTAAATATCTATGAATA-TTGCTACAAGAAAGA-TTTTATTGAAAG-TTTAAGTTATCACTCAAC-AAAGAAAAATAAAAA-TTATT-----AAAAGATAAGATCAA-TTCCG-AAGCGCTTTA-TTTTCAATATAGCAGACAGAATTCCATTGTCTAATTCGGG--ACTTTACAGTAGATTTTGATTCTATCTATCCTACGAATATAT-------------------CAAGATAAATAATAGCGAACTGGTCCTTAGA-TTTATTTGTGA-CCTTTGAGGAGCC-GTAT----------------------TATTTAGATTA---------------------------------------------------------------------------------------------------------------------------------------------------------------------------------CTAAA------AATATTTACRATTTTTCTTT-AA-----ATTTTAAAAAA--ATATGTAAGTCAA--------------------------------------------------------------------------------TGTGAAAT-AAAAAAGGAGCAATAATCCCCTTCTTGTTCTATCAAG-AGGGCGATATTGCTCCTTTTTT-ATTTCTTTCAAATACTCGTATACACTAAGGCCGGGTCTTATCCATTTATAGATGGAGCTTCAAGAGCAGCTAGGTCTAGAGGGAAGTTATGAGCATTACGTTCATGCATAACTTCCATACCAAGGTTAGCGCGGTTAATGATATCAGCCCAAGTATTAATTACACGACCTTGACTATCAACTACAGATTGGTTGAAATTAAAACCATTTAGGTTGAAAGCCATAGTGCTGATACCTAAAGCAGTGAACCARATACCTACTACAGGCCAAGCAGCTAGGAAGAAATGTAAAGAACGAGAGTTGTTGAAACTAGCATATTGGAAGATCAATCGGCCAAAATAACCATGAGCAGCTACAATATTATAAGTTTCTTCTTCTTGA-CCGAATC---TGTAACCTTCATTAGCAATAAATGCAAGAATATTTAC-TTCCATAATCTCATCGTTTTTTTACTTCAAAATAACT-CGGGATTTAATCCCATAGAGATAATAAATCTTTCGCCTGTCAATTCA----ATGAATTACCTCTCGATGATC-TTGAAATCGGATCAATATCATGAATAACAATATCTGAGCTATCAAATCAATTYGTCGTCGAGAATTGAATAGTATAACATAGAAAGATCTTT-TATCCATACCG------AATCCAA------------------------AATTTCTTTATTTATCAATCATTCTTTTCTGTTCTTTCTTTATTTACAACCTATYTTAGGTCCTCCTTGTACAATCATCGGATAAAGTATYGTCTGACCGCCCGTCCGTTTCCATTAGTCACAAACGCCCAACAAACAATAGAAGCGAAGTGGAAAAAGAAATAAGTTACGTTCGAAACTCCG----TTTTTTTAATGATCTAGTTTTCTTGGAAGACAAAGAAGTGTGATAAAGAGGAGTTCCGGGATAAAGGATGTAATATTCCATCAAACTAACTATTTGAGTTTGGGTTTTGTTCGTTYTTCGACGGG--CCCT------AAAAAAAAAAATAG-AAAAAAAATAGGAAGGAAAAAT-GATTTATTCCCCTGCTACTTGTTAAGCTAAAAAAGGGGTGGGATCTTTGATTGATCTTTATTTTTCTTTTA--CCCCCCTTCCTT---------------------------------------------------------------------------------------------------------------------------------------------------------------------------------------------------------------------------------------------------------------------------------------------------------------AACGCTCTCAATAATTGTACTATTCTACATATGTCTTTCTCCTACCAATCAGTATTATTTGAAATAATGAAAATGCCCCTATTTGTTTGATGAGAAGTGC----GAAATGCCAAAGGAAA--GAAAAAAGAACCCCCTT-GGGAATG-AAATTCTGCTCCCCGTGCCCCCTTTAACAGAAAAGGG--------------------------------------------------------------------------AACTCCTCAACCTGGAGTTCCACCTGAAGAAGCAGGGGCCGCGGTAGCTGCCGAATCTTCAACTGGTACATGGACAACTGTGTGGACCGATGGACTTACCAGCCTTGATCGTTACAAAGGGCGATGCTACCACATCGAGCCCGTTGCTGGAGAAGAAAATCAATATATTGCTTATGTAGCTTACCCATTAGACCTTTTTGAAGAAGGTTCTGTTACTAACATGTTTACTTCTATTGTGGGTAATGTATTTGGGTTCAAAGCCCTGCGCGCTCTACGTCTGGAAGATCTGCGAATCCCTGCCGCTTATACTAAAACTTTCCAAGGCCCGCCTCATGGCATCCAAGTTGAGAGAGATAAATTGAACAAGTATGGTCGCCCCCTGTTGGGATGTACTATTAAACCTAAATTGGGGTTATCTGCTAAAAACTATGGTAGAGCGGTTTATGAATGTCTACGTGGTGGACTTGATTTTACCAAAGATGATGAGAA--CGT--GAACTCCCAACCATTTATGC--------------------

Abelia_grandiflora_BOP012207 CCTGAGCCAAATCCAGTTTTACGA----AAACAAGGGTTCAGAAAGCTAAAATC-AAAAAGGATAGGTGCAGAGACTCAATGGAAGCTGTTCTAACAAATGGAGTTGACTGTGTTGTGTTGGTAGAAAGAATCCTTCCATAGAAACTTCAGAAAGGATAAACGTATAAACATGGATATACGCATTGAAATACTAT-ATACTCTACCAAATGATTAATGACGACCCGAATCTGTATTTATA-----TATATATCAAAATGGGAGAATGGTTGTGAAGTGATTCCATATTGAAGAAAGAATCGAATATTTATTGATCAAATCATTCACTCCATAGTCTGATAGATC-TTTTGAAGAACTGATTAATTGGACGAGAATAAAGATAGAGTCCCATTCTACATGTCAATACTGGCAACAATGAAATTTATAGTAAAAGGAAAATCCGTCGACTTTAGAAATCGTGAGGGTTCAAGTCCCTCTATCCCCAAAAAACCCATATGGACTCCCTAATTATTTATCCTCTCCTT-TTATCC-TTTTTTGTTAGCGGTTAAAAATTCG------TTATCTTTCTCATTCACCCTACTCTTTTACAAAGAGATCTGAGCAGAAATGTTTTTCTCTTATC----ACAAGTCTTGTGATCTAAG----ATAA-TACGTGTACAAATGAACATCTTTGAGTAAGGAATCCCCATTTAAATGATTCATGGTCAA------TGTCATTATTCATACTGAAACTTACAAAGTCTTCCTT-TTGAAGATCCAAGAAATTCCAGGACCTGGATAAGACTTTGTAAGACCCTTTCAATTGACA-TAGACCCGAGTTATCTAGC--AAAATGAGGATGCAGCGGTATTTATGTTAATGCACTTCCCAATGATACGTAAACAAGGCATTTCTGGTCCTTTA--------TAGAAAAGATCTATCATAGATATTTGTAATCTATCATTTATCGCTTGGGGGAGGAACAATAGTATTTCATTGCTACAAGTATGGATTATTGAGAATAATAAGACATGTATTTGGATATTTCCCTTGAACTCCGCAATCTTTTTGATTTGACATGGATAGTTGAAGGGAATTTTCCGAAGAAAAAATGGATTAGATTATGGGAGTGTGTGACTTGAACTATTGATTGGTCTGTGCAGATATATGCCTTTCGCTATCTGCCACATTGGAATTCACAACCAAATGTGTCTTTGTTCCAACCATTGCGTAAGCCC----------CATACAGAGGATAGGCTGGTTCACTTGAAGAGAATCTTTTCTATGATCAGATCCGAATCATGTCGTACATGAGCAGGTTCCGTAAGATCCAGTAG-----AATAAGTG-AACTAGATAACCCATAATCTCGATTATGGGTTATCTAG-TTCACTTACATACGATTGAATAGTATGGAAATGCATTCATTTCCTATGCATTGACACGATCTATGATACTATCGGAGTGAAACAGGGGATCTAAGGAAGAACAGAGGCTAGGCTATATTAGTAACAAGTAAACCC-----TTTGTGTGTCTGTCAAAAGTCTCCAAGTATTTTGGGGATAAACACCGATCCTAAGGTCTGAGACGACCCAGAAAGCATT--TGATCCTATCATGATCCACCTTGTAAGCCTACTTGGGTATTGAGTATTTACTTGTAAGAACCAAAATTTTTGCGCTGGATAGTTGTAACTCCGGAAAAAGAAATCC------------------AGTCAAATCTTTC------TTTTAAT--------AAAATCATTCATATTATCATA-----TATGTGTGGATCTAGATAACATATAGATTTTATATGGATTCCTTATGGTTCTTTTTCTTTTTGCTCGAGCCGTATGATGAAAAATTATCATGTCCGGTTCCTTCGGGGGATGGAT-----CTATAAAAATTCACCTATCCCAATAACAAAAAAACCTGACCTGAATGATCCTGTATTAAGAGCTAAATTGGCTAAAGGTATGGGTCATAATTATTACGGAGAGCCCGCATGGCCCAATGATCTTTTATATATTTTTCCAGTAGAGATTG-----------------------AAG-----AAACCAATGCAATATTTTTTAGGAAAGATAAAACTGGATGAATTCAAATTCGTTCAAATGGG-----------------------ACTGGAAGGTTCCTTATTTTCATTTAGGGTGTTCGTTTTATTTCTTCCCTTAGGACTTTGGTGTAGTTTATGCTCTCTCTCCTGGAATCG-AATTGTTGT-AACTGGACGCTTCTATCCT-CTAGCTAGTA--GGGATAGAAC-----AAAAAAAATATTTTCA------TTTTTTAATGAATTTTTTCTCATTTATCCGATTTATCAAATTTGAAACAAAAAGATACATTTTTTCAATGAACACA----------AAAAAATCCTAAAGTT---------ATACTATACAAAGGGTTGTCAAAATGGAATCAATTAGTTTCACCAATTCCTTCATTTTTATTTTTACTAAATTTTTATTTTTACTAATGATCTTATA----TATGCCC-------TTCTATAGATATATAGAG------------------ATAGAGAAACCC----------------------------ATTTTTCTTATTATATATTATA-----GATAAATAGGTTGATGGGGAAAATAAGACCCCGC-------CCTCGAAATGATAAAATCTACTAAAAAGAAAGGTAAAACCTTGTATCTTGTCTTTA-------------------TTTTCAAAAAAAAAAAATAATTTTTTTTTTGAATTTAGAAATTTAGTAAACAGAAGCATTTTTATTCTACTTCCATTCCCTATTGTTTTCGGCC-------AATGAATAGGGAATGGGAATTATTCATTTTATTTTTAGATTAACAATGAAATCAGAC---------AGTCAAATCAATTGGGATTATTCTAACGTTTTATGACTTATTTGCTTATTTGTTTGTCGTACAAAAAAACTTTTTACATTTCCGGTAGGTCGTACAAAAAAAC-TTTTTGAATTCCCGGT-ACAAAGGCCATCTCCCCCAATTGAACAAGAGAATAATGACTATGTTACATTACACATGAAGTAA-----GAAAAAAGTC--TTG-CTTTCTCTTTCTTTATGATATAG-------ATATGTACAACTTTGACCAGCAATTTCATTTAGATC-TAAGTAAGGGCTCGAAAGAT-CCAATAGACAAAT-------------ATAAAGAAAAATAAAGAAGACCC-----CTTTGATTTTGTTCCCTTTATTCCCACGGCCTGGCCTGGTCAATACCTAGCCGGGCC----TTTTTTTGTTCCAACAAATCCTAGCTAAAAGAATTTAGCTG-------------CTTTGAACACA---------------AAAATGCTTGCTATTAAAGCAGC-------------------AATAAAAAGATGAGGGGTTATTTCCATTCTTACTTATTATTCCATTCTTACTTAT--------------------TATA--TATTATA-------------------------TATATATATAAATTATATAAAATCAAAGTATCCTTTCTTATT-----ATTCCTTCTTCCCTTTTGAGTTACTTGACGACCTTACGGGAAT--ATAAAATGAAACTG-----TGGGTTCTTA---AATAATAATGAATGCATTTTTCTGTTATGATTTCAGTGGTTTTAGTGAGCCATATCTATCAAAATCCCCCCAGCAAAAGAAAAAATAGAACTT--GTTATTTC----------------ATTTAGTTATTTAAAAGAGCCC-----TCCTTTCCGGAATCTCATTAAATCGAAATCCCCCGCGAACAAACGT-CGACACTCTCATTTTCATGA-----TTATGATC-CTA--T-CTTTATTACGCCTAATTCCTCGGTT-CGACAAAAAGTTCATTTGTATATAATAAGTATTAG-TATATCTAATACCTTACCCAGCCCATTTGGAAATTTTGGTTCAAACTCTTCGCTACTGGGTAAAAGATGCCCCCTCTTTGCATTTATTACGATTCTTTCTTCACGACTATCGGCATTGGACTAGTCTTTTTATTCCAAAGAAAGCCAGTTCTTTTTTTTCAAAACGAAAG------------------CAAAGATTATTCTTCTTCCTATATAATTCTCATGTATGTGAATACGAATCCATCTTCGTCTTTCTCCGTAACCAATCTTCTCATTTACAATCAACATCTTCTGGAGCCCTTCTTGAACGAATATATTTCTATGAAAAAATAGAACATCTTGTCGAAGTCTTTGCTAAA-GATTTTCAAGGCAATCTATGGTTGGTCAAGGATCCTTTCATGCATTATGTTAGGTATCAAGGAAAGTCAATTCTCGCTTCAA-GGAGAACTTTCTTTTGATGCGTAAATGGAAATATTACTTTGTACGTTTCTGGCAATGTCATTTTTACCAGTGGTTTCAACCAGGAAGGATCTATATAAACCAATTATCCAAACATTCCCTCGACCTTCTGGGCTATCTTTCAAGTGTGCGGCTAAATCCTTTAACGATACGCAGTCAAATGCTAGAAAATTCATTTCTAATTGATAATGCTGTTAAGAAGTTCGATACCATTGTTCCAATCATTCCTCTGATTGGATCATTGGTTAAAGCGAAATTTTGTAACGTATTAGGGCATCCTGTTAGTAAGGTAGTTTGGGCAGATTTATCAGATTTAAGACAT-AAAATTTACCCTACTTCTTTATCTTTC-TAGGAAGGGCCTTTCATGAGTTGAATAGAGATTTTCA-TTTTTTATTCATCATTCGGGTTGATGACCTAAA-CCAGATAGTTATATGAGTGAAAGAAACAGCTTATAAATTTGCAGTAAAAAGATTGAGTCTCATTTT-CTATGTAC-AAGAG-TTAAGTGAAAGTAACCATAAACATTA-GAAACGGTTTACCCCAAGATTGGTTAATTAGTGATCATGGCTTGAAGCGGGTGC-AAAAGATCAACTATATGGGG-TTTTTACTATCTATTACCATACATGTATTACCCTAACGGGCGATTAGCAAAAAGAGGTGGATAGTTAGGAACACCAAAGTACACAAAGGATTCGTAATAGAGATTATGTAAGTTATTCAACAGAATTTTTCTGTGCATAA-AAGGAATTCTGATTGGGACTTTAAGTTGGTAGAAATGATGAAGAAGTACTCCCCCTGATTCCGATCCAGAGTATACTCCTATCCACCGATTAAGTAAATAACTATCAAGAACGAAGTAATCCTTT-ACTTT------GTTTAAAGTCCCTTTTTCTGAGAAAGGAGAATAGGAACGAAAAAAATC-------AAATAG-AAAGA-ATAGAATTGCACTAG-----AAAGAAAGAGATCTTTTTTTATTCTTTCTTTCC-------------TCTATTTAGAAAGATAGAATTCTTGTCATCATTCGTGAACTAATGCGATGCCTAATTGTTTTTCGTAATCGAAAATGCTAGGTTG--------AAATATCTATGAATA-TTGCTACAAGAAAGA-TTTTATTGAAAA-TTTAAGTTATCACTCAAC-AAAGAAAAATAAAAA-TTATT----------ATAAGATCAA-TTCCG-AAGCGCTTTA-TTTTCAATATAGCAGACAGAATTCCATTGTCTAATTCGGG--ACTTTACAGTAGATTTTGATTCTATCTATCCTACGAATATAT-------------------CAAGATAAATAATAGCGAACTGGTCCTTAGA-TTTATTTGTGA-CCTTTGAGGAGCC-GTATGAGATGAAAATCTCATGTACGGTATTTAGATTA---------------------------------------------------------------------------------------------------------------------------------------------------------------------------------CTAAA------AATATTTAGTATTTTTCTTT-AA-----ATTAAAAAAAAA-ATATGTAAGTCAA--------------------------------------------------------------------------------TGTGAAAT-AAAAAAGGAGCAATAATCCCCTTCTTGTTCTATCAAG-AGGGCGATATTGCTCCTTTTTT-ATTTCTTTCAAATACTCGTATACACTAAGGCCGGGTCTTATCCATTTATAGATGGAGCTTCAAGAGCAGCTAGGTCTAGAGGGAAGTTATGAGCATTACGTTCATGCATAACTTCCATACCAAGGTTAGCGCGGTTAATGATATCAGCCCAAGTATTAATTACACGACCTTGACTATCAACTACAGATTGGTTGAAATTAAAACCATTTAGGTTGAAAGCCATAGTGCTGATACCTAAAGCAGTGAACCARATACCTACTACAGGCCAAGCAGCTAGGAAGAAATGTAAAGAACGAGAGTTGTTGAAACTAGCATATTGGAAGATCAATCGGCCAAAATAACCATGAGCAGCTACAATATTATAAGTTTCTTCTTCTTGA-CCGAATC---TGTAACCTTCATTAGCAATAAATGCAAGAATATTTAC-TTCCATAATCTCATCGTTTTTTTACTTCAAAATAACT-CGGGATTTAATCCCAT----ATAATAAATCTTTCGCCTGTCAATTCA----ATGAATTACCTCTCGATGATC-TTGAAATCGGATCAATATCATGAATAACAATATCTGAGCTATCAAATCAATTCGTCGTCGAGAATTGAATAGTATAACATAGAAAGATCTTT-TATCCATACCG------AATCCAA------------------------AATTTCTTTATTTATCAATCATTCTTTTCTGTTCTTTCTTTATTTACAACCTATCTTAGGTCCTCCTTGTACAATCATCGGATAAAGTATCGTCTGACCGCCCGTCCGTTTCCATTAGTCACAAACGCCCAACAAACAATAGAAGCGAAGTGGAAAAAGAAATAAGTTACGTTCTAAACTCCG----TTTTTTTAATGATCTAGTTTTCTTGGAAGACAAAGAAGTGTGATAAAGAGGAGTTCCGGGATAAAGGATGGAATATTCCATCAAACTAACTATTTGAGTTTGGGTTTTGTTCGTTCTTCGACAGG--CCCT-------AAAAAAAAAATAGAAAAAAAAATAGGAAGGAAAAAT-GATTTATTCCCC-GCTACTTGTTAAGCTAAAAAAGGGGTGGGATCTTTGATTGATCTTTATTTTTCTTTTA-CCCCCCCTTCCTT---------------------------------------------------------------------------------------------------------------------------------------------------------------------------------------------------------------------------------------------------------------------------------------------------------------AACGCTCTCAATAATTGTACTATTCTACATATGTCTTTCTCCTACCAATCAGTATTATTTGAAATAATGAAAATTCCCCTATTTGTTTGATGAGAAGTGC----GAAATGCCAAAGGAAA--GAAAAAAGAACCCCCTT-GGGAATG-AAATTCTGCTCCCTGTGCCCCCTTTAACAGAAAAGGGAAGATTACAAATTGACTTATTATACTCCTGACTATGAAACCAAAGATACTGATATCTTGGCAGCATT-CCGAGTAACTCCTCAACCTGGAGTTCCACCTGAAGAAGCAGGGGCCGCGGTAGCTGCCGAATCTTCAACTGGTACATGGACAACTGTGTGGACCGATGGACTTACCAGCCTTGATCGTTACAAAGGGCGATGCTACCACATCGAGCCCGTTGCTGGAGAAGAAAATCAATATATTGCTTATGTAGCTTACCCATTAGACCTTTTTGAAGAAGGTTCTGTTACTAACATGTTTACTTCTATTGTGGGTAATGTATTTGGGTTCAAAGCCCTGCGCGCTCTACGTCTGGAAGATCTGCGAATCCCTRTCGCTTATRCTAAAACTTTCCAAGGCCCGCCTCATGGCATCCAAGTTGAGAGAGATAAATTGAACAAGTATGGTCGCCCCCTGTTGGGATGTACTATTAAACCTAAATTGGGGTTATCTGCTAAAAACTATGGTAGAGCGGTTTATGAATGTCTACGTGGTGGACTTGATTTTACCAAAGATGATGAGAA--CGT--GAACTCCCAACCATTTATGCGTT---GGAGAGATCGATTC

Abelia_grandiflora_BOP012208 CCTGAGCCAAATCCAGTTTTACGA----AAACAAGGGTTCAGAAAGCTAAAATC-AAAAAGGATAGGTGCAGAGACTCAATGGAAGCTGTTCTAACAAATGGAGTTGACTGTGTTGTGTTGGTAGAAAGAATCCTTCCATAGAAACTTCAGAAAGGATAAACGTATAAACATGGATATACGCATTGAAATACTAT-ATACTCTACCAAATGATTAATGACGACCCGAATCTGTATTTATA-----TATATATCAAAATGGGAGAATGGTTGTGAAGTGATTCCATATTGAAGAAAGAATCGAATATTTATTGATCAAATCATTCACTCCATAGTCTGATAGATC-TTTTGAAGAACTGATTAATTGGACGAGAATAAAGATAGAGTCCCATTCTACATGTCAATACTGGCAACAATGAAATTTATAGTAAAAGGAAAATCCGTCGACTTTAGAAATCGTGAGGGTTCAAGTCCCTCTATCCCCAAAAAACCCATATGGACTCCCTAATTATTTATCCTCTCCTT-TTATCC-TTTTTTGTTAGCGGTTAAAAATTCG------TTATCTTTCTCATTCACCCTACTCTTTTACAAAGAGATCTGAGCAGAAATGTTTTTCTCTTATC----ACAAGTCTTGTGATCTAAG----ATAA-TACGTGTACAAATGAACATCTTTGAGTAAGGAATCCCCATTTAAATGATTCATGGTCAA------TGTCATTATTCATACTGAAACTTACAAAGTCTTCCTT-TTGAAGATCCAAGAAATTCCAGGACCTGGATAAGACTTTGTAAGACCCTTTCAATTGACA-TAGACCCGAGTTATCTAGC--AAAATGAGGATGCATCGGTATTTATGTTAATGCACTTCCCAATGATACGTAAACAAGGCATTTCTGGTCCTTTA--------TAGAAAAGATCTATCATAGATATTTGTAATCTATCATTTATCGCTTGGGGGAGGAACAATAGTATTTCATTGCTACAAGTATGGATTATTGAGAATAATAAGACATGTATTTGGATATTTCCCTTGAACTCCGCAATCTTTTTGATTTGACATGGATAGTTGAAGGGAATTTTCCGAAGAAAAAATGGATTAGATTATGGGAGTGTGTGACTTGAACTATTGATTGGTCTGTGCAGATATATGCCTTTCGCTATCTGCCACATTGGAATTCACAACCAAATGTGTCTTTGTTCCAACCATTGCGTAAGCCC----------CATACAGAGGATAGGCTGGTTCACTTGAAGAGAATCTTTTCTATGATCAGATCCGAATCATGTCGTACATGAGCAGGTTCCGTAAGATCCAGTAG-----AATAAGTG-AACTAGATAACCCATAATCTCGATTATGGGTTATCTAG-TTCACTTACATACGATTGAATAGTATGGAAATGCATTCATTTCCTATGCATTGACACGATCTATGATACTATCGGAGTGAAACAGGGGATCTAAGGAAGAACAGAGGCTAGGCTATATTAGTAACAAGTAAACCC-----TTTGTGTGTCTGTCAAAAGTCTCCAAGTATTTTGGGGATAAACACCGATCCTAAGGTCTGAGACGACCCAGAAAGCATT--TGATCCTATCATGATCCACCTTGTAAGCCTACTTGGGTATTGAGTATTTACTTGTAAGAACCAAAATTTTTGCGCTGGATAGTTGTAACTCCGGAAAAAGAAATCC------------------AGTCAAATCTTTC------TTTTAAT--------AAAATCATTCATATTATCATA-----TATGTGTGGATCTAGATAACATATAGATTTTATATGGATTCCTTATGGTTCTTTTTCTTTTTGCTCGAGCCGTATGATGAAAAATTATCATGTCCGGTTCCTTCGGGGGATGGAT-----CTATAAAAATTCACCTATCCCAATAACAAAAAAACCTGACCTGAATGATCCTGTATTAAGAGCTAAATTGGCTAAAGGTATGGGTCATAATTATTACGGAGAGCCCGCATGGCCCAATGATCTTTTATATAT---------AGAGATTG-----------------------AAG-----AAACCAATGCAATATTTTTTAGGAAAGATAAAACTGGATGAATTCAAATTCGTTCAAATGGG-----------------------ACTGGAAGGTTCCTTATTTTCATTTAGGGTGTTCGTTTTATTTCTTCCCTTAGGACTTTGGTGTAGTTTATGCTCTCTCTCCTGGAATCG-AATTGTTGT-AACTGGACGCTTCTATCCT-CTAGCTAGTA--GGGATAGAAC-----AAAAAAAATATTTTCA------TTTTTTAATGAATTTTTTCTCATTTATCCGATTTATCAAATTTGAAACAAAAAGATACATTTTTTCAATGAACACA----------AAAAAATCCTAAAGTT---------ATACTATACAAAGGGTTGTCAAAATGGAATCAATTAGTTTCACCAATTCCTTCATTTTTATTTTTACTAAATTTTTATTTTTACTAATGATCTTATA----TATGCCC-------TTCTATAGATATATAGAG------------------ATAGAGAAACCC----------------------------ATTTTTCTTATTATATATTATA-----GATAAATAGGTTGATGGGGAAAATAAGACCCCGC-------CCTCGAAATGATAAAATCTACTAAAAAGAAAGGTAAAACCTTGTATCTTGTCTTTA-------------------TTTTCAAAAAAAAAAAATAATTTTTTTTTTGAATTTAGAAATTTAGTAAACAGAAGCATTTTTATTCTACTTCCATTCCCTATTGTTTTCGGCC-------AATGAATAGGGAATGGGAATTATTCATTTTATTTTTAGATTAACAATGAAATCAGAC---------AGTCAAATCAATTGGGATTATTCTAACGTTTTATGACTTATTTGCTTATTTGTTTGTCGTACAAAAAAACTTTTTACATTTCCGGTAGGTCGTACAAAAAAAC-TTTTTGAATTCCCGGT-ACAAAGGCCATCTCCCCCAATTGAACAAGAGAATAATGACTATGTTACATTACACATGAAGTAA-----GAAAAAAGTC--TTG-CTTTCTCTTTCTTTATGATATAG-------ATATGTACAACTTTGACCAGCAATTTCATTTAGATC-TAAGTAAGGGCTCGAAAGAT-CCAATAGACAAAT-------------ATAAAGAAAAATAAAGAAGACCC-----CTTTGATTTTGTTCCCTTTATTCCCACGGCCTGGCCTGGTCAATACCTAGCCGGGCC----TTTTTTTGTTCCAACAAATCCTAGCTAAAAGAATTTAGCTG-------------CTTTGAACACA---------------AAAATGCTTGCTATTAAAGCAGC-------------------AATAAAAAGATGAGGGGTTATTTCCATTCTTACTTATTATTCCAT------------------TCTTACTTATTATATA--TTATA---------------------------TATATATATAAATTATATAAAATCAAAGTATCCTTTCTTATT-----ATTCCTTCTTCCCTTTTGAGTTACTTGACGACCTTACGGGAAT--ATAAAATGAAACTG-----TGGGTTCTTA---AATAATAATGAATGCATTTTTCTGTTATGATTTCAGTGGTTTTAGTGAGCCATATCTATCAAAATCCCCCCAGCAAAAGAAAAAATAGAACTT--GTTATTTC----------------ATTTAGTTATTTAAAAGAGCCC-----TCCTTTCCGGAATCTCATTAAATCGAAATCCCCCGCGAACAAACGT-CGACACTCTCATTTTCATGA-----TTATGATC-CTA--T-CTTTATTACGCCTAATTCCTCGGTT-CGACAAAAAGTTCATTTGTATATAATAAGTATTAG-TATATCTAATACCTTACCCAGCCCATTTGGAAATTTTGGTTCAAACTCTTCGCTACTGGGTAAAAGATGCCCCCTCTTTGCATTTATTACGATTCTTTCTTCACGACTATCGGCATTGGACTAGTCTTTTTATTCCAAAGAAAGCCAGTTCTTTTTTTTCAAAACGAAAG------------------CAAAGATTATTCTTCTTCCTATATAATTCTCATGTATGTGAATACGAATCCATCTTCGTCTTTCTCCGTAACCAATCTTCTCATTTACAATCAACATCTTCTGGAGCCCTTCTTGAACGAATATATTTCTATGAAAAAATAGAACATCTTGTCGAAGTCTTTGCTAAA-GATTTTCAAGGCAATCTATGGTTGGTCAAGGATCCTTTCATGCATTATGTTAGGTATCAAGGAAAGTCAATTCTCGCTTCAA-GGAGAACTTTCTTTTGATGCGTAAATGGAAATATTACTTTGTACGTTTCTGGCAATGTCATTTTTACCAGTGGTTTCAACCAGGAAGGATCTATATAAACCAATTATCCAAACATTCCCTCGACCTTCTGGGCTATCTTTCAAGTGTGCGGCTAAATCCTTTAACGATACGCAGTCAAATGCTAGAAAATTCATTTCTAATTGATAATGCTGTTAAGAAGTTCGATACCATTGTTCCAATCATTCCTCTGATTGGATCATTGGTTAAAGCGAAATTTTGTAACGTATTAGGGCATCCTGTTAGTAAGGTAGTTTGGGCAGATTTATCAGATTTAAGACAT-AAAATTTACCCTACTTCTTTATCTTTC-TAGGAAGGGCCTTTCATGAGTTGAATAGAGATTTTCA-TTTTTTATTCATCATTCGGGTTGATGACCTAAA-CCAGATAGTTATATGAGTGAAAGAAACAGCTTATAAATTTGCAGTAAAAAGATTGAGTCTCATTTT-CTATGTAC-AAGAG-TTAAGTGAAAGTAACCATAAACATTA-GAAACGGTTTACCCCAAGATTGGTTAATTAGTGATCATGGCTTGAAGCGGGTGC-AAAAGATCAACTATATGGGG-TTTTTACTATCTATTACCATACATGTATTACCCTAACGGGCGATTAGCAAAAAGAGGTGGATAGTTAGGAACACCAAAGTACACAAAGGATTCGTAATAGAGATTATGTAAGTTATTCAACAGAATTTTTCTGTGCATAA-AAGGAATTCTGATTGGGACTTTAAGTTGGTAGAAATGATGAAGAAGTACTCCCCCTGATTCCGATCCAGAGTATACTCCTATCCACCGATTAAGTAAATAACTATCAAGAACGAAGTAATCCTTT-ACTTT------GTTTAAAGTCCCTTTTTCTGAGAAAGGAGAATAGGAACGAAAAAAATC-------AAATAG-AAAGA-ATAGAATTGCACTAG-----AAAGAAAGAGATCTTTTTTTATTCTTTCTTTCC-------------TCTATTTAGAAAGATAGAATTCTTGTCATCATTCGTGAACTAATGCGATGCCTAATTGTTTTTCGTAATCGAAAATGCTAGGTTG--------AAATATCTATGAATA-TTGCTACAAGAAAGA-TTTTATTGAAAA-TTTAAGTTATCACTCAAC-AAAGAAAAATAAAAA-TTATT----------ATAAGATCAA-TTCCG-AAGCGCTTTA-TTTTCAATATAGCAGACAGAATTCCATTGTCTAATTCGGG--ACTTTACAGTAGATTTTGATTCTATCTATCCTACGAATATAT-------------------CAAGATAAATAATAGCGAACTGGTCCTTAGA-TTTATTTGTGA-CCTTTGAGGAGCC-GTATGAGATGAAAATCTCATGTACGGTATTTAGATTA---------------------------------------------------------------------------------------------------------------------------------------------------------------------------------CTAAA------AATATTTAGTATTTTTCTTT-AA-----ATTAAAAAAAAA-ATATGTAAGTCAA--------------------------------------------------------------------------------TGTGAAAT-AAAAAAGGAGCAATAATCCCCTTCTTGTTCTATCAAG-AGGGCGATATTGCTCCTTTTTT-ATTTCTTTCAAATACTCGTATACACTAAGGCCGGGTCTTATCCATTTATAGATGGAGCTTCAAGAGCAGCTAGGTCTAGAGGGAAGTTATGAGCATTACGTTCATGCATAACTTCCATACCAAGGTTAGCGCGGTTAATGATATCAGCCCAAGTATTAATTACACGACCTTGACTATCAACTACAGATTGGTTGAAATTAAAACCATTTAGGTTGAAAGCCATAGTGCTGATACCTAAAGCAGTGAACCAGATACCTACTACAGGCCAAGCAGCTAGGAAGAAATGTAAAGAACGAGAGTTGTTGAAACTAGCATATTGGAAGATCAATCGGCCAAAATAACCATGAGCAGCTACAATATTATAAGTTTCTTCTTCTTGA-CCGAATC---TGTAACCTTCATTAGCAATAAATGCAAGAATATTTAC-TTCCATAATCTCATCGTTTTTTTACTTCAAAATAACT-CGGGATTTAATCCCAT----ATAATAAATCTTTCGCCTGTCAATTCA----ATGAATTACCTCTCGATGATC-TTGAAATCGGATCAATATCATGAATAACAATATCTGAGCTATCAAATCAATTCGTCGTCGAGAATTGAATAGTATAACATAGAAAGATCTTT-TATCCATACCG------AATCCAA------------------------AATTTCTTTATTTATCAATCATTCTTTTCTGTTCTTTCTTTATTTACAACCTATCTTAGGTCCTCCTTGTACAATCATCGGATAAAGTATCGTCTGACCGCCCGTCCGTTTCCATTAGTCACAAACGCCCAACAAACAATAGAAGCGAAGTGGAAAAAGAAATAAGTTACGTTCTAAACTCCG----TTTTTTTAATGATCTAGTTTTCTTGGAAGACAAAGAAGTGTGATAAAGAGGAGTTCCGGGATAAAGGATGGAATATTCCATCAAACTAACTATTTGAGTTTGGGTTTTGTTCGTTCTTCGACAGG--CCCT-------AAAAAAAAAATAGAAAAAAAAATAGGAAGGAAAAAT-GATTTATTCCCC-GCTACTTGTTAAGCTAAAAAAGGGGTGGGATCTTTGATTGATCTTTATTTTTCTTTTA-CCCCCCCTTCCTT---------------------------------------------------------------------------------------------------------------------------------------------------------------------------------------------------------------------------------------------------------------------------------------------------------------AACGCTCTCAATAATTGTACTATTCTACATATGTCTTTCTCCTACCAATCAGTATTATTTGAAATAATGAAAATTCCCCTATTTGTTTGATGAGAAGTGC----GAAATGCCAAAGGAAA--GAAAAAAGAACCCCCTT-GGGAATG-AAATTCTGCTCCCTGTGCCCCCTTTAACAGAAAAGGGAAGATTACAAATTGACTTATTATACTCCTGACTATGAAACCAAAGATACTGATATCTTGGCAGCATT-CCGAGTAACTCCTCAACCTGGAGTTCCACCTGAAGAAGCAGGGGCCGCGGTAGCTGCCGAATCTTCAACTGGTACATGGACAACTGTGTGGACCGATGGACTTACCAGTCTTGATCGTTACAAAGGGCGATGCTACCACATCGAGCCCGTTGCTGGAGAAGAAAATCAATATATTGCTTATGTAGCTTACCCATTAGACCTTTTTGAAGAAGGTTCTGTTACAAACATGTTTACTTCTATTGTGGGTAATGTATTTGGGTTCAAAGCCCTGCGCGCTCTACGTCTGGAAGATCTGCGAATCCCTGTCGCTTATACTAAAACTTTCCAAGGCCCGCCTCATGGCATCCAAGTTGAGAGAGATAAATTGAACAAGTATGGTCGCCCCCTGTTGGGATGTACTATTAAACCTAAATTGGGGTTATCTGCTAAAAACTATGGTAGAGCGGTTTATGAATGTCTACGTGGTGGACTTGATTTTACCAAAGATGATGAGAA--CGT--GAACTCCCAACCATTTATGCGTT---GGAGAGATCGATTC

Abelia_grandiflora_BOP012290 CCTGAGCCAAATCCAGTTTTACGA----AAACAAGGGTTCAGAAAGCTAAAATC-AAAAAGGATAGGTGCAGAGACTCAATGGAAGCTGTTCTAACAAATGGAGTTGACTGTGTTGTGTTGGTAGAAAGAATCCTTCCATAGAAACTTCAGAAAGGATAAACGTATAAACATGGATATACGCATTGAAATACTAT-ATACTCTACCAAATGATTAATGACGACCCGAATCTGTATTTATA-----TATATATCAAAATGGGAGAATGGTTGTGAAGTGATTCCATATTGAAGAAAGAATCGAATATTTATTGATCAAATCATTCACTCCATAGTCTGATAGATC-TTTTGAAGAACTGATTAATTGGACGAGAATAAAGATAGAGTCCCATTCTACATGTCAATACTGGCAACAATGAAATTTATAGTAAAAGGAAAATCCGTCGACTTTAGAAATCGTGAGGGTTCAAGTCCCTCTATCCCCAAAAAACCCATATGGACTCCCTAATTATTTATCCTCTCCTT-TTATCC-TTTTTTGTTAGCGGTTAAAAATTCG------TTATCTTTCTCATTCACCCTACTCTTTTACAAAGAGATCTGAGCAGAAATGTTTTTCTCTTATC----ACAAGTCTTGTGATCTAAG----ATAA-TACGTGTACAAATGAACATCTTTGAGTAAGGAATCCCCATTTAAATGATTCATGGTCAA------TGTCATTATTCATACTGAAACTTACAAAGTCTTCCTT-TTGAAGATCCAAGAAATTCCAGGACCTGGATAAGACTTTGTAAGACCCTTTCAATTGACA-TAGACCCGAGTTATCTAGC--AAAATGAGGATGCA-CGGTATTTATGTTAATGCACTTCCCAATGATACGTAAACAAGGCATTTCTGGTCCTTTA--------TAGAAAAGATCTATCATAGATATTTGTAATCTATCATTTATCGCTTGGGGGAGGAACAATAGTATTTCATTGCTACAAGTATGGATTATTGAGAATAATAAGACATGTATTTGGATATTTCCCTTGAACTCCGCAATCTTTTTGATTTGACATGGATAGTTGAAGGGAATTTTCCGAAGAAAAAATGGATTAGATTATGGGAGTGTGTGACTTGAACTATTGATTGGTCTGTGCAGATATATGCCTTTCGCTATCTGCCACATTGGAATTCACAACCAAATGTGTCTTTGTTCCAACCATTGCGTAAGCCC----------CATACAGAGGATAGGCTGGTTCACTTGAAGAGAATCTTTTCTATGATCAGATCCGAATCATGTCGTACATGAGCAGGTTCCGTAAGATCCAGTAG-----AATAAGTG-AACTAGATAACCCATAATCTCGATTATGGGTTATCTAG-TTCACTTACATACGATTGAATAGTATGGAAATGCATTCATTTCCTATGCATTGACACGATCTATGATACTATCGGAGTGAAACAGGGGATCTAAGGAAGAACAGAGGCTAGGCTATATTAGTAACAAGTAAACCC-----TTTGTGTGTCTGTCAAAAGTCTCCAAGTATTTTGGGGATAAACACCGATCCTAAGGTCTGAGACGACCCAGAAAGCATT--TGATCCTATCATGATCCACCTTGTAAGCCTACTTGGGTATTGAGTATTTACTTGTAAGAACCAAAATTTTTGCGCTGGATAGTTGTAACTCCGGAAAAAGAAATCC------------------AGTCAAATCTTTC------TTTTAAT--------AAAATCATTCATATTATCATA-----TATGTGTGGATCTAGATAACATATAGATTTTATATGGATTCCTTATGGTTCTTTTTCTTTTTGCTCGAGCCGTATGATGAAAAATTATCATGTCCGGTTCCTTCGGGGGATGGAT-----CTATAAAAATTCACCTATCCCAATAACAAAAAAACCTGACCTGAATGATCCTGTATTAAGAGCTAAATTGGCTAAAGGTATGGGTCATAATTATTACGGAGAGCCCGCATGGCCCAATGATCTTTTATATATTTTTCCAGTAGAGATTG-----------------------AAG-----AAACCAATGCAATATTTTTTAGGAAAGATAAAACTGGATGAATTCAAATTCGTTCAAATGGG-----------------------ACTGGAAGGTTCCTTATTTTCATTTAGGGTGTTCGTTTTATTTCTTCCCTTAGGACTTTGGTGTAGTTTATGCTCTCTCTCCTGGAATCG-AATTGTTGT-AACTGGACGCTTCTATCCT-CTAGCTAGTA--GGGATAGAAC-----AAAAAAAATATTTTCA------TTTTTTAATGAATTTTTTCTCATTTATCCGATTTATCAAATTTGAAACAAAAAGATACATTTTTTCAATGAACACA----------AAAAAATCCTAAAGTT---------ATACTATACAAAGGGTTGTCAAAATGGAATCAATTAGTTTCACCAATTCCTTCATTTTTATTTTTACTAAATTTTTATTTTTACTAATGATCTTATA----TATGCCC-------TTCTATAGATATATAGAG------------------ATAGAGAAACCC----------------------------ATTTTTCTTATTATATATTATA-----GATAAATAGGTTGATGGGGAAAATAAGACCCCGC-------CCTCGAAATGATAAAATCTACTAAAAAGAAAGGTAAAACCTTGTATCTTGTCTTTA-------------------TTTTCAAAAAAAAAAAATAATTTTTTTTTTGAATTTAGAAATTTAGTAAACAGAAGCATTTTTATTCTACTTCCATTCCCTATTGTTTTCGGCC-------AATGAATAGGGAATGGGAATTATTCATTTTATTTTTAGATTAACAATGAAATCAGAC---------AGTCAAATCAATTGGGATTATTCTAACGTTTTATGACTTATTTGCTTATTTGTTTGTCGTACAAAAAAACTTTTTACATTTCCGGTAGGTCGTACAAAAAAAC-TTTTTGAATTCCCGGT-ACAAAGGCCATCTCCCCCAATTGAACAAGAGAATAATGACTATGTTACATTACACATGAAGTAA-----GAAAAAAGTC--TTG-CTTTCTCTTTCTTTATGATATAG-------ATATGTACAACTTTGACCAGCAATTTCATTTAGATC-TAAGTAAGGGCTCGAAAGAT-CCAATAGACAAAT-------------ATAAAGAAAAATAAAGAAGACCC-----CTTTGATTTTGTTCCCTTTATTCCCACGGCCTGGCCTGGTCAATACCTAGCCGGGCC----TTTTTTTGTTCCAACAAATCCTAGCTAAAAGAATTTAGCTG-------------CTTTGAACACA---------------AAAATGCTTGCTATTAAAGCAGC-------------------AATAAAAAGATGAGGGGTTATTTCCATTCTTACTTATTATTCCAT------------------TCTTACTTATTATATACTTATTATA-------------------------TATATATATAAATTATATAAAATCAAAGTATCCTTTCTTATT-----ATTCCTTCTTCCCTTTTGAGTTACTTGACGACCTTACGGGAAT--ATAAAATGAAACTG-----TGGGTTCTTA---AATAATAATGAATGCATTTTTCTGTTATGATTTCAGTGGTTTTAGTGAGCCATATCTATCAAAATCCCCCCAGCAAAAGAAAAAATAGAACTT--GTTATTTC----------------ATTTAGTTATTTAAAAGAGCCC-----TCCTTTCCGGAATCTCATTAAATCGAAATCCCCCGCGAA-AAACGT-CGACACTCTCATTTTCATGA-----TTATGATC-CTA--T-CTTTATTACGCCTAATTCCTCGGTT-CGACAAAAAGTTCATTTGTATATAATAAGTATTAG-TATATCTAATACCTTACCCAGCCCATTTGGAAATTTTGGTTCAAACTCTTCGCTACTGGGTAAAAGATGCCCCCTCTTTGCATTTATTACGATTCTTTCTTCACGACTATCGGCATTGGACTAGTCTTTTTATTCCAAAGAAAGCCAGTTCTTTTTTTTCAAAACGAAAG------------------CAAAGATTATTCTTCTTCCTATATAATTCTCATGTATGTGAATACGAATCCATCTTCGTCTTTCTCCGTAACCAATCTTCTCATTTACAATCAACATCTTCTGGAGCCCTTCTTGAACGAATATATTTCTATGAAAAAATAGAACATCTTGTCGAAGTCTTTGCTAAA-GATTTTCAAGGCAATCTATGGTTGGTCAAGGATCCTTTCATGCATTATGTTAGGTATCAAGGAAAGTCAATTCTCGCTTCAAAGGAGAACTTTCTTTTGATGCGTAAATGGAAATATTACTTTGTACGTTTCTGGCAATGTCATTTTTACCAGTGGTTTCAACCAGGAAGGATCTATATAAACCAATTATCCAAACATTCCCTCGACCTTCTGGGCTATCTTTCAAGTGTGCGGCTAAATCCTTTAACGATACGCAGTCAAATGCTAGAAAATTCATTTCTAATTGATAATGCTGTTAAGAAGTTCGATACCATTGTTCCAATCATTCCTCTGATTGGATCATTGGTTAAAGCGAAATTTTGTAACGTATTAGGGCATCCTGTTAGTAAGGTAGTTTGGGCAGATTTATCAGATTTAAGACAT-AAAATTTACCCTACTTCTTTATCTTTC-TAGGAAGGGCCTTTCATGAGTTGAATAGAGATTTTCA-TTTTTTATTCATCATTCGGGTTGATGACCTAAA-CCAGATAGTTATATGAGTGAAAGAAACAGCTTATAAATTTGCAGTAAAAAGATTGAGTCTCATTTT-CTATGTAC-AAGAG-TTAAGTGAAAGTAACCATAAACATTA-GAAACGGTTTACCCCAAGATTGGTTAATTAGTGATCATGGCTTGAAGCGGGTGC-AAAAGATCAACTATATGGGG-TTTTTACTATCTATTACCATACATGTATTACCCTAACGGGCGATTAGCAAAAAGAGGTGGATAGTTAGGAACACCAAAGTACACAAAGGATTCGTAATAGAGATTATGTAAGTTATTCAACAGAATTTTTCTGTGCATAA-AAGGAATTCTGATTGGGACTTTAAGTTGGTAGAAATGATGAAGAAGTACTCCCCCTGATTCCGATCCA-AGTATACTCCTATCCACCGATWAAGTAAATAACTATCAAGAACGAASTAATCCTTT-ACTTT------GTTTAAAGTCCCTTTTTCTGAGAAAGGAGAATAGGAACGAAAAAAATC-------AAATAG-AAAGA-ATAGAATTGCACTAG-----AAAGAAAGAGATCTTTTTTTATTCTTTCTTTCC-------------TCTATTTAGAAAGATAGAATTCTTGTCATCATTCGTGAACTAATGCGATGCCTAATTGTTTTTCGTAATCGAAAATGCTAGGTTG--------AAATATCTATGAATA-TTGCTACAAGAAAGA-TTTTATTGAAAA-TTTAAGTTATCACTCAAC-AAAGAAAAATAAAAA-TTATT----------ATAAGATCAA-TTCCG-AAGCGCTTTA-TTTTCAATATAGCAGACAGAATTCCATTGTCTAATTCGGG--ACTTTACAGTAGATTTTGATTCTATCTATCCTACGAATATAT-------------------CAAGATAAATAATAGCGAACTGGTCCTTAGA-TTTATTTGTGA-CCTTTGAGGAGCC-GTATGAGATGAAAATCTCATGTACGGTATTTAGATTA---------------------------------------------------------------------------------------------------------------------------------------------------------------------------------CTAAA------AATATTTAGTATTTTTCTTT-AA-----ATTAAAAAAAAA-ATATGTAAGTCAA--------------------------------------------------------------------------------TGTGAAAT-AAAAAAGGAGCAATAATCCCCTTCTTGTTCTATCAAG-AGGGCGATATTGCTCCTTTTTT-ATTTCTTTCAAATACTCGTATACACTAAGGCCGGGTCTTATCCATTTATAGATGGAGCTTCAAGAGCAGCTAGGTCTAGAGGGAAGTTATGAGCATTACGTTCATGCATAACTTCCATACCAAGGTTAGCGCGGTTAATGATATCAGCCCAAGTATTAATTACACGACCTTGACTATCAACTACAGATTGGTTGAAATTAAAACCATTTAGGTTGAAAGCCATAGTGCTGATACCTAAAGCAGTGAACCAGATACCTACTACAGGCCAAGCAGCTAGGAAGAAATGTAAAGAACGAGAGTTGTTGAAACTAGCATATTGGAAGATCAATCGGCCAAAATAACCATGAGCAGCTACAATATTATAAGTTTCTTCTTCTTGA-CCGAATC---TGTAACCTTCATTAGCAATAAATGCAAGAATATTTAC-TTCCATAATCTCATCGTTTTTTTACTTCAAAATAACT-CGGGATTTAATCCCAT----ATAATAAATCTTTCGCCTGTCAATTCA----ATGAATTACCTCTCGATGATC-TTGAAATCGGATCAATATCATGAATAACAATATCTGAGCTATCAAATCAATTCGTCGTCGAGAATTGAATAGTATAACATAGAAAGATCTTT-TATCCATACCG------AATCCAA------------------------AATTTCTTTATTTATCAATCATTCTTTTCTGTTCTTTCTTTATTTACAACCTATCTTAGGTCCTCCTTGTACAATCATCGGATAAAGTATCGTCTGACCGCCCGTCCGTTTCCATTAGTCACAAACGCCCAACAAACAATAGAAGCGAAGTGGAAAAAGAAATAAGTTACGTTCTAAACTCCG----TTTTTTTAATGATCTAGTTTTCTTGGAAGACAAAGAAGTGTGATAAAGAGGAGTTCCGGGATAAAGGATGGAATATTCCATCAAACTAACTATTTGAGTTTGGGTTTTGTTCGTTCTTCGACAGG--CCCT-------AAAAAAAAAATAGAAAAAAAAATAGGAAGGAAAAAT-GATTTATTCCCC-GCTACTTGTTAAGCTAAAAAAGGGGTGGGATCTTTGATTGATCTTTATTTTTCTTTTA-CCCCCCCTTCCTT---------------------------------------------------------------------------------------------------------------------------------------------------------------------------------------------------------------------------------------------------------------------------------------------------------------AACGCTCTCAATAATTGTACTATTCTACATATGTCTTTCTCCTACCAATCAGTATTATTTGAAATAATGAAAATTCCCCTATTTGTTTGATGAGAAGTGC----GAAATGCCAAAGGAAA--GAAAAAAGAACCCCCTT-GGGAATG-AAATTCTGCTCCCTGTGCCCCCTTTAACAGAAAAGGG--------------------------------------------------------TTGGCAGCATT-CCGAGTAACTCCTCAACCTGGAGTTCCACCTGAAGAAGCAGGGGCCGCGGTAGCTGCCGAATCTTCAACTGGTACATGGACAACTGTGTGGACCGATGGACTTACCAGTCTTGATCGTTACAAAGGGCGATGCTACCACATCGAGCCCGTTGCTGGAGAAGAAAATCAATATATTGCTTATGTAGCTTACCCATTAGACCTTTTTGAAGAAGGTTCTGTTACAAACATGTTTACTTCTATTGTGGGTAATGTATTTGGGTTCAAAGCCCTGCGCGCTCTACGTCTGGAAGATCTGCGAATCCCTGTCGCTTATACTAAAACTTTCCAAGGCCCGCCTCATGGCATCCAAGTTGAGAGAGATAAATTGAACAAGTATGGTCGCCCCCTGTTGGGATGTACTATTAAACCTAAATTGGGGTTATCTGCTAAAAACTATGGTAGAGCGGTTTATGAATGTCTACGTGGTGGACTTGATTTTACCAAAGATGATGAGAA--CGT--GAACTCCCAACCATTTATGCGTT---GGAGAGATCGTTTC

Abelia_macrotera_BOP012250 CCTGAGCCAAATCCAGTTTTACGA----AAACAAGGGTTCAGAAAGCTAAAATC-AAAAAGGATAGGTGCAGAGACTCAATGGAAGCTGTTCTAACAAATGGAGTTGACTGTGTTGTGTTGGTAGAAAGAATCCTTCCATAGAAACTTCAGAAAGGATAAACGTATAAACATGGATATACGCATTGAAATACTAT-ATACTCTACCAAATGATTAATGACGACCCGAATCTGTATT--TA-----TATATATCAAAATGGGAGAATGGTTGTGAAGTGATTCCATATTGAAGAAAGAATCGAATATTTATTGATCAAATCATTCACTCCATAGTCTGATAGATC-TTTTGAAGAACTGATTAATTGGACGAGAATAAAGATAGAGTCCCATTCTACATGTCAATACCGGCAACAATGAAATTTATAGTAAAAGGAAAATCCGTCGACTTTAGAAATCGTGAGGGTTCAAGTCCCTCTATCCCCAAAAAACCCATATGGACTCCCTAATTATTTATCCTCTCCTT-TTATCC-TTTTTTGTTAGCGGTTAAAAATTCG------TTATCTTTCTCATTCACCCTACTCTTTTACAAAGAGATCTGAGCGGAAATGTTTTTCTCTTATC----ACAAGTCTTGTGATCTAAG----ATAA-TACGTGTACAAATGAACATCTTTGAGTAAGGAATCCCCATTTAAATGATTCATGGTCAA------TGTCATTATTCATACTGAAACTTACAAAGTCTTCCTT-TTGAAGATCCAAGAAATTCCAGGACCTGGATAAGACTTTGTAAGACCCTTTCAATTGACA-TAGACCCGAGTTATCTAGC--AAAATGAGGATGCAGCGGTATTTATGTTAATGCACTTCCCAATGATACGTAAACAAGGCATTTCTGGTCCTTTA--------TAGAGAAGATCTATCATAGATATTTGTAATCTATCATTTATCGCTTGGGGGAGGAACAATAGTATTTCATTGCTACAAGTATGGATTATTGCGAATAATAAGACATGTATTTGGATATTTCCCTTGAACTCCGCAATCTTTTTGATTTGACATGGATAGTTGAAGGGAATTTTCCGAAGAAAAAATGGATTAGATTATGGGAGTGTGTGACTTGAACTATTGATTGGTCTGTGCAGATATATGCCTTTCGCTATCTGCCACATTGGAATTCACAACCAAATGTGTCTTTGTTCCAACCATTGCGTAAGCCC----------CATACAGAGGATAGGCTGGTTCACTTGAAGAGAATCTTTTCTATGATCAGATCCGAATCATGTCGTACATGAGCAGGTTCCGTAAGATCCAGTAG-----AATAAGTG-AACTAGATAACCCATAATCTCGATTATGGGTTATCTAG-TTCACTTCCATACGATTGAATAGTATGGAAATGCATTCATTTCCTATGCATTGACACGATCTATGATACTATCGGAGTGAAACAGGGGATCTAAGGAAGAACAGAGGCTAGGCTATATTAGTAACAAGTAAACCC-----TTTGTGTGTCTGTCAAAAGTCTCCAAGTATTTTGGGGATAAACACCGATCCTAAGGTCTGAGACGACCCAGAAAGCATT--TGATCCTATCATGATCCACCTTGTAAGCCTACTTGGGTATTGAGTATTTACTTGTAAGAACCAAAATTTTTGCGCTGGATAGTTGCAACTCCGGAAAAAGAAATCC------------------AGTCAAATTTTTC------TTTTAAT--------AAAATCATTCATATTATCATA-----TATGTGTGGATCTAGATAACATATAGATTTTATATGGATTCCTTATGGTTCTTTTTCTTTTTGCTCGAGCCGTATGATAAAAAATTATCATGTCCGGTTCCTTCGGGGGATGGAT-----CTATAAAAATTCACCTATCCCAATAACAAAAAAACCTGACCTGAATGATCCTGTATTAAGAGCTAAATTGGCTAAAGGTATGGGTCATAATTATTACGGAGAGCCCGCATGGCCCAATGATCTTTTATATATTTTTCCAGTAGAGATTG-----------------------AAG-----AAATCAATGCAATATTTTTTAGGAAAGATAAAACTGGATGAATTCAAATTCGTTCAAATGGG-----------------------ATTGGAAGGTTCCTTATTTTCATTTAGGGTGTTCGTTTTATTTCTTCCCTTAGGACTTTGGTGTAGTTTATGCTCTCTCTTCTGGAATCG-AATTGTTGT-AACTGGACGCTTCTATCCT-CTAGCTAGTA--GGGATAGAAC-----AAAAAAAATATTTTCA------TTTTTTAATGAATTTTTTCTCATTTATCCGATTTATCAAATTTGAAACAAAAAGATACATTTTTTCAATGAACACAAAAAAATCCTAAAAAATCCTAAAGTT---------ATACTATACAAAGGGTTGTCAAAATGGAATCAATTAGTTTCACCAATTCCTTC-----------------------ATTTTTACTAATGATCTTATA----TATGCCC-------TTCTATAGATATATATAGAG----------------ATAGAGAAACCC----------------------------ATTTTTCT-------TATTATA-----GATAAATAGGTTGATGGGGAAAATAAGACCCCGC-------CCTCGAAATGATAAAATCTACTAAAAAGAAAGGTAAAACCTTGTATCTTGTCTTTA--------------------TTTTCAAAAAAA--------TTCTTTTTTTTATTTAGAAATTTAGTAAACAGAAGCATTTTTATTCTACTTCCATTCCCTATTGTTTTCGGCC-------AATGAATAGGGAATGGGAATTATTCATTTTATTTTTAGATTAACAATGAAATCAGAC---------AGTCAAATCAATTGGGATTATTCTAACGTTTTATGA--------CTTATTTGTTTGTCGTACAAAAAAACTTTTTACATTTCCGGTAGGTCGTACAAAAAGAC-TTTTTGAATTCCCGGT-ACAAAGGCCATCTCCCCCAATTGAACAAGAGAATAATGACTATGTTACATTACACATGAAGTAA-----GAAAAAAGTC--TTG-CTTTCTCTTTCTTTATGATATAG-------ATATGTACAACTTTGACCAGCAATTTCATTTAGATC-TAAGTAAGGGCTCGAAAGAT-CCAATAGACAAAT-------------ATAAAGAAAAATAAAGAAGACCC-----CTTTAATTTTGTTCCCTTTATTCCCACGGCCTGGCCTGGTCAATACCTAGCCGGGCC----TTTTTTTGTTCCAACAAATCCTAGCTAAAAGAATTTAGCTG-------------CTTTGAACACA---------------AAAATGCTTGCTATTAAAGCAGC-------------------AATAAAAAGATGAGGGGTTATTTCCATTCTTACTTAT--------------------------------------TATA--TATTATTTATATTA------------------AATATATATAAATTATATAAAATCAAAGTATCCTTTCTTATT-----ATTCCTTCTTCCCTTTTGAGTTACTTGACGACCTTACGGGAAT--ATAAAATGAAACTG-----TGGGTTCTTA---AATAATAATGAATGCATTTTTCTGTTATGATTTCAGTGGTTTTAGTGAGCCATATCTATCAAAATCCCCCCAGCAAAAGAAAAAATAGAACTT--GTTATTTC----------------ATTTAGTTATTTAAAAGAGCCC-----TCCTTTCCGGAATCTCATTAAATCGAAATCCCCCGCGAA-AAACGT-CGACACTCTCATTTTCATGA-----TTATGATC-CTA--T-CTTTATTACGCCTAATTCCTCGGTT-CGACAAAAAGTTCATTTGTATATAATAAGTATTAG-TATATCTAATACCTTACCCAGCCCATTTGGAAATTTTGGTTCAAACTCTTCGCTACTGGGTAAAAGATGCCCCCTCTTTGCATTTATTACGATTCTTTCTTCACGACTATCGGCATTGGACTAGTCTTTTTATTCCAAAGAAAGCCAGTTCTTTTTTTTCAAAACGAAAG------------------CAAAGATTATTCTTCTTCCTATATAATTCTCATGTATGTGAATACGAATCCATCTTCGTCTTTCTCCGTAACCAATCTTCTCATTTACAATCAACATCTTCTGGAGCCCTTCTTGAACGAATATATTTCTATGAAAAAATAGAACATCTTGTCGAAGTCTTTGCTAAA-GATTTTCAAGGCAATCTATGGTTGTTCAAGGATCCTTTCATGCATTATGTTAGGTATCAAGGAAAGTCAATTCTCGCTTCAAAGGAGAACTTTCTTTTGATGCGTAAATGGAAATATTACTTTGTACGTTTCTGGCAATGTCA-TTTTACCAGTGGTTTCAACCAGGAAGGATCTATATAAACCAATTATCCAAACATTCCCTCGACCTTCTGGGCTATCTTTCAAGTGTGCGGCTAAATCCTTTAACGATACGCAGTCAAATGCTAGAAAATTCATTTCTAATTGATAATGCTGTTAAGAAGTTCGATACCATTGTTCCAATTATTCCTCTGATTGGATCATTGGTTAAAGCGAAATTTTGTAACGTATTAGGGCATCCTGTTAGTAAGGTAGTTTGGGCAGATTTATCAGATTTAAGACAT-AAAATTTACCCTACTTCTTTATCTTTC-TAGGAAGGGCCTTTCATGAGTTGAATAGAGATTTTCA-TTTTTTATTCATCATTCGGGTTGATGAACTAAA-CCAGATAGTTATATGAGTGAAAGAAACAGCTTATAAATTTGCAGTAAAAAGATTGAGTCTCATTTT-CTATGTACAAAGAG-TTAAGTGAAAGTAACCATAAACATTA-GAAACGGTTTACCCCAAGATTGGTCAATTAGTGATCATGGCTTGAAGCGGGTGC-AAAAGATCAACTATATGGGG--TTTTACTATCTATTACCATACATGTATTACCCTAACGGGCGATTAGCAAAAAGAGGTGGATAGTTAGGAACACCAAAGTACACAAAGGATTCGTAATAGAGATTATGTAAGTTATTCAACAGAATTTTTCTGTGCATAA-AAGGAATTCTGATTGGGACTTTAAGTTGGTAGAAATGATGAAGAAGTACTCCCCCTGATTCCGATCCAGAGTATACTCCTATCCACCGATTAAGTAAATAACTATCAAGAACGAAGTAATCCTTT-ACTTT------GTTTAAAGTCCCTTTTTCTGAGAAAGGAGAATAGGAACGAAAAAAATC-------AAATAG-AAAGA-ATAGAATTGCACTAGAAAGAAAAGAAAGAGATCTTTTTTTATTCTTTCTTTCC-------------TCTATTTAGAAAGATAGAATTCTTGTCATCATTCGTGAACTAATGCGATGCCTAATTGTTTTTCGTAATCGAAAATGCTAGGTTG--------AAATATCTATGAATA-TTGCTACAAGAAAGA-TTTTATTGAAAA-TTTAAGTTATCACTCAAC-AAAGAAAAATAAAAA-TTATT-----AAAAGATAAGATCAA-TTCCG-AAGCGCTTTA-TTTTCAATATAGCAGACAGAATTCCATTGTCTAATTCGGG--ACTTTACAGTAGATTTTGATTCTATCTATCCTACGAATATAT-------------------CAAGATAAATAATAGCGAACTGGTCCTTAGA-TTTATTTGTGA-CCTTTGAGGAGCC-GTATGAGATGAAAATCTCATGTACGGTATTTAGATTA---------------------------------------------------------------------------------------------------------------------------------------------------------------------------------CTAAA------AATATTTACGATTTTTCTTT-AA-----ATAAAAAAAAAATATATGTAAGTCAA--------------------------------------------------------------------------------TGTGAAAT-AAAAAAGGAGCAATAATCCCCTTCTTGTTCTATCAAG-AGGGCGATATTGCTCCTTTTTT-ATTTCTTTCAAATACTCGTATACACTAAGGCCAGGTCTTATCCATTTATAGATGGAGCTTCAAGAGCAGCTAGGTCTAGAGGGAAGTTATGAGCATTACGTTCATGCATAACTTCCATACCAAGGTTAGCACGGTTAATGATATCAGCCCAAGTATTAATTACACGACCTTGACTATCAACTACAGATTGGTTGAAATTAAAACCATTTAGGTTGAAAGCCATAGTGCTGATACCTAAAGCAGTGAACCARATACCTACTACAGGCCAAGCAGCTAGGAAGAAATGTAAAGAACGAGAGTTGTTGAAACTAGCATATTGGAAGATCAATCGGCCAAAATAACCATGAGCAGCTACAATATTATAAGTTTCTTCTTCTTGA-CCGAATC---TGTAACCTTCATTAGCAATAAATGCAAGAATATTTAC-TTCCATAATCTCATCGTTTTTTTACTTCAAAATAACT-CGGGATTTAATCCCATAGAGATAATAAATCTTTCGCCTGTCAATTCA----ATGAATTACCTCTCGATGATC-TTGAAATCGGATCAATATCATGAATAACAATATCTGAGCTATCAAATCAATTCGTCGTCGAGAATTGAATAGTATAACATAGAAAGATCTTT-TATCCATACCG------AATCCAA------------------------AATTTCTTTATTTATCAATCATTCTTTTCTGTTCTTTCTTTATTTACAACCTATCTTAGGTCCTCCTTGTACAATCATCGGATAAAGTATCGTCTGACCGCCCGTCCGTTTCCATTAGTCACAAACGCCCAACAAACAATAGAAGCGAAGTGGAAAAAGAAATAAGTTACGTTCTAAACTCCG----TTTTTTTAATGATCTAGTTTTCTTGGAAGACAAAGAAGTGTGATAAAGAGGAGTTCCGGGATAAAGGATGTAATATTCCATCAAACTAACTATTTGAGTTTGGGTTTTGTTCGTTYTTCGACGGG--CCCT------AAAAAAAAAAATAG--AAAAAAATAGGAAGGAAAAAT-GATTTATTCCCCCGCTACTTGTTAAGCTAAAAAAGGGGTGGGATCTTTGATTGATCTTTATTTTTCTTTTA-CCCCCCCTTCCTT---------------------------------------------------------------------------------------------------------------------------------------------------------------------------------------------------------------------------------------------------------------------------------------------------------------AACGCTCTCAATAATTGTACTATTCTACATATGTCTTTCTCCTACCAATCAGTATTATTTGAAATAATGAAAATTCCCCTATTTGTTTGATGAGAAGTGC----GAAATGCCAAAGGAAA--GAAAAAAGAACCCCCTT-GGGAATG-AAATTCTGCTCCCCGTGCCCCCTTTAACAGAAAAGGGAAGATTACAAATTGACTTATTATACTCCTGACTATGAAACCAAAGATACTGATATCTTGGCAGCATT-CCGAGTAACTCCTCAACCTGGAGTTCCACCTGAAGAAGCAGGGGCCGCGGTAGCTGCCGAATCTTCAACTGGTACATGGACAACTGTGTGGACCGATGGACTTACCAGCCTTGATCGTTACAAAGGGCGATGCTACCACATCGAGCCCGTTGCTGGAGAAGAAACTCAATTTATTGCTTATGTAGCTTACCCATTAGACCTTTTTGAAGAAGGTTCTGTTACTAACATGTTTACTTCTATTGTGGGTAATGTATTTGGGTTCAAAGCCCTGCGCGCTCTACGTCTGGAAGATCTGCGAATCCCTGTCGCTTATACTAAAACTTTCCAAGGTCCGCCTCATGGCATCCAAGTTGAGAGAGATAAATTGAACAAGTATGGTCGCCCCCTGTTGGGATGTACTATTAAACCTAAATTGGGGTTATCTGCTAAAAACTATGGTAGAGCGGTTTATGAATGTCTACGTGGTGGACTTGATTTTACCAAAGATGATGAGAA--CGT--GAACTCCCAACCATTTATGCGTT---GGAGAGATCGATTC

Abelia_macrotera_BOP012263 CCTGAGCCAAATCCAGTTTTACGA----AAACAAGGGTTCAGAAAGCTAAAATC-AAAAAGGATAGGTGCAGAGACTCAATGGAAGCTGTTCTAACAAATGGAGTTGACTGTGTTGTGTTGGTAGAAAGAATCCTTCCATAGAAACTTCAGAAAGGATAAACGTATAAACATGGATATACGCATTGAAATACTAT-ATACTCTACCAAATGATTAATGACGACCCGAATCTGTATT--TA-----TATATATCAAAATGGGAGAATGGTTGTGAAGTGATTCCATATTGAAGAAAGAATCGAATATTTATTGATCAAATCATTCACTCCATAGTCTGATAGATC-TTTTGAAGAACTGATTAATTGGACGAGAATGAAGATAGAGTCCCATTCTACATGTCAATACCGGCAACAATGAAATTTATAGTAAAAGGAAAATCCGTCGACTTTAGAAATCGTGAGGGTTCAAGTCCCTCTATCCCCAAAAAACCCATATGGACTCCCTAATTATTTATCCTCTCCTT-TTATCC-TTTTTTGTTAGCGGTTAAAAATTCG------CTATCTTTCTCATTCACCCTACTCTTTTACAAAGAGATCTGAGCGGAAATGTTTTTCTCTTATC----ACAAGTCTTGTGATCTAAG----ATAA-TACGTGTACAAATGAACATCTTTGAGTAAGGAATCCCCATTTAAATGATTCATGGTCAA------TGTCATTATTCATACTGAAACTTACAAAGTCTTCCTT-TTGAAGATCCAAGAAATTCCAGGACCTGGATAAGACTTTGTAAGACCCTTTCAATTGACA-TAGACCCGAGTTATCTAGC--AAAATGAGGATGCAGCGGTATTTATGTTAATGCACTTCCCAATGATACGTAAACAAGGCATTTCTGGTCCTTTA--------TAGAGAAGATCTATCATAGATATTTGTAATCTATCATTTATCGCTTGGGGGAGGAACAATAGTATTTCATTGCTACAAGTATGGATTATTGCGAATAATAAGACATGTATTTGGATATTTCCCTTGAACTCCGCAATCTTTTTGATTTGACATGGATAGTTGAAGGGAATTTTCCGAAGAAAAAATGGATTAGATTATGGGAGTGTGTGACTTGAACTATTGATTGGTCTGTGCAGATATATGCCTTTCGCTATCTGCCACATTGGAATTCACAACCAAATGTGTCTTTGTTCCAACCATTGCGTAAGCCC----------CATACAGAGGATAGGCTGGTTCACTTGAAGAGAATCTTTTCTATGATCAGATCCGAATCATGTCGTACATGAGCAGGTTCCGTAAGATCCAGTAG-----AATAAGTG-AACTAGATAACCCATAATCTCGATTATGGGTTATCTAG-TTCACTTCCATACGATTGAATAGTATGGAAATGCATTCATTTCCTATGCATTGACACGATCTATGATACTATCGGAGTGAAACAGGGGATCTAAGGAAGAACAGAGGCTAGGCTATATTAGTAACAAGTAAACCC-----TTTGTGTGTCTGTCAAAAGTCTCCAAGTATTTTGGGGATAAACACCGATCCTAAGGTCTGAGACGACCCAGAAAGCATT--TGATCCTATCATGATCCACCTTGTAAGCCTACTTGGGTATTGAGTATTTACTTGTAAGAACCAAAATTTTTGCGCTGGATAGTTGCAACTCCGGAAAAAGAAATCC------------------AGTCAAATTTTTC------TTTTAAT--------AAAATCATTCATATTATCATA-----TATGTGTGGATCTAGATAACATATAGATTTTATATGGATTCCTTATGGTTCTTTTTCTTTTTGCTCGAGCCGTATGATAAAAAATTATCATGTCCGGTTCCTTCGGGGGATGGAT-----CTATAAAAATTCACCTATCCCAATAACAAAAAAACCTGACCTGAATGATCCTGTATTAAGAGCTAAATTGGCTAAAGGTATGGGTCATAATTATTACGGAGAGCCCGCATGGCCCAATGATCTTTTATATATTTTTCCAGTAGAGATTG-----------------------AAG-----AAATCAATGCAATATTTTTTAGGAAAGATAAAACTGGATGAATTCAAATTCGTTCAAATGGG-----------------------ATTGGAAGGTTCCTTATTTTCATTTAGGGTGTTCGTTTTATTTCTTCCCTTAGGACTTTGGTGTAGTTTATGCTCTCTCTTCTGGAATCG-AATTGTTGT-AACTGGACGCTTCTATCCT-CTAGCTAGTA--GGGATAGAAC-----AAAAAAAATATTTTCA------TTTTTTAATGAATTTTTTCTCATTTATCCGATTTATCAAATTTGAAACAAAAAGATACATTTTTTCAATGAACACAAAAAAATCCTAAAAAATCCTAAAGTT---------ATACTATACAAAGGGTTGTCAAAATGGAATCAATTAGTTTCACCAATTCCTTC-----------------------ATTTTTACTAATGATCTTATA----TATGCCC-------TTCTATAGATATATATAGAG----------------ATAGAGAAACCC----------------------------ATTTTTCT-------TATTATA-----GATAAATAGGTTGATGGGGAAAATAAGACCCCGC-------CCTCGAAATGATAAAATCTACTAAAAAGAAAGGTAAAACCTTGTATCTTGTCTTTA--------------------TTTTCAAAAAAA--------TTCTTTTTTTTATTTAGAAATTTAGTAAACAGAAGCATTTTTATTCTACTTCCATTCCCTATTGTTTTCGGCC-------AATGAATAGGGAATGGGAATTATTCATTTTATTTTTAGATTAACAATGAAATCAGAC---------AGTCAAATCAATTGGGATTATTCTAACGTTTTATGA--------CTTATTTGTTTGTCGTACAAAAAAACTTTTTACATTTCCGGTAGGTCGTACAAAAAGAC-TTTTTGAATTCCCGGT-ACAAAGGCCATCTCCCCCAATTGAACAAGAGAATAATGACTATGTTACATTACACATGAAGTAA-----GAAAAAAGTC--TTG-CTTTCTCTTTCTTTATGATATAG-------ATATGTACAACTTTGACCAGCAATTTCATTTAGATC-TAAGTAAGGGCTCGAAAGAT-CCAATAGACAAAT-------------ATAAAGAAAAATAAAGAAGACCC-----CTTTAATTTTGTTCCCTTTATTCCCACGGCCTGGCCTGGTCAATACCTAGCCGGGCC----TTTTTTTGTTCCAACAAATCCTAGCTAAAAGAATTTAGCTG-------------CTTTGAACACA---------------AAAATGCTTGCTATTAAAGCAGC-------------------AATAAAAAGATGAGGGGTTATTTCCATTCTTACTTAT--------------------------------------TATA--TATTATTTATATTA------------------AATATATATAAATTATATAAAATCAAAGTATCCTTTCTTATT-----ATTCCTTCTTCCCTTTTGAGTTACTTGACGACCTTACGGGAAT--ATAAAATGAAACTG-----TGGGTTCTTA---AATAATAATGAATGCATTTTTCTGTTATGATTTCAGTGGTTTTAGTGAGCCATATCTATCAAAATCCCCCCAGCAAAAGAAAAAATAGAACTT--GTTATTTC----------------ATTTAGTTATTTAAAAGAGCCC-----TCCTTTCCGGAATCTCATTAAATCGAAATCCCCCGCGAA-AAACGT-CGACACTCTCATTTTCATGA-----TTATGATC-CTA--T-CTTTATTACGCCTAATTCCTCGGTT-CGACAAAAAGTTCATTTGTATATAATAAGTATTAG-TATATCTAATACCTTACCCAGCCCATTTGGAAATTTTGGTTCAAACTCTTCGCTACTGGGTAAAAGATGCCCCCTCTTTGCATTTATTACGATTCTTTCTTCACGACTATCGGCATTGGACTAGTCTTTTTATTCCAAAGAAAGCCAGTTCTTTTTTTTCAAAACGAAAG------------------CAAAGATTATTCTTCTTCCTATATAATTCTCATGTATGTGAATACGAATCCATCTTCGTCTTTCTCCGTAACCAATCTTCTCATTTACAATCAACATCTTCTGGAGCCCTTCTTGAACGAATATATTTCTATGAAAAAATAGAACATCTTGTCGAAGTCTTTGCTAAA-GATTTTCAAGGCAATCTATGGTTGTTCAAGGATCCTTTCATGCATTATGTTAGGTATCAAGGAAAGTCAATTCTCGCTTCAAAGGAGAACTTTCTTTTGATGCGTAAATGGAAATATTACTTTGTACGTTTCTGGCAATGTCATTTTTACCAGTGGTTTCAACCAGGAAGGATCTATATAAACCAATTATCCAAACATTCCCTCGACCTTCTGGGCTATCTTTCAAGTGTGCGGCTAAATCCTTTAACGATACGCAGTCAAATGCTAGAAAATTCATTTCTAATTGATAATGCTGTTAAGAAGTTCGATACCATTGTTCCAATTATTCCTCTGATTGGATCATTGGTTAAAGCGAAATTTTGTAACGTATTAGGGCATCCTGTTAGTAAGGTAGTTTGGGCAGATTTATCAGATTTAAGACAT-AAAATTTACCCTACTTCTTTATCTTTC-TAGGAAGGGCCTTTCATGAGTTGAATAGAGATTTTCA-TTTTTTATTCATCATTCGGGTTGATGAACTAAA-CCAGATAGTTATATGAGTGAAAGAAACAGCTTATAAATTTGCAGTAAAAAGATTGAGTCTCATTTT-CTATGTACAAAGAG-TTAAGTGAAAGTAACCATAAACATTA-GAAACGGTTTACCCCAAGATTGGTCAATTAGTGATCATGGCTTGAAGCGGGTGC-AAAAGATCAACTATATGGGG--TTTTACTATCTATTACCATACATGTATTACCCTAACGGGCGATTAGCAAAAAGAGGTGGATAGTTAGGAACACCAAAGTACACAAAGGATTCGTAATAGAGATTATGTAAGTTATTCAACAGAATTTTTCTGTGCATAA-AAGGAATTCTGATTGGGACTTTAAGTTGGTAGAAATGATGAAGAAGTACTCCCCCTGATTCCGATCCAGAGTATACTCCTATCCACCGATTAAGTAAATAACTATCAAGAACGAAGTAATCCTTT-ACTTT------GTTTAAAGTCCCTTTTTCTGAGAAAGGAGAATAGGAACGAAAAAAATC-------AAATAG-AAAGA-ATAGAATTGCACTAGAAAGAAAAGAAAGAGATCTTTTTTTATTCTTTCTTTCC-------------TCTATTTAGAAAGATAGAATTCTTGTCATCATTCGTGAACTAATGCGATGCCTAATTGTTTTTCGTAATCGAAAATGCTAGGTTG--------AAATATCTATGAATA-TTGCTACAAGAAAGA-TTTTATTGAAAA-TTTAAGTTATCACTCAAC-AAAGAAAAATAAAAA-TTATT-----AAAAGATAAGATCAA-TTCCG-AAGCGCTTTA-TTTTCAATATAGCAGACAGAATTCCATTGTCTAATTCGGG--ACTTTACAGTAGATTTTGATTCTATCTATCCTACGAATATAT-------------------CAAGATAAATAATAGCGAACTGGTCCTTAGA-TTTATTTGTGA-CCTTTGAGGAGCC-GTATGAGATGAAAATCTCATGTACGGTATTTAGATTA---------------------------------------------------------------------------------------------------------------------------------------------------------------------------------CTAAA------AATATTTACGATTTTTCTTT-AA-----ATAAAAAAAAAATATATGTAAGTCAA--------------------------------------------------------------------------------TGTGAAAT-AAAAAAGGAGCAATAATCCCCTTCTTGTTCTATCAAG-AGGGCGATATTGCTCCTTTTTT-ATTTCTTTCAAATACTCGTATACACTAAGGCCAGGTCTTATCCATTTATAGATGGAGCTTCAAGAGCAGCTAGGTCTAGAGGGAAGTTATGAGCATTACGTTCATGCATAACTTCCATACCAAGGTTAGCACGGTTAATGATATCAGCCCAAGTATTAATTACACGACCTTGACTATCAACTACAGATTGGTTGAAATTAAAACCATTTAGGTTGAAAGCCATAGTGCTGATACCTAAAGCAGTGAACCARATACCTACTACAGGCCAAGCAGCTAGGAAGAAATGTAAAGAACGAGAGTTGTTGAAACTAGCATATTGGAAGATCAATCGGCCAAAATAACCATGAGCAGCTACAATATTATAAGTTTCTTCTTCTTGA-CCGAATC---TGTAACCTTCATTAGCAATAAATGCAAGAATATTTAC-TTCCATAATCTCATCGTTTTTTTACTTCAAAATAACT-CGGGATTTAATCCCATAGAGATAATAAATCTTTCGCCTGTCAATTCA----ATGAATTACCTCTCGATGATC-TTGAAATCGGATCAATATCATGAATAACAATATCTGAGCTATCAAATCAATTCGTCGTCGAGAATTGAATAGTATAACATAGAAAGATCTTT-TATCCATACCG------AATCCAA------------------------AATTTCTTTATTTATCAATCATTCTTTTCTGTTCTTTCTTTATTTACAACCTATCTTAGGTCCTCCTTGTACAATCATCGGATAAAGTATCGTCTGACCGCCCGTCCGTTTCCATTAGTCACAAACGCCCAACAAACAATAGAAGCGAAGTGGAAAAAGAAATAAGTTACGTTCTAAACTCCG----TTTTTTTAATGATCTAGTTTTCTTGGAAGACAAAGAAGTGTGATAAAGAGGAGTTCCGGGATAAAGGATGTAATATTCCATCAAACTAACTATTTGAGTTTGGGTTTTGTTCGTTYTTCGACGGG--CCCT------AAAAAAAAAAATAG--AAAAAAATAGGAAGGAAAAAT-GATTTATTCCCCCGCTACTTGTTAAGCTAAAAAAGGGGTGGGATCTTTGATTGATCTTTATTTTTCTTTTA-CCCCCCCTTCCTT---------------------------------------------------------------------------------------------------------------------------------------------------------------------------------------------------------------------------------------------------------------------------------------------------------------AACGCTCTCAATAATTGTACTATTCTACATATGTCTTTCTCCTACCAATCAGTATTATTTGAAATAATGAAAATTCCCCTATTTGTTTGATGAGAAGTGC----GAAATGCCAAAGGAAA--GAAAAAAGAACCCCCTT-GGGAATG-AAATTCTGCTCCCCGTGCCCCCTTTAACAGAAAAGGGAAGATTACAAATTGACTTATTATACTCCTGACTATGAAACCAAAGATACTGATATCTTGGCAGCATT-CCGAGTAACTCCTCAACCTGGAGTTCCACCTGAAGAAGCAGGGGCCGCGGTAGCTGCCGAATCTTCAACTGGTACATGGACAACTGTGTGGACCGATGGACTTACCAGCCTTGATCGTTACAAAGGGCGATGCTACCACATCGAGCCCGTTGCTGGAGAAGAAACTCAATTTATTGCTTATGTAGCTTACCCATTAGACCTTTTTGAAGAAGGTTCTGTTACTAACATGTTTACTTCTATTGTGGGTAATGTATTTGGGTTCAAAGCCCTGCGCGCTCTACGTCTGGAAGATCTGCGAATCCCTGTCGCTTATACTAAAACTTTCCAAGGTCCGCCTCATGGCATCCAAGTTGAGAGAGATAAATTGAACAAGTATGGTCGCCCCCTGTTGGGATGTACTATTAAACCTAAATTGGGGTTATCTGCTAAAAACTATGGTAGAGCGGTTTATGAATGTCTACGTGGTGGACTTGATTTTACCAAAGATGATGAGAA--CGT--GAACTCCCAACCATTTATGCGTT---GGAGAGATCGATTC

Abelia_myrtilloides_BOP012264 CCTGAGCCAAATCCAGTTTTACGA----AAACAAGGGTTCAGAAAGCTAAAATC-AAAAAGGATAGGTGCAGAGACTCAATGGAAGCTGTTCTAACAAATGGAGTTGACTGTGTTGTGTTGGTAGAAAGAATCCTTCCATAGAAACTTCAGAAAGGATAAACGTATAAACATGGATATACGCATTGAAATACTAT-ATACTCTACCAAATGATTAATGACAACCCGAATCTGTATT--TA-----TATATATCAAAATGGGAGAATGGTTGTGAAGTGATTCCATATTGAAGAAAGAATCGAATATTTATTGATCAAATCATTCACTCCATAGTCTGATAGATC-TTTTGAAGAACTGATTAATTGGACGAGAATAAAGATAGAGTCCCATTCTACATGTCAATACCGGCAACAATGAAATTTATAGTAAAAGGAAAATCCGTCGACTTTAGAAATCGTGAGGGTTCAAGTCCCTCTATCCCCAAAAAACCCATATGGACTCCCTAATTATTTATCCTCTCCTT-TTATCC-TTTTTTGTTAGCGGTTAAAAATTCG------TTATCTTTCTCATTCACCCTACTCTTTTACAAAGAGATCTGAGCGGAAATGTTTTTCTCTTATC----ACAAGTCTTGTGATCTAAG----ATAA-TACGTGTACAAATGAACATCTTTGAGTAAGGAATCCCCATTTAAATGATTCATGGTCAA------TGTCATTATTCATACTGAAACTTACAAAGTCTTCCTT-TTGAAGATCCAAGAAATTCCAGGACCTGGATAAGACTTTGTAAGACCCTTTCAATTGACA-TAGACCCGAGTTATCTAGC--AAAATGAGGATGCAGCGGTATTTATGTTAATGCACTTCCCAATGATACGTAAACAAGGCATTTCTGGTCCTTTA--------TAGAGAAGATCTATCATAGATATTTGTAATCTATCATTTATCGCTTGGGGGAGGAACAATAGTATTTCATTGCTACAAGTATGGATTATTGAGAATAATAAGACATGTATTTGGATATTTCCCTTGAACTCCGCAATCTTTTTGATTTGACATGGATAGTTGAAGGGAATTTTCCGAAGAAAAAATGGATTAGATTATGGGAGTGTGTGACTTGAACTATTGATTGGTCTGTGCAGATATATGCCTTTCGCTATCTGCCACATTGGAATTCACAACCAAATGTGTCTTTGTTCCAACCATTGCGTAAGCCC----------CATACAGAGGATAGGCTGGTTCACTTGAAGAGAATCTTTTCTATGATCAGATCCGAATCATGTCGTACATGAGCAGGTTCCGTAAGATCCAGTAG-----AATAAGTG-AACTAGATAACCCATAATCGAGATTATGGGTTATCTAG-TTCACTTACATACGATTGAATAGTATGGAAATGCATTCATTTCCTATGCATTGACACGATCTATGATACTATCGGAGTGAAACAGGGGATCTAAGGAAGAACAGAGGCTAGGCTATATTAGTAACAAGTAAACCC-----TTTGTGTGTCTGTCAAAAGTCTCCAAGTATTTTGGGGATAAACACCGATCCTAAGGTCTGAGACGACCCAGAAAGCATT--TGATCCTATCATGATCCACCTTGTAAGCCTACTTGGGTATTGAGTATTTACTTGTAAGAACCAAAATTTTTGCGCTGGATAGTTGCAACTCCGGAAAAAGAAATCC------------------AGTCAAATTTTTC------TTTTAAT--------TAAATCATTCATATTATCATA-----TATGTGTGGATCTAGATAACATATAGATTTTATATGGATTCCTTATGGTTCTTTTTCTTTTTGCTCGAGCCGTATGATAAAAAATTATCATGTCCGGTTCCTTCGGGGGATGGAT-----CTATAAAAATTCACCTATCCCAATAACAAAAAAACCTGACCTGAATGATCCTGTATTAAGAGCTAAATTGGCTAAAGGTATGGGTCATAATTATTACGGAGAGCCCGCATGGCCCAATGATCTTTTATATATTTTTCCAGTAGAGATTG-----------------------AAG-----AAATCAATGCAATATTTTTTAGGAAAGATAAAACTGGATGAATTCAAATTCGTTCAAATGGG-----------------------ATTGGAAGGTTCCTTATTTTCATTTAGGGTGTTCGTTTTATTTCTTCCCTTAGGACTTTGGTGTAGTTTATGCTCTCTCTTCTGGAATCG-AATTGTTGT-AACTGGACGCTTCTATCCT-CTAGCTAGTA--GGGATAGAAC-----AAAAAAAATATTTTCA------TTTTTTAATGAATTTTTTCTCATTTATCCGATTTATCAAATTTGAAACAAAAAGATACATTTTTTCAATGAACACA----------AAAAAATCCTAAAGTT---------ATACTATACAAAGGGTTGTCAAAATGGAATCAATTAGTTTCACCAATTCCTTC-----------------------ATTTTTACTAATGATCTTATA----TATGCCC-------TTCTATAGATATATATAGAG----------------ATAGAGAAACCC----------------------------ATTTTTCT-------TATTATA-----GATAAATAGGTTGATGGGGAAAATAAGACCCCGC-------CCTCGAAATGATAAAATCTACTAAAAAGAAAGGTAAAACCTTGTATCTTGTCTTTA--------------------TTTTCAAAAAAA--------TTCTTTTTTTTATTTAGAAATTTAGTAAACAGAAGCATTTTTATTCTACTTCCATTCCCTATTGTTTTCGGCC-------AATGAATAGGGAATGGGAATTATTCATTTTATTTTTAGATTAACAATGAAATCAGAC---------AGTCAAATCAATTGGGATTATTCTAACGTTTTATGA--------CTTATTTGTTTGTCGTACAAAAAAACTTTTTACATTTCCGGTAGGTCGTACAAAAAGAC-TTTTTGAATTCCCGGT-ACAAAGGCCATCTCCCCCAATTGAACAAGAGAATAATGACTATGTTACATTACACATGAAGTAA-----GAAAAAAGTC--TTG-CTTTCTCTTTCTTTATGATATAG-------ATATGTACAACTTTGACCAGCAATTTCATTTAGATC-TAAGTAAGGGCTCGAAAGAT-CCAATAGACAAAT-------------ATAAAGAAAAATAAAGAAGACCC-----CTTTAATTTTGTTCCCTTTATTCCCACGGCCTGGCCTGGTCAATACCTAGCCGGGCC----TTTTTTTGTTCCAACAAATCCTAGCTAAAAGAATTTAGCTG-------------CTTTGAACACA---------------AAAATGCTTGCTATTAAAGCAGC-------------------AATAAAAAGATGAGGGGTTATTTCCATTCTTACTTAT--------------------------------------TATA--TATTATTTATATTA------------------AATATATATAAATTATATAAAATCAAAGTATCCTTTCTTATT-----ATTCCTTCTTCCCTTTTGAGTTACTTGACGACCTTACGGGAAT--ATAAAATGAAACTG-----TGGGTTCTTA---AATAATAATGAATGCATTTTTCTGTTATGATTTCAGTGGTTTTAGTGAGCCATATCTATCAAAATCCCCCCAGCAAAAGAAAAAATAGAACTT--GTTATTTC----------------ATTTAGTTATTTAAAAGAGCCC-----TCCTTTCCGGAATCTCATTAAATCGAAATCCCCCGCGAA-AAACGT-CGACACTCTCATTTTCATGA-----TTATGATC-CTA--T-CTTTATTACGCCTAATTCCTCGGTT-CGACAAAAAGTTCATTTGTATATAATAA???????????????????????????????????????????????????????????????????????????????????????????????????????????????????????????????????????????????????????????????????????????????????????????????????????????????????????????????????????????????????????????????????????????????????????????????????????????????????????????????????????????????????????????????????????????????????????????????????????????????????????????????????????????????????????????????????????????????????????????????????????????????????????????????????????????????????????????????????????????????????????????????????????????????????????????????????????????????????????????????????????????????????????????????????????????????????????????????????????????????????????????????????????????????????????????????????????????TTAAGACAT-AAAATTTACCCTACTTCTTTATCTTTC-TAGGAAGGGCCTTTCATGAGTTGAATAGAGATTTTCA-TTTTTTATTCATCATTCGGGTTGATGAACTAAA-CCAGATAGTTATATGAGTGAAAGAAACAGCTTATAAATTTGCAGTAAAAAGATTGAGTCTCATTTT-CTATGTAC-AAGAG-TTAAGTGAAAGTAACCATAAACATTA-GAAACGGTTTACCCCAAGATTGGTCAATTAGTGATCATGGCTTGAAGCGGGTGC-AAAAGATCAACTATATGGGG--TTTTACTATCTATTACCATACATGTATTACCCTAACGGGCGATTAGCAAAAAGAGGTGGATAGTTAGGAACACCAAAGTACACAAAGGATTCGTAATAGAGATTATGTAAGTTATTCAACAGAATTTTTCTGTGCATAA-AAGGAATTCTGATTGGGACTTTAAGTTGGTAGAAATGATGAAGAAGTACTCCCCCTGATTCCGATCCAGAGTATACTCCTATCCACCGATTAAGTAAATAACTATCAAGAACGAAGTAATCCTTT-ACTTT------GTTTAAAGTCCCTTTTTCTGAGAAAGGAGAATAGGAACGAAAAAAATC-------AAATAG-AAAGA-ATAGAATTGCACTAGAAAGAAAAGAAAGAGATCTTTTTTTATTCTTTCTTTCC-------------TCTATTTAGAAAGATAGAATTCTTGTCATCATTCGTGAACTAATGCGATGCCTAATTGTTTTTCGTAATCGAAAATGCTAGGTTG--------AAATATCTATGAATA-TTGCTACAAGAAAGA-TTTTATTGAAAA-TTTAAGTTATCACTCAAC-AAAGAAAAATAAAAA-TTATT-----AAAAGATAAGATCAA-TTCCG-AAGCGCTTTA-TTTTCAATATAGCAGACAGAATTCCATTGTCTAATTCGGG--ACTTTACAGTAGATTTTGATTCTATCTATCCTACGAATATAT-------------------CAAGATAAATAATAGCGAACTGGTCCTTAGA-TTTATTTGTGA-CCTTTGAGGAGCC-GTATGAGATGAAAATCTCATGTACGGTATTTAGATTA---------------------------------------------------------------------------------------------------------------------------------------------------------------------------------CTAAA------AATATTTACGATTTTTCTTT-AA-----ATAAAAAAAA---ATATGTAAGTCAA--------------------------------------------------------------------------------TGTGAAAT-AAAAAAGGAGCAATAATCCCCTTCTTGTTCTATCAAG-GGGGCGATATTGCTCCTTTTTT-ATTTCTTTCAAATACTCGTATACACTAAGGCCAGGTCTTATCCATTTATAGATGGAGCTTCAAGAGCAGCTAGGTCTAGAGGGAAGTTATGAGCATTACGTTCATGCATAACTTCCATACCAAGGTTAGCGCGGTTAATGATATCAGCCCAAGTATTAATTACACGACCTTGACTATCAACTACAGATTGGTTGAAATTAAAACCATTTAGGTTGAAAGCCATAGTGCTGATACCTAAAGCAGTGAACCAGATACCTACTACAGGCCAAGCAGCTAGGAAGAAATGTAAAGAACGAGAGTTGTTGAAACTAGCATATTGGAAGATCAATCGGCCAAAATAACCATGAGCAGCTACAATATTATAAGTTTCTTCTTCTTGA-CCGAATC---TGTAACCTTCAT???????????????????????????????????????????????????????????????????????????????????????????????????????????????????????????????????????????????????????????????????????????????????????????????????????????????????????????????????????????????????????????????????????????????????????????????????????????????????????????????????????????????????????????????????????????????????????????????????????????????????????????????????????????????????????????????????????????????????????????????????????????????????????????????????????????????????????????????????????????????????????????????????????????????????????????????????????????????????????????????????????????????????????????????????????????????????????????????????????????????????????????????????????????????????????????????????????????????????????????????????????????????????????????????????????????????????????????????????????????????????????????????????????????????????????????????????????????????????????????????????????????????????????????????????????????????????????????????????????????????????????????????????????????????????????????????????????????????????????????????????????????????????????????????????????????????????????????????????????????????????????????????????????????????????????????????????????????????????????????????????????????????????????????????????????????????????????????????????????????????????????????????????????????????????????????????????????????????????????????????????????????????????????????????????????????????????????????????????????????????????????????????????????????????????????????????????????????????????????????????????????????????????????????????????????????????????????????????????????????????????????????????????????????????????????????????????????????????????????????????????????????????????????????????????????????????????????????????

Abelia_parvifolia_BOP012265 CCTGAGCCAAATCCAGTTTTACGA----AAACAAGGGTTCAGAAAGCTAAAATC-AAAAAGGATAGGTGCAGAGACTCAATGGAAGCTGTTCTAACAAATGGAGTTGACTGTGTTGTGTTGGTAGAAAGAATCCTTCCATAGAAACTTCAGAAAGGATAAACGTATAAACATGGATATACGCATTGAAATACTAT-ATACTCTACCAAATGATTAATGACGACCCGAATCTGTATT--TA-----TATATATCAAAATGGGAGAATGGTTGTGAAGTGATTCCATATTGAAGAAAGAATCGAATATTTATTGATCAAATCATTCACTCCATAGTCTGATAGATC-TTTTGAAGAACTGATTAATTGGACGAGAATAAAGATAGAGTCCCATTCTACATGTCAATACCGGCAACAATGAAATTTATAGTAAAAGGAAAATCCGTCGACTTTAGAAATCGTGAGGGTTCAAGTCCCTCTATCCCCAAAAAACCCATATGGACTCCCTAATTATTTATCCTCTCCTT-TTATCC-TTTTTTGTTAGCGGTTAAAAATTCG------TTATCTTTCTCATTCGCCCTACTCTTTTACAAAGAGATCTGAGCGGAAATGTTTTTCTCTTATC----ACAAGTCTTGTGATCTAAG----ATAA-TACGTGTACAAATGAACATCTTTGAGTAAGGAATCCCCATTTAAATGATTCATGGTCAA------TGTCATTATTCATACTGAAACTTACAAAGTCTTCCTT-TTGAAGATCCAAGAAATTCCAGGACCTGGATAAGACTTTGTAAGACCCTTTCAATTGACA-TAGACCCGAGTTATCTAGC--AAAATGAGGATGCAGCGGTATTTATGTTAATGCACTTCCCAATGATACGTAAACAAGGCATTTCTGGTCCTTTA--------TAGAGAAGATCTATCATAGATATTTGTAATCTATCATTTATCGCTTGGGGGAGGAACAATAGTATTTCATTGCTACAAGTATGGATTATTGAGAATAATAAGACATGTATTTGGATATTTCCCTTGAACTCCGCAATCTTTTTGATTTGACATGGATAGTTGAAGGGAATTTTCCGAAGAAAAAATGGATTAGATTATGGGAGTGTGTGACTTGAACTATTGATTGGTCTGTGCAGATATATGCCTTTCGCTATCTGCCACATTGGAATTCACAACCAAATGTGTCTTTGTTCCAACCATTGCGTAAGCCC----------CATACAGAGGATAGGCTGGTTCACTTGAAGAGAATCTTTTCTATGATCAGATCCGAATCATGTCGTATATGAGCAGGTTCCGTAAGATCCAGTAG-----AATAAGTG-AACTAGATAACCCATAATCGAGATTATGGGTTATCTAG-TTCACTTACATACGATTGAATAGTATGGAAATGCATTCATTTCCTATGCATTGACACGATCTATGATACTATCGGAGTGAAACAGGGGATCTAAGGAAGAACAGAGGCTAGGCTATATTAGTAACAAGTAAACCC-----TTTGTGTGTCTGTCAAAAGTCTCCAAGTATTTTGGGGATAAACACCGATCCTAAGGTCTGAGACGACCCAGAAAGCATT--TGATCCTATCATGATCCACCTTGTAAGCCTACTTGGGTATTGAGTATTTACTTGTAAGAACCAAAATTTTTGCGCTGGATAGTTGCAACTCCGGAAAAAGAAATCC------------------AGTCAAATTTTTC------TTTTAAT--------AAAATCATTCATATTATCATA-----TATGTGTGGATCTAGATAACATATAGATTTTATATGGATTCCTTATGGTTCTTTTTCTTTTTGCTCGAGCCGTATGATGAAAAATTATCATGTCCGGTTCCTTCGGGGGATGGAT-----CTATAAAAATTCACCTATCCCAATAACAAAAAAACCTGACCTGAATGATCCTGTATTAAGAGCTAAATTGGCGAAAGGTATGGGTCATAATTATTACGGAGAGCCCGCATGGCCCAATGATCTTTTATATATTTTTCCAGTAGAGATTG-----------------------AAG-----AAATCAATGCAATATTTTTTAGGAAAGATAAAACTGGATGAATTCAAATTCGTTCAAATGGG-----------------------ATTGGAAGGTTCCTTATTTTCATTTAGGGTGTTCGTTTTATTTCTTCCCTTAGGACTTTGGTGTAGTTTATGCTCTCTCT-TTGGAATCGAAATTGTTGTAAACTGGACGCTTCTATCCT-CTAGCTAGTA--GGGATAGAAC-----AAAAAAAATATTTTCA------TTTTTTAATGAATTTTTTCTCATTTATCCGATTTATCAAATTTGAAACAAAAAGATACATTTTTTCAATGAACACAAAAAAATCCTAAAAAATCCTAAAGTT---------ATACTATACAAAGGGTTGTCAAAATGGAATCAATTAGTTTCACCAATTCCTTC-----------------------ATTTTTACTAATGATCTTATA----TATGCCC-------TTCTATAGATATATATAGAG----------------ATAGAGAAACCC----------------------------ATTTTTCT-------TATTATA-----GATAAATAGGTTGGTGGGGAAAATAAGACCCCGC-------CCTCGAAATGATAAAATCTACTAAAAAGAAAGGTAAAACCTTGTATCTTGTCTTTA--------------------TTTTCAAAAAAA--------TTCTTTTTTTTATTTAGAAATTTAGTAAACAGAAGCATTTTTATTCTACTTCCATTCCCTATTGTTTTCGGCC-------AATGAATAGGGAATGGGAATTATTCATTTTATTTTTAGATTAACAATGAAATCAGAC---------AGTCAAATCAATTGGGATTATTCTAACGTTTTATGA--------CTTATTTGTTTGTCGTACAAAAAAACTTTTTACATTTCCGGTAGGTCGTACAAAAAGACTTTTTTGAATTCCCGGT-ACAAAGGCCATCTCCCCCAATTGAACAAGAGAATAATGACTATGTTACATTACACATGAAGTAA-----GAAAAAAGTC--TTG-CTTTCTCTTTCTTTATGATATAG-------ATATGTACAACTTTGACCAGCAATTTCATTTAGATC-TAAGTAAGGGCTCGAAAGAT-CCAATAGACAAAT-------------ATAAAGAAAAATAAAGAAGACCC-----CTTTGATTTTGTTCCCTTTATTCCCACGGCCTGGCCTGGTCAATACCTAGCCGGGCC----TTTTTTTGTTCCAACAAATCCTAGCTAAAAGAATTTAGCTG-------------CTTTGAACACA---------------AAAATGCTTGCTATTAAAGCAGC-------------------AATAAAAAGATGAGGGGTTATTTCCATTCTTACTTAT--------------------------------------TATA--TATTATTTATATAT------------------TATATGTAAAAATTATATAAAATCAAAGTATCCTTTCTTATT-----ATTCCTTCTTCCCTTTTGAGTTACTTGACGACCTTACGGGAAT--ATAAAATGAAACTG-----TGGGTTCTTA---AATAATAATGAATGCATTTTTCTGTTATGATTTCAGTGGTTTTAGTGAGCCATATCTATCAAAATCCCCCCAGCAAAAGAAAAAATAGAACTT--GTTATTTC----------------ATTTAGTTATTTAAAAGAGCCC-----TCCTTTCCGGAATCTCATTAAATCGAAATCCCCCGCGAA-AAACGT-CGACACTCTCATTTTCATGA-----TTATGATC-CTA--T-CTTTATTACGCCTAATTCCTCGGTT-CGACAAAAAGTTCATTTGTATATAATAAGTATTAG-TATATCTAATACCTTACCCAGCCCATTTGGAAATTTTGGTTCAAACTCTTCGCTACTGGGTAAAAGATGCCCCCTCTTTGCATTTATTACGATTCTTTCTTCACGACTATCGGCATTGGACTAGTCTTTTTATTCCAAAGAAAGCCAGTTCTTTTTTTTCAAAACGAAAG------------------CAAAGATTATTCTTCTTCCTATATAATTCTCATGTATGTGAATACGAATCCATCTTCGCCTTTCTCCGTAACCAATCTTCTCATTTACAATCAACATCTTCTGGAGCCCTTCTTGAACGAATATATTTCTATGAAAAAATAGAACATCTTGTCGAAGTCTTTGCTAAA-GATTTTCAAGGCAATCTATGGTTGTTCAAGGATCCTTTCATGCATTATGTTAGGTATCAAGGAAAGTCAATTCTCGCTTCAAAGGAGAACTTTCTTTTGATGCGTAAATGGAAATATTACTTTGTACGTTTCTGGCAATGTCATTTTTACCAGTGGTTTCAACCAGGAAGGATCTATATAAACCAATTATCCAAACATTCCCTCGACCTTCTGGGCTATCTTTCAAGTGTGCGGCTAAATCCTTTAACGATACGCAGTCAAATGCTAGAAAATTCATTTATAATTGATAATGCTGGTAAGAAGTTCGATACCATTGTTCCAATTATTCCTCTGATTGGATCATTGGTTAAAGCGAAATTTTGTAACGTATTAGGGCATCCTGTTAGTAAGGTAGTTTGGGCAGATTTATCAGATTTAAGACAT-AAAATTTATCCTACTTCTTTATCTTTC-TAGGAAGGGCCTTTCATGAGTTGAATAGAGATTTTCA-TTTTTTATTCATCATTCGGGTTGATGAACTAAA-CCAGATAGTTATATGAGTGAAAGAAACAGCTTATAAATTTGCAGTAAAAAGATTGAGTCTCATTTT-CTATGTAC-AAGAG-TTAAGTGAAAGTAACCATAAACATTA-GAAACGGTTTACCCCAAGATTGGTTAATTAGTGATCATGGCTTGAAGCGGGTGC-AAAAGATCAACTATATGGGG--TTTTACTATCTATTACCATACATGTATTACCCTAACGGGCGATTAGCAAAAAGAGGTGGATAGTTAGGAACACCAAAGTACACAAAGGATTCGTAATAGAGATTATGTAAGTTATTCAACAGAATTTTTCTGTGCATAA-AAGGAATTCTGATTGGGACTTTAAGTTGGTAGAAATGATGAAGAAGTACTCCCCCTGATTCCGATCCAGAGTATACTCCTATCCACCGATTAAGTAAATAACTATCAAGAACGAAGTAATCCTTT-ACTTT------GTTTAAAGTCCCTTTTTCTGAGAAAGGAGAATAGGAACGAAAAAAATC-------AAATAG-AAAGA-ATAGAATTGCACTAGAAAGAAAAGAAAGAGATCTTTTTTTATTCTTTCTTTCC-------------TCTATTTAGAAAGATAGAATTCTTGTCATCATTCGTGAACTAATGCGATGCCTAATTGTTTTTCGTAATCGAAAATGCTAGGTTG--------AAATATCTATGAATA-TTGCTACAAGAAAGA-TTTTATTGAAAA-TTTAAGTTATCACTCAAC-AAAGAAAAATAAAAA-TTATT-----AAAAGATAAGATCAA-TTCCG-AAGCGCTTTA-TTTTCAATATAGCAGACAGAATTCCATTGTCTAATTCGGG--ACTTTACAGTAGATTTTGATTCTATCTATCCTACGAATATAT-------------------CAAGATAAATAATAGCGAACTGGTCCTTAGA-TTTATTTGTGA-CCTTTGAGGAGCC-GTATGAGATGAAAATCTCATGTACGGTATTTAGATTA---------------------------------------------------------------------------------------------------------------------------------------------------------------------------------GTAAA------AATATTTACGATTTTTCTTT-AA-----ATT--AAAAAAA-ATATGTAAGTCAA--------------------------------------------------------------------------------TGTGAAAT-AAAAAAGGAGCAATAATCCCCTTCTTGTTCTATCAAG-AGGGCGATATTGCTCCTTTTTT-ATTTCTTTCAAATACTCGTATACACTAAGGCCGGGTCTTATCCATTTATAGATGGAGCTTCAAGAGCAGCTAGGTCTAGAGGGAAGTTATGAGCATTACGTTCATGCATAACTTCCATACCAAGGTTAGCGCGGTTAATGATATCAGCCCAAGTATTAATTACACGACCTTGACTATCAACTACAGATTGGTTGAAATTAAAACCATTTAGGTTGAAAGCCATAGTGCTGATACCTAAAGCAGTGAACCAGATACCTACTACAGGCCAAGCAGCTAGGAAGAAATGTAAAGAACGAGAGTTGTTGAAACTAGCATATTGGAAGATCAATCGGCCAAAATAACCATGAGCAGCTACAATATTATAAGTTTCTTCTTCTTGA-CCGAATC---TGTAACCTTCATTAGCAATAAATGCAAGAATATTTAC-TTCCATAATCTCATCGTTTTTTTACTTCAAAATAACT-CGGGATTTAATCCCATAGAGATAATAAATCTTTCGCCTGTCAATTCA----ATGAATTACCTCTCGATGATC-TTGAAATCGGATCAATATCATGAATAACAATATCTGAGCTATCAAATCAATTCGTCGTCGAGAATTGAATAGTATAACATAGAAAGATCTTT-TATCCATACCG------AATCCAA------------------------AATTTCTTTATTTATCAATCATTCTTTTCTGTTCTTTCTTTATTTACAACCTATCTTAGGTCCTCCTTGTACAATCATCGGATAAAGTATCGTCTGACCGCCCGTCCGTTTCCATTAGTCACAAACGCCCAACAAACAATAGAAGCGAAGTGGAAAAAGAAATAAGTTACGTTCTAAACTCCG----TTTTTTTAATGATCTAGTTTTCTTGGAAGACAAAGAAGTGTGATAAAGAGGAGTTCCGGGATAAAGGATGTAATATTCCATCAAACTAACTATTTGAGTTTGGGTTTTGTTCGTTCTTCGACGGG--CCCT------AAAAAAAAAAATAG-AAAAAAAATAGGAAGGAAAAAT-GATTTATTCCCCCGCTACTTGTTAAGCTAAAAAAGGGGTGGGATCTTTGATTGATCTTTATTTTTCTTTTA-CCCCCCCTTCCTT---------------------------------------------------------------------------------------------------------------------------------------------------------------------------------------------------------------------------------------------------------------------------------------------------------------AACGCTCTCAATAATTGTACTATTCTACATATGTCTTTCTCCTACCAATCAGTATTATTTGAAATAATGAAAATTCCCCTATTTGTTTGATGAGAAGTGC----GAAATGCCAAAGGAAA--GAAAAAAGAACCCCCTT-GGGAATG-AAATTCTGCTCCCCGTGCCCCCTTTAACAGAAAAGGGAAGATTACAAATTGACTTATTATACTCCTGACTATGAAACCAAAGATACTGATATCTTGGCAGCATT-CCGAGTAACTCCTCAACCTGGAGTTCCACCTGAAGAAGCAGGGGCCGCGGTAGCTGCCGAATCTTCAACTGGTACATGGACAACTGTGTGGACCGATGGACTTACCAGCCTTGATCGTTACAAAGGGCGATGCTACCACATCGAGCCCGTTGCTGGAGAAGAAAATCAATATATTGCTTATGTAGCTTACCCATTAGACCTTTTTGAAGAAGGTTCTGTTACTAACATGTTTACTTCTATTGTGGGTAATGTATTTGGGTTCAAAGCCCTGCGCGCTCTACGTCTGGAAGATCTGCGAATCCCTGCCGCTTATACTAAAACTTTCCAAGGCCCGCCTCATGGCATCCAAGTTGAGAGAGATAAATTGAACAAGTATGGTCGCCCCCTGTTGGGATGTACTATTAAACCTAAATTGGGGTTATCTGCTAAAAACTATGGTAGAGCGGTTTATGAATGTCTACGTGGTGGACTTGATTTTACCAAAGATGATGAGAA--CG-------------------------------------------

Abelia_uniflora_BOP012275 CCTGAGCCAAATCCAGTTTTACGA----AAACAAGGGTTCAGAAAGCTAAAATC-AAAAAGGATAGGTGCAGAGACTCAATGGAAGCTGTTCTAACAAATGGAGTTGACTGTGTTGTGTTGGTAGAAAGAATCCTTCCATAGAAACTTCAGAAAGGATAAACGTATAAACATGGATATACGCATTGAAATACTAT-ATACTCTACCAAATGATTAATGACGACCCGAATCTGTATT--TA-----TATATATCAAAATGGGAGAATGGTTGTGAAGTGATTCCATATTGAAGAAAGAATCGAATATTTATTGATCAAATCATTCACTCCATAGTCTGATAGATC-TTTTGAAGAACTGATTAATTGGACGAGAATAAAGATAGAGTCCCATTCTACATGTCAATACCGGCAACAATGAAATTTATAGTAAAAGGAAAATCCGTCGACTTTAGAAATCGTGAGGGTTCAAGTCCCTCTATCCCCAAAAAACCCATATGGACTCCCTAATTATTTATCCTCTCCTT-TTATCC-TTTTTTGTTAGCGGTTAAAAATTCG------TTAGCTTTCTCATTCACCCTACTCTTTTACAAAGAGATCTGAGCGGAAATGTTTTTCTCTTATC----ACAAGTCTTGTGATCTAAG----ATAA-TACGTGTACAAATGAACATCTTTGAGTAAGGAATCCCCATTTAAATGATTCATGGTCAA------TGTCATTATTCATACTGAAACTTACAAAGTCTTCCTT-TTGAAGATCCAAGAAATTCCAGGACCTGGATAAGACTTTGTAAGACCCTTTCAATTGACATTAGACCCGAGTTATCTAGC--AAAATGAGGATGCAGCGGTATTTATGTTAATGCACTTCCCAATGATACGTAAACAAGGCATTTCTGGTCCTTTA--------TAGAGAAGATCTATCATAGATATTTGTAATCTATCATTTATCGCTTGGGGGAGGAACAATAGTATTTCATTGCTACAAGTATGGATTATTGAGAATAATAAGACATGTATTTGGATATTTCCCTTGAACTCCGCAATCTTTTTGATTTGACATGGATAGTTGAAGGGAATTTTCCGAAGAAAAAATGGATTAGATTATGGGAGTGTGTGACTTGAACTATTGATTGGTCTGTGCAGATATATGCCTTTCGCTATCTGCCACATTGGAATTCACAACCAAATGTGTCTTTGTTCCAACCATTGCGTAAGCCC----------CATACAGAGGATAGGCTGGTTCACTTGAAGAGAATCTTTTCTATGATCAGATCCGAATCATGTCGTACATGAGCAGGTTCCGTAAGATCCAGTAG-----AATAAGTG-AACTAGATAACCCATAATCGAGATTATGGGTTATCTAG-TTCACTTACATACGATTGAATAGTATGGAAATGCATTCATTTCCTATGCATTGACACGATCTATGATACTATCGGAGTGAAACAGGGGATCTAAGGAAGAACAGAGGCTAGGCTATATTAGTAACAAGTAAACCC-----TTTGTGTGTCTGTCAAAAGTCTCCAAGTATTTTGGGGATAAACACCGATCCTAAGGTCTGAGACGACCCAGAAAGCATT--TGATCCTATCATGATCCACCTTGTAAGCCTACTTGGGTATTGAGTATTTACTTGTAAGAACCAAAATTTTTGCGCTGGATAGTTGCAACTCCGGAAAAAGAAATCC------------------AGTCAAATTTTTC------TTTTAAT--------AAAATCATTCATATTATCATA-----TATGTGTGGATCTAGATAACATATAGATTTTATATGGATTCCTTATGGTTCTTTTTCTTTTTGCTCGAGCCGTATGATGAAAAATTATCATGTCCGGTTCCTTCGGGGGATGGAT-----CTATAAAAATTCACCTATCCCAATAACAAAAAAACCTGACCTGAATGATCCTGTATTAAGAGCTAAATTGGCTAAAGGTATGGGTCATAATTATTACGGAGAGCCCGCATGGCCCAATGATCTTTTATATATTTTTCCAGTAGAGATTG-----------------------AAG-----AAATCAATGCAATATTTTTTAGGAAAGATAAAACTGGATGAATTCAAATTCGTTCAAATGGG-----------------------ATTGGAAGGTTCCTTATTTTCATTTAGGGTGTTCGTTTTATTTCTTCCCTTAGGACTTTGGTGTAGTTTATGCTCTCTCTTCTGGAATCG-AATTGTTGT-AACTGGACGCTTCTATCCT-CTAGCTAGTA--GGGATAGAAC-----AAAAAAAATATTTTCA------TTTTTTAATGAATTTTTTCTCATTTATCCGATTTATCAAATTTGAAACAAAAAGATACATTTTTTCAATAAACACAAAAAAATCCTAAAAAATCCTAAAGTT---------ATACTATACAAAGGGTTGTCAAAATGGAATCAATTAGTTTCACCAATTCCTTC-----------------------ATTTTTACTAATGATCTTATA----TATGCCC-------TTCTATAGATATATATAGAG----------------ATAGAGAAACCC----------------------------ATTTTTCT-------TATTATA-----GATAAATAGGTTGATGGGGAAAATAAGACCCCGC-------CCTCGAAATGATAAAATCTACTAAAAAGAAAGGTAAAACCTTGTATCTTGTCTTTA--------------------TTTTCAAAAAAA--------TTCTTTTTTT-ATTTAGAAATTTAGTAAACAGAAGCATTTTTATTCTACTCCCATTCCCTATTGTTTTCGGCC-------AATGAATAGGGAATGGGAATTATTCATTTTATTTTTAGATTAACAATGAAATCAGAC---------AGTCAAATCAATTGGGATTATTCTAACGTTTTATGA--------CTTATTTGTTTGTCGTACAAAAAAACTTTTTACATTTCCGGTAGGTCGTACAAAAAGAC-TTTTTGAATTCCCAGT-ACAAAG------------------------------------------CATACACATGAAGTAA-----GAAAAAAGTCGTTGG-CTTTCTCTTTCTTTATGATATAG-------ATATGTACAACTTTGACCAGCAATTTCATTTAGATC-TAAGTAAGGGCTCGAAAGAT-CCAATAGACAAAT-------------ATAAAGAAAAATAAAGAAGACCC-----CTTTGATTTTGTTCCCTTTATTCCCACGGCCTGGCCTGGTCAATACCTAGCCGGGCC----TTTTTTTGTTCCAACAAATCCTAGCTAAAAGAATTTAGCTG-------------CTTTGAACACA---------------AAAATGCTTGCTATTAAAGCAGC-------------------AATAAAAAGATGA-GGGTTATTTCCATTCTTACTTAT--------------------------------------TATA--TATTATTTCTATTT------------------TATATATATAAATTATATAAAATCAAAGTATCCTTTCTTATT-----ATTCCTTCTTCCCTTTTGAGTTACTTGACGACCTTACGGGAAT--ATAAAATGAAACTG-----TGGGTTCTTA---AATAATAATGAATGCATTTTTCTGTTATGATTTCAGTGGTTTTAGTGAGCCATATCTATCAAAATCCCCCCAGCAAAAGAAAAAATAGAACTT--GTTATTTC----------------ATTTAGTTATTTAAAAGAGCCC-----TCCTTTCCGGAATCTCATTAAATCGAAATCCCCCGCGAA-AAACGT-CGACACTCTCATTTTCATGA-----TTATGATC-CTA--T-CTTTATTACGCTCAATTCCTC---------------------------------GTATTAG-TATATCTAATACCTTACCCAGCCCATTTGGAAATTTTGGTTCAAACTCTTCGCTACTGGGTAAAAGATGCCCCCTCTTTGCATTTATTACGATTCTTTCTTCACGACTATCGGCATTGGACTAGTCTTTTTATTCCAAAGAAAGCCAGTTCGTTTTTTTCAAAACGAAAG------------------CAAAGATTATTCTTCTTCCTATATAATTCTCATGTATGTGAATACGAATCCATCTTCGTCTTTCTCCGTAACCAATCTTCTCATTTACAATCAACATCTTCTGGAGCCCTTCTTGAACGAATATATTTCTATGAAAAAATAGAACATCTTGTCGAAGTCTTTGCTAAA-GATTTTCAAGGCAATCTATGGTTGTTCAAGGATCCTTTCATGCATTATGTTAGGTATCAAGGAAAGTCAATTCTCGCTTCAAAGGAGAACTTTCTTTTGATGCGTAAATGGAAATATTACTTTGTACGTTTCTGGCAATGTCATTTTTACCAGTGGTTTCAACCAGGAAGGATCTATATAAACCAATTATCCAAACATTCCCTCGACCTTCTGGGCTATCTTTCAAGTGTGCGGCTAAATCCTTTAACGATACGCAGTCAAATGCTAGAAAATTCATTTCTAATTGATAATGCTGTTAAGAAGTTCGATACCATTGTTCCAATTATTCCTCTGATTGGATCATTGGTTAAAGCGAAATTTTGTAACGTATTAGGGCATCCTGTTAGTAAGGTAGTTTGGGCAGATTTATCAGAT??????????????????????????????????????????????????????????????????????????????????????????????????????????????????????????????????????????????????????????????????????????????????????????????????????????????????????????????????????????????????????????????????????????????????????????????????????????????????????????????????????????????????????????????????????????????????????????????????????????????????????????????????????????????????????????????????????????????????????????????????????????????????????????????????????????????????????????????????????????????????????????????????????????????????????????????????????????????????????????????????????????????????????????????????????????????????????????????????????????????????????????????????????????????????????????????????????????????????????????????????????????????????????????????????????????????????????????????????????????????????????????????????????????????????????????????????????????????????????????????????????????????????????????????????????????????????????????????????????????????????????????????????????????????????????????????????????????????????????TATTTAGGTTA---------------------------------------------------------------------------------------------------------------------------------------------------------------------------------GTAAA------AATATTTACTATTTTTCTTTAAA-----TTAAAGAAAAAAAATATGTAAGTCAA--------------------------------------------------------------------------------TGTGAAAT-AAAAAAGGAGCAATAATCCCCTTCTTGTTCTATCAAG-AGGGCGATATTGCTCCTTTTTT-ATTTCTTTCAAATACTCGTATACACTAAGGCCGGGTCTTATCCATTTATAGATGGAGCTTCAAGAGCAGCTAGGTCTAGAGGGAAGTTATGAGCATTACGTTCATGCATAACTTCCATACCAAGGTTAGCGCGGTTAATGATATCAGCCCAAGTATTAATTACACRACCTTGACTATCAACTACAGATTGATTGAAATTAAAACCATTTAGGTTGAAAGCCATAGTGCTGATACCTAAAGCAGTGAACCARATACCTACTACAGGCCAAGCAGCTAGGAARAAATGTAAAGAACRAGAGTTGTTGAAACTAGCATATTGRAARATCAATCGGCCAAAATAACCATGAGCAGCTAMAATATTATAAGTTTCTTCTTCTTGA-CCGAATC---TGTAACCTTCATTAGCAATAAATGCAAGAATATTTAC-TTCCATAATCTCATCGTTTTTTTACTTCAAAATAACT-CGGGATTTAATCCCATAGAGATAATAAATCTTTCGCCTGTCAATTCA----ATGAATTACCTCTCGATGATC-TTGAAATCAGATCAATATCATGAATAACAATATCTGAGCTATCAAATCAATTCGTCGTCGAGAATTGAATAGTATAACATAGAAAGATYTTT-TATCCATACCG------AATCCAA------------------------AATTTCTTTATTTATCAATAATTCTTTTCTGTTCTTTCTTTATTTACAACCTATYTTAGGTCCTCCTTGTACAATCATCGGATAAAGTATYGTYTGACCGCCCGTCCGTTTCCATTAGTCACAAACGCCCAACAAACAATAGAAGCGAAGTGGAAAAAGAAATAAGTTACGTTCTAAACTCCG----TTTTTTTAATGATCTAGTTTTCTTGGAAGACAAAGAAGTGTGATAAAGAGGAGTTCCGGGATAAAGGATGTAATATTCCATCAAACTAACTATTTGAGTTTGGGTTTTGTTCGTTYTTYGACGGG--CCCT------AAAAAAAAAAATAG-AAAAAAAATAGGAAGGAAAAAT-GATTTATTCCCCCGCTACTTGTTAAGCTAAAAAAGGGGTGGGATCTTTGATTGATCTTTATTTTTCTTTTA-CCCCCCCTTCCTT---------------------------------------------------------------------------------------------------------------------------------------------------------------------------------------------------------------------------------------------------------------------------------------------------------------AACGCTCTCAATAATTGTACTATTCTACATATGTCTTTCTCCTACCAATCAGTATTATTTGAAATAATGAAAATTCCCCTATTTGTTTGATGAGAAGTGC----GAAATGCCAAAGGAAA-GAAAAAAAGAACACCCCTTGGGAATG-AAATTCTGC-----------------------------------------------------------------------------------------------------CAACTCCTCAACCTGGAGTTCCACCTGAAGAAGCAGGGGCCGCGGTAGCTGCCGAATCTTCAACTGGTACATGGACAACTGTGTGGACCGATGGACTTACCAGCCTTGATCGTTACAAAGGGCGATGCTACCACATCGAGCCCGTTGCTGGAGAAGAAAATCAATATATTGCTTATGTAGCTTACCCATTAGACCTTTTTGAAGAAGGTTCTGTTACTAACATGTTTACTTCTATTGTGGGTAATGTATTTGGGTTCAAAGCCCTGCGCGCTCTACGTCTGGAAGATCTGCGAATCCCTGTCGCTTATACTAAAACTTTCCAAGGCCCGCCTCATGGCATCCAAGTTGAGAGAGATAAATTGAACAAGTATGGTCGCCCCCTGTTGGGATGTACTATTAAACCTAAATTGGGGTTATCTGCTAAAAACTATGGTAGAGCGGTTTATGAATGTCTACGTGGTGGACTTGATTTTACCAAAGATGATGAGAA--CSTTCGAACTCCCAACCATTTATGGGGG-----------------

Acanthocalyx_albus ???????????????????????????????????????????????????????????????????????????????????????????????????????????????????????????????????????????????????????????????????????????????????????????????????????????????????????????????????????????????????????????????????????????????????????????????????????????????????????????????????????????????????????????????????????????????????????????????????????????????????????????????????????????????????????????????????????????????????????????????????????????????????????????????????????????????????????????????????????????????????????????????????????????????????????????????????????????????????????????????????????????????????????????????????????????????????????????????????????????????????????????????????????????????????????????????????????????????????????????????????????????????????????????????????????????????????????????????????????????????????????????????????????????????????????????????????????????????????????????????????????????????????????????????????????????????????????????????????????????????????????????????????????????????????????????????????????????????????????????????????????????????????????????????????????????????????????????????????????????????????????????????????????????????????????????????????????????????????????????????????????????????????????????????????????????????????????????????????????????????????????????????????????????????????????????????????????????????????????????????????????????????????????????????????????????????????????????????????????????????????????????????????????????????????????????????????????????????????????????????????????????????????????????????????????????????????????????????????????????????????????????????????????????????????????????????????????????????????????????????????????????????????????????????????????????????????????????????????????????????????????????????????????????????????????????????????????????????????????????????????????????????????????????????????????????????????????????????????????????????????????????????????????????????????????????????????????????????????????????????????????????????????????????????????????????????????????????????????????????????????????????????????????????????????????????????????????????????????????????????????????????????????????????????????????????????????????????????????????????????????????????????????????????????????????????????????????????????????????????????????????????????????????????????????????????????????????????????????????????????????????????????????????????????????????????????????????????????????????????????????????????????????????????????????????????????????????????????????????????????????????????????????????????????????????????????????????????????????????????????????????????????????????????????????????????????????????????????????????????????????????????????????????????????????????????????????????????????????????????????????????????????????????????????????????????????????????????????????????????????????????????????????????????????????????????????????????????????????????????????????????????????????????????????????????????????????????????????????????????????????????????????????????????????????????????????????????????????????????????????????????????????????????????????????????????????????????????????????????????????????????????????????????????????????????????????????????????????????????????????????????????????????????????????????????????????????????????????????????????????????????????????????????????????????????????????????????????????????????????????????????????????????????????????????????????????????????????????????????????????????????????????????????????????????????????????????????????????????????????????????????????????????????????????????????????????????????????????????????????????????????????????????????????????????????????????????????????????????????????????????????????????????????????????????????????????????????????????????????????????????????????????????????????????????????????????????????????????????????????????????????????????????????GTATTAG-TATACCTAATACCTTACCCAGCCCATTTGGAAATTTTGGTTCAAACTCTTCGCTACTGGGTAAAAGATGCCCCTTCTTTGCATTTATTACGATTCTTTCTCCACGACTATCGGCATTGGACTAGGCTTTATATTCCAAAGAAAGCCAGTTCTTTTTTTTCAAAACGAAAG------------------CAAAGATTATTCTTCTTACTATATAATTCTCATGTATGTGAATACGAATCCATCTTTGTCTTTCTCCGTAACCAATCTTCTCATTTACAATCAACATCTTCGGGAGCCCTTCTTGAACGAATATATTTCTATGAAAAAATAGAACACCTTGTAGAAGTCTTTGCTAAA-GATTTTCAAGGAAATCTATGGTTGTGCAAGGATCCTTTCATTCATTATGTTAGGTATCAAGGAAAGTCAATTCTCGCTTCAAAGGGGAACTTTCTTTTGATGCATAAATGTAAATATTACTTTGTACATTTCTGGCAATGTCATTTTTACCAGTGGTTTCAACCAGGAAGGATCCATATAAACCAATTATCCAAACATTCCCTCGACCTTCTGGGCTATCTTTCAAGTGTGCGGCTAAACCCTTTAACGGTACGCAGTCAAATGCTAGCAAATTCATTTCTAATCGATAATGCTGTTAAGAAGTTCGATACCATTGTTCCAATTATTCCTCTGATTGGATCATTGGTTAAAGCGAAATTTTGTAACGTATTAGGGCATCCTCTTAGTAAGGTAGTTTGGGCAGATTTATCAGAT?????????????????????????????????????????????????????????????????????????????????????????????????????????????????????????????????????????????????????????????????????????????????????????????????????????????????????????????????????????????????????????????????????????????????????????????????????????????????????????????????????????????????????????????????????????????????????????????????????????????????????????????????????????????????????????????????????????????????????????????????????????????????????????????????????????????????????????????????????????????????????????????????????????????????????????????????????????????????????????????????????????????????????????????????????????????????????????????????????????????????????????????????????????????????????????????????????????????????????????????????????????????????????????????????????????????????????????????????????????????????????????????????????????????????????????????????????????????????????????????????????????????????????????????????????????????????????????????????????????????????????????????????????????????????????????????????????????????????????????????????????????????????????????????????????????????????????????????????????????????????????????????????????????????????????????????????????????????????????????????????????????????????????????????????????????????????????????????????????????????????????????????????????????????????????????????????????????????????????????????????????????????????????????????????????????????????????????????????????????????????????????????????????????????????????????????????????????????????????????????????????????????????????????????????????????????????????????????????????????????????????????????????????????????????????????????????????????????????????????????????????????????????????????????????????????????????????????????????????????????????????????????????????????????????????????????????????????????????????????????????????????????????????????????????????????????????????????????????????????????????????????????????????????????????????????????????????????????????????????????????????????????????????????????????????????????????????????????????????????????????????????????????????????????????????????????????????????????????????????????????????????????????????????????????????????????????????????????????????????????????????????????????????????????????????????????????????????????????????????????????????????????????????????????????????????????????????????????????????????????????????????????????????????????????????????????????????????????????????????????????????????????????????????????????????????????????????????????????????????????????????????????????????????????????????????????????????????????????????????????????????????????????????????????????????????????????????????????????????????????????????????????????????????????????????????????????????????????????????????????????????????????????????????????????????????????????????????????????????????????????????????????????????????????????????????????????????????????????????????????????????????????????????????????????????????????????????????????????????????????????????????CTGATTACAAATTGACTTATTATACTCCTGACTATGAAACCAAAGATACTGATATCTTGGCAGCATT-CCGAGTAACTGCTCAACCTGGAGTTCCACCTGAAGAAGCAGGGGCCGCGGTAGCTGCCGAATCTTCAACTGGTACATGGACAACTGTGTGGACCGATGGACTTACCAGCCTTGATCGTTACAAAGGGCGATGCTACCACATCSAGCCCGTTCCTGGAGAAGAAAGTCAATTTATTGCTTATGTAGCTTACCCATTAGACCTTTTTGAAGAAGGTTCTGTTACTAACATGTTTACTTCTATTGTGGGTAATGTATTTGGGTTCAAAGCCCTGCGCGCTCTACGTCTGGAAGATCTGCGAATCCCTGTCGCTTATGTTAAAACTTTCCAAGGCCCGCCTCATGGCATCCAAGTTGAGAGAGATAAATTGAACAAGTWTGGTCCCCCCTTGTTGGGATGTACTATTAAACCTAAATTGGGGTTATTTGCTAAAAACTATGGTAGAGCGGTTTATGAATGGCTACGTGGKGGACTTGATTTTACCAAAGATGATGAGAA--CGT--GAACTCCCAACCATTTATGCGTT---GGAGAGATCGTTTT

Cryptothladia_chinensis CCTGAGCCAAATCCAGTTTTCCGAAAACAAACAAGGGTTCAGAAAGCTAAAATC-AAAAAGGATAGGTGCAGAGACTCAATGGAAGCTGTTCTAACAAATGGAGTTGAC-----TGTGTTGGTAGAAAGAATCCTTCCATAGAAACTTCAGAAAGGATAAACCTATAAACATAGATATATGCATTGAAATACTAT-ATATTCTACCAAATGATTAATGACAACCCGAATCTGTATATATATATATTATATATCAAAATGGGAGA-----TGTGAAGTGATTCCATATTGAAGAAAGAATCGAATATTCATTGATCAAATCATTCACTCCATAGTCTGATAGATCTTTTTAAAGAACTGATTAATCGGACGAGAATAAAGATAGAGTCCCATTCTACATGTCAATACCGGCAACAATGAAATTTATAGTAAGAGGAAAATCCGTCGACTTTAGAAATCGTGAGGGTTCAAGTCCCTCTATCCCCAAAAAACCCATATGAACTCTCTAATTCTTTATTCTCTCCTT-TTATGC-TTTTTTGTTAGCGGTTCCAAATTCGTTATCTTTATCTTTCTCATTCACCCTACTCTTTTACAAAGAGATCTGAGCGGAAATATTTTTCTCTTATC----ACAAGTCTTGGGATCTAAG----ATAA-TCCGTGTACAAATGAACATCTTTGAGTAAGGAATCCCCATTTGAATGATTCATGATCAA------TATCATTATTCATACTGAAACTTACAAAGTCTTCCTTATTGAAGATCCAAGAAATTCCAGGACCTGGATAAGACTTTGTAATA-CCTTTCAATTGACA-TAGACCCGAGTTATCTATC--AAAATGAGGATGCA?????????????????????????????????????????????????????????????????????????????????????????????????????????????????????????????????????????????????????????????????????????????????????????????????????????????????????????????????????????????????????????????????????????????????????????????????????????????????????????????????????????????????????????????????????????????????????????????????????????????????????????????????????????????????????????????????????????????????????????????????????????????????????????????????????????????????????????????????????????????????????????????????????????????????????????????????????????????????????????????????????????????????????????????????????????????????????????????????????????????????????????????????????????????????????????????????????????????????????????????????????????????????????????????????????????????????????????????????????????????????????????????????????????????????????????????????????????????????????????????????????????????????????????????????????????????????????????????????????????????????????????????????????????????????????????????????????????????????????????????????????????????????????????????????????????????????????????????????????????????????????????????????????????????????????????????????????????????????????????????????????????????????????????????????????????????????????????????????????????????????????????????????????????????????????????????????????????????????????????????????????????????????????????????????????????????????????????????????????????????????????????????????????????????????????????????????????????????????????????????????????????????????????????????????????????????????????????????????????????????????????????????????????????????????????????????????????????????????????????????????????????????????????????????????????????????????????????????????????????????????????????????????????????????????????????????????????????????????????????????????????????????????????????????????????????????????????????????????????????????????????????????????????????????????????????????????????????????????????????????????????????????????????????????????????????????????????????????????????????????????????????????????????????????????????????????????????????????????????????????????????????????????????????????????????????????????????????????????????????????????????????????????????????????????????????????????????????????????????????????????????????????????????????????????????????????????????????????????????????????????????????????????????????????????????????????????????????????????????????????????????????????????????????????????????????????????????????????????????????????????????????????????????????????????????????????????????????????????????????????????????????????????????????????????????????????????????????????????????????????????????????????????????????????????????????????????????????????????????????????????????????????????????????????????????????????????????????????????????????????????????????????????????????????????????????????????????????????????????????????????????????????????????????????????????????????????????????????????????????????????????????????????????????????????????????????GTATTAG-TATACCTAATACCTTACCCAGCCCATTTGGAAATTTTGGTTCAAACTCTTCGCTACTGGGTAAAAGATGCCCCTTCTTTGCATTTATTACGATTCTTTCTCCACGACTATCAGCATTGGACTAGTCTTTATATTCCAAAGAAAGCCAGTGCTTTTTTTTCAAAACGAAAGCAAAGATTATTCTTCTTCCAAAGATTATTCTTCTTCCTATATAATTCGCATGTATGTAAATACGAATCCATCTTTGTCTTTCTCCGTAACCAATCTTCTCATTTACAATCAACATCTTCGGGAGCCCTTCTTGAACGAATATCTTTCTATGAAAAAATGGAGCATCTTGTAGAAGTCTTTGCTAAA-GATTTTCAAGTCAATCTATGGTTGTGCAAGGATCCTTTCATGCATTATGTTAGGTATCAAGGAAAGTCAATTCTCGCTTCAAAGGGGAACTTTCTTTTGATGCATAAATGTAAATATTACTTTGTACGTTTCTGGCAATGTCATTTTTACCAGTGGTTTCAACCAGGAAGGATCTATATAAACCAATTATCCAAACATTCCCTCGACCTTCTGGGCTATCTTTCAAGTGTGCGGCTAAACCCTTTAACGGTACGCAGTCAAATGCTAGCAAATTCATTTCTAATCGATAATGCTGTTAAGAAGTTCGATACCATTGTTCCAATTATTCCTCTGATTGGATCATTGGTTAAAGCGAAATTTTGTAACGTATTAGGGCATCCTGTTAGTAAGGTAGTTTGGGCAGATTTATCAGAT?????????????????????????????????????????????????????????????????????????????????????????????????????????????????????????????????????????????????????????????????????????????????????????????????????????????????????????????????????????????????????????????????????????????????????????????????????????????????????????????????????????????????????????????????????????????????????????????????????????????????????????????????????????????????????????????????????????????????????????????????????????????????????????????????????????????????????????????????????????????????????????????????????????????????????????????????????????????????????????????????????????????????????????????????????????????????????????????????????????????????????????????????????????????????????????????????????????????????????????????????????????????????????????????????????????????????????????????????????????????????????????????????????????????????????????????????????????????????????????????????????????????????????????????????????????????????????????????????????????????????????????????????????????????????????????????????????????????????????????????????????????????????????????????????????????????????????????????????????????????????????????????????????????????????????????????????????????????????????????????????????????????????????????????????????????????????????????????????????????????????????????????????????????????????????????????????????????????????????????????????????????????????????????????????????????????????????????????????????????????????????????????????????????????????????????????????????????????????????????????????????????????????????????????????????????????????????????????????????????????????????????????????????????????????????????????????????????????????????????????????????????????????????????????????????????????????????????????????????????????????????????????????????????????????????????????????????????????????????????????????????????????????????????????????????????????????????????????????????????????????????????????????????????????????????????????????????????????????????????????????????????????????????????????????????????????????????????????????????????????????????????????????????????????????????????????????????????????????????????????????????????????????????????????????????????????????????????????????????????????????????????????????????????????????????????????????????????????????????????????????????????????????????????????????????????????????????????????????????????????????????????????????????????????????????????????????????????????????????????????????????????????????????????????????????????????????????????????????????????????????????????????????????????????????????????????????????????????????????????????????????????????????????????????????????????????????????????????????????????????????????????????????????????????????????????????????????????????????????????????????????????????????????????????????????????????????????????????????????????????????????????????????????????????????????????????????????????????????????????????????????????????????????????????????????????????????????????????????????????????????????????AAGATTACAAATTGACTTATTATACTCCTGACTATGAAACCAATTATACTGATATCTTGGCACCATT-CCGAGTAACTCCTCAACCTGGAGTTCCACCGGAAGAAGCAGGGGGCCGGGTAGCTGCCGAATCTTCAACTGGTACATGGACAACTGTGTGGACCGATGGACTTACCAGCCTTGATCGTTACAAAGGGCGATGCTACCACATCCAGCCCGTTCCTGGAGAAGAAAATCAATTTATTGCTTATGTAGCTTACCCATTAGACCTTTTTGAAGAAGGTTCTGTTACTAACATGTTTACTTCTATTGTGGGTAATGTATTTGGGTTCAAAGCCCTGCGCGCTCTACGTCTGGAAGATCTGCGAATCCCTGTCGCTTATGTTAAAACTTTCCAAGGCCCGCCTCATGGCATCCAAGTTGAGAGAGATAAATTGAACAAGTATGGTCCCCCCTTGTTGGGATGTACTATTAAACCTAAATTGGGGTTATCTGCTAAAAACTATGGTAGAGCGGTTTATGAATGTCTACGTGGTGGACTTGATTTTACCAAAGATGATGAGAA--CGT--GAACTCCCAACCATTTATGCGTT---GGAAARATTGTTTC

Diabelia_serrata_BOP012266 CCTGAGCCAAATCCAGTTTTACGAAAACAAACAAGGGTTCAGAAAGCTAAAATC-AAAACGGATAGGTGCAGAGACTCAATGGAAGCTGTTCTAACAAATGGAGTTGACTGTGTTGTGTTGGTAGAAAGAACCCTTCCATAGAAACTTCAGAAAGGATAAACGTATAAACATAGATATACGCATTGAAATACTAT-ATAATCTACCAAATGATTAATGACGACGCGAATCTGTATT--TA-----TATATATCAAAATGGGAGAATGGTTGTGAAGTGATTCCATATTGAAGAAAGAATCGAATATTTATTGATCAAATCATTCACTCCATAGTCTGATAGATC-TTTTGAAGAACTGATTAATTGGACGAGAATAAAGATAGAGTCCCATTCTACATGTCAATACCGGCAACAATGAAATTTATAGTAAGAGGAAAATCCGTCGACTTTAGAAATCGTGAGGGTTCAAGTCCCTCTATCCCCAAAAAAACCATATGGACTCCCTAATTATTTATCCTCCCCTT-TTATCC-TTTTTTGTTAGCGGTTAAAAATTCG------TTATCTTTCTCATTCACCCTACTCTTTTACAAAGAGATCTGAGCGGAAATGTTTTTCTCTTATC----ACAAGTCTTGTGATCTAAG----ATAA-TACGTGTACAAATGAACATCTTTGAGTAAGGAATCCCCATTTAAATGATTCATGGTCAA------TGTCATTATTCATACTGAAACTTACAAAGTCTTCCTT-TTGAAGATCCAAGAAATTCCAGGACCTGGATAAGACTTTGTAAGACCCTTTCAATTGACA-TAGACCCGCGTTATCTAGC--AAAATGAGGATGCAGCGGTATTTATGTTAATGCACTTCCCAATGATACGTAAACAAGGCATTTCTGGTCCTTTA--------TAGAGAAGATCTATCATAGATATTTGTAATCTATCATTTATCGCTTGGGGGAGGAACAATAGTATTTCATTGCTACAAGTATGGATTATTGAGAATAATAAGACATGTATTTGGATATTTCCCTTGAACTCCGCAATCTTTTTGATTTGACATGGATAGTTGAAGGGAATTTTCCGAAGAAAAAATGGATTAGATTATGGGAGTGTGTGACTTGAACTATTGATTGGTCTGTGCAGATATATGCCTTTCGCTATCTGCCACATTGCAATTCACAACCAAATGTGTCTTTGTTCCAACCATTGCGTAAGCCC----------CATACAGAGGATAGGCTGGTTCACTTGAAGAGAATCTTTTCTATGATCAGATCCGAATCATGTCGTACATGAGCAGGTTCCGTAAGATCCAGTAG-----AATAAGTG-AACTAGATAACCCATAATCGAGATTATGGGTTATCTAG-TTCACTTACATACGATTGAATAGTATGGAAATGCATTCATTTCCTATGCATTGACACGATCTATGATACTATCGGAGTGAAACAGGGGATCTAAGGAAGAACAGAGGCTAGGCTATATTAGTAACAAGTAAACCC-----TTTGTGTGTCTGTCAAAAGTCTCCAAGTATTTTGGGGATAAACACCGATCCTAAGGTCTGAGACGACCCAGAAAGCATT--TGATCCTATCATGATCCACCTTGTAAGCCTACTTGGGTATTGAGTATTTACTTGTAAGAACCAAATTTTTTGCGCTGGATAGTTGCAACTCCGGAAAAAGAAATCC------------------AGTCAAATTTTTC------TTTTAAT--------AAAATCATTCATATTATTATA-----TATGTGTGGATCTAGATAACATATAGATTTTATATGGATTCCTTATGGTTCTTTTTCTTTTTGCTCGAGCCGTATGATGAAAAATTATCATGTCCGGTTCCTTCGGGGGATGGAT-----CTATAAAAATTCACCTATCCCAATAACAAAAAAACCTGACCTGAATGATCCTGTATTAAGAGCTAAATTGGCTAAAGGTATGGGTCATAATTATTACGGAGAGCCCGCATGGCCCAATGATCTTTTATATATTTTTCCAGTAGAGATTG-----------------------AAG-----AAATCAATGCAATATTTTTTAGGAAAGATAAAACTGGATGAATTCAAATTCGTTCAAATGGG-----------------------ATTGGAAGGTTCCTTATTTTCATTTAGGGTGTTCGTTTTATTTCTTTTCTTAGGACTTTGGTGTAGTTTATGCTCTCTCTCCTGGAATCG-AATTGTTGT-AACTGGACGCTTCTATCCTACTAGCTAGTA--GGGATAGAAC-------AAAAAATATTTTCA------TTTTTTAATGAATTCTTTCTCATTTATCCGATTTATCAAATTTGAAACAAAAAGATACATTGTTTCAATAAACAC-----------AAAAAATCCTAAAGTT---------ATACTATACAAAAGGTTGTCAAAATTGAATCAATTAGTTTCAACAATTCCTTT-----------------------ATTTTTACTAATGATCTTACA----TATGCCCTTCTATATTCTATAGATATATATAGAA----------------ATAGAGAAACCC----------------------------ATTTTTCT-------TATTATA-----GATAAATAGGTTGATGGGGAAAATAAGACCCCGC-------CCTCGAAATGATAAAATCTACTAAAAAGAAAGGTAAAACCTTGTATCTTGTCTTTATTC---------------TTTTTTCAAAAAAA--------TTCTTTTTAAAATTTAGAAATTTAGTAAACAGAAGCATTTTTATTCTACTTCCATTCCCTATTCATT--GGCCGAAAACAAAACAATAGGGAATGGGAATTATTCATTTTATTTTTAGATTAACAATGAAATCAGAT---------AGTCAAATCAATTGGGATTATTCTAACGTTTTATGA--------CTTATTTGTTTGTCGTACAAAAAAACTTTTTACATTCCCGGTAGGTCGTACAAAAAAAC-TTTTTGAATTCCCGGT-ACAAAGGCCATCTCCCCCAATTGAACAAGAGAATAATGACTATGTTACATTACACATGAAGTAA-----GAAAAAAGTC--TTG-CTTTCTCTTTCTTTATGATATAG-------ATATGTACAACTTTGACCAGCAATTTCATTTAGATC-TAAGTAAGGGCTCGAAAGAT-CCAATAGACAAAT-------------ATAAAGAAAAATAAAGAAGACCC-----TTTTGATTTTGTTCCCTTTATTCCCACGGCCTGGCCTGGTCAATACCTAGCCGGGCC----TTTTTTTGTTCCAACAAATCCTAGCTAAAAGAATTTAGCTG-------------CTTTGAACACA---------------AAAATGCTTGCTATTAAAGCAGC-------------------AATAAAAAGATGAGGGGTTATTTCCATTCTTACTTATTATTCCAT------------------TCTTACTTATTATATA--TATTATTTATATTTATATATATATTAT--TTATATTTATATAAATTATATAAAATCAAAGTATCCTTTCTTATT-----ATTCCTTCTTCCCTTTTGAGTTACTTGACGACCTTATGGGAAT--ATAAAATGAAACTG-----TGGGTTCTTA---AATAATAATGAATGCATTTTTCTGTTATGATTTCAGTGGTTTTAGTGAGCCATATCTATCAAAATCCCCCTAGCAAAAGAAAAAATAGAACTT--GTTATTTC----------------ATTTAGTTATTTAAAAGAGCCC-----TCCTTTCCGGAATCTCATTAAATTGAAATCCCCCGCGAA-AAACGT-CGACACTCTCATTTTCATGA-----TTATGATC-CTA--T-CTTTATTACGCCTAATTCCTCTGTT-CGACAAAAAGTTCATTTGTATATAATAAGTATTAG-TATATCTAATACCTTACCCAGCCCATTTGGAAATTTTGGTTCAAACTCTTCGCTACTGGGTAAAAGATGCCCCCTCTTTGCATTTATTACGATTCTTTCTTCACGACTATCGGCATTGGACTAGTCTTTTTATTCCAAAGAAAGCCAGTTCTTTTTTTTCAAAACGAAAG------------------CAAAGATTATTCTTCTTCCTATATAATTCTCATGTATGTGAATACGAATCCATCTTCGTCTTTCTCCGTAACCAATCTTCTCATTTACAATCAACATCTTCTGGAGCCCTTTTTGAACGAATATATTTCTATGAAAAAATAGAACATCTTGTCGAAGTCTTTGCTAAA-GATTTTCAAGGCAATCTATGGTTGTTCAAGGATCCTTTCATGCATTATGTTAGGTATCAAGGAAAGTCAATTCTCGCTTCAAAGGAGAACTTTCTTTTGATGCGTAAATGGAAATATTACTTTGTACGTTTCTGGCAATGTCATTTTTACCAGTGGTTTCAACCAGGAAGGATCTATATAAACCAATTATCCAAACATTCCCTCGACCTTCTGGGCTATCTTTCAAGTGTGCGGCTAAACCCTTTAACGATACGCAGTCAAATGCTAGAAAATTCACTTCTAATTGATAATGCTGTTAAGAAGTTCGATACCATTGTTCCCATTATTCCTCTGATTGGATCATTGGTTAAAGCGAAATTTTGTAACGTATTAGGGCATCCTGTTAGTAAGGTAGTTTGGGCAGATTTATCAGATTTAAGACAT-AAAATTTACCCTACTTCTTTATCTTTT-TAGGAAGGGCCTTTCATGAGTTGAATAGAGATTTTCA-TTTTTTATTCATCATTCGGGTTGATGAACTAAA-CCAGATAGTTATATGAGTGAAAGAAACAGCTTATAAATTTGCAGTAAAAAGATTGAGTCTCATTTT-CTATGTAC-AAGAG-TTAAGTGAAAGTAACCATAAACATTA-GAAACGGTTTACCCCAAGATTGGTTAATTAGTGATCATGGCTTGAAGCGGGTGC-AAAAGATCAACTGTATGGGG-TTTTTACTATCTATTACCATACATGTATTACCCTAACGGGCGATTAGCAAAAAGAGGTGGATAGTTAGGAACACCAAGGTACACAAAGGATTCGTAATAGAGATTATGTAAGTTATTCAACAGAATTTTTCTGTGCATAA-AAGGAATTCTGATTGGGACTTTAAGTTGGTAGAAATGATGAAGAAGTACTCCCCCTGATTCCGATCCAGAGTATACTCCTATCCACCGATTAAGTAAATAACTATCAAGAACGAAGTAATCCTTT-ACTTT------GTTTAAAGTCCCTTTTTCTGAGAAAGGAGAATAGGAACGAAAAAAATC-------AAATAG-AAAGA-ATAGAATTGCACTAG-----AAAGAAAGAGATCT-----------TTCTTTCC-------------TCTATTTAGAGAGATAGAATTCTTGTCATCATTCGTGAACTAATGCGATGCCTAATTGTTTTTCGTAATCGAAAATGCTAGGTTG--------AAATATCTATGAATA-TTGCTACAAGAAAGA-TTTTATTGAAAG-CTTAAGTTATCACTCAAC-AAAGAAAAATAAAAA-TTATT-----AAAAGATAAGATCAA-TTCCG-AAGCACTTTA-TTTTCAATATAGCAGACAGAATTCCATTGTCTAATTCGGG--ACTTTACGGTAGATTTTGATTCTATCTATCCTACGAATATAT-------------------CAAGATAAATAATAGCGAACTGGTCCTTAGA-TTTATTTGTGA-CCTTTGAGGAGCC-GTATGAGATGAAAATCTCATGTACGGTATTTAGATTA---------------------------------------------------------------------------------------------------------------------------------------------------------------------------------ATAAAAATATTAATATTAACGACTTTTCTTT-AA-----------AGAAA--ATATGTAAGTCAA--------------------------------------------------------------------------------TGTGAAAT-AAAAAAGGAGCAATA-TCCCCTCCTTGTTCTATCAAG-AGGGGGATATTGCTCCTTTTTT-ATTTCGTTCAAATACTCGTATACACTAAGGCCGGGTCTTATCCATTTATAGATGGAGCTTCAAGAGCAGCTAGGTCTAGAGGGAAGTTATGAGCATTACGTTCATGCATAACTTCCATACCAAGGTTAGCGCGATTAATGATATCAGCCCAAGTATTAATTACACGACCTTGACTATCAACTACAGATTGGTTGAAATTAAAACCATTTAGGTTGAAAGCCATAGTGCTGATACCTAAAGCAGTGAACCAGATACCTACTACAGGCCAAGCAGCTAGGAAGAAATGTAAAGAACGAGAGTTGTTGAAACTAGCATATTGGAAGATCAATCGGCCAAAATAACCATGAGCAGCTACAATATTATAAGTTTCTTCTTCTTGA-CCGAATC---TGTAACCTTCATTAGCAATAAATGCAAGAATATTTAC-TTCCATAATCTTATCGTTTTTTTACTTCAAAATAACTCCGGGATTTAATCCCATAGAGATAATAAATCTTTCGCCTGTCAATTCA----ATGAATTACCTCTCGATGATCTTTGAAATCGGATCAATATCATGAATAACAATATCTGAGCTATCAAATCAATTCGTCGTCGAGAATTGAATAGTATAACATAGAAAGATCTTTGTATCCATACCGAATCCAAATCCAA------------------------AATTTCTTTATTTATCAATCATTCTTTTCTGTTCTTTCTTTATCTACAACCTATCTTAGGTCCTCCTTGTACAATCATCGGATAAAGTATCGTCTGACCGCCCGTCCGTTTCCATTAGTCACAAACGCCCAACAAACAATAGAAGCGAAGTGGAAAAAGAAATAAGTTACGTTCTAAACTCCG----TTTTTTTAATGATCTAGTTTTCTTGGAAGACAAAGAAGTGTGATAAAGAGGAGTTCCGGGATAAAGGATGTAATATTCCATCAAACTAACTATTTGAGTTTGGGTTTTGTTCGTTCTTCGACGGG--CCCT------AAAAAAAAAAATAG--AAAAAAATAGGAAGGAAAAAT-GATTTATTCCCCTGCTACTTGCTAAGCTAAAAAAGGGGTGGGATCTTTGATTGATCTTTATTTTTCTTTTA-CCCCCCCTTCCTT---------------------------------------------------------------------------------------------------------------------------------------------------------------------------------------------------------------------------------------------------------------------------------------------------------------AACGCTCTCAATAATTGTACTATTCTACATATGTCTTTCTCCTACCAATCAGTATTATTTGAAATAATGAAAATTCCCCTATTTGTTTGATGAGAAGTGC----GAAATGCCAAAGGAAA--GAAAAAAGAACCCCCTT-GGGAATG-AAATTCTGCTCCCCGTGCCCCCTTTAACAGAAAAGGGAAGATTACAAATTGACTTATTATACTCCTGACTATGAAACCAAAGATACTGATATCTTGGCAGCATT-CCGAGTAACTCCTCAACCTGGAGTTCCACCTGAAGAAGCAGGGGCCGCGGTAGCTGCCGAATCTTCAACTGGTACATGGACAACTGTGTGGACCGATGGACTTACCAGCCTTGATCGTTACAAAGGGCGATGCTACCACATCGAGCCCGTTGCTGGAGAAGAAAATCAATTTATTGCTTATGTAGCTTACCCATTAGACCTTTTTGAAGAAGGTTCTGTTACTAACATGTTTACTTCTATTGTGGGTAATGTATTTGGGTTCAAAGCCCTGCGCGCTCTACGTCTGGAAGATCTGCGAATCCCTGTCGCTTATGTTAAAACTTTCCAAGGCCCGCCTCATGGCATCCAAGTTGAGAGAGATAAATTGAACAAGTATGGTCGCCCCCTGTTGGGATGTACTATTAAACCTAAATTGGGGTTATCTGCTAAAAACTATGGTAGAGCGGTTTATGAATGTCTACGTGGTGGACTTGATTTTACCAAAGATGATGAGAA--TGT--GAACTCCCAACCATTTATGCGTT---GGAGAGATCGATTC

Diabelia_serrata_BOP012269 CCTGAGCCAAATCCAGTTTTACGAAAACAAACAAGGGTTCAGAAAGCTAAAATC-AAAACGGATAGGTGCAGAGACTCAATGGAAGCTGTTCTAACAAATGGAGTTGACTGTGTTGTGTTGGTAGAAAGAACCCTTCCATAGAAACTTCAGAAAGGATAAACGTATAAACATAGATATACGCATTGAAATACTAT-ATAATCTACCAAATGATTAATGACGACGCGAATCTGTATT--TA-----TATATATCAAAATGGGAGAATGGTTGTGAAGTGATTCCATATTGAAGAAAGAATCGAATATTTATTGATCAAATCATTCACTCCATAGTCTGATAGATC-TTTTGAAGAACTGATTAATTGGACGAGAATAAAGATAGAGTCCCATTCTACATGTCAATACCGGCAACAATGAAATTTATAGTAAGAGGAAAATCCGTCGACTTTAGAAATCGTGAGGGTTCAAGTCCCTCTATCCCCAAAAAAACCATATGGACTCCCTAATTATTTATCCTCCCCTT-TTATCC-TTTTTTGTTAGCGGTTAAAAATTCG------TTATCTTTCTCATTCACCCTACTCTTTTACAAAGAGATCTGAGCGGAAATGTTTTTCTCTTATC----ACAAGTCTTGTGATCTAAG----ATAA-TACGTGTACAAATGAACATCTTTGAGTAAGGAATCCCCATTTAAATGATTCATGGTCAA------TGTCATTATTCATACTGAAACTTACAAAGTCTTCCTT-TTGAAGATCCAAGAAATTCCAGGACCTGGATAAGACTTTGTAAGACCCTTTCAATTGACA-TAGACCCGCGTTATCTAGC--AAAATGAGGATGCAGCGGTATTTATGTTAATGCACTTCCCAATGATACGTAAACAAGGCATTTCTGGTCCTTTA--------TAGAGAAGATCTATCATAGATATTTGTAATCTATCATTTATCGCTTGGGGGAGGAACAATAGTATTTCATTGCTACAAGTATGGATTATTGAGAATAATAAGACATGTATTTGGATATTTCCCTTGAACTCCGCAATCTTTTTGATTTGACATGGATAGTTGAAGGGAATTTTCCGAAGAAAAAATGGATTAGATTATGGGAGTGTGTGACTTGAACTATTGATTGGTCTGTGCAGATATATGCCTTTCGCTATCTGCCACATTGCAATTCACAACCAAATGTGTCTTTGTTCCAACCATTGCGTAAGCCC----------CATACAGAGGATAGGCTGGTTCACTTGAAGAGAATCTTTTCTATGATCAGATCCGAATCATGTCGTACATGAGCAGGTTCCGTAAGATCCAGTAG-----AATAAGTG-AACTAGATAACCCATAATCGAGATTATGGGTTATCTAG-TTCACTTACATACGATTGAATAGTATGGAAATGCATTCATTTCCTATGCATTGACACGATCTATGATACTATCGGAGTGAAACAGGGGATCTAAGGAAGAACAGAGGCTAGGCTATATTAGTAACAAGTAAACCC-----TTTGTGTGTCTGTCAAAAGTCTCCAAGTATTTTGGGGATAAACACCGATCCTAAGGTCTGAGACGACCCAGAAAGCATT--TGATCCTATCATGATCCACCTTGTAAGCCTACTTGGGTATTGAGTATTTACTTGTAAGAACCAAATTTTTTGCGCTGGATAGTTGCAACTCCGGAAAAAGAAATCC------------------AGTCAAATTTTTC------TTTTAAT--------AAAATCATTCATATTATTATA-----TATGTGTGGATCTAGATAACATATAGATTTTATATGGATTCCTTATGGTTCTTTTTCTTTTTGCTCGAGCCGTATGATGAAAAATTATCATGTCCGGTTCCTTCGGGGGATGGAT-----CTATAAAAATTCACCTATCCCAATAACAAAAAAACCTGACCTGAATGATCCTGTATTAAGAGCTAAATTGGCTAAAGGTATGGGTCATAATTATTACGGAGAGCCCGCATGGCCCAATGATCTTTTATATATTTTTCCAGTAGAGATTG-----------------------AAG-----AAATCAATGCAATATTTTTTAGGAAAGATAAAACTGGATGAATTCAAATTCGTTCAAATGGG-----------------------ATTGGAAGGTTCCTTATTTTCATTTAGGGTGTTCGTTTTATTTCTTTTCTTAGGACTTTGGTGTAGTTTATGCTCTCTCTCCTGGAATCG-AATTGTTGT-AACTGGACGCTTCTATCCTACTAGCTAGTA--GGGATAGAAC-------AAAAAATATTTTCA------TTTTTTAATGAATTCTTTCTCATTTATCCGATTTATCAAATTTGAAACAAAAAGATACATTGTTTCAATAAACAC-----------AAAAAATCCTAAAGTT---------ATACTATACAAAAGGTTGTCAAAATGGAATCAATTAGTTTCAACAATTCCTTT-----------------------ATTTTTACTAATGATCTTACA----TATGCCC-------TTCTATAGATATATATAGAA----------------ATAGAGAAACCC----------------------------ATTTTTCT-------TATTATA-----GATAAATAGGTTGATGGGGAAAATAAGACCCCGC-------CCTCGAAATGATAAAATCTACTAAAAAGAAAGGTAAAACCTTGTATCTTGTCTTTATTC---------------TTTTTTCAAAAAAA--------TTCTTTTTAAAATTTAGAAATTTAGTAAACAGAAGCATTTTTATTCTACTTCCATTCCCTATTCATT--GGCCGAAAACAAAACAATAGGGAATGGGAATTATTCATTTTATTTTTAGATTAACAATGAAATCAGAT---------AGTCAAATCAATTGGGATTATTCTAACGTTTTATGA--------CTTATTTGTTTGTCGTACAAAAAAACTTTTTACATTCCCGGTAGGTCGTACAAAAAAAC-TTTTTGAATTCCCGGT-ACAAAGGCCATCTCCCCCAATTGAACAAGAGAATAATGACTATGTTACATTACACATGAAGTAA-----GAAAAAAGTC--TTG-CTTTCTCTTTCTTTATGATATAG-------ATATGTACAACTTTGACCAGCAATTTCATTTAGATC-GAAGTAAGGGCTCGAAAGAT-CCAATAGACAAAT-------------ATAAAGAAAAATAAAGAAGACCC-----TTTTGATTTTGTTCCCTTTATTCCCACGGCCTGGCCTGGTCAATACCTAGCCGGGCC----TTTTTTTGTTCCAACAAATCCTAGCTAAAAGAATTTAGCTG-------------CTTTGAACACA---------------AAAATGCTTGCTATTAAAGCAGC-------------------AATAAAAAGATGAGGGGTTATTTCCATTCTTACTTATTATTCCAT------------------TCTTACTTATTATATA--TATTATTTATATTTATATATATATTAT--TTATATTTATATAAATTATATAAAATCAAAGTATCCTTTCTTATT-----ATTCCTTCTTCCCTTTTGAGTTACTTGACGACCTTATGGGAAT--ATAAAATGAAACTG-----TGGGTTCTTA---AATAATAATGAATGCATTTTTCTGTTATGATTTCAGTGGTTTTAGTGAGCCATATCTATCAAAATCCCCCCAGCAAAAGAAAAAATAGAACTT--GTTATTTC----------------ATTTAGTTATTTAAAAGAGCCC-----TCCTTTCCGGAATCTCATTAAATTGAAATCCCCCGCGAA-AAACGT-CGACACTCTCATTTTCATGA-----TTATGATC-CTA--T-CTTTATTACGCCTAATTCCTCTGTT-CGACAAAAAGTTCATTTGTATATAATAAGTATTAG-TATATCTAATACCTTACCCAGCCCATTTGGAAATTTTGGTTCAAACTCTTCGCTACTGGGTAAAAGATGCCCCCTCTTTGCATTTATTACGATTCTTTCTTCACGACTATCGGCATTGGACTAGTCTTTTTATTCCAAAGAAAGCCAGTTCTTTTTTTTCAAAACGAAAG------------------CAAAGATTATTCTTCTTCCTATATAATTCTCATGTATGTGAATACGAATCCATCTTCGTCTTTCTCCGTAACCAATCTTCTCATTTACAATCAACATCTTCTGGAGCCCTTTTTGAACGAATATATTTCTATGAAAAAATAGAACATCTTGTCGAAGTCTTTGCTAAA-GATTTTCAAGGCAATCTATGGTTGTTCAAGGATCCTTTCATGCATTATGTTAGGTATCAAGGAAAGTCAATTCTCGCTTCAA-GGAGAACTTTCTTTTGATGCGTAAATGGAAATATTACTTTGTACGTTTCTGGCAATGTCATTTTTACCAGTGGTTTCAACCAGGAAGGATCTATATAAACCAATTATCCAAACATTCCCTCGACCTTCTGGGCTATCTTTCAAGTGTGCGGCTAAACCCTTTAACGATACGCAGTCAAATGCTAGAAAATTCACTTCTAATTGATAATGCTGTTAAGAAGTTCGATACCATTGTTCCAATTATTCCTCTGATTGGATCATTGGTTAAAGCGAAATTTTGTAACGTATTAGGGCATCCTGTTAGTAAGGTAGTTTGGGCAGATTTATCAGATTTAAGACAT-AAAATTTACCCTACTTCTTTATCTTTT-TAGGAAGGGCCTTTCATGAGTTGAATAGAGATTTTCA-TTTTTTATTCATCATTCGGGTTGATGAACTAAA-CCAGATAGTTATATGAGTGAAAGAAACAGCTTATAAATTTGCAGTAAAAAGATTGAGTCTCATTTT-CTATGTAC-AAGAG-TTAAGTGAAAGTAACCATAAACATTA-GAAACGGTTTACCCCAAGATTGGTTAATTAGTGATCATGGCTTGAAGCGGGTGC-AAAAGATCAACTGTATGGGG-TTTTTACTATCTATTACCATACATGTATTACCCTAACGGGCGATTAGCAAAAAGAGGTGGATAGTTAGGAACACCAAGGTACACAAAGGATTCGTAATAGAGATTATGTAAGTTATTCAACAGAATTTTTCTGTGCATAA-AAGGAATTCTGATTGGGACTTTAAGTTGGTAGAAATGATGAAGAAGTACTCCCCCTGATTCCGATCCAGAGTATACTCCTATCCACCGATTAAGTAAATAACTATCAAGAACGAAGTAATCCTTT-ACTTT------GTTTAAAGTCCCTTTTTCTGAGAAAGGAGAATAGGAACGAAAAAAATC-------AAATAG-AAAGA-ATAGAATTGCACTAG-----AAAGAAAGAGATCT-----------TTCTTTCC-------------TCTATTTAGAGAGATAGAATTCTTGTCATCATTCGTGAACTAATGCGATGCCTAATTGTTTTTCGTAATCGAAAATGCTAGGTTG--------AAATATCTATGAATA-TTGCTACAAGAAAGA-TTTTATTGAAAG-CTTAAGTTATCACTCAAC-AAAGAAAAATAAAAA-TTATT-----AAAAGATAAGATCAA-TTCCG-AAGCACTTTA-TTTTCAATATAGCAGACAGAATTCCATTGTCTAATTCGGG--ACTTTACGGTAGATTTTGATTCTATCTATCCTACGAATATAT-------------------CAAGATAAATAATAGCGAACTGGTCCTTAGA-TTTATTTGTGA-CCTTTGAGGAGCC-GTATGAGATGAAAATCTCATGTACGGTATTTAGATTA---------------------------------------------------------------------------------------------------------------------------------------------------------------------------------ATAAA------AATATTAACGATTTTTCTTT-AA-----------AGAAA--ATATGTAAGTCAA--------------------------------------------------------------------------------TGTGAAAT-AAAAAAGGAGCAATA-TCCCCTCCTTGTTCTATCAAG-AGGGGGATATTGCTCCTTTTTT-ATTTCGTTCAAATACTCGTATACACTAAGGCCGGGTCTTATCCATTTATAGATGGAGCTTCAAGAGCAGCTAGGTCTAGAGGGAAGTTATGAGCATTACGTTCATGCATAACTTCCATACCAAGGTTAGCGCGGTTAATGATATCAGCCCAAGTATTAATTACACGACCTTGACTATCAACTACAGATTGGTTGAAATTAAAACCATTTAGGTTGAAAGCCATAGTGCTGATACCTAAAGCAGTGAACCAGATACCTACTACAGGCCAAGCAGCTAGGAAGAAATGTAAAGAACGAGAGTTGTTGAAACTAGCATATTGGAAGATCAATCGGCCAAAATAACCATGAGCAGCTACAATATTATAAGTTTCTTCTTCTTGA-CCGAATC---TGTAACCTTCATTAGCAATAAATGCAAGAATATTTAC-TTCCATAATCTTATCGTTTTTTTACTTCAAAATAACT-CGGGATTTAATCCCATAGAGATAATAAATCTTTCGCCTGTCAATTCA----ATGAATTACCTCTCGATGATC-TTGAAATCGGATCAATATCATGAATAACAATATCTGAGCTATCAAATCAATTCGTCGTCGAGAATTGAATAGTATAACATAGAAAGATCTTTGTATCCATACCGAATCCAAATCCAA------------------------AATTTCTTTATTTATCAATCATTCTTTTCTGTTCTTTCTTTATCTACAACCTATCTTAGGTCCTCCTTGTACAATCATCGGATAAAGTATCGTCTGACCGCCCGTCCGTTTCCATTAGTCACAAACGCCCAACAAACAATAGAAGGGAAGTGGAAAAAGAAATAAGTTACGTTCTAAACTCCG----TTTTTTTAATGATCTAGTTTTCTTGGAAGACAAAGAAGTGTGATAAAGAGGAGTTCCGGGATAAAGGATGTAATATTCCATCAAACTAACTATTTGAGTTTGGGTTTTGTTCGTTTTTCGACGGG--CCCT-------AAAAAAAAAATAG--AAAAAAATAGGAAGGAAAAAT-GATTTATTCCCCTGCTACTTGCTAAGCTAAAAAAGGGGTGGGATCTTTGATTGATCTTTATTTTTCTTTTA-CCCCCCCTTCCTT---------------------------------------------------------------------------------------------------------------------------------------------------------------------------------------------------------------------------------------------------------------------------------------------------------------AACGCTCTCAATAATTGTACTATTCTACATATGTCTTTCTCCTACCAATCAGTATTATTTGAAATAATGAAAATTCCCCTATTTGTTTGATGAGAAGTGC----GAAATGCCAAAGGAAA--GAAAAAAGAACCCCCTT-GGGAATG-AAATTCTGCTCCCCGTGCCCCCTTTAACAGAAAAGGGAAGATTACAAATTGACTTATTATACTCCTGACTATGAAACCAAAGATACTGATATCTTGGCAGCATT-CCGAGTAACTCCTCAACCTGGAGTTCCACCTGAAGAAGCAGGGGCCGCGGTAGCTGCCGAATCTTCAACTGGTACATGGACAACTGTGTGGACCGATGGACTTACCAGCCTTGATCGTTACAAAGGGCGATGCTACCACATCGAGCCCGTTGCTGGAGAAGAAACTCAATTTATTGCTTATGTAGCTTACCCATTAGACCTTTTTGAAGAAGGTTCTGTTACTAACATGTTTACTTCTATTGTGGGTAATGTATTTGGGTTCAAAGCCCTGCGCGCTCTACGTCTGGAAGATCTGCGAATCCCTGTCGCTTATGCTAAAACTTTCCAAGGCCCGCCTCATGGCATCCAAGTTGAGAGAGATAAATTGAACAAGTATGGTCGCCCCCTGTTGGGATGTACTATTAAACCTAAATTGGGGTTATCTGCTAAAAACTATGGTAGAGCGGTTTATGAATGTCTACGTGGTGGACTTGATTTTACCAAAGATGATGAGAA--TGT--GAACTCCCAACCATTTATGCGTT---GGAGAGATCGATTC

Diabelia_spathulata_BOP012317 CCTGAGCCAAATCCAGTTTTACGAAAACAAACAAGGGTTCAGAAAGCTAAAATC-AAAACGGATAGGTGCAGAGACTCAATGGAAGCTGTTCTAACAAATGGAGTTGACTGTGTTGTGTTGGTAGAAAGAATCCTTCCATAGAAACTTCAGAAAGGATAAACGTATAAACATAGATATACGCATTGAAATACTAT-ATAATCTACCAAATGATTAATGACGACGCGAATCTGTATT--TA-----TATATATCAAAATGGGAGAATGGTTGTGAAGTGATTCCATATTGAAGAAAGAATCGAATATTTATTGATCAAATCATTCACTCCATAGTCTGATAGATC-TTTTGAAGAACTGATTAATTGGACGAGAATAAAGATAGAGTCCCATTCTACATGTCAATACCGGCAACAATGAAATTTATAGTAAGAGGAAAATCCGTCGACTTTAGAAATCGTGAGGGTTCAAGTCCCTCTATCCCCAAAAAAACCATATGGACTCCCTAATTATTTATCCTCCCCTT-TTATCC-TTTTTTGTTAGCGGTTAAAAATTCG------TTATCTTTCTCATTCACCCTACTCTTTTACAAAGAGATCTGAGCGGAAATGTTTTTCTCTTATC----ACAAGTCTTGTGATCTAAG----ATAA-TACGTGTACAAATGAACATCTTTGAGTAAGGAATCCCCATTTAAATGATTCATGGTCAA------TGTCATTATTCATACTGAAACTTACAAAGTCTTCCTT-TTGAAGATCCAAGAAATTCCAGGACCTGGATAAGACTTTGTAAGACCCTTTCAATTGACA-TAGACCCGCGTTATCTAGC--AAAATGAGGATGCAGCGGTATTTATGTTAATGCACTTCCCAATGATACGTAAACAAGGCATTTCTGGTCCTTTA--------TAGAGAAGATCTATCATAGATATTTGTAATCTATCATTTATCGCTTGGGGCAGGAACAATAGTATTTCATTGCTACAAGTATGGATTATTGAGAATAATAAGACATGTATTTGGATATTTCCCTTGAACTCCGCAATCTTTTTGATTTGACATGGATAGTTGAAGGAAATTTTCCGAAGAAAAAATGGATTAGATTATGGGAGTGTGTGACTTGAACTATTGATTGGTCTGTGCAGATATATGCCTTTCGCTATCTGCCACATTGCAATTCACAACCAAATGTGTCTTTGTTCCAACCATTGCGTAAGCCC----------CATACAGAGGATAGGCTGGTTCACTTGAAGAGAATCTTTTCTATGATCAGATCCGAATCATGTCGTACATGAGCAGGTTCCGTAAGATCCAGTAG-----AATAAGTG-AACTAGATAACCCATAATCGAGATTATGGGTTATCTAG-TTCACTTACATACGATTGAATAGTATGGAAATGCATTCATTTCCTATGCATTGACACGATCTATGATACTATCGGAGTGAAACAGGGGATCTAAGGAAGAACAGAGGCTAGGCTATATTAGTAACAAGTAAACCCTTTGTTTTGTGTGTCTGTCAAAAGTCTCCAAGTATTTTGGGGATAAACACCGATCCTAAGGTCTGAGACGACCCAGAAAGCATT--TGATCCTATCATGATCCACCTTGTAAGCCTACTTGGGTATTGAGTATTTACTTGTAAGAACCAAATTTTTTGCGCTGGATAGTTGCAACTCCGGAAAAAGAAATCC------------------AGTCAAATTTTTC------TTTTAAT--------AAAATCATTCATATTATCATA-----TATGTGTGGATCTAGATAACATATAGATTTTATATGGATTCCTTATGGTTCTTTTTCTTTTTGCTCGAGCCGTATGATGAAAAATTATCATGTCCGGTTCCTTCGGGGGATGGAT-----CTATAAAAATTCACCTATCCCAATAACAAAAAAACCTGACCTGAATGATCCTGTATTAAGAGCTAAATTGGCTAAAGGTATGGGTCATAATTATTACGGAGAGCCCGCATGGCCCAATGATCTTTTATATATTTTTCCAGTAGAGATTGAAGAAATCAATGCAATATTTTTTAAG-----AAATCAATGCAATATTTTTTAGGAAAGATAAAACTGGATGAATTCAAATTCGTTCAAATGGG-----------------------ATTGGAAGGTTCCTTATTTTCATTTAGGGTGTTCGTTTTATTTCTTTTCTTAGGACTTTGGTGTAGTTTATGCTCTCTCTCCTGGAATCG-AATTGTTGT-AACTGGACGCTTCTATCCT-CTAGCTAGTA--GGGATAGAAC-------AAAAAATATTTTCA------TTTTTTAATGAATTCTTTCTCATTTATCCGATTTATCAAATTTGAAACAAAAAGATACATTTTTTCAATAAACAC-----------AAAAAATCCTAAAGTT---------ATACTATACAAAAGGTTGTCAAAATGGAATCAATTAGTTTCACCAATTCCTTT-----------------------ATTTTTACTAATGATCTTACA----TATGCCC-------TTCTATAGATATATATAGAA----------------ATAGAGAAACCC----------------------------ATTTTTCT-------TATTATA-----GATAAATAGGTTGATGGGGAAAATAAGACCCCGC-------CCTCGAAATGATAAAATCTACTAAAAAGAAAGGTAAAACCTTGTATCTTGTCTTTATTC---------------TTTTTTCAAAAATT--------TTTTTTTTTAAATTTAGAAATTTAGTAAACAGAAGCATTTTTATTCTACTTCCATTCCCTATTCATT--GGCCGA-----AAACAATAGGGAATGGGAATTATTCATTTTATTTTTAGATTAACAATGAAATCAGAT---------AATCAAATCAATTGGGATTATTCTAACGTTTTATGA--------CTTATTTATTTGTCGTACAAAAAAACTTTTTACATTCCCGGTAGGTTGTACAAAAAAAC-TTTTTGAATTCCCGGT-ACAAAGGCCATCTCCCCCAATTGAACAAGAGAATAATGACTATGTTACATTACACATGAAGTAA-----GAAAAAAGTC--TTG-CTTTCTCTTTCTTTATGATATAG-------ATATGTACAACTTTGACCAGCAATTTCATTTAGATC-TAAGTAAGGGCTCGAAAGAT-CCAATAGACAAAT-------------ATAAAGAAAAATAAAGAAGACCC-----TTTTGATTTTGTTCCCTTTATTCCCACGGCCTGGCCTGGTCAATACCTAGCCGGGCC----TTTTTTTGTTCCAACAAATCCTAGCTAAAAGAATTTAGCTG-------------CTTTGAACACA---------------AAAATGCTTGCTATTAAAGCAGC-------------------AATAAAAAGATGAGGGGTTATTTCCATTCTTACTTATTATTCCAT------------------TCTTACTTATTATATA--TAT-----------------------------TATTTATATAAATTATATAAAATCAAAGTATCCTTTCTTATT-----ATTCCTTCTTCCCTTTTGAGTTACTTGACGACCTTATGGGAAT--ATAAAATGAAACTG-----TGGGTTCTTA---AATAATAATGAATGCATTTTTCTGTTATGATTTCAGTGGTTTTAGTGAGCCATATCTATCAAAATCCCCCCAGCAAAAGAAAAAATAGAACTT--GTTATTTC----------------ATTTAGTTATTTAAAAGAGCCC-----TCCTTTCCGGAATCTCATTAAATTGAAATCCCCCGCGAA-AAACGT-CGACACTCTCATTTTCATGA-----TTATGATC-CTA--T-CTTTATTATGCCTAATTCCTCTGTT-CGACAAAAAGTTCATTTGTATATAATAAGTATTAG-TATATCTAATACCTTACCCAGCCCATTTGGAAATTTTGGTTCAAACTCTTCGCTACTGGGTAAAAGATGCCCCCTCTTTGCATTTATTACGATTCTTTCTTCACGACTATCGGCATTGGACTAGTCTTTTTATTCCAAAGAAAGCCAGTTCTTTTTTTTCAAAACGAAAG------------------CAAAGATTATTCTTCTTCCTATATAATTCTCATGTATGTGAATACGAATCCATCTTCGTCTTTCTCCGTAACCAATCTTCTCATTTACAATCAACATCTTCTGGAGCCCTTCTTGAACGAATATATTTCTATGAAAAAATAGAACATCTTGTCGAAGTCTTTGCTAAA-GATTTTCAAGGCAATCTATGGTTGTTCAAGGATCCTTTCATGCATTATGTTAGGTATCAAGGAAAGTCAATTCTCGCTTCAAAGGAGAACTTTCTTTTGATGCGTAAATGGAAATATTACTTTGTACGTTTCTGGCAATGTCATTTTTACCAGTGGTTTCAACCAGGAAGGATCTATATAAACAAATTATCCAAACATTCCCTCGACATTCTGGGCTATCTTTCAAGTGTGCGGCTAAACCCTTTAACGATACGCAGTCAAATGCTAGAAAATTCACTTCTAATTGATAATGCTGTTAAGAAGTTCGATACCATTGTTCCAATTATTCCTCTGATTGGATCATTGGTTAAAGCGAAATTTTGTAACGTATTAGGGCATCCTGTTAGTAAGGTAGTTTGGGCAGATTTATCAGATTTAAGACAT-AAAATTTACCCTACTTCTTTATCTTTC-TAGGAAGGGCCTTTCATGAGTTGAATAGAGATTTTCA-TTTTTTATTCATCATTCGGGTTGATGAACTAAA-CCAGATAGTTATATGAGTGAAAGAAACAGCTTATAAATTTGCAGTAAAAAGATTGAGTCTCATTTT-CTATGTAC-AAGAG-TTAAGTGAAAGTAACCATAAACATTA-GAAACGGTTTACCCCAAGATTGGTTAATTAGTGATCATGGCTTGAAGCGGGTGC-AAAAGATCAACTGTATGGGG-TTTTTACTATCTATTACCATACATGTATTACCCTAACGGGCGATTAGCAAAAAGAGGTGGATAGTTAGGAACACCAAGGTACACAAAGGATTCGTAATAGAGATTATGTAAGTTATTCAACAGAATTTTTCTGTGCATAA-AAGGAATTCTGATTGGGACTTTAAGTTGGTAGAAATGATGAAGAAGTACTCCCCCTGATTCCGATCCAGAGTATACTCCTATCCACCGATTAAATAAATAACTATCAAGAACGAAGTAATCCTTT-ACTTT------GTTTAAAGTCCCTTTTTCTGAGAAAGGAGAATAGGAACGAAAAAAATA-------AAATAG-AAAGA-ATAGAATTGCACTAG-----AAAGAAAGAGATCT-----------TTCTTTCC-------------TCTATTTAGAGAGATAGAATTCTTGTCATCATTCGTGAACTAATGCGATGCCTAATTGTTTTTCGTAATCGAAAATGCTAGGTTG--------AAATATCTATGAATA-TTGCTACAAGAAAGA-TTTTATTGAAAG-CTTAAGTTATCACTCAAC-AAAGAAAAATAAAAA-TTATT-----AAAAGATAAGATCAA-TTCCG-AAGCACTTTA-TTTTCAATATAGCAGACAGAATTCCATTGTCTAATTCGGG--ACTTTACGGTAGATTTTGATTCTATCTATCCTACGAATATAT-------------------CAAGATAAATAATAGCGAACTGGTCCTTAGA-TTTATTTGTGA-CCTTTGAGGAGCC-GTATGAGATGAAAATCTCATGTACGGTATTTAGATTC---------------------------------------------------------------------------------------------------------------------------------------------------------------------------------ATAAA-----------TAACGATTTTTCTTT-AA-----------AGAAA--ATATGTAAGTCAA--------------------------------------------------------------------------------TGTGAAAT-AAAAAAGGAGCAATA-CCCCCTCCTTGTTCTATCAAG-AGGGGGATATTGCTCCTTTTTT-ATTTCTTTCAAATACTCGTATACACTAAGGCCGGGTCTTATCCATTTATAGATGGAGCTTCAAGAGCAGCTAGGTCTAGAGGGAAGTTATGAGCATTACGTTCATGCATAACTTCCATACCAAGGTTAGCGCGGTTAATGATATCAGCCCAAGTATTAATTACACGACCTTGACTATCAACTACAGATTGGTTGAAATTAAAACCATTTAGGTTGAAAGCCATAGTGCTGATACCTAAAGCAGTGAACCARATACCTACTACAGGCCAAGCAGCTAGGAAGAAATGTAAAGAACGAGAGTTGTTGAAACTAGCATATTGGAAGATCAATCGGCCAAAATAACCATGAGCAGCTACAATATTATAAGTTTCTTCTTCTTGACCCGAATCCCTTGTAACCTTCATTAGCAATAAATGCAAGAATATTTAC-TTCCATAATCTCATCGTTTTTTTACTTCAAAATAACT-CGGGATTTAATCCCATAGAGATAATAAATCTTTCGCCTGTCAATTCA----ATGAATTACCTCTCGATGATC-TTGAAATCGGATCAATATCATGAATAACAATATCTGAGCTATCAAATCAATTCGTCGTCGAGAATTGAATAGTATAACATAGAAAGATCTTTGTATCCATACCGAATCCAAATCCAA------------------------AATTTCTTTATTTATCAATCATTCTTTTCTGTTCTTTCTTTATCTACAACCTATCTTAGGTCCTCCTTGTACAATCATCGGATAAAGTATCGTCTGACCGCCCGTCCGTTTCCATTAGTCACAAACGCCCAACAAACAATAGAAGCGAAGTGGAAAAAGAAATAAGTTACGTTCTAAACTCCG----TTTTTTTAATGATCTAGTTTTCTTGGAAGACAAAGAAGTGTGATAAAGAGGAGTTCCGGGATAAAGGATGTAATATTCCATCAAACTAACTATTTGAGTTTGGGTTTTGTTCGTTCTTCGACGGG--CCCT-------AAAAAAAAAATAG--AAAAAAATAGGAAGGAAAAAT-GATTTATTCCCCTGCTACTTGCTAAGCTAAAAAAGGGGTGGGATCTTTGATTGATCTTTATTTTTCTTTTA-CCCCCCCTTCCTT---------------------------------------------------------------------------------------------------------------------------------------------------------------------------------------------------------------------------------------------------------------------------------------------------------------AACGCTCTCAATAATTGTACTATTCTACATATGTCTTTCTCCTACCAATCAGTATTATTTGAAATAATGAAAATTCCCCTATTTGTTTGATGAGAAGTGC----GAAATGCCAAAGGAAA--GAAAAAAGAACCCCCTT-GGGAATG-AAATTCTGCTCCCCGTGCCCCCTTTAACAGAAAAGGG-------------------------------------------------------------------------------------------------------------GGGCCGCGGTAGCTGCCGAATCTTCAACTGGTACATGGACAACTGTGTGGACCGATGGACTTACCAGCCTTGATCGTTACAAAGGGCGATGCTACGGCATCGAGCCCGTTGCTGGAGAAGAAAATCAATATATTGCTTATGTAGCTTACCCATTAGACCTTTTTGAAGAAGGTTCTGTTACTAACATGTTTACTTCGATTGTGGGTAATGTATTTGGGTTCAAAGCCCTGCGCGCTCTACGTCTGGAAGATCTGCGAATCCCTGTCGCTTATGTTAAAACTTTCCAAGGCCCGCCTCATGGCATCCAAGTTGAGAGAGATAAATTGAACAAGTATGGTCGCCCCCTGTTGGGATGTACTATTAAACCTAAATTGGGGTTATCTGCTAAAAACTATGGTAGAGCGGTTTATGAATGTCTACGTGGTGGACTTGATTTTACCAAAGATGATGAGAA--CGT--GAACTCCCAACCATTTATGCGTT---GGAGAGATCGTTTC

Diabelia_tetrasepala_BOP012270 CCTGAGCCAAATCCAGTTTTACGAAAACAAACAAGGGTTCAGAAAGCTAAAATC-AAAACGGATAGGTGCAGAGACTCAATGGAAGCTGTTCTAACAAATGGAGTTGACTGTGTTGTGTTGGTAGAAAGAATCCTTCCATAGAAACTTCAGAAAGGATAAACGTATAAACATAGATATACGCATTGAAATACTAT-ATAATCTACCAAATGATTAATGACGACGCGAATCTGTATT--TA-----TATATATCAAAATGGGAGAATGGTTGTGAAGTGATTCCATATTGAAGAAGGAATCGAATATTTATTGATCAAATCATTCACTCCATAGTCTGATAGATC-TTTTGAAGAACTGATTAATTGGACGAGAATAAAGATAGAGTCCCATTCTACATGTCAATACCGGCAACAATGAAATTTATAGTAAGAGGAAAATCCGTCGAC-TTAGAAATCGTGAGG--TCAAGT-CCTCTATCCCAAAAA----CATATGGACTCCCTAATTATTTATCCTCCCCTT-TTATCC-TTTTTTGTTAGCGGTTAAAAATTCG------TTATCTTTCTCATTCACCCTACTCTTTTACAAAGAGATCTGAGCGGAAATGTTTTTCTCTTATC----ACAAGTCTTGTGATCTAAG----ATAA-TACGTGTACAAATGAACATCTTTGAGTAAGGAATCCCCATTTAAATGATTCATGGTCAA------TGTCATTATTCATACTGAAACTTACAAAGTCTTCCTT-TTGAAGATCCAAGAAATTCCAGGACCTGGATAAGACTTTGTAAGACCCTTTCAATTGACA-TAGACCCGCGTTATCTAGC--AAAATGAGGATGCAGCGGTATTTATGTTAATGCACTTCCCAATGATACGTAAACAAGGCATTTCTGGTCCTTTA--------TAGAGAAGATCTATCATAGATATTTGTAATCTATCATTTATCGCTTGGGGGAGGAACAATAGTATTTCATTGCTACAAGTATGGATTATTGAGAATAATAAGACATGTATTTGGATATTTCCCTTGAACTCCGCAATCTTTTTGATTTGACATGGATAGTTGAAGGGAATTTTCCGAAGAAAAAATGGATTAGATTATGGGAGTGTGTGACTTGAACTATTGATTGGTCTGTGCAGATATATGCCTTTCGCTATCTGCCACATTGCAATTCACAACCAAATGTGTCTTTGTTCCAACCATTGCGTAAGCCC----------CATACAGAGGATAGGCTGGTTCACTTGAAGAGAATCTTTTCTATGATCAGATCCGAATCATGTCGTACATGAGCAGGTTCCGTAAGATCCAGTAG-----AATAAGTG-AACTAGATAACCCATAATCGAGATTATGGGTTATCTAG-TTCACTTACATACGATTGAATAGTATGGAAATGCATTCATTTCCTATGCATTGACACGATCTATGATACTATCGGAGTGAAACAGGGGATCTAAGGAAGAACAGAGGCTAGGCTATATTAGTAACAAGTAAACCC-----TTTGTGTGTCTGTCAAAAGTCTCCAAGTATTTTGGGGATAAACACCGATCCTAAGGTCTGAGACGACCCAGAAAGCATT--TGATCCTATCATGATCCACCTTGTAAGCCTACTTGGGTATTGAGTATTTACTTGTAAGAACCAAATTTTTTGCGCTGGATAGTTGCAACTCCGGAAAAAGAAATCC------------------AGTCAAATTTTTC------TTTTAAT--------AAAATCATTCATATTATTATA-----TATGTGTGGATCTAGATAACATATAGATTTTATATGGATTCCTTATGGTTCTTTTTCTTTTTGCTCGAGCCGTATGATGAAAAATTATCATGTCCGGTTCCTTCGGGGGATGGAT-----CTATAAAAATTCACCTATCCCAATAACAAAAAAACCTGACCTGAATGATCCTGTATTAAGAGCTAAATTGGCTAAAGGTATGGGTCATAATTATTACGGAGAGCCCGCATGGCCCAATGATCTTTTATATATTTTTCCAGTAGAGATTG-----------------------AAG-----AAATCAATGCAATATTTTTTAGGAAAGATAAAACTGGATGAATTCAAATTCGTTCAAATGGG-----------------------ATTGGAAGGTTCCTTATTTTCATTTAGGGTGTTCGTTTTATTTCTTTTCTTAGGACTTTGGTGTAGTTTATGCTCTCTCTCCTGGAATCG-AATTGTTGT-AACTGGACGCTTCTATCCTACTAGCTAGTA--GGGATAGAAC-------AAAAAATATTTTCA------TTTTTTAATGAATTCTTTCTCATTTATCCGATTTATCAAATTTGAAACAAAAAGATACATTGTTTCAATAAACAC-----------AAAAAATCCTAAAGTT---------ATACTATACAAAAGGTTGTCAAAATGGAATCAATTAGTTTCAACAATTCCTTT-----------------------ATTTTTACTAATGATCTTACA----TATGCCC-------TTCTATAGATATATATAGAA----------------ATAGAAAAACCC----------------------------ATTTTTCT-------TATTATA-----GATAAATAGGTTGATGGGGAAAATAAGACCCCGC-------CCTCGAAATGATAAAATCTACTAAAAAGAAAGGTAAAACCTTGTATCTTGTCTTTATTC---------------TTTTTTCAAAA-----------TTTTTTTTTTAATTTAGAAATTTAGTAAACAGAAGCATTTTTATTCTACTTCCATTCCCTATTCATT--GGCCGAAAACAAAACAATAGGGAATGGGAATTATTCATTTTATTTTTAGATTAACAATGAAATCAGAT---------AGTCAAATCAATTGGGATTATTCTAACGTTTTATGA--------CTTATTTGTTTGTCGTACAAAAAAACTTTTTACATTCCCGGTAGGTCGTACAAAAAAAC-TTTTTGAATTCCCGGT-ACAAAGGCCATCTCCCCCAATTGAACAAGAGAATAATGACTATGTTACATTACACATGAAGTAA-----GAAAAAAGTC--TTG-CTTTCTCTTTCTTTATGATATAG-------ATATGTACAACTTTGACCAGCAATTTCATTTAGATC-TAAGTAAGGGCTCGAAAGAT-CCAATAGACAAAT-------------ATAAAGAAAAATAAAGAAGGCCC-----TTTTGATTTTGTTCCCTTTATTCCCACGGCCTGGCCTGGTCAATACCTAGCCGGGCC----TTTTTTTGTTCCAACAAATCCTAGCTAAAAGAATTTAGCTG-------------CTTTGAACACA---------------AAAATGCTTGCTATTAAAGCAGC-------------------AATAAAAAGATGAGGGGTTATTTCCATTCTTACTTATTATTCCATTCTTACTTATTATATATATCTTACTTATTATATA--TAT-----------------------------TATTTATATAAATTATATAAAATCAAAGTATCCTTTCTTATT-----ATTCCTTCTTCCCTTTTGAGTTACTTGACGACCTTATGGGAAT--ATAAAATGAAACTG-----TGGGTTCTTA---AATAATAATGAATGCATTTTTCTGTTATGATTTCAGTGGTTTTAGTGAGCCATATCTATCAAAATCCCCCCAGCAAAAGAAAAAATAGAACTT--GTTATTTC----------------ATTTAGTTATTTAAAAGAGCCC-----TCCTTTCCGGAATCTCATTAAATTGAAATCCCCCGCGAA-AAACGT-CGACACTCTCATTTTCATGA-----TTATGATC-CTA--T-CTTTATTACGCCTAATTCCTCGGTT-CGACAAAAAGTTCATTTGTATATAATAAGTATTAG-TATATCTAATACCTTACCCAGCCCATTTGGAAATTTTGGTTCAAACTCTTCGCTACTGGGTAAAAGATGCCCCCTCTTTGCATTTATTACGATTCTTTCTTCACGACTATCGGCATTGGACTAGTCTTTTTATTCCAAAGAAAGCCAGTTCTTTTTTTTCAAAACGAAAG------------------CAAAGATTATTCTTCTTCCTATATAATTCTCATGTATGTGAATACGAATCCATCTTCGTCTTTCTCCGTAACCAATCTTCTCATTTACAATCAACATCTTCTGGAGCCCTTTTTGAACGAATATATTTCTATGAAAAAATAGAACATCTTGTCGAAGTCTTTGCTAAA-GATTTTCAAGGCAATCTATGGTTGTTCAAGGATCCTTTCATGCATTATGTTAGGTATCAAGGAAAGTCAATTCTCGCTTCAAAGGAGAACTTTCTTTTGATGCGTAAATGGAAATATTACTTTGTACGTTTCTGGCAATGTCATTTTTACCAGTGGTTTCAACCAGGAAGGATCTATATAAACCAATTATCCAAACATTCCCTCGACCTTCTGGGCTATCTTTCAAGTGTGCGGCTAAACCCTTTAACGATACGCAGTCAAATGCTAGAAAATTCACTTCTAATTGATAATGCTGTTAAGAAGTTCGATACCATTGTTCCAATTATTCCTCTGATTGGATCATTGGTTAAAGCGAAATTTTGTAACGTATTAGGGCATCCTGTTAGTAAGGTAGTTTGGGCAGATTTATCAGAT??????????????????????????????????????????????????????????????????????????????????????????????????????????????????????????????????????????????????????????????????????????????????????????????????????????????????????????????????????????????????????????????????????????????????????????????????????????????????????????????????????????????????????????????????????????????????????????????????????????????????????????????????????????????????????????????????????????????????????????????????????????????????????????????????????????????????????????????????????????????????????????????????????????????????????????????????????????????????????????????????????????????????????????????????????????????????????????????????????????????????????????????????????????????????????????????????????????????????????????????????????????????????????????????????????????????????????????????????????????????????????????????????????????????????????????????????????????????????????????????????????????????????????????????????????????????????????????????????????????????????????????????????????????????????????????????????????????????????????TATTTAGATTA---------------------------------------------------------------------------------------------------------------------------------------------------------------------------------AAAAA------AATATTAACGATTTTTCTTT-AA-----------AGAAA--ATATGTAAATCAA--------------------------------------------------------------------------------TGTGAAAT-AAAAAAGGAGCAATA-TCCCCTCCTTGTTCTATCAAG-AGGGGGATATTGCTCCTTTTTT-ATTTCGTTCAAATACTCGTATACACTAAGGCCGGGTCTTATCCATTTATAGATGGAGCTTCAAGAGCAGCTAGGTCTAGAGGGAAGTTATGAGCATTACGTTCATGCATAACTTCCATACCAAGGTTAGCGCGGTTAATGATATCAGCCCAAGTATTAATTACACGACCTTGACTATCAACTACAGATTGGTTGAAATTAAAACCATTTAGGTTGAAAGCCATAGTGCTGATACCTAAAGCAGTGAACCAGATACCTACTACAGGCCAAGCAGCTAGGAAGAAATGTAAAGAACGAGAGTTGTTGAAACTAGCATATTGGAAGATCAATCGGCCAAAATAACCATGAGCAGCTACAATATTATAAGTTTCTTCTTCTTGA-CCGAATC---TGTAACCTTCATTAGCAATAAATGCAAGAATATTTAC-TTCCATAATCTTATCGTTTTTTTACTTCAAAATAACT-CGGGATTTAATCCCATAGAGATAATAAATCTTTCGCCTGTCAATTCA----ATGAATTACCTCTCGATGATC-TTGAAATCGGATCAATATCATGAATAACAATATCTGAGCTATCAAATCAATTCGTCGTCGAGAATTGAATAGTATAACATAGAAAGATCTTTGTATCCATACCGAATCCAAATCCAA------------------------AATTTCTTTATTTATCAATCATTCTTTTCTGTTCTTTCTTTATCTACAACCTATCTTAGGTCCTCCTTGTACAATCATCGGATAAAGTATCGTCTGACCGCCCGTCCGTTTCCATTAGTCACAAACGCCCAACAAACAATAGAAGCGAAGTGGAAAAAGAAATAAGTTACGTTCTAAACTCCG----TTTTTTTAATGATCTAGTTTTCTTGGAAGACAAAGAAGTGTGATAAAGAGGAGTTCCGGGATAAAGGATGTAATATTCCATCAAACTAACTATTTGAGTTTGGGTTTTGTTCGTTCTTCGACGGG--CCCT-------AAAAAAAAAATAG--AAAAAAATAGGAAGGAAAAAT-GATTTATTCCCCTGCTACTTGCTAAGCTAAAAAAGGGGTGGGATCTTTGATTGATCTTTATTTTTCTTTTA-CCCCCCCTTCCTT---------------------------------------------------------------------------------------------------------------------------------------------------------------------------------------------------------------------------------------------------------------------------------------------------------------AACGCTCTCAATAATTGTACTATTCTACATATGTCTTTCTCCTACCAATCAGTATTATTTTAAATAATGAAAATTCCCCTATTTGTTTGATGAGAAGTGC----GAAATGCCAAAGGAAA--GAAAAAAGAACCCCCTT-GGGAATG-AAATTCTGCTCCCCGTGCCCCCTTTAACAGAAAAGGG------------------------------------------------------TCTTGGCAGCATT-CCGAGTAACTCCTCAACCTGGAGTTCCACCTGAAGAAGCAGGGGCCGCGGTAGCTGCCGAATCTTCAACTGGTACATGGACAACTGTGTGGACCGATGGACTTACCAGCCTTGATCGTTACAAAGGGCGATGCTACCACATCGAGCCCGTTGCTGGAGAAGAAAGTCAATTTATTGCTTATGTAGCTTACCCATTAGACCTTTTTGAAGAAGGTTCTGTTACTAACATGTTTACTTCTATTGTGGGTAATGTATTTGGGTTCAAAGCCCTGCGCGCTCTACGTCTGGAAGATCTGCGAATTCCTGTCGCTTATGTTAAAACTTTCCAAGGCCCGCCTCATGGCATCCAAGTTGAGAGAGATAAATTGAACAAGTATGGTCGCCCCCTGTTGGGATGTACTATTAAACCTAAATTGGGGTTATCTGCTAAAAACTATGGTAGAGCGGTTTATGAATGTCTACGTGGTGGACTTGATTTTACCAAAGATGATGAGAA--CGT--GAACTCCCAACCATTTATGCGTT---GGAGAGATCGTTTC

Dipelta_elegans CCTGAGCCAAATCCAGTTTTACGAAAACAAACAAGGGTTCAGAAAGCTAAAATC-AAAACGGATAGGTGCAGAGACTCAATGGAAGCTGTTCTAACAAATGGAGTTGACTGTGTTGTGTTGGTAGAAAGAATCCTTCCATAGAAACTTCAGAAAGGATAAACGTATAAACATAGATATACGCATTGAAATACTATAATAATCTACCAAATGATTAATGACGACGCGAATCTGTATTTATA-----TATATATCAAAATGGGAGAATGGTTGTGAAGTGATTCCATATTGAAGAAAGAATCGAATATTTATTGATCAAATCATTCACTCCATAGTCTGATAGATC-TTTTGAAGAACTGATTAATTGGACGAGAATAAAGATAGAGTCCCATTCTACATGTCAATACCGGCAACAATGAAATTTATAGTAAGAGGAAAATCCGTCGACTTTTGAAATCGTGAGGGTTCAAGTCCCTCTATCCCCAAAAAACCCATATGGACTCCCTAATTATTTATCCTCTCCTT-TTATCC-TTTTTTGTTAGCGGTTATAAATTCG------TTATCTTTCTCATTCACCCTACTCTTTTACAAAGAGATCTGAGCGGAAATGTTTTTCTCTTATC----ACAAGTCTTGTGATCTAAG----ATAA-TACGTGTACAAATGAACATCTTTGAGTAAGGAATCCCCATTTAAATGATTCATGGTCAA------TGTCATTATTCATACTGAAACTTACAAAGTCTTCCTT-TTGAAGATCCAAGAAATTCCAGGACCTGGATAAGACTTTGTAAGACCCTTTCAATTGACA-TAGACCCGCGTTATCTAGC--AAAATGAGGATGCA?????????????????????????????????????????????????????????????????????????????????????????????????????????????????????????????????????????????????????????????????????????????????????????????????????????????????????????????????????????????????????????????????????????????????????????????????????????????????????????????????????????????????????????????????????????????????????????????????????????????????????????????????????????????????????????????????????????????????????????????????????????????????????????????????????????????????????????????????????????????????????????????????????????????????????????????????????????????????????????????????????????????????????????????????????????????????????????????????????????????????????????????????????????????????????????????????????????????????????????????????????????????????????????????????????????????????????????????????????????????????????????????????????????????????????????????????????????????????????????????????????????????????????????????????????????????????????????????????????????????????????????????????????????????????????????????????????????????????????????????????????????????????????????????????????????????????????????????????????????????????????????????????????????????????????????????????????????????????????????????????????????????????????????????????????????????????????????????????????????????????????????????????????????????????????????????????????????????????????????????????????????????????????????????????????????????????????????????????????????????????????????????????????????????????????????????????????????????????????????????????????????????????????????????????????????????????????????????????????????????????????????????????????????????????????????????????????????????????????????????????????????????????????????????????????????????????????????????????????????????????????????????????????????????????????????????????????????????????????????????????????????????????????????????????????????????????????????????????????????????????????????????????????????????????????????????????????????????????????????????????????????????????????????????????????????????????????????????????????????????????????????????????????????????????????????????????????????????????????????????????????????????????????????????????????????????????????????????????????????????????????????????????????????????????????????????????????????????????????????????????????????????????????????????????????????????????????????????????????????????????????????????????????????????????????????????????????????????????????????????????????????????????????????????????????????????????????????????????????????????????????????????????????????????????????????????????????????????????????????????????????????????????????????????????????????????????????????????????????????????????????????????????????????????????????????????????????????????????????????????????????????????????????????????????????????????????????????????????????????????????????????????????????????????????????????????????????????????????????????????????????????????????????????????????????????????????????????????????????????????????????????????????????????????????????????????????????????????????????????????????????????????????????????????????????????????????????????????????????????????????????????????????????????????????????????????????????????????????????????????????????????????????????????????????????????????????????????????????????????????????????????????????????????????????????????????????????????????????????????????????????????????????????????????????????????????????????????????????????????????????????????????????????????????????????????????????????????????????????????????????????????????????????????????????????????????????????????????????????????????????????????????????????????????????????????????????????????????????????????????????????????????????????????????????????????????????????????????????????????????????????????????????????????????????????????????????????????????????????????????????????????????????????????????????????????????????????????????????????????????????????????????????????????????????????????????????????????????????????????????????????????????????????????????????????????????????????????????????????????????????????????????????????????????????????????????????????????????????????????????????????????????????????????????????????????????????????????????????????????????????????????????????????????????????????????????????????????????????????????????????????????????????????????????????????????????????????????????????????????????????????????????????????????????????????????????????????????????????????????????????????????????????????????????????????????????????????????????????????????????????????????????????????????????????????????????????????????????????????????????????????????????????????????????????????????????????????????????????????????????????????????????????????????????????????????????????????????????????????????????????????????????????????????????????????????????????????????????????????????????????????????????????????????????????????????????????????????????????????????????????????????????????????????????????????????????????????????????????????????????????????????????????????????????????????????????????????????????????????????????????????????????????????????????????????????????????????????????????????????????????????????????????????????????????????????????????????????????????????????????????????????????????????????????????????????????????????????????????????????????????????????????????????????????????????????????????????????????????????????????????????????????????????????????????????????????????????????????????????????????????????????????????????????????????????????????????????????????????????????????????????????????????????????????????????????????????????????????????????????????????????????????????????????????????????????????????????????????????????????????????????????????????????????????????????????????????????????????????????????????????????????????????????????????????????????????????????????????????????????????????????????????????????????????????????????????????????????????????????????????????????????????????????????????????????????????????????????????????????????????????????????????????????????????????????????????????????????????????????????????????????????????????????????????????????????????????????????????????????????????????????????????????????????????????????????????????????????????????????????????????????????????????????????????????????????????????????????????????????????????????????????????????????????????????????????????????????????????????????????????????????????????????????????????????????????????????????????????????????????????????????????????????????????????????????????????????????????????????????????????????????????????????????????????????????????????????????????????????????????????????????????????????????????????????????????????????????????????????????????????????????????????????????????????????????????????????????????????????????????????????????????????????????????????????AAGATTACAAATTGACTTATTATACTCCTGACTATGAAACCAAAGATACTGATATCTTGGCAGCATT-CCGAGTAACTCCTCAACCTGGAGTTCCACCTGAAGAAGCAGGGGCCGCGGTAGCTGCCGAATCTTCAACTGGTACATGGACAACTGTGTGGACCGATGGACTTACCAGCCTTGATCGTTACAAAGGGCGATGCTACCACATCGAGCCCGTTGCTGGAGAAGAAAATCAATATATTGCTTATGTAGCTTACCCATTAGACCTTTTTGAAGAAGGTTCTGTTACTAACATGTTTACTTCTATTGTGGGTAATGTATTTGGGTTCAAAGCCCTGCGCGCTCTACGTCTGGAAGATCTGCGAATCCCTACCGCTTATGTTAAAACTTTCCAAGGCCCGCCTCATGGCATCCAAGTTGAGAGAGATAAATTGAACAAGTATGGTCGCCCCCTGTTGGGATGTACTATTAAACCTAAATTGGGGTTATCTGCTAAAAACTATGGTAGAGCGGTTTATGAATGTCTACGTGGTGGACTTGATTTTACCAAAGATGATGAGAA--CGT--GAACTCCCAACCATTTATGCGTT---GGAGAGATCGTTTC

Dipelta_floribunda CCTGAGCCAAATCCAGTTTTACGAAAACAAACAAGGGTTCAGAAAGCTAAAATC-AAAACGGATAGGTGCAGAGACTCAATGGAAGCTGTTCTAACAAATGGAGTTGACTGTGTTGTGTTGGTAGAAAGAATCCTTCCATAGAAACTTCAGAAAGGATAAACGTATAAACATAGATATACGCATTGAAATACTATAATAATCTACCAAATGATTAATGACGACGCGAATCTGTATTTATA-----TATATATCAAAATGGGAGAATGGTTGTGAAGTGATTCCATATTGAAGAAAGAATCAAATATTTATTGATCAAATCATTCACTCCATAGTCTGATAGATC-TTTTGAAGAACTGATTAATTGGACGAGAATAAAGATAGAGTCCCATTCTACATGTCAATACCGGCAACAATGAAATTTATAGTAAGAGGAAAATCCGTCGACTTTAGAAATCGTGAGGGTTCAAGTCCCTCTATCCCCAAAAAACCCATATGGACTCCCTAATTATTTATCCTCTCCTT-TTATCC-TTTTTTGTTAGCGGTTATAAATTCG------TTATCTTTCTCATTCACCCTACTCTTTTACAAAGAGATCTGAGCGGAAATGTTTTTCTCTTATC----ACAAGTCTTGTGATCTAAG----ATAA-TACGTGTACAAATGAACATCTTTGAGTAAGGAATCCCCATTTAAATGATTCATGGTCAA------TGTCATTATTCATACTGAAACTTACAAAGTCTTCCTT-TTGAAGATCCAAGAAATTCCAGGACCTGGATAAGACTTTGTAAGACCCTTTCAATTGACA-TAGACCCGCGTTATCTAGC--AAAATGAGGATGCA?????????????????????????????????????????????????????????????????????????????????????????????????????????????????????????????????????????????????????????????????????????????????????????????????????????????????????????????????????????????????????????????????????????????????????????????????????????????????????????????????????????????????????????????????????????????????????????????????????????????????????????????????????????????????????????????????????????????????????????????????????????????????????????????????????????????????????????????????????????????????????????????????????????????????????????????????????????????????????????????????????????????????????????????????????????????????????????????????????????????????????????????????????????????????????????????????????????????????????????????????????????????????????????????????????????????????????????????????????????????????????????????????????????????????????????????????????????????????????????????????????????????????????????????????????????????????????????????????????????????????????????????????????????????????????????????????????????????????????????????????????????????????????????????????????????????????????????????????????????????????????????????????????????????????????????????????????????????????????????????????????????????????????????????????????????????????????????????????????????????????????????????????????????????????????????????????????????????????????????????????????????????????????????????????????????????????????????????????????????????????????????????????????????????????????????????????????????????????????????????????????????????????????????????????????????????????????????????????????????????????????????????????????????????????????????????????????????????????????????????????????????????????????????????????????????????????????????????????????????????????????????????????????????????????????????????????????????????????????????????????????????????????????????????????????????????????????????????????????????????????????????????????????????????????????????????????????????????????????????????????????????????????????????????????????????????????????????????????????????????????????????????????????????????????????????????????????????????????????????????????????????????????????????????????????????????????????????????????????????????????????????????????????????????????????????????????????????????????????????????????????????????????????????????????????????????????????????????????????????????????????????????????????????????????????????????????????????????????????????????????????????????????????????????????????????????????????????????????????????????????????????????????????????????????????????????????????????????????????????????????????????????????????????????????????????????????????????????????????????????????????????????????????????????????????????????????????????????????????????????????????????????????????????????????????????????????????????????????????????????????????????????????????????????????????????????????????????????????????????????????????????????????????????????????????????????????????????????????????????????????????????????????????????????????????????????GTATTAG-TATATCTAATACCTTACCCAGCCCATTTGGAAATTTTGGTTCAAACTCTTCGCTACTGGGTAAAAGATGCCCCCTCTTTGCATTTATTACGATTCTTTCTTCACGACTATCGGCATTGGACTAGTCTTTTTATTCCAAAGAAAGCCAGTTCTTTTTTTTCAAAACGAAAG------------------CAAAGATTATTCTTCTTCCTATATAATTCTCATGTATGTGAATACGAATCCATCTTCGTCTTTCTCCGTAACCAATCTTCTCATTTACAATCAACATCTTCTGGAGCCCTTCTTGAACGAATATATTTCTATGAAAAAATAGAACATCTTGTCGAAGTCTTTGCTAAA-GATTTTCAAGACAATCTATGGTTGTTCAAGGATCCTTTCATGCATTATGTTAGGTATCAAGGAAAGTCAATTCTCGCTTCAAAGGAGAACTTTCTTTTGATGCGTAAATGGAAATATTACTTTGTACGTTTCTGGCAATGTCATTTTTACCAGTGGTTTCAACCAGGAAGGATCTATATAAACCAATTATCCAAACATTCCCTCGACCTTCTGGGCTATCTTTCAAGTGTGCGGCTAAACCCTTTAACGATACGCAGTCAAATGCTAGAAAATTCATTTCTAATTGATAATGCTGTTAAGAAGTTCGATACCATTGTTCCAATTATTCCTCTGATTGGATCATTGGTTAAAGCGAAATTTTGTAACGTATTAGGGCATCCTGTTAGTAAGGTAGTTTGGGCAGATTTATCAGAT?????????????????????????????????????????????????????????????????????????????????????????????????????????????????????????????????????????????????????????????????????????????????????????????????????????????????????????????????????????????????????????????????????????????????????????????????????????????????????????????????????????????????????????????????????????????????????????????????????????????????????????????????????????????????????????????????????????????????????????????????????????????????????????????????????????????????????????????????????????????????????????????????????????????????????????????????????????????????????????????????????????????????????????????????????????????????????????????????????????????????????????????????????????????????????????????????????????????????????????????????????????????????????????????????????????????????????????????????????????????????????????????????????????????????????????????????????????????????????????????????????????????????????????????????????????????????????????????????????????????????????????????????????????????????????????????????????????????????????????????????????????????????????????????????????????????????????????????????????????????????????????????????????????????????????????????????????????????????????????????????????????????????????????????????????????????????????????????????????????????????????????????????????????????????????????????????????????????????????????????????????????????????????????????????????????????????????????????????????????????????????????????????????????????????????????????????????????????????????????????????????????????????????????????????????????????????????????????????????????????????????????????????????????????????????????????????????????????????????????????????????????????????????????????????????????????????????????????????????????????????????????????????????????????????????????????????????????????????????????????????????????????????????????????????????????????????????????????????????????????????????????????????????????????????????????????????????????????????????????????????????????????????????????????????????????????????????????????????????????????????????????????????????????????????????????????????????????????????????????????????????????????????????????????????????????????????????????????????????????????????????????????????????????????????????????????????????????????????????????????????????????????????????????????????????????????????????????????????????????????????????????????????????????????????????????????????????????????????????????????????????????????????????????????????????????????????????????????????????????????????????????????????????????????????????????????????????????????????????????????????????????????????????????????????????????????????????????????????????????????????????????????????????????????????????????????????????????????????????????????????????????????????????????????????????????????????????????????????????????????????????????????????????????????????????????????????????????????????????????????????????????????????????????????????????????????????????????????????????????????????????????AAGATTACAAATTGACTTATTATACTCCTGACTATGAAACCAAAGATACTGATATCTTGGCAGCATT-CCGAGTAACTCCTCAACCTGGAGTTCCACCTGAAGAAGCAGGGGCCGCGGTAGCTGCCGAATCTTCAACTGGTACATGGACAACTGTGTGGACCGATGGACTTACCAGCCTTGATCGTTACAAAGGGCGATGCTACCACATCGAGCCCGTTGCTGGAGAAGAAAATCAATATATTGCTTATGTAGCTTACCCATTAGACCTTTTTGAAGAAGGTTCTGTTACTAACATGTTTACTTCTATTGTGGGTAATGTATTTGGGTTCAAAGCCCTGCGCGCTCTACGTCTGGAAGATCTGCGAATCCCTACCGCTTATGTTAAAACTTTCCAAGGCCCGCCTCATGGCATCCAAGTTGAGAGAGATAAATTGAACAAGTATGGTCGCCCCCTGTTGGGATGTACTATTAAACCTAAATTGGGGTTATCTGCTAAAAACTATGGTAGAGCGGTTTATGAATGTCTACGTGGTGGACTTGATTTTACCAAAGATGATGAGAA--CGT--GAACTCCCAACCATTTATGCGTT---GGAGAGATCGTTTC

Dipelta_yunnanensis_BOP012201 CCTGAGCCAAATCCAGTTTTACGAAAACAAACAAGGGTTCAGAAAGCTAAAATC-AAAACGGATAGGTGCAGAGACTCAATGGAAGCTGTTCTAACAAATGGAGTTGACTGTGTTGTGTTGGTAGAAAGAATCCTTCCATAGAAACTTCAGAAAGGATAAACGTATAAACATAGATATACGCATTGAAATACTATAATAATCTACCAAATGATTAATGACGACGCGAATCTGTATA---------TATATATCAAAATGGGAGAATGGTTGTGAAGTGATTCCATATTGAAGAAAGAATCGAATATTTATTGATCAAATCATTCACTCCATTGTCTGATAGATC-TTTTGAAGAACTGATTAATTGGACGAGAATAAAGATAGAGTCCCATTCTACATGTCAATACCGGCAACAATGAAATTTATAGTAAGAGGAAAATCCGTCGACTTTAGAAATCGTGAGGGTTCAAGTCCCTCTATCCCCAAAAAACCCATATGGACTCCCTAATTATTTATCCTCTCCTT-TTATCC-TTTTTTGTTAGCGGTTATAAATTCG------TTATCTTTCTCATTCACCCTACTCTTTTACAAAGAGATCTGAACAGAAATGTTTTTCTCTTATC----ACAAGTCTTGTGATCTAAG----ATAA-TACGTGTACAAATGAACATCTTTGAGTAAGGAATCCCCATTTAAATGATTCATGGTCAA------TGTCATTATTCATACTGAAACTTACAAAGTCTTCCTT-TTGAAGATCCAAGAAATTCCAGGACCTGGATAAGACTTTGTAAGACCCTTTCAATTGACA-TAGACCCGCGTTATCTAGC--AAAATGAGGATGCAGCGGTATTTATGTTAATGCACTTCCCAATGATACGTAAACAAGGCATTTCTGGTCCTTTA--------TAGAGAAGATCTATCATAGATATTTGTAATCTATCATTTATCGCTTGGGGGAGGAACAATAGTATTTCATTGCTACAAGTATGGATTATTGAGAATAATAAGACATGTATTTGGATATTTCCCTTGAACTCCGCAATCTTTTTGATTTGACATGGATAGTTGAAGGGAATTTTCCGAAGAAAAAATGGATTAGATTATGGGAGTGTGTGACTTGAACTATTGATTGGTCTGTGCAGATATATGCCTTTCGCTATCTGCCACATTGGAATTCACAACCAAATGTGTCTTTGTTCCAACCATTGCGTAAGCCC----------CATACAGAGGATAGGCTGGTTCACTTGAAGAGAATCTTTTCTATGATCAGATCCGAATCATGTCGTACATGAGCAGGTTCCGTAAGATCCAGTAG-----AATAAGTG-AACTAGATAACCCATAATCAAGATTATGGGTTATCTAG-TTCACTTACATACGATTGAATAGTATGGAAATGCATTCATTTCCTATGCATTGACACGATCTATGATACTATCGGAGTGAAACAGGGGATCTAAGGAAGAACGGAGGCTAGGCTATATTAGTAACAAGTAAACCC-----TTTGTGTGTCTGTCAAAAGTCTCCAAGTATTTTGGGGATAAACACCGATCCTAAGGTCTGAGACGACCCAGAAAGCATT--TGATCCTATCATGATCCACCTTGTAAGCCTACTTGGGTATTGAGTATTTACTTGTAAGAACCAAATTTTTTGCGCTGGATAGTTGCAACTCCGTAAAAAGAAATCC------------------AGTCAAATTTTTC------TTTTAAT--------AAAATCATTCATATTATCATA-----TATGTGTGGATCTAGATAACATATAGATTTTATATAGATTCCTTATGGTTCTTTTTCTTTTTGCTCGAGCCGTATGATGAAAAATTATCATGTCCGGTTCCTTCGGGGGATGGAT-----CTATAAAAATTCACCTATCCCAATAACAAAAAAACCTGACCTGAATGATCCTGTATTAAGAGCTAAATTGGCTAAAGGTATGGGTCATAATTATTACGGAGAGCCCGCATGGCCCAATGATCTTTTATATATTTTTCCAGTAGAGATTG-----------------------AAG-----AAATCAATGCAATATTTTTTAGGAAAGATAAAACTGGATGAATTCAAATTCGTTCAAATGGG-----------------------ATTGGAAGGTTCCTTATTTTCATTTAGGGTGTTCGTTTTATTTCTTTTCTTAGTACTTTGGTGTAGTTTATGCTCTCTCTCCTGGAATCG-AATTGTTGT-AACTGAACGCTTCTATCCT-CTAGCTAGTA--GGGATAGAAC-----AAAAAAAATATTTTCA------TTTTTTAATGAATTCTTTCTCATTTATCCGATTTATCAAATTTGAAACAAAAAGATACATTTTTTCAATAAACACA----------AAAAAATCCTAAAGTT---------ATACTATACAAAAGGTTGTCAAAATGGAATCAATTAGTTTCACCAATTCCTTA-----------------------ATTTTTACTAATGATCTTACATATGTATGCCC-------TTCTATAGATATATATAGAG----------------ATAGAGAAACCC----------------------------ATTTTTCT-------TATTATA-----GATAAATAGGTTGATGGGGAAAATAAGACCCTGC-------CCTCGAAATGATAAAATCTACTAAAAAGAAAGGTAAAACTTTGTATCTTGTCTTTATTCTTTTTTCCTTTATTCTTTTTTCAAAAAAAAAAATTATTTTTTTTTTGAATTTATAAATTTAGTAAACAGAAGCATTTTTATTCTACTTCCATTCCCTATTGTTTTCGGTC-------AATGAATAGGGAATGGGAATTATTCATTTTATTTTTAGATTAACAATGAAATCAGAT---------AGTCAAATCAATTGGGATTATTTTAACGTTTTATGA--------CTTATTTGTTTGTCGTACAAAAAAACTTTTTGAATTCCCGGTA-------CAAAGAGAT---------TCCCCAATGACAAAG--------------------------------------------------------------------------------------------------ATAG-------ATATGTACAACTTTGACCAGCAATTTCATTTAGATC-TAAGTAAGGGCTCGAAAGAT-CCAATAGACAAAT-------------ATAAAGAAAAATAAAGAAGACCC-----TTTTGATTTTGTTCCCTTTATTCCCACGGCCTGGCCTGGTCAATACCTAGCCGGGCC----TTTTTTTGTTCCAACAAATCCTAGCTAAAAGAATTTAGCTG-------------CTTTGAACACA---------------AAAATGCTTGCTATTAAAGCAGC-------------------AATAAAAAGATGAGGGGTTATTTCCATTCTTACTTATTATTCCAT------------------TCTTACTTATTATATA--TATTTATATTATTTATATAAATTATATTATATTATTTATATAAATTATATAAAATCAAAGTATCCTTTCTTATT-----ATTCCTTCTTCCCTTTTGAGTTACTTGACGACTTTACGGGAAT--ATAAAATGAAACTG-----TGGGTTCTTA---AATAATAATGAATGCATTTTTCTGTTATGATTTCAGTGGTTTTAGTGAGCCATATCTATCAAAATCCCCCCAGCAAAAGAAAAAATAGAACTT--GTTATTTC----------------ATTTAGTTATTTAAAAGAGCCC-----TCCTTTCCGGAATCTCATTAAATTGAAATCCCCCGCGAA-AAACGT-CGACACTCTCATTTTCATGA-----TTATGATC-CTA--T-CTTTATTACGCTCAATTCCTCTGTT-CGACAAAA--------------------GTATTAG-TATATCTAATACCTTACCCAGCCCATTTGGAAATTTTGGTTCAAACTCTTCGCTACTGGGTAAAAGATGCCCCCTCTTTGCATTTATTACGATTCTTTCTTCACGACTATCGGCATTGGACTAGTCTTTTTATTCCAAAGAAAGCCAGTTCTTTTTTTTCAAAACGAAAG------------------CAAAGATTATTCTTCTTCCTATATAATTCTCATGTATGTGAATACGAATCCATCTTCGTCTTTCTCCGTAACCAATCTTCTCATTTACAATCAACATCTTCTGGAGCCCTTCTTGAACGAATATATTTCTATGAAAAAATAGAACATCTTGTCGAAGTCTTTGCTAAA-GATTTTCAAGACAATCTATGGTTGTTCAAGGATCCTTTCATGCATTATGTTAGGTATCAAGGAAAGTCAATTCTCGCTTCAAAGGAGAACTTTCTTTTGATGCGTAAATGGAAATATTACTTTGTACGTTTCTGGCAATGTCATTTTTACCAGTGGTTTCAACCAGGAAGGATCTATATAAACCAATTATCCAAACATTCCCTCGACCTTCTGGGCTATCTTTCAAGTGTGCGGCTAAACCCTTTAACGATACGCAGTCAAATGCTAGAAAATTCATTTCTAATTGATAATGCTGTTAAGAAGTTCGATACCATTGTTCCAATTATTCCTCTGATTGGATCATTGGTTAAAGCGAAATTTTGTAACGTATTAGGGCATCCTGTTAGTAAGGTAGTTTGGGCAGATTTATCAGATTTAAGACAT-AAAATTTACCCTACTTCTTTATCTTTC-TAGGAAGGGCCTTTCATGAGTTGAATAGAGATTTTCA-TTTTTTATTCATCATTCGGGTTGATGAACTAAA-CCAGATAGTTATATGAGTGAAAGAAACAGCTTATAAATTTGCAGTAAAAAGATTGAGTCTCATTTT-CTATGTAC-AAGAG-TTAAGTGAAAGTAACCATAAACATTA-GAAACGGTTTACCCCAAGATTGGTTAATTAGTGATCATGGCTTGAAGCGGGTGC-AAAAGATCAACTGTATGGGG-TTTTTACTATCTATTACCATACATGTATTACCCTAACGGGCGATTAGCAAAAAGAGGTGGATAGTTAGGAACACCAAGGTACACAAAGGATTCGTAATAGAGATTATGTAAGTTATTCAACAAAATTTTTCTGTGCATAA-AAGGAATTCTGATTGGGACTTTAAGTTGGTAGAAATGATGAAGAAGTACTCCCCCTGATTCCGATCCAGAGTATACTCCTATCCACCGATTAAGTAAATAACTATCAAGAACGAAGTAATCCTTT-ACTTT------GTTTAAAGTCCCTTTTTCTGAGAAAGGAGAATAGGAACGAAAAAAATC-------AAATAGTAAAGATATAGAATTGCACTAG-----AAAGAAAGAGATCT-----------TTCTTTCC-------------TCTATTTAGAGAGATAGAATTCTTGTCATCATTCGTGAACTAATGCGATGCCTAATTGTTTTTCGTAATCGAAAATGCTAGGTTG--------AAATATCTATGAATA-TTGCTACAAGAAAGA-TTTTATTGAAAG-CTTAAGTTATCACTCAAC-AAAGAAAAATAAAAATTTATT-----AAAAGATAAGATCAA-TTCCG-AAGCACTTTA-TTTTCAATATAGCAGACAGAATTCCATTGTCTAATTCGGG--ACTTTACGGTAGATTTTGATTCTATCTATCCTACGAATATAT-------------------CAAGATAAATAATAGCGAACTGGTCCTTAGA-TTTAGTTGTGA-CCTTTGAGGAGCC-GTATGAGATGAAAATCTCATGTACGGTATTTAGATTA---------------------------------------------------------------------------------------------------------------------------------------------------------------------------------CTAAA------AATATTAACGATTTTTCTTT-CATTTTTCTTTAAATAAA--ATATGTAAGTCAA--------------------------------------------------------------------------------TGTGAAAT-AAAAAAGGAGCAATAATCCCCTTCTTGTTCTATCAAG-AGGGCGATATTGCTCCTTTTTT-ATTTCTTTCAAATACTCGTATACACTAAGGCCGGGTCTTATCCATTTATAGATGGAGCTTCAAGAGCAGCTAGGTCTAGAGGGAAGTTATGAGCATTACGTTCATGCATAACTTCCATACCAAGGTTAGCGCGGTTAATGATATCAGCCCAAGTATTAATTACACGACCTTGACTATCAACTACAGATTGGTTGAAATTAAAACCATTTAGGTTGAAAGCCATAGTGCTGATACCTAAAGCAGTGAACCAGATACCTACTACAGGCCAAGCAGCTAGGAAGAAATGTAAAGAACGAGAGTTGTTGAAACTAGCATATTGGAAGATCAATCGGCCAAAATAACCATGAGCAGCTACAATATTATAAGTTTCTTCTTCTTGA-CCGAATC---TGTAACCTTCATTAGCAATAAATGCAAGAATATTTAC-TTCCATAATCTCATCGTTTTTTTACTTCAAAATAACT-CGGGATTTAATCCCATAGAGATAATAAATCTTTCGCCTGTCAATTCA----ATGAATTACCTCTCGATGATC-TTGAAATCGGATCAATATCATGAATAACAATATCTGAGCTATCAAATCAATTCGTCGTCGAGAATTTAATAGTATAACATAGAAAGATCTTTGTATCCATATCGAATCCAAATCCAA------------------------AATTTCTTTATTTAAAAATCATTCTTTTCTGTTCTTTCTTTATCTACAACCTATCTTAGGTCCTCCTTGTACAATCATCGGATAAAGTATCGTCTGACCGCCCGTCCGTTTCCATTAGTCACAAACGCCCAACAAACAATAGAAGCGAAGTGGAAAAAGAAATAAGTTACGTTCTAAACTCCG----TTTTTTTAATGATCTAGTTTTCTTGGAAGACAAAGAAGTGTGATAAAGAGGAGTTCCGGGATAAAGGATGTAATATTCCATCAAACTAACTATTTGAGTTTGGGTTTTGTTCGTTCTTCGACGGG--CCCTAAAAAAAAAAAAAAAAATAG--AAAAAAATAGGAAGGAAAAAT-GATTTATTCCCCTGCTACTTGCTAAGCTAAAAAAGGGGTGGGATCTTTGATTGATCTTTATTTTTCTTTTA-CCCCCCCTTCCTT---------------------------------------------------------------------------------------------------------------------------------------------------------------------------------------------------------------------------------------------------------------------------------------------------------------AACGCTCTCAATAATTGTACTATTCTACATATGTCTTTCTCCTACCAATCAGTATTATTTGAAATAATGAAAATTCCCCTATTTGTTTGATGAGAAGTGC----GAAATGCCAAAGGAAA--GAAAAAAGAACCCCCTT-GGGAATG-AAATTCTGCTCCCCGTGCCCC--------GAAAAGGGAAGATTACAAATTGACTTATTATACTCCTGACTATGAAACCAAAGATACTGATATCTTGGCAGCATT-CCGAGTAACTCCTCAACCTGGAGTTCCACCTGAAGAAGCAGGGGCCGCGGTAGCTGCCGAATCTTCAACTGGTACATGGACAACTGTGTGGACCGATGGACTTACCAGCCTTGATCGTTACAAAGGGCGATGCTACCACATCGAGCCCGTTGCTGGAGAAGAAAATCAATATATTGCTTATGTAGCTTACCCATTAGACCTTTTTGAAGAAGGTTCTGTTACTAACATGTTTACTTCTATTGTGGGTAATGTATTTGGGTTCAAAGCCCTGCGCGCTCTACGTCTGGAAGATCTGCGAATCCCTACCGCTTATGTTAAAACTTTCCAAGGCCCGCCTCATGGCATCCAAGTTGAGAGAGATAAATTGAACAAGTATGGTCGCCCCCTGTTGGGATGTACTATTAAACCTAAATTGGGGTTATCTGCTAAAAACTATGGTAGAGCGGTTTATGAATGTCTACGTGGTGGACTTGATTTTACCAAAGATGATGAGAA--CGT--GAACTCCCAACCATTTATGCGTT---GGAGAGATCGTTTC

Heptacodium_miconioides_BOP012292 CCTGAGCCAAATCCAGTTTTCTGAAAACAAACAGGGGTTCAGAAAGCAAAAAT--AAAAAGGATAGGTGCAGAGACTCAATGGAAGCTGTTCTAACAAACGGAGTTGACTGTCTTGTGTTGGTAGAAAGAATCCTTCCATAGAAACTTCAGAAAGGATAAACCTATAAACATAGATATACGTATTGAAATGCTATGATACTATATCAAATGATTAATGACGATCCGAATCTGTATCTGTATTTTACATATATCAAAATGGAAGAATTGTTGTGAAGTGATTCCATATTGAAGAAAGAATCGAATATTCATTGATCAAATCATTCACTCCATAGTCTGATCGATC-TTTTGAAGAACTGATTAATCGGACGAGAATAAAGATAGAGTCCCATTCTACATGTCAAGACTGGCAACAATGAAATTTATAGTAAGAGGAAAATCCG-CGACTTTAGAAATCGTGAGGGTTCAAGTCCCTCTATCCCCAAAAAAACCATATTGACTCCCTAATTATTTATCCTCTCCTTTTTATCCTTTTTTTGTTCCCGGTCCAAAATTCG------TTATATTTCTTATCCACCCTACTCTTTTACAAAGAGATCTGAGCGGAAATGTTTTTCTCTTATC----ACAAGTCTTGTGATCTAAGTAATATATATACGTGTACAAATGAACATCTTTGAGTAAGGAATCCCCATTTGAATGATTCACGGTCAA------TATCATTATTCATACTGAAACTTACAAAGTCTTCCTT-TTGAAGATCCAAGAAATTCCAGGACCTGGATAAGACTTTGTAATACCCTTTCAATTGACA-TAGACTCGAGTTATCTAGT--AAAATGAGGATGCA????????????????????????????????????????????????????????????????????????????????????????????????????????????????????????????????????????????????????????????????????????????????????????????????????????????????????????????????????????????????????????????????????????????????????????????????????????????????????????????????????????????????????????????????????????????????????????????????????????????????????????????????????????????????????????????????????????????????????????????????????????????????????????????????????????????????????????????????????????????????????????????????????????????????????????????????????????????????????????????????????????????????????????????????????????????????????????????????????????????????????????????????????????????????????????????????????????????????????????????????????????????????????????????????????????????????????????????????????????????????????????????????????????????????????????????????????????????????????????????????????????????????????????????????????????????????????????????????????????????????????????????????????????????????????????????????????????????????????????????????????????????????????????????????????????????????????????????????????????????????????????????????????????????????????????????????????????????????????????????????????????????????????????????????????????????????????????????????????????????????????????????????????????????????????????????????????????????????????????????????????????????????????????????????????????????????????????????????????????????????????????????????????????????????????????????????????????????????????????????????????????????????????????????????????????????????????????????????????????????????????????????????????????????????????????????????????????????????????????????????????????????????????????????????????????????????????????????????????????????????????????????????????????????????????????????????????????????????????????????????????????????????????????????????????????????????????????????????????????????????????????????????????????????????????????????????????????????????????????????????????????????????????????????????????????????????????????????????????????????????????????????????????????????????????????????????????????????????????????????---------------------------------------------------------------------------------------------ATTATATAG-------ATATGTACAACTTTTACCAGCAATTTCATTTAGATA-TAAGTAAGAGGTCGAAAGATCCCAATAGACAAAT-------------ATAAAGAAAAATAAAGAAGMCCCTGTTGCTTTGATTTTGTTCCTTTTATTCCCCCGGCCTGGCCTGGTCAATACCTAGCCGGGCC----TTTTTTTGTTCCAACGAATCCTAGCTAAAAGAATTGATCTGATTTGAAATTTGAATTTGAAAACA---------------AACATGCTTGCTATTAAAGCAGG-------------------AATAAAAAGACGAGGGGCTATTTCCATTC----------------------------------------------GATTTCCATTC---------------------------TTATTATATAAATTATATAAAAA-AAAGTCTCATTTCTTATTTGATTATTCCTTCTTCCTTTTTGAGTTACTTGACGACCTTACGGGAATAAAAAAAATGAAACTA-----TGGATTCTGA---AATAATAATGAATGCATTTTTCTGTTATGATTTCAGTGGTTTTAGCGAGCCATATCTATCAAAACCCCTCCGGCAAAAGAAAAGATAGAACTT--GTT---------------------ATTTAGTTATTTAAAAAAGCCCTCCCCTCCTTTCCGGAATCTCATTAAACTGAAACCCCCCGTGAA-AAACGTCCGACACTCTCATTTTCATGATTCTTTTATGATC-CTA--TACTTTATTACGCCT-----------------------------------------GTATTAGATATA-CTAATACCTTACCCAGCCCATTTGGAAATTTTGGTTCAAACTCTTCCCTACTGGGTAAAAGATGCCCCTTCTTTGCATTTATTACGATTCTTTCTCCACGACTATCGCCATTGGACTAGGCTTTTTATTCCAAAGAAAGCCAGTTCTTCTTTTTCAAAACGAAAT------------------CAAAGATTATTCTTCTTACTATATAATTCTCATGTATGTGAATACGAATCCATCTTTGTCTTTTTCCGTAACCAATCTTCTCATTTACGATCAACATCTTTGGGAGCCCTTCTTGAACGAATATATTTCTATGAAAAAATAGAACATCTTGTAGAAGTCTTTGCTAAG-GATTTTCAAGCCCATCTATGGTTGTTCAAGGATCCTTTCATGCATTATGTTAGATATCAAGGAAAGTCAACTCTCGCTTCAAAGGGGAACTTTCTTTTGATGAATAAATTGAAATATTACTTTGTACGTTTCTGGCAATGTCATTTTTACCAGTGGTTTCAACCAGGAAGGATCTCTATAAACCAATTAGCCAAACATTCCCTCGACCTTCTGGGCTATCTTTCAAGTGTGCGGCTAAACCCCTTAACGGTACGCAGTCAAATGCTAGAAAATACATTTCTAATAAATAATGCTGTTAATAAGTTCGATACTATTGTTCCAATTATTCCTCTGATTGGATCATTGACTAAAGCGAACTTTTGTAACATATTAGGGCATCCTGTTAGTAAGGTGGTTTGGGCCGATTTATCAGAT????????????????????????????????????????????????????????????????????????????????????????????????????????????????????????????????????????????????????????????????????????????????????????????????????????????????????????????????????????????????????????????????????????????????????????????????????????????????????????????????????????????????????????????????????????????????????????????????????????????????????????????????????????????????????????????????????????????????????????????????????????????????????????????????????????????????????????????????????????????????????????????????????????????????????????????????????????????????????????????????????????????????????????????????????????????????????????????????????????????????????????????????????????????????????????????????????????????????????????????????????????????????????????????????????????????????????????????????????????????????????????????????????????????????????????????????????????????????????????????????????????????????????????????????????????????????????????????????????????????????????????????????????????????????????????????????????????????????????????????????????????????????????????????????????????????????????????????????????????????????????????????????????????????????????????????????????????????????????????????????????????????????????????????????????????????????????????????????????????????????????????????????????????????????????????????????????????????????????????????????????????????????????????????????????????????????????????????????????????????????????????????????????????????????????????????????????????????????????????????????????????????????????????????????????????????????????????????????????????????????????????????????????????????????????????????????????????????????????????????????????????????????????????????????????????????????????????????????????????????????????????????????????????????????????????????????????????????????????????????TAGCAATAAATGCAAGGATATTTAC-TTCCATAATCTTATC-TTTTTTTACTTCAAAATAACT-CGGGATTTAATCCCATAGAGATGATAAATGTTTCGCCTGTAAATTCAATGGATGAATTACCTCTCGATGATA-TTG-AATCGGATCAATATCATGAATAACAATATCTGAGCTATCAAATCAATTCATCGTCGAGAATTGAATAGTATACCATAGGAAGATCTTT-TATCCATACCG------AATCCAAAATAGGATTCCTGATCCAATCAAGAATTCTTTTATTTATCATTCTTTCTGTTCCGTTCTTTCTTT-TCTATAACCTACTCTACGCCTTCCTT------------------GTATCATCTGACCACCGTTCCGCGTCCAGTAGTCACAAACA-CAAATAAACCATAGAAGTGAAGTGGAAAAAGAAAGAAGTTAAGTTCTAAACTCCG-----TTTTTTAATGATCTAATTTTCTTGGAAGACAAAGAAGTGTGATAAAGATGAATCTCGGTAGAAAAGATCGAATATTCCATCAA--------------TTTAGGGTT---------TTCAATGGAACTCCT--------AAAAGAAAATAA--------ATAGGAAGAAAAAA------------------------------AAAGATCCCACGAAATCCT-------------------------GCCCCCTGTCCT----------------------------------------------------------------------------------------------------------------------------------------------------------------------------------------------------------------------------------------------------------------------------------------------------------------ACCTTTCGCAGAAA------------------------------------AGAGAGAGTTGAATTCATG----------TATTTATTGCATCCGTCG-------GGACTGAC----------GGGACTCGAACCCGC-----------AGCTTCCGCC---------------------------AAGATTACAAATTGACTTATTATACTCCTGACTATGAAACCAAAGATACTGATATCTTGGCAGCATT-TCGAGTAACTCCTCAACCTGGAGTTCCACCTGAAGAAGCAGGGNNNNNAGTAGCTGCCGAATCTTCAACTGGTACATGGACAACTGTGTGGACCGATGGACTTACCAGCCTTGATCGTTACAAAGGGCNNTGCTACCACATCGAGCCCGTTGCTGGAGAAGAAACTCAATNTATTGCTTATGTAGCTTACCCANNNNNCCTTTTTGAAGAAGGTTCTGTTACTAACATGTTTACTTCTATTGTGGGTAATGTATTTGGGTTCAAANNNCTGCGTGCTCTACGTCTGGAAGATCTGCGAATCCCTGTCGCTTATGTTAAAACTTTCCAAGGCCNNNCTCATGGCATCCAAGTTGAGAGAGATAAATTGAACAAGTACGGTCGCCCCCTGTTGGGATGTACTATTAAACCTAAATTAGGGTTATCTGCTAAAAACTACGGTAGAGCGGTTTATGAATGTCTACGTGGTGGACTTGATTTTACCAAAGATGATGAGAA--CGT--GAACTCCCAACCATTTATGCGTT---GGAGAGATCGTTTC

Kolkwitzia_amabilis_BOP012293 CCTGAGCCAAATCCAGTTTTACGAAAACAAACAAGGGTTCAGAAAGCTAAAATC-AAAAAGGATAGGTGCAGAGACTCAATGGAAGCTGTTCTAACAAATGGAGTTGACTGTGTTGTGTTGGTAGAAAGAATCCTTCCATAGAAACTTCAGAAAGGATAAACGTATAAACATAGATATACGCATTGAAATACTAT-ATACTCTACCAAATGATTAATGACGACCCGAATCTGTATT--TA-----TATATATCAAAATGGGAGAATGGTTGTGAAGTGATTCCATATTGAAGAAAGAATCGAATATTTATTGATCAAATCATTCACTCCATAGTCTGATAGATC-TTTTGAAGAACTGATTAATTGGACGAGAATAAAGATAGAGTCCCATTCTACATGTCAATACCGGCAACAATGAAATTTATAGTAAGAGGAAAATCCGTCGACTTTAGAAATCGTGAGGGTTCAAGTCCCTCTATCCCCAAAAAACCCATATGGACTCCCTAATTATTTATCCCCTCCTT-TTATCC-TTTTTTGTTAGCGGTTAAAAATTCG------TTATCTTTCTCATTCACCCTACTCTTTTACAAAGAGATCTGAGCGGAAATGTTTTTCTCTTATC----ACAAGTCTTGTGATCTAAG----ATAA-TACGTGTACAAATGAACATCTTTGAGTAAGGAATCCCCATTTAAATGATTCATGGTCAA------TGTCATTATTCATACTGAAACTTACAAAGTCTTCCTT-TTGAAGATCCGAGAAATTCCAGGACCTGGATAAGACTTTGTAAGACCCTTTCAATTGACA-TAGACCCGCGTTATCTAGC--AAAATGAGGATGCAGCGGTATTTATGTTAATGCACTTCCCAATGATACGTAAACAAGGCATTTCTGGTCCTTTATAGAGAAGTAGAGAAGATCTATCATAGATATTTGTAATCTATCATTTATCGCTTGGGGGAGGAACAATAGTATTTCATTGCTACAAGTATGGATTATTGAGAATAATAAGACATGTATTTGGATATTTCCCTTGAACTCCGCAATCTTTTTGATTTGACATGGATAGTTGAAGGGAATTTTCCGAAGAAAAAATGGATTAGATTATGGGAGTGTGTGACTTGAACTATTGATTGGTCTGTGCAGATATATGCCTTTCGCTATCTGCCACATTGGAATTCACAACCAAATGTGTCTTTGTTCCAACCATTGCGTAAGCCC----------CATACAGAGGATAGGCTGGTTCACTTGAAGAGAATCTTTTCTATGATCAGATCCGAATCATGTCGTACATGAGCAGGTTCCGTAAGATCCAGTAG-----AATAAGTG-AACTAGATAACCCATAATCGAGATTATGGGTTATCTAG-TTCACTTACATATGATTGAATAGTATGGAAATGCATTCATTTCCTATGCATTGACACGATCTATGATACTATCGGAGTGAAACAGGGGATCTAAGGAAGAACAGAGGCTAGGCTATATTAGTAACAAGTAAACCC-----TTTGTGTGTCTGTCAAAAGTCTCCAAGTATTTTGGGGATAAACACCGATCCTAAGGTCTGAGACGACCCAGAAAGCATT--TGATCCTATCATGATCCACCTTGTAAGCCTACTTGGGTATTGAGTATTTACTTGTAAGAACCAAATTTTTTGCGCTGGATAGTTGCAACTCCGGAAAAAGAAATCC------------------AGTCAAATTTTTCTTTTAATTTTAAT--------AAAATCATTCATATTATCATA-----TATGTGTGGATCTAGATAACATATAGATTTTATATGGATTCCTTATGGTTCTTTTTCTTTTTGCTCGAGCCGTATGATGAAAAATTATCATGTCCGGTTCCTTCGGGGGATGGAT-----CTATAAAAATTCACCTATCCCAATAACAAAAAAACCTGACCTGAATGATCCTGTATTAAGAGCTAAATTGGCTAAAGGTATGGGTCATAATTATTACGGAGAGCCCGCATGGCCCAATGATCTTTTATATATTTTTCCAGTAGAGATTG-----------------------AAG-----AAATCAATGCAATATTTTTTAGGAAAGATAAAACTGGATGAATTCAAATTCGTTCAAATGGG-----------------------ATTGGAAGGTTCCTTATTTTCATTTAGGGTGTTCGTTTTATTTCTTTTCTTAGGACTTTGGTGTAGTTTATGCTCTCTC--CTGGAATCG-AATTGTTGT-AACTGGACGCTTCTATCCT-CTAGCTAGTAGGGGGATAGAAC-----AAAAAAAATATTTTCA------TTTTTTAATGAATTCTTTCTCATTTATCCGATTTATCAAATTTGAAACAAAAAGATACATTTTTTCAATAAACACA----------AAAAAATCCTAAAGTT---------ATACTATACAAAAGGTTGTCAAAATGGAATCAATTAGTTTCACCAATTCCTTA-----------------------ATTTTTACTAATGATCTTACA----TATGCCC-------TTCTATAGATATATAGAG------------------ATAGAGAAACCC----------------------------ATTTTTCT-------TATTATA-----GATAAATAGGTTGATGGGGAAAATAAGACCCCGC-------CCTCGAAATGATAAAATCTACTAAAAAGAAAGGTAAAACCTTGTATCTTGTCTTTATTC---------------TTTTTTCAAAAAA---------TTCTTTTTTTAATTTAGAAATTTAGTAAACAGAAGCATTTTTATTCTACTTCCATTCCCTATTGTTTTCGGCC-------AATGAATAGGGAATGGGAATTAGTCATTTTATTTTTAGATTAACAATGAAATCAGAT---------AGTCAAATCAATTGGGATTATTCTAACGTTTTATGA--------CTTATTTGTTTGTCGTACAAAAAAACTTTTTGAATTCCCGGT-----------------------------------ACAAAGGCCATCTCCCCCAATTGAACAAGAGAATAATGACTATGTTACATTACACATGAAGTAA-----GAAAAAAGTC--TTG-CTTTCTCTTTCTTTATGATATAG-------ATATGTACAACTTTGACCAGCAATTTCATTTAGATC-TAAGTAAGGGCTCGAAAGAT-CCAATAGACAAAT-------------ATAAAGAAAAATAAAGAAGACCC-----TTTTGATTTTGTTCCCTTTATTCCCACGGCCTGGCCTGGTCAATACCTAGCCGGGCC----TTTTTTTGTTCCAACAAATCCTAGCTAAAAGAATTTAGCTG-------------CTTTGAACACA---------------AAAATGCTTGCTATTAAAGCAGC-------------------AATAAAAAGATGAGGGGTTATTTCCATTCTTACTTATTATTCCAT------------------TCTTACTTATTATATA--TAT-----------------------------TATTTATATAAATTATATAAAATCAAAGTATCCTTTCTTATT-----ATTCCTTCTTCCCTTTTGAGTTACTTGACGACCTTACGGGAAT--ATAAAATGAAACTA-----TGGGTTCTTA---AATAATAATGAATGCATTTTTCTGTTATGATTTCAGTGGTTTTAGTGAGCCATATCTATCAAAATCCCCCCAGCAAAAGAAAAAATAGAACTT--GTTATTTC----------------ATTTAGTTATTTAAAAGAGCCC-----TCCTTTCCGGAATCTCATTAAATTGAAATCCCCCGCGAA-AAACGT-CGACACTCTCATTTTCATGA-----TTATGATC-CTA--T-CTTTATTACGCCTAATTCCTCGGTT-CGACAAAAAGTTCATTTGTATATAATAAGTATTAG-TATATCTAATACCTTACCCAGCCCATTTGGAAATTTTGGTTCAAACTCTTCGCTACTGGGTAAAAGATGCCCCCTCTTTGCATTTATTACGATTCTTTCTTCACGACTATCGGCATTGTTCTAGTCTTTTTATTCCAAAGAAAGCCAGTTCTTTTTTTTCAAAACGAAAG------------------CAAAGATTGTTCTTCTTCCTATATAATTCTCATGTATGTGAATACGAATCCATCTTCGTCTTTCTCCGTAACCAATCTTCTCATTTACAATCAACATCTTCTGGAGCCCTTCTTGAACGAATATATTTCTATGAAAAAATAGAACATCTTGTCGAAGTCTTTGCTAAA-GATTTTCAAGGCAATCTATGGTTGTTCAAGGATCCTTTCATGCATTATGTTAGGTATCAAGGAAAGTCAATTCTCGCTTCAAAGGAGAACTTTCTTTTGATGCGTAAATGGAAATATTACTTTGTACGTTTCTGGCAATGTCATTTTTACCAGTGGTTTCAACCAGGAAGGATCTATATAAACCAATTATCCAAACATTCCCTCGACCTTCTGGGCTATCTTTCAAGTGTGCGGCTAAACCCTTTAACGATACGCAGTCAAATGCTAGAAAATTCATTTCTAATTGATAATGCTGTTAAGAAGTTCGATACCATTGTTCCAATTATTCCAATGATTGGATCATTGGTTAAAGCGAAATTTTGTAACGTATTAGGGCATCCTGTTAGTAAGGTAGTTTGGGCAGATTTATCAGATTTAAGACAT-AAAATTTACCCTACTTCTTTATCTTTC-TAGGAAGGGCCTTTCATGAGTTGAATAGAGATTTTCA-TTTTTTATTCATCATTCGGGTTGATGAACTAAACCCAGATAGTTATATGAGTGAAAGAAACAGCTTATAAATTTGCAGTARAAAGATTGAGTCTCATTTT-CTATGTAC-AAGAG-TTAAGTGAAAGTAACCATAAACWTTA-SAAACGGKTTACCCCAAGATTGGTTAATTAGTGATCATGGCTTGAAGCGGGTGC-AAAAGATCAACTGTATGGGG-TTTTTACTATCTATTACCATACATGTATTATCCTAACGGGCGATTAGCAAAAAGAGGTGGATAGTTAGGAACACCAAGGTACACAAAGGATTCGTAATAGAGATTATGTAAGTTATTCAACAGAATTTTTCTGTGCATAA-AAGGAATTCTGATTGGGACTTTAAGTTGGTAGAAATGATGAAGAAGTACTCCCCCTGATTCCGATCCAGAGTATACTCCTATCCACCGATTAAGTAAATAACTATCAAGAACGAAGTAATCCTTT-ACTTT------GTTTAAAGTCCCTTTTTCTGAGAAAGGAGAATAGGAACGAAAAAAATC-------AAATAG-AAAGA-ATAGAATTGCACTAG-----AAAGAAAGAGATCTTTTTT-ATTCTTTCTTTCC-------------TCTATTTAGAGAGATAGAATTCTTGTCATCATTCGTGAACTAATGCGATGCCTAATTGTTTTTCGTAATCGAAAATGCTAGGTTG--------AAATATCTATGAATA-TTGCTACAAGAAAGA-TTTTATTGAAAG-CTTAAGTTATCACTCAAC-AAAGAAAAATAAAAA-TTCTT-----AAAAGATAAGATCAA-TTCCG-AAGCACTTTA-TTTTCAATATAGCAGACAGAATTCCATTGTCTAATTCGGG--ACTTTACGGTAGATTTTGATTCTATCTATCCTACGAATATAT-------------------CAAGATAAATAATAGCGAACTGGTCCTTAGA-TTTATTTGTGA-CCTTTGAGGAGCC-GTATGAGATGAAA-------------TATTTTTATTA---------------------------------------------------------------------------------------------------------------------------------------------------------------------------------CTAAA------AAAATTTAGTATTTTTCTTT-AA-----------AGAAA--ATATGTAAGTCAA--------------------------------------------------------------------------------TGTGAAAT--AAAAAGGAGCAATAATCCCCT-CTTGATAGAACAAGAAGGGGATTATTGCTCCTTTTTT-ATTTCTTTCAAATACTCGTATACACTAAGGCCGGGTCTTATCCATTTATAGATGGAGCTTCAAGAGCAGCTAGGTCTAGAGGGAAGTTATGAGCATTACGTTCATGCATAACTTCCATACCAAGGTTAGCGCGGTTAATGATATCAGCCCAAGTATTAATTACACGACCTTGACTATCAACTACAGATTGGTTGAAATTAAAACCATTTAGGTTGAAAGCCATAGTGCTGATACCTAAAGCAGTGAACCAGATACCTACTACAGGCCAAGCAGCTAGGAAGAAATGTAAAGAACGAGAGTTGTTGAAACTAGCATATTGGAAGATCAATCGGCCAAAATAACCATGAGCAGCTACAATATTATAAGTTTCTTCTTCTTGA-CCGAATC---TGTAACCTTCATTAGCAATAAATGCAAGAATATTTAC-TTCCATAATCTCATCGTTTTTTTACTTCAAAATAACT-CGGGATTTAATCCCATAGAGATAATAAATCTTTCGCCTGTCAATTCA----ATGAATTACCTCTCGATGATC-TTGAAATCGGATCAATATCATGAATAACAATATCTGAGCTATCAAATCAATTCGTCGTCGAGAATTGAATAGTATAACATATAAAGATCTTT-TATCCATACCG------AATCCAA------------------------AATTTCTTTATTTCTCAATCATTCTTTTCTGTTCTTTCTTTATCTACAACCTATCTTAGGTCCTCCTTGTACAATCATCGGATAAAGTATCGTCTGACCGCCCGTCCGTTTCCATTAGTCACAAACGCCCAACAAACAATAGAAGCGAAGTGGAAAAAGAAATAAGTTACGTTCTAAACTCCG----TTTTTTTAATGATCTAGTTTTCTTGGAAGACAAAGAAGTGTGATAAAGAGGAGTTCCGGTATAAAGGATGTAATATTCCATCAAACTAACTATTTGAGTTTGGGTTTTGTTCGTTCTTCGACGGG--CCCT-------AAAAAAAAAATAG-AAAAAAAATAGGAAGGAAAAAT-GATTTATTCCCCTGCTACTTGCTAAGCTAAAAAAGGGGTGGAATCTTTGATTGATCTTTATTTTTCTTTTA--CCCCCCTTCCTT---------------------------------------------------------------------------------------------------------------------------------------------------------------------------------------------------------------------------------------------------------------------------------------------------------------AACGCTCTCAATAATTGTCCTATTCTACATATGTCTTTCTCCTACCAATCAGTATTATTTGAAATAATGAAAATTCCCCTATTTGTTTGATGAGAAGTGC----GAAATGCCAAAGGAAA--GAAAAAAGAACCCCCTT-GGGAATG-AAATTCTGCTCCCCGTGCCCCCTTTAACAGAAAAGGGAAGATTACAAATTGACTTATTATACTCCTGACTATGAAACCAAAGATACTGATATCTTGGCAGCATT-CCGAGTAACTCCTCAACCTGGAGTTCCACCTGAAGAAGCAGGGGCCGCGGTAGCTGCCGAATCTTCAACTGGTACATGGACAACTGTGTGGACCGATGGACTTACCAGCCTTGATCGTTACAAAGGGCGATGCTATCACATCGAGCCCGTTGCTGGAGAAGAAAATCAATTTATTGCTTATGTAGCTTACCCACTAGACCTTTTTGAAGAAGGTTCTGTTACTAACATGTTTACTTCTATTGTGGGTAATGTATTTGGGTTCAAAGCCCTGCGCGCTCTACGTCTGGAAGATCTGCGAATCCCTCYYGCTTATGTTAAAACTTTCCAAGGCCCGCCTCATGGCATCCAAGTTGAGAGAGATAAATTGAACAAGTATGGTCGCCCCCTGTTGGGATGTACTATTAAACCTAAATTGGGATTATCTGCTAAAAACTAYGGTAGAGCAGTTTATGAATGTCTACGYGGTGGACTTGATTTTACCAAAGATGATGAGAA--CGT--G---------------------------------------

Leycesteria_crocothyrsos CCTGAGCCAAATCCAGTTTTCCGAAAACAAACAAGGGTTCAGAAAGCAAAAATC-AAAAAGGATAGGTGCAGAGACTCAATGGAAGCTGTTCTAACAAACGGAGTTGACTGTCTTGTGTTGGTGGAAAAAATCCTTCCATAGAAACTTCAGAAAGGATAAACCTATAAACATAGATATACGTATTGAAATACTATGATACTATATCAAATGATTAATGACGATCCGAATCTGTATCTGTATTTTATATATATCAAAATGGAAGAATTGTTGTGAAGTGATTCCATATTGAAGAAAGAATCGAATATTCATTAATCAAATCATTCACTCCATAGTCTGATCGATC-TTTTGAAGAACTGATTAATCGGACGAGAATAAAGATAGAGTCCCATTCTACATGTCAAGACCGGCAACAATGAAATTTATAGTAAGAGGAAAATCCGTCGACTTTAGAAATCGTGAGGGTTCAAGTCCCTCTATCCCCAAAAAACCCATATTGACTCCCTAATTATTTATCCTCTCCTTTTTATCCTTTTTTTGTTAGCGGTTCAAAATTCG------TTATATTTTTCATCCACCCTACTCTTTTACAAAGAGATCTGAGCGGAAATGTTTTTCTCTTATC----ACAAGTCTTGTGATCTAAG----ATAATTCCTTGTACAAATGAACATCTTTGAGTAAGGAATCCCCATTTGAATGATTCACGGTCAA------TATCATTATTCATACTGAAACTTACAAAGTCTTCCTT-TTGAAGATCCAAGAAATTCCAGGACCTGGATAAGACTTTGTAATACCCTTTCAATTGACA-TAGACCCGAGTTATCTAGT--AAAATGAGGATGCA????????????????????????????????????????????????????????????????????????????????????????????????????????????????????????????????????????????????????????????????????????????????????????????????????????????????????????????????????????????????????????????????????????????????????????????????????????????????????????????????????????????????????????????????????????????????????????????????????????????????????????????????????????????????????????????????????????????????????????????????????????????????????????????????????????????????????????????????????????????????????????????????????????????????????????????????????????????????????????????????????????????????????????????????????????????????????????????????????????????????????????????????????????????????????????????????????????????????????????????????????????????????????????????????????????????????????????????????????????????????????????????????????????????????????????????????????????????????????????????????????????????????????????????????????????????????????????????????????????????????????????????????????????????????????????????????????????????????????????????????????????????????????????????????????????????????????????????????????????????????????????????????????????????????????????????????????????????????????????????????????????????????????????????????????????????????????????????????????????????????????????????????????????????????????????????????????????????????????????????????????????????????????????????????????????????????????????????????????????????????????????????????????????????????????????????????????????????????????????????????????????????????????????????????????????????????????????????????????????????????????????????????????????????????????????????????????????????????????????????????????????????????????????????????????????????????????????????????????????????????????????????????????????????????????????????????????????????????????????????????????????????????????????????????????????????????????????????????????????????????????????????????????????????????????????????????????????????????????????????????????????????????????????????????????????????????????????????????????????????????????????????????????????????????????????????????????????????????????????????----------------------------------------------------------------------GTC--TTT-CTTTCTCTTTCTTTATTATATAG-------ATATGTACAACTTTTACCAGCAATTTCATTTAGATA-TAAGTAAGGGCTCGAAAGAT-CCAATAGAAAAAT-------------CTAAAGAAAAATAAAGAAGACCCCGTTGCTTTGATTTTGTTCCTTTTATTCCCACAGCCTGGCCCGGTCAATACCTAGCCGGGCC----TTTTTTTGTTCCAACGAATCCTAGCTAAAAGAATTTAGCTG-------ATTTGAATTTGAAAACA---------------AAAATGCTTGCTATTAAAGCAGC-------------------AATAAAAAGACGCGGGGCTATTTCCATTCTT-TTTAT----------------------------------ATTATATA----------------------------------AATTGATATAAATTATATA-----AAAGTCGCATTTCTTATTTTATAATTCCTTCTCCCTTTTTGAGTTACTTGACTACCTTACGGGAAT--AAAAAATGAAACTA-----TGGATTCTTA---AATAATAATGAATGCATTTTTCTGTTATGATTTCAGTGGTTTTAGCGAGCCATATCTATTAAAACCCCTCCAGCAAAAGAAAAGATAGAGCTTTAGTTATTTG----------------------TTATTTTAAAGAGCCC-CCTTTTCTTTCCGGAATCTTATGAAATTGAAATCCCCCGCGAA-AAACGT-CGACACTCGCATTTTCATGATTCTTTTATGATC-CTA--T-CTTTATTACGCCC-----------------------------------------GTATTAGGTATA-CTAATACCTTACTCAACCCATTTGGAAATTCTGGTTCAAACTCTTCGCTACTGGGTAAAAGATGCCCCTTCTTTGCATTTATTACGATTCTTTCTCCACGAGTATCCTAATTTCACTAATCTTATTATTCCAAAGAAAGCCGGTTCTTCTTTTTCAAAACGAAAT------------------CAAAGATTCTTTTTCTTCCTATATAATTCTCATGTATGTGAATACGAATTCATTCTCGTCTTTCTCCGTAACCAATCTTCTCATTTACGATCAACATCTTCTGGAACCCTTCTTGAACGAATATATTTCTATGAAAAAATAGAACATCTTGTAGAAGTCTTTGCTAAG-TATTTTAAAGCCAATCTATGGTTGTTCAAGGATCCTTTCATGCATTATGTTAGGTATCAAGGAAAGTCAATTCTCGCTTCAAGGGGGACCTTTCTTTTGATGAATAAATGGAAATATTATTTTGTACGTTTCTGGCAATGGCATTTTTCGCTGTG-------------------------------------------------------------------------------------------------------------------------------------------------------------------------------------------------------------------------------------------------------------------???????????????????????????????????????????????????????????????????????????????????????????????????????????????????????????????????????????????????????????????????????????????????????????????????????????????????????????????????????????????????????????????????????????????????????????????????????????????????????????????????????????????????????????????????????????????????????????????????????????????????????????????????????????????????????????????????????????????????????????????????????????????????????????????????????????????????????????????????????????????????????????????????????????????????????????????????????????????????????????????????????????????????????????????????????????????????????????????????????????????????????????????????????????????????????????????????????????????????????????????????????????????????????????????????????????????????????????????????????????????????????????????????????????????????????????????????????????????????????????????????????????????????????????????????????????????????????????????????????????????????????????????????????????????????????????????????????????????????????????????????????????????????????????????????????????????????????????????????????????????????????????????????????????????????????????????????????????????????????????????????????????????????????????????????????????????????????????????????????????????????????????????????????????????????????????????????????????????????????????????????????????????????????????????????????????????????????????????????????????????????????????????????????????????????????????????????????????????????????????????????????????????????????????????????????????????????????????????????????????????????????????????????????????????????????????????????????????????????????????????????????????????????????????????????????????????????????????????????????????????????????????????????????????????????????????????????????????????????????????????????????????????????????????????????????????????????????????????????????????????????????????????????????????????????????????????????????????????????????????????????????????????????????????????????????????????????????????????????????????????????????????????????????????????????????????????????????????????????????????????????????????????????????????????????????????????????????????????????????????????????????????????????????????????????????????????????????????????????????????????????????????????????????????????????????????????????????????????????????????????????????????????????????????????????????????????????????????????????????????????????????????????????????????????????????????????????????????????????????????????????????????????????????????????????????????????????????????????????????????????????????????????????????????????????????????????????????????????????????????????????????????????????????????????????????????????????????????????????????????????????????????????????????????????????????????????????????????????????????????????????????????????????????????????????????????????????????????????????????????????????????????????????????????????????????????????????????????????????????????????????????????????????????????????????????????????????????????????????????????????????????????????????????????????????????????????????????????????????????????????????????????????????????????????????????????????????????????????????????????????????????????????????????????????????????????????????????????????????????????????????????????????????????????????????????????????????????????????????????????????????????????????????????????????????????????????????????????????????????????????????????????????????????????????????????????????????????????????????????????????????????????????????????????????????????????????????????????

Leycesteria_formosa_BOP012301 CCTGAGCCAAATCCAGTTTTCCGAAAACAAACAAGGGTTCAGAAAGCAAAAATC-AAAAAGGATAGGTGCAGAGACTCAATGGAAGCTGTTCTAACAAACGGAGTTGACTGTCTTGTGTTGGTAGAAAAAATCCTTCCATAGAAACTTCAGAAAGGATAAACCTATAAACATAGATATACGTATTGAAATACTATGATACTATATCAAATGATTAATGACGATCCGAATCTGTATCTGTATTTTATATATATCAAAATGGAAGAATTGTTGTGAAGTGATTCCATATTGAAGAAAGAATCGAATATTCATTAATCAAATAATTCACTCCATAGTCTGATCGATC-TTTTGAAGAACTGATTAATCGGACGAGAATAAAGATAGAGTCCCATTCTACATGTCAAGACCGGCAACAATGAAATTTATAGTAAGAGGAAAATCCGTCGACTTTAGAAATCGTGAGGGTTCAAGTCCCTCTATCCCCAAAAAACCCATATTGACTCCCTAATTATTTATCCTCTCCTTTTTATCCTTTTTTTGTTAGGGGTTCAAAATTCG------TTATATTTTTCATCCACCCTACTCTTTTACAAAGAGATCTGAGCGGAAATGTTTTTCTCTTATC----ACAAGTCTTGTGATCTAAG----ATAATTCCTTGTACAAATGAACATCTTTGAGTAAGGAATCCCCATTTGAATGATTCACGGTTAATATCATTATCATTATTCATACTGAAACTTACAAAGTCTTCCTT-TTGAAGATCCAAGAAATTCCAGGACCTGGATAAGACTTTGTAATACCCTTTCAATTGACA-TAGACCCGAGTTATCTAGT--AAAATGAGGATGCA----------------------------------------------------------------------------------------TATTTGTAATCTATCATTTATCGCTTGGGGGAGGAACAATAGTATTTCATTGCTACAAGTATGGATTATTGAGAATAATAAGACATGTATTTGGATATTTCCCTTGAACTCCGCAATCTTTTTTATTTGACATGGATAGTTGAAGGGAATTTTTCGAAGAAAAAATGGATTAAATTATGGGAGTGTGTGACTTGAACTATTGATTGGTCTGTGCAGATATATGCCTTTCTCTATCTGCCACATTGGAATTCACAACCAAATGTGTCTTTGTTCCAACCACCGCGTAAGCCCCATACAGAGGCATACAGAGGATAGGCTGGTTCGCTTGAAGAGAATCTTTTCTATGATCAGATCCGAACCATGTCATCCATGAGCAGGCTCCGTAAGATCCAGTAG-----AATAAGTG-AACTAGATAACCGATAATCTTGATTGTGGGTTATCTAG-TTCACTTACATACGATTGAATAGTATGGAAATGCATTCATTTCCTATGCATTGACACAATCTATGATACTATCGGAGTGAAACAGGGGATCTAAGGAAGAACAGAGGTTAGGCTATATTAGTAACAAGTAAACCC-----TTTGCGTGTCTGTCAAAAGTCTCCAAGTATTTGGGGGATAAACGCCGATCCTAAGGTYTGAGACGACCCAGAAAGCACTTGTGATCCTATCACGATCCACCTTGTAAGCCTACTTGGGTATTGAGTATTTACTTGTAAGAATAAAATTCTTTGCACTGGATAGTTACAACCCCGGAAAAGAAAATCCAGTCAAAAAAGAAAATCCAGTCAAATTTGAC------TTTTCATAAAATAAAAAAATCATTCATATTATCATA-----TATGTGTGGATCTAGATAACATATAGATTTGATATGGATTCTTT-TGGTTCTTTTTATTTTTGCTCGAGCCGTATGATGAAAAATTATCATGTCCGGTTCCTTCGGGGGATGGAT-----CTATAAAAATTCACCTATCCCAATAACAAAAAAACCTGACCTGAATGATCCTGTATTAAGAGCGAAATTGGCTAAAGGTATGGGTCATAATTATTACGGAGAGCCCGCATGGCCCAATGATCTTTTATATATTTTTCCAGTAGAGATTGAA---------------------AAA-----AAACCAATGCAATATTTTTTAGGAAAGATAAAAATGAATGAATAAAAATTCGTTCAAATGGG-----------------------ATTGGAAGGTTCATTTTTTTCGTTTAGGGTGTTCATTTTATTTCTTCCCTTAGGACTTTGGTGTAGTTTATGCTCTC----CTGGAATCG-AACTGTTGT-AACTGGACGCTTCTATCCT-CTAGCTA-----GGGGTAGAACG--AAAAAAAAAAGATTTTCA------TTTTTTTATGAATTCTTTCTCATTTATCCGATTTAAAAAATTTGAAGCAAAGGGATACATTTTTTCAATGAACACAA---------AAGAAATCTGAAAGTT--------------ATACAAAAGGTTGTCAAAATGGAATCCATTCGTTTCACCAATTCCTTA-----------------------ATTTTAACTAAAGATCTTACA----TATGCCC-------TTCTATAGATATATATAGAT----------------ATAGAGAAAACTTTTTAGATATATATAGATATAGAGAAAAAATTTTCT-------TATTAGAATTGTGACAAATAGGTTGATGGGGAAAATAAGACCCCGC-------TCTCAAAATGAGAAAATCTACTAAAAAGAAAGGTAAACCCTTGTATTTTGTCTTTATTCT--------------TTTTTTCAAAAGTC--------TTCTTTTTC--------AAATTGAGTAAACAGAAGCATTTTTATTCTACTTCCATTCCCTATTGTTTTCGGCT-------AATCAATAGGAAATGGAAGTTATTCATTTTATTTTTAGATTCACAATGAAATCAGACATTTTTTCGATTCAAATTAATTAGGATTATTCCAATGTTTTATGA--------CTTATTTGTTTGTCGCACAAAAAAACTTTTTGAATTCCCGGTAG-------AAAGAGAT---------TCCCCAATGACAAAG---------------------------------------------------------------------------------------------ATTATATAG-------ATATGTACAACTTTTACCAGCAATTTCATTTAGATA-TAAGTAAGGGCTCTAAAGAT-CCAATAGAAAAAT-------------CTAAAGAAAAATAAAGAAGACCCCGTTGCTTTGATTTTGTTCCTTTTATTCCCACAGCCTGGCCCGGTCAATACCTAGCCGGGCC----TTTTTTTGTTCCAACGAATCCTAGCTAAAAGAATTTAGCTG-------ATTTAAATTTGAAAACA---------------AAAATGCTTGCTATTAAAGCAGC-------------------AGTAAAAAGACGCGGGGCTATTTCCATTTTT-TTTAT---------------------------------------ATA----------------------------------AATTGATATAAATTATATA-----AAAGTTGCATTTCTTATTTTATAATTCCTTCTCCCTTTTTGAGTTACTTGACGACTTTACGGGAAT--AAAAAATGAAACTA-----TGGATTCTTA---AATAATAATGAATGCATTTTTCTGTTATGATTTCAGTGGTTTTAGCGAGCCATATCTATTAAAACCCCTCCAGCAAAAGAAAAGATAGAGCTT--GTTATTTATATTTAGTTACTTGTTATTTAGTTATTTAAAAGAGCCC-CCTTTTCTTTCCGGAATCTTATTAAATTGAAATCCCCCGCGAA-AAACGT-CGACACTCACATTTTCATGATTCTTTTATGATC-CTA--T-CTTTATTACGCTCAATTCCTCTGTT-CGACAAAA--------------------GTATTAGGTATA-CTAATACCTTACTCAACCCATTTGGAAATTCTGGTTCAAACTCTTCGCTACTGGGTAAAAGATGCCCCTTCTTTGCATTTATTACGATTCTTTCTCCACGAGTATCCTAATTTCACTAGTCTTATTATTCCAAAGAAAGACGGTTCTTCTTTTTCAAAACGAAAT------------------CAAAGATTCTTTTTCTTCCTATATAATTCTCATGTATGTGAATACGAATTCATTCTCGTCTTTCTCCGTAACCAATCTTCTCATTTACGATCAACATCTTCTGGAACCCTTCTTGAACGAATATATTTCTATGAAAAAATAGAACATCTTGTAGAAGTCTTTGCTAAG-GATTTTCAAGCCAATCTATGGTTGTTCAAGGATCCTTTCATGCATTATGTTAGGTATCAAGGAAAGTCAATTCTCGCTTCAAGGGGGACCCTTCTTTTGATGAATAAATGGAAATATTACTTTGTACGTTTCTGGCAATGTCATTTTTACCAGTGGTTTCAACCAGGAAGGATCTCTATAAACCAATTATCCAAACATTCGCTCGACCTTCTGGGTTATCTTTCAAGTGTGCGGCTAAACCCCTTAACGGCGCGCGGTCAAATGCTAGAAAATGCATTTCTAATTGATAATGCTGTTAAGAAGTTCGATACCATTGTTCCAATTAGTCCTCTGATTGGATCATTGGCTAAAGCGAAATTTTGTAACGTATTAGGGCATCCCGTTAGTAAGGT----------------------TTGAGACAT-AAAATTGACCCTACTTCTTT-TCTTTC-TAGGAAGGGCCTTTGATGAGTTGAATATATATTTTTC-TTTTTGATTCATCATTCGGGTTGATGAACTAAA-CCAGATAGTTATATGAGTGAAAGAAACAGCTTCTAAATTTGCAGTAAAAAAATGGAATCTCATTTT-CTATGTAC-AAGAG-TGAAGTGAAAGAAAACATAAACATTA-GAAACTGTTTACCCCAAGATTGGTTAATTAGTGATCATGGCTTGAAGCGGGTGC-AAAAGATCAACTATATGGGGTTTTTTACTATCTATTCCTATACATGTATTACCCTAACGGCGGATTCGCA-AAAGAGGTGGATAGTTAGGAACACCAAGGTACACAAAGGACTCGTAATAGAGATTACGTAAGTTATTCAACAGGATTTTTCTGTGCATAA-AAGGAATTCTAATTGGAACTTTAAGTTGGTAGAAATGATGAAGAAGTACTTCCCCCGATTCCGATCCAGAGTATCCTCCTATCCACCAATTAAGTAAATAACTATCAAGAACGAAGTAATCCTTT-ACTTTGTTTAAGTTTAAAGTCCCTTTTTCTGAGAAAGGAGAATAGGAATGAAAAAAAGA-------AACTAG-AAAGA-ATATAATTGCACTAG-----AAAGAAAGAGATCTTTTTTA-----TTCTTTTC-------------TCTATTTAGAGAAATAGAATTCTTGTCATGATTCATGAACTAATGTA----CTAATTGTTTTTCGTAATCGAAAATGCTGGGTTG--------AAATATCTATTGATA-TTGCTACAAGAAAGA-TTTTATTGAAAG-CTTAAGTTATTCCTCAAC-AAAGAAAAATTAAAA-TTCTT-----AAAAGATAAGATCAA-TTCCG-AAGCACTTTA-TTTTAAATATAGCAGACAGAATTCCATTGTCTAATTCGGGACACCTTATGGTAGATTTTGACTCTACCTATCCTACGAAGATATCAAGATAAATAATAGCGAACAAGATAAATAATAGCGAACCGGTCCTTAGA-TTTATTTATGA-CCTTTGAGGAGCC-GTATGAGGTGAAAATCTCATGTACGGTTCCTAGTTCA-------------------GCCATTCTTGATTTTTCTTTCTTTTTTCTCAAGTACAATTATAGATAAGTCAGCAAATAAATTCCAACAGTTTCTCTTTTATTTTAATTTTAGAGGATATAAATATTTTTCATACAGAATAAAAAATGAAAGCCCAAAACACAATCATGACCCAACCCCTAAAAATAAGAAGATTGACCATTTTTATTG-AA-----------AGAAA--ATAGGTAAGTCAA----------------------------------------------------------------------------ATACTGTTAAAT-AAAAAAGGAGCAATGGCGCCCT-CTTGATAAAACAAGAAGGGGATTATTGCTCCTTTTTTCAT----TTCAAAAACTCGTATACACTAAGACCGGGTCTTATCCATTTGTAGATGGAGCTTCAAGAGCGGCTAGGTCTAGGGGGAAATTGTGAGCATTACGTTCATGCATAACTTCCATACCAAGGTTAGCACGATTAATGATATCAGCCCAAGTATTAATTACACGACCTTGACTATCAACTACAGATTGGTTGAAATTAAAACCATTTAGGTTGAAAGCCATAGTGCTGATACCTAAAGCAGTGAACCAGATACCTACTACAGGCCAAGCGGCTAGGAAGAAATGTAAAGAGCGAGAGTTGTTGAAACTAGCATATTGGAAGATCAATCGTCCAAAATAACCATGAGCGGCTACGATATTATAAGTTTCTTCTTCTTGA-CCGAATC---TGTAACCTTCAT?????????????????????????????????????????????????????????????????????????????????????????????????????????????????????????????????????????????????????????????????????????????????????????????????????????????????????????????????????????????????????????????????????????????????????????????????????????????????????????????????????????????????????????????????????????????????????????????????????????????????????????????????????????????????????????????????????????????????????????????????????????????????????????????????????????????????????????????????????????????????????????????????????????????????????????????????????????????????????????????????????????????????????????????????????????????????????????????????????????????????????????????????????????????????????????????????????????????????????????????????????????????????????????????????????????????????????????????????????????????????????????????????????????????????????????????????????????????????????????????????????????????????????????????????????????????????????????????????????????????????????????????????????????????????????????????????????????????????????????????????????????????????????????????????????????????????????????????????????????????????????????????-----------------------------------------------------------------TT-CCGAGTAACTCCTCAACCTGGAGTTCCACCTGAAGAAGCAGGGGCCGCGGTAGCTGCTGAATCTTCAACTGGTACATGGACAACTGTGTGGACCGATGGACTTACCAGCCTTGATCGTTACAAAGGGCGATGCTACCACATCGAGCCCGTTGCTGGAGACGAAAATCAATATATTGCTTATGTAGCTTACCCATTAGACCTTTTTGAAGAAGGTTCTGTTACTAACATGTTTACTTCTATTGTGGGTAATGTATTTGGGTTCAAAGCCCTGCGCGCCCTACGTCTGGAAGATCTGCGAATCCCTGTCGCTTATGTTAAAACTTTCCAAGGCCCGCCTCATGGTATCCAAGTTGAGAGAGATAAATTGAACAAGTACGGTCGCCCCCTGTTGGGATGTACTATTAAACCTAAATTGGGGTTGTCTGCTAAAAACTACGGTAGGGCGGTTTATGAATGTCTACGTGGTGGACTTGATTTTACCAAAGATGATGAGAA--CGT--GAACTCCCAACCATTTATGCGTT---GGAGAGATCGTTTC

Linnaea_borealis_subsp_americana CCTGAGCCAAATCCAGTTTTACGAAAACAAACAAGGGTTCAGAAAGCTAAAATC-AAAAAGGATAGGTGCAGAGACTCAATGGAAGCTGTTCTAACAAATGGAGTTGACTGTGTTGTGTTGGTAGAAAGAATCCTTCCATAGAAACTTCAGAAAGGATAAACGTATAAACATAGATATACGCATTGAAATACTAT-ATACTCTACCAAATGATTAATGACGACCCGAATCTGTATT--TA-----TATATATCAAAATGGGAGAATGGTTGTGAAGTGATTCCATATTGAAGAAAGAATCGAATATTCATTGATCAAATCATTCACTCCATAGTCTGATAGATC-TTTTGAAGAACTGATTAATTGGACGAGAATAAAGATAGAGTCCCATTCTACATGTCAATACCGGCAACAATGAAATTTATAGTAAGAGGAAAATCCGTCGACTTTAGAAATCGTGAGGGTTCAAGTCCCTCTATCCCCAAAAAACCCATATGGACTCCCTAATTATTTATCCTCTCCTT-TTATCC-TTTTTTGTTAGCGGTTAAAAATTCGTT-TCGTTATCTTTCTCATTCACCCTACTCTTTTACAAAGAGATCCGAGCGGAAATGTTTTTCTCTTATCACAAACAAGTCTTGTGATCTAAG----ATAA-TACGTGTACAAATGAACATCTTTGAGTAAGGAATCCCCATTTGAATGATTCATGGTCAA------TGTCATTATTCATACTGAAACTTACAAAGTCTTCCTT-TTGAAGATCCAAGAAATTCCAGGACCTGGATAAGACTTTGTAAGACCCTTTCAATTGACA-TAGACCCGAGTTATCTAGC--AAAATGAGGATGCAGCGGTATTTATGTTAATGCACTTCCCAATGATACGTAAACAAGGCATTTCTGGTCCTTTA--------TAGAGAAGATCTATCATAGATATTTGTAATCTATCATTTATCGCTTGGGGGAGGAACAATAGTATTTCATTGCTACAAGTATGGATTATTGAGAATAATAAGACATGTATTTGGACATTTCCCTTGAACTCCGCAATCTTTTTTATTTGACATGGATAGTTGAAGGGAATTTTCCGAAGAAAAAATGGATTAGATTATGGGAGTGTGTGACTTGAACTATTGATTGGTCTGTGCAGATATATGCCTTTCTCTATCTGCCACATTGGAATTCACAACCAAATGTGTCTTTGTTCCAACCATTGCGTAAGCCC----------CATACAGAGGATAGGCTGGTTCACTTGAAGAGAATCTTTTCTATGATCAGATCCGAATCATGTCGTACATGAGCAGGTTCCGTAAGATCCAGTAGAATAAAATAAGTG-AACTAGATAACCCATAATCTCGATTATGGGTTATCTAG-TTCACTTACATACGATTGAATAGTATGGAAATGCATTCATTTCCTATGCATTGACACGATCTATGATACTATCGGAGTGAAACAGGGGATCTAAGGAAGAACAGAGGCTAGGCTATATTAGTAACAAGTAAACCC-----TTTGTGTGTCTGTCAAAAGTCTCCAAGTATTTTGGGGATAAACACCGATCCTAAGGTCTGAGACGACCCAGAAAGCATT--TGATCCTATCATGATCCACCTTGTAAGCCTACTTGGGTATTGAGTATTTACTTGTAAGAACCAAATTTTTTGCGCTGGATAGTTGCAACTCCGGAAAAAGAAATCCAGTCA-------------AGTCAAATTTTTC------TTTGAAT--------AAAATCATTCATATTATCATATATGTTATGTGTGGATCTAGATAACATATAGATTTTATATGGATTCCTTATGGTTCTTTTTCTTTTTGCTCGAGCCGTATGATGAAAAATTATCATGTCCGGTTCCTTCGGGGGATGGAT-----CTATAAAAATTCACCTATCCCAATAACAAAAAAACCTGACCTGAATGATCCTGTATTAAGAGCTAAATTGGCTAAAGGTATGGGTCATAATTATTACGGAGAGCCCGCATGGCCCAATGATCTTTTATATATTTTTCCAGTAGAGATTG-----------------------AAG-----AAATCAATGCAATATTTTTTAGGAAAGATAAAACTGGATGAATTCAAATTCGTTCAAATGGG-----------------------ATTGGAAGGTTACTTATTTTCATTTAGGATGTTCGTTTTATTTCTTTCCTTAGGACTTTGGTGTAGTTTCTGCTCTCTCTCCTGGAATCG-AATTGTTGT-AACTGGACGCTTCTATCCT-CTAGCTAGTA--GGGATAG----------AAAAAATATTTTCATTTTTTTTTTTTAATGAATTCTTTCTCATTTATCCGATTTATCAAATTTGAAACAAAAAGATACATTTTTTTAATGAACACA----------AAAAAATCCTAAAGTT---------ATACTATACAGAAGGTTGTCAAAATGGAATCAATTAGTTTCCCCAATTCCTTA-----------------------ATTTTTACTAATGATCTTACA----TACGCCC-------TTCTATAGATAT------------------------ATAGAGAAACCC----------------------------ATTTTTCT-------TATTATA-----GATAAATAGGTTGATGGGGAAAATAAGACCCCGC-------CCTCGAAATGATAAAATCTACTAAAAAGAAAGGTAAAACCTTGTATCTTGTCTTTATTC---------------TTTTTTCAAAAACA--------TT-TTTTTCGAATTTAGAAATTTAGTAAACAGAAGCATTTTTATTCTACTTCCATTCCCTATTGTTTTCGGCC-------AATGAATAGGGAATGGGAATTATTCATTTGATTTTTAGATTAACAATGAAATCAGAC---------AGTCAAATCAATTGGGATTATTCCAACGTTTTATGA--------CTTATTTGTTTGTCGTACAAAAAAACCTTTTACATTCCCGGTAGGTCGTACAAAAAAAC-TTTTTGAATTCCCGGT-AGAAAG------------------------------------------------------------------------------------------------ATATAG-------ATATGTACAACTTTGACCAGCAATTTCATTTAGATC-TAAGTAAGGGCTCGAAAGAT-CCAATAGACAAAT-------------ATAAAGAAAAATAAAGAAGACCC-----CTTTGATTTTGTTCCCTTTATTCCCACGGCCTGGCCTGGTCAATACCTAGCCGGGCC----TTTTTTTGTTCCAACAAATCCTAGCTAAAAGAATTTAGCTG-------------CTTTGAACACAAAAATGCTTGCTATTAAAATGCTTGCTATTAAAGCAGC-------------------AATAAAAAGATGAGGGGTTATTTCCATTCTTACTTATTATTCCAT------------------TCTTACTTATTATATA--TATTATTTTTAA--------------------TATATATATAAATTATATAAAATCAAAGTATCCTTTCTTATT-----ATTCCTTCTTCCCTTTTGAGTTACTTGACGACCTTACGGGAAT--ATAAAATGAAACTG-----TGGGTTCTTA---AATAATAATGAATGCATTTTTCTGTTATGATTTCAGTGGTTTTAGTGAGCCATATCTATCAAAATCCCCCCAGCAAAAGAAAAAATAGAACTT--GTTATTTC----------------ATTTAGTTATTTTAAAGAGCCC-----TCCTTTCCGGAATCTCATTAAATTGAAATCCCCCGCGAA-AAACGT-CGACACTCTCATTTTCATGA-----TTATGATC-CTA--T-CTTTATTACGCTCAATTCCTCTGTT-CGACAAA---------------------GTATTAG-TATATCTAATACCTTACCCAGCCCATTTGGAAATTTTGGTTCAAACTCTTCGCTACTGGGTAAAAGATGCCCCCTCTTTGCATTTTTTACGATTCTTTCTTCACGACTATCGGCATTGGACTAGTCTTTTTATTCCAAAGAAAGCCAGTTCTTTTTTTTCAAAACGAAAG------------------CAAAGATTATTCTTCTTCCTATATAATTCTCATGTATGTGAATACGAATCCATCTTCGTCTTTCTCCGTAACCAATCTTCTCATTTACAATCAATATCTTCTGGAGCCCTTCTTGAACGAATATATTTCTATGAAAAAATAGAACATCTTGTCGAAGTCTTTGCTAAA-GATTTTCAAGGCAATCTATGGTTGTTCAAGGATCCTTTCATGCATTATGTTAGGTATCAAGGAAAGTCAATTCTCGCTTCAAAGGAGAACTTTCTTTTGATGCGTAAATGGAAATATTACTTTGTACGTTTCTGGCAATGTCATTTTTACCAGTGGTTTCAACCAGGAAGGATCTATATAAACCAATTATCCAAACATTCCCTCGACCTTCTGGGCTATCTTTCAAGTGTGCGGCTAAACCCTTTAACGATACGCAGTCAAATGCTAGAAAATTCATTTCTAATCGATAATGCTGTTAAGAAGTTCGATACCATTGTTCCAATTATTCCTTTGATTGGATCATTGGTTAAAGCGAAATTTTGTAACGTATTAGGGCATCCTGTTAGTAAGGTAGTTTGGGCAGATTTATCAGATTTAAGACATAAAAATTTACCCTACTTCTTTATCTTTCTTATGAAGGGCCTTTCATGAGTTGAATAGAGATTTTCA-TTTTTTATTCATCATTCGGGTTGATGAACTAAA-CCAGATAGTTATATGAGTGAAAGAAACAGCTTATAAGTTTGCAGTAAAAAGATTGAGTCTCATTTT-CTATGTAC-AAGAG-TTAAGTGAAAGTAACCATAAACATTA-GAAACGGTTTACCCCAAGATTGGTTAATTAGTGATCATGGCTTGAAGCGGGTGC-AAAAGATCAACTGTATGGGG-TTTTTACTATCTATTACCATACATGTATTACCCTAACGGGCGATTAGCAAAAAGAGGTGGATAGTTAGGAACACCAAGGTACACAAAGGATTCGTAATAGAGATTATGTAAGTTATTCAACAGAATTTTTCTGTGCATAA-AAGGAATTCTGATTGGGACTTTAAGTTGGTAGAAATGATGAAGAAGTACTCCCCCTGATTCCGATCCAGAGTATACTCCTATCCACCGATTAAGTAAATAACTATCAAGAACGAAGTAATCCTTTAACTTT------GTTTAAAGTCCCTTTTTCTGAGAAAGGAGAATAGGAACGAAAAAAATA-------AAATAG-AAAGA-ATAGAATTGCACTAG-----AAAGAAAGAGATCTTTTTTTATTCTTTCTTTCCTCTATTTAGAGAGTCTATTTAGAGAGATAGAATTCTTGTCATCATTCGTGAACTAATGCGATGCCTAATTGTTTTTCGTAATCGAAAATGCTAGGTTG--------AAATATCTATGAATA-TTGCTACAAGAAAGA-TTTTATTGAAAG-CTTAAGTTATCACTCAAC-AAAGAAAAATAAAAA-TTATTAAAAGAAAAGATAAGATCAA-TTCCG-AAGCGCTTTA-TTTTCAATATAGCAGACAGAATTCCATTGTCTAATTCGGG--ACTTTACGGTAGATTTAGATTCTATCTATCCTACGAATATAT-------------------CAAGATAAATAATAGCGAACTGGTCCTTAGA-TTTATTTGTGA-CCTTTGAGGAGCC-GTATGAGATGAAAATCTCATGTACGGTATTTAGATTA---------------------------------------------------------------------------------------------------------------------------------------------------------------------------------ATCAA------AATATTTACGATTTTTCTTT-AA-----------AGAAA--ATATGTAAGTCAA--------------------------------------------------------------------------------TGTGAAAT-AAAAAAGGAGCAATAGCTCCCT-CTTGATAGAACAACAAGGGGATTATTGCTCCTTTTTT-ATTTCTTTCAAATACTCGTATACACTAAGGCCAGGTCTTATCCATTTATAGATGGAGCTTCAAGAGCAGCTAGGTCTAGAGGGAAGTTATGAGCATTACGTTCATGCATAACTTCCATACCAAGGTTAGCGCGGTTAATGATATCAGCCCAAGTATTAATTACACGACCTTGACTATCAACTACAGATTGGTTGAAATTAAAACCATTTAGGTTGAAAGCCATAGTGCTGATACCTAAAGCAGTGAACCAGATACCTACTACCGGCCAAGCAGCTAGGAAGAAATGTAAAGAACGAGAGTTGTTGAAACTAGCATATTGGAAGATCAATCGGCCAAAATAACCATGAGCAGCTACAATATTATAAGTTTCTTCTTCTTGA-CCGAATC---TGTAACCTTCATTAGCAATAAATGCAAGAATATTTAC-TTCCATAATCTCATCGTTTTTTTACTTCAAAATAACT-CGGGATTTAATCCCATAGAGATAATAAATCTTTCGCCTGTCAATTCA----ATGAATTACCTCTCGATGATC-TTGAAATCGGATCAATATCATGAATAACAATATCTGAGCTATCAAATCAATTCGTCGTCGAGAATTGAATAGTATAACATAGAAAGATCTTT-TATCCATACCG------AATCCAA------------------------AATTTCTTTATTTATCAATCATTCTTTTCTGTTCTTTCTTTATCTACAACCTATCTTAGGTCCTCCTTGTACAATCATCGGATAAAGTATCGTTTGACCGCCCGTCCGTTTCCATTAGTCACAAACGCCCAACAAACAATAGAAGCGAAGTGGAAAAAGAAATAAGTTACGTTCTAAACTCCG----TTTTTTTAATGATCTAGTTTTCTTGGAAGACAAAGAAGTGTGATAAAGAGGAGTTCCGGGATAAAGGATCTAATATTCCATCAAACTAACTATTTGAGTTTGGGTTTTGTTCGTTCTTCGACGGG--CCCT--------AAAAAAAAATAG--AAAAAAATAGGAAGGAAAAAT-GATTTATTCCCCTGCTACTTGCTAAGCTAAAAAAGGGGTGGGATCTTTGATTGATCTTTATTTTTCTTTTA-CCCCCCCTTCCTT---------------------------------------------------------------------------------------------------------------------------------------------------------------------------------------------------------------------------------------------------------------------------------------------------------------AACGCTCTCAATAATTGTACTATTCTACATATGTCTTTCTCCTACCAATCAGTATTATTTGAAATAATGAAAATTCCCCTATTTGTTTGATGAGAAGTGC----GAAATGCCAAAGGAAA--GAAAAAAGAACCCCCTT-GGGAATG-AAATTCTGCTCCCCGTGCCCCCTTTAACAGAAAAGGGAAGATTACAAATTGACTTATTATACTCCTGACTATGAAACCAAAGATACTGATATCTTGGCAGCATT-TCGAGTAACTCCTCAACCTGGAGTTCCACCTGAAGAAGCAGGGGCCGCGGTAGCTGCCGAATCTTCAACTGGTACATGGACAACTGTGTGGACCGATGGACTTACCAGCCTTGATCGTTACAAAGGGCGATGCTACCACATCGAGCCCGTTGCTGGAGAAGAAACTCAATTTATTGCTTATGTAGCTTACCCATTAGACCTTTTTGAAGAAGGTTCTGTTACTAACATGTTTACTTCTATTGTGGGTAATGTATTTGGGTTCAAAGCCCTGCGCGCTCTACGTCTGGAAGATCTGCGAATCCCTGTCGCTTATGTTAAAACTTTCCAAGGCCCACCTCATGGCATCCAAGTTGAGAGAGATAAATTGAACAAGTATGGTCGCCCCCTGTTGGGATGTACTATTAAACCTAAATTGGGGTTATCTGCTAAAAACTATGGTAGAGCGGTTTATGAATGTCTACGTGGTGGACTTGATTTTACCAAAGATGATGAGAA--CGT--GAACTCCCAACCATTTATGCGTT---GGAGAGATCGATTC

Linnaea_borealis_subsp_borealis_BOP012344 -CTGAGCC-AATCCAGTTTTACGAAAACAAACAAGGGTTCAGAAAGCTAAAATC-AAAAAGGATAGGTGCAGAGACTCAATGGAAGCTGTTCTAACAAATGGAGTTGACTGTGTTGTGTTGGTAGAAAGAATCCTTCCATAGAAACTTCAGAAAGGATAAACGTATAAACATAGATATACGCATTGAAATACTAT-ATACTCTACCAAATGATTAATGACGACCCGAATCTGTATT--TA-----TATATATCAAAATGGGAGAATGGTTGTGAAGTGATTCCATATTGAAGAAAGAATCGAATATTCATTGATCAAATCATTCACTCCATAGTCTGATAGATC-TTTTGAAGAACTGATTAATTGGACGAGAATAAAGATAGAGTCCCATTCTACATGTCAATACCGGCAACAATGAAATTTATAGTAAGAGGAAAATCCGTCGACTTTAGAAATCGTGAGGGTTCAAGTCCCTCTATCCCCAAAAAACCCATATGGACTCCCTAATTATTTATCCTCTCCTT-TTATCC-TTTTTTGTTAGCGGTTAAAAATTCGTT-TCGTTATCTTTCTCATTCACCCTACTCTTTTACAAAGAGATCCGAGCGGAAATGTTTTTCTCTTATCACAAACAAGTCTTGTGATCTAAG----ATAA-TACGTGTACAAATGAACATCTTTGAGTAAGGAATCCCCATTTGAATGATTCATGGTCAA------TGTCATTATTCATACTGAAACTTACAAAGTCTTCCTT-TTGAAGATCCAAGAAATTCCAGGACCTGGATAAGACTTTGTAAGACCCTTTCAATTGACA-TAGACCCGAGTTATCTAGC--AAAATGAGGATGCAGCGGTATTTATGTTAATGCACTTCCCAATGATACGTAAACAAGGCATTTCTGGTCCTTTA--------TAGAGAAGATCTATCATAGATATTTGTAATCTATCATTTATCGCTTGGGGGAGGAACAATAGTATTTCATTGCTACAAGTATGGATTATTGAGAATAATAAGACATGTATTTGGACATTTCCCTTGAACTCCGCAATCTTTTTTATTTGACATGGATAGTTGAAGGGAATTTTCCGAAGAAAAAATGGATTAGATTATGGGAGTGTGTGACTTGAACTATTGATTGGTCTGTGCAGATATATGCCTTTCTCTATCTGCCACATTGGAATTCACAACCAAATGTGTCTTTGTTCCAACCATTGCGTAAGCCC----------CATACAGAGGATAGGCTGGTTCACTTGAAGAGAATCTTTTCTATGATCAGATCCGAATCATGTCGTACATGAGCAGGTTCCGTAAGATCCAGTAGAATAAAATAAGTG-AACTAGATAACCCATAATCTCGATTATGGGTTATCTAG-TTCACTTACATACGATTGAATAGTATGGAAATGCATTCATTTCCTATGCATTGACACGATCTATGATACTATCGGAGTGAAACAGGGGATCTAAGGAAGAACAGAGGCTAGGCTATATTAGTAACAAGTAAACCC-----TTTGTGTGTCTGTCAAAAGTCTCCAAGTATTTTGGGGATAAACACCGATCCTAAGGTCTGAGACGACCCAGAAAGCATT--TGATCCTATCATGATCCACCTTGTAAGCCTACTTGGGTATTGAGTATTTACTTGTAAGAACCAAATTTTTTGCGCTGGATAGTTGCAACTCCGGAAAAAGAAATCCAGTCA-------------AGTCAAATTTTTC------TTTGAAT--------AAAATCATTCATATTATCATATATGTTATGTGTGGATCTAGATAACATATAGATTTTATATGGATTCCTTATGGTTCTTTTTCTTTTTGCTCGAGCCGTATGATGAAAAATTATCATGTCCGGTTCCTTCGGGGGATGGAT-----CTATAAAAATTCACCTATCCCAATAACAAAAAAACCTGACCTGAATGATCCTGTATTAAGAGCTAAATTGGCTAAAGGTATGGGTCATAATTATTACGGAGAGCCCGCATGGCCCAATGATCTTTTATATATTTTTCCAGTAGAGATTG-----------------------AAG-----AAATCAATGCAATATTTTTTAGGAAAGATAAAACTGGATGAATTCAAATTCGTTCAAATGGG-----------------------ATTGGAAGGTTACTTATTTTCATTTAGGATGTTCGTTTTATTTCTTTCCTTAGGACTTTGGTGTAGTTTCTGCTCTCTCTCCTGGAATCG-AATTGTTGT-AACTGGACGCTTCTATCCT-CTAGCTAGTA--GGGATAG----------AAAAAATATTTTCATTTTTTTTTTTTAATGAATTCTTTCTCATTTATCCGATTTATCAAATTTGAAACAAAAAGATACATTTTTTTAATGAACACA----------AAAAAATCCTAAAGTT---------ATACTATACAGAAGGTTGTCAAAATGGAATCAATTAGTTTCCCCAATTCCTTA-----------------------ATTTTTACTAATGATCTTACA----TACGCCC-------TTCTATAGATAT------------------------ATAGAGAAACCC----------------------------ATTTTTCT-------TATTATA-----GATAAATAGGTTGATGGGGAAAATAAGACCCCGC-------CCTCGAAATGATAAAATCTACTAAAAAGAAAGGTAAAACCTTGTATCTTGTCTTTATTC---------------TTTTTTCAAAAACA--------TT-TTTTTCGAATTTAGAAATTTAGTAAACAGAAGCATTTTTATTCTACTTCCATTCCCTATTGTTTTCGGCC-------AATGAATAGGGAATGGGAATTATTCATTTGATTTTTAGATTAACAATGAAATCAGAC---------AGTCAAATCAATTGGGATTATTCCAACGTTTTATGA--------CTTATTTGTTTGTCGTACAAAAAAACCTTTTACATTCCCGGTAGGTCGTACAAAAAAAC-TTTTTGAATTCCCGGT-AGAAAG------------------------------------------------------------------------------------------------ATATAG-------ATATGTACAACTTTGACCAGCAATTTCATTTAGATC-TAAGTAAGGGCTCGAAAGAT-CCAATAGACAAAT-------------ATAAAGAAAAATAAAGAAGACCC-----CTTTGATTTTGTTCCCTTTATTCCCACGGCCTGGCCTGGTCAATACCTAGCCGGGCC----TTTTTTTGTTCCAACAAATCCTAGCTAAAAGAATTTAGCTG-------------CTTTGAACACAAAAATGCTTGCTATTAAAATGCTTGCTATTAAAGCAGC-------------------AATAAAAAGATGAGGGGTTATTTCCATTCTTACTTATTATTCCAT------------------TCTTACTTATTATATA--TATTATTTTTAA--------------------TATATATATAAATTATATAAAATCAAAGTATCCTTTCTTATT-----ATTCCTTCTTCCCTTTTGAGTTACTTGACGACCTTACGGGAAT--ATAAAATGAAACTG-----TGGGTTCTTA---AATAATAATGAATGCATTTTTCTGTTATGATTTCAGTGGTTTTAGTGAGCCATATCTATCAAAATCCCCCCAGCAAAAGAAAAAATAGAACTT--GTTATTTC----------------ATTTAGTTATTTTAAAGAGCCC-----TCCTTTCCGGAATCTCATTAAATTGAAATCCCCCGCGAA-AAACGT-CGACACTCTCATTTTCATGA-----TTATGATC-CTA--T-CTTTATTACGCTCAATTCCTCTTTTCCGACAAAAA-------------------GTATTAG-TATATCTAATACCTTACCCAGCCCATTTGGAAATTTTGGTTCAAACTCTTCGCTACTGGGTAAAAGATGCCCCCTCTTTGCATTTTTTACGATTCTTTCTTCACGACTATCGGCATTGGACTAGTCTTTTTATTCCAAAGAAAGCCAGTTCTTTTTTTTCAAAACGAAAG------------------CAAAGATTATTCTTCTTCCTATATAATTCTCATGTATGTGAATACGAATCCATCTTCGTCTTTCTCCGTAACCAATCTTCTCATTTACAATCAACATCTTCTGGAGCCCTTCTTGAACGAATATATTTCTATGAAAAAATAGAACATCTTGTCGAAGTCTTTGCTAAA-GATTTTCAAGGCAATCTATGGTTGTTCAAGGATCCTTTCATGCATTATGTTAGGTATCAAGGAAAGTCAATTCTCGCTTCAAAGGAGAACTTTCTTTTGATGCGTAAATGGAAATATTACTTTGTACGTTTCTGGCAATGTCATTTTTACCAGTGGTTTCAACCAGGAAGGATCTATATAAACCAATTATCCAAACATTCCCTCGACCTTCTGGGCTATCTTTCAAGTGTGCGGCTAAACCCTTTAACGATACGCAGTCAAATGCTAGAAAATTCATTTCTAATCGATAATGCTGTTAAGAAGTTCGATACCATTGTTCCAATTATTCCTTTGATTGGATCATTGGTTAAAGCGAAATTTTGTAACGTATTAGGGCATCCTGTTAGTAAGGTAGTTTGGGCAGATT-ATCAGATTTAAGACAT-AAAATTTACCCTACTTCTTTATCTTTC-TATGAAGGGCCTTTCATGAGTTGAATAGAGATTTTCATTTTTTTATTCATCATTCGGGTTGATGAACTAAA-CCAGATAGTTATATGAGTGAAAGAAACAGCTTATAAGTTTGCAGTAAAAAGATTGAGTCTCATTTT-CTATGTAC-AAGAGTTTAAGTGAAAGTAACCATAAACATTATGAAACGGTTTACCCCAAGATTGGTTAATTAGTGATCATGGCTTGAAGCGGGTGCAAAAAGATCAACTGTATGGGG-TTTTTACTATCTATTACCATACATGTATTACCCTAACGGGCGATTAGCAAAAAGAGGTGGATAGTTAGGAACACCAAGGTACACAAAGGATTCGTAATAGAGATTATGTAAGTTATTCAACAGAATTTTTCTGTGCATAA-AAGGAATTCTGATTGGGACTTTAAGTTGGTAGAAATGATGAAGAAGTACTCCCCCTGATTCCGATCCAGAGTATACTCCTATCCACCGATTAAGTAAATAACTATCAAGAACGAAGTAATCCTTTAACTTT------GTTTAAAGTCCCTTTTTCTGAGAAAGGAGAATAGGAACGAAAAAAATA-------AAATAG-AAAGA-ATAGAATTGCACTAG-----AAAGAAAGAGATCTTTTTTTATTCTTTCTTTCC-------------TCTATTTAGAGAGATAGAATTCTTGTCATCATTCGTGAACTAATGCGATGCCTAATTGTTTTTCGTAATCGAAAATGCTAGGTTG--------AAATATCTATGAATA-TTGCTACAAGAAAGATTTTTATTGAAAGCTTTAAGTTATCACTCAAC-AAAGAAAAATAAAAA-TTATTAAAAGAAAAGATAAGATCAATTTCCG-AAGCGCTTTA-TTTTCAATATAGCAGACAGAATTCCATTGTCTAATTCGGG--ACTTTACGGTAGATTTAGATTCTATCTATCCTACGAATATAT-------------------CAAGATAAATAATAGCGAACTGGTCCTTAGA-TTTATTTGTGA-CCTTTGAGGAGCC-GTATGAGATGAAAATCTCATGTACGGTATTTAGATTA---------------------------------------------------------------------------------------------------------------------------------------------------------------------------------ATCAA------AATATTTACGATTTTTCTTT-AA-----------AGAAA--ATATGTAAGTCAA--------------------------------------------------------------------------------TGTGAAAT-AAAAAAGGAGCAATAGCTCCCT-CTTGATAGAACAACAAGGGGATTATTGCTCCTTTTTT-ATTTCTTTCAAATACTCGTATACACTAAGGCCAGGTCTTATCCATTTATAGATGGAGCTTCAAGAGCAGCTAGGTCTAGAGGGAAGTTATGAGCATTACGTTCATGCATAACTTCCATACCAAGGTTAGCGCGGTTAATGATATCAGCCCAAGTATTAATTACACGACCTTGACTATCAACTACAGATTGGTTGAAATTAAAACCATTTAGGTTGAAAGCCATAGTGCTGATACCTAAAGCAGTGAACCAGATACCTACTACCGGCCAAGCAGCTAGGAAGAAATGTAAAGAACGAGAGTTGTTGAAACTAGCATATTGGAAGATCAATCGGCCAAAATAACCATGAGCAGCTACAATATTATAAGTTTCTTCTTCTTGA-CCGAATC---TGTAACCTTCATTAGCAATAAATGCAAGAATATTTAC-TTCCATAATCTCATCGTTTTTTTACTTCAAAATAACT-CGGGATTTAATCCCATAGAGATAATAAATCTTTCGCCTGTCAATTCA----ATGAATTACCTCTCGATGATC-TTGAAATCGGATCAATATCATGAATAACAATATCTGAGCTATCAAATCAATTCGTCGTCGAGAATTGAATAGTATAACATAGAAAGATCTTT-TATCCATACCG------AATCCAA------------------------AATTTCTTTATTTATCAATCATTCTTTTCTGTTCTTTCTTTATCTACAACCTATCTTAGGTCCTCCTTGTACAATCATCGGATAAAGTATCGTTTGACCGCCCGTCCGTTTCCATTAGTCACAAACGCCCAACAAACAATAGAAGCGAAGTGGAAAAAGAAATAAGTTACGTTCTAAACTCCG----TTTTTTTAATGATCTAGTTTTCTTGGAAGACAAAGAAGTGTGATAAAGAGGAGTTCCGGGATAAAGGATCTAATATTCCATCAAACTAACTATTTGAGTTTGGGTTTTGTTCGTTCTTCGACGGG--CCCT--------AAAAAAAAATAG--AAAAAAATAGGAAGGAAAAAT-GATTTATTCCCCTGCTACTTGCTAAGCTAAAAAAGGGGTGGGATCTTTGATTGATCTTTATTTTTCTTTTA-CCCCCCCTTCCTT---------------------------------------------------------------------------------------------------------------------------------------------------------------------------------------------------------------------------------------------------------------------------------------------------------------AACGCTCTCAATAATTGTACTATTCTACATATGTCTTTCTCCTACCAATCAGTATTATTTGAAATAATGAAAATTCCCCTATTTGTTTGATGAGAAGTGC----GAAATGCCAAAGGAAA--GAAAAAAGAACCCCCTT-GGGAATG-AAATTCTGCTCCCCGTGCCCCCTTTAACAGAAAAGGG------------------------------------------------------------CAGCATT-TCCGGTAACTCCTCAACCTGGAGTTCCACCTGAAGAAGCAGGGGCCGCGGTAGCTGCCGAATCTTCAACTGGTACATGGACAACTGTGTGGACCGATGGACTTACCAGCCTTGATCGTTACAAAGGGCGATGCTACCACATCGAGCCCGTTGCTGGAGAAGAAACTCAATTTATTGCTTATGTAGCTTACCCATTAGACCTTTTTGAAGAAGGTTCTGTTACTAACATGTTTACTTCTATTGTGGGTAATGTATTTGGGTTCAAAGCCCTGCGCGCTCTACGTCTGGAAGATCTGCGAATCCCTGTCGCTTATGTTAAAACTTTCCAAGGCCCACCTCATGGCATCCAAGTTGAGAGAGATAAATTGAACAAGTATGGTCGCCCCCTGTTGGGATGTACTATTAAACCTAAATTGGGGTTATCTGCTAAAAACTATGGTAGAGCGGTTTATGAATGTCTACGTGGTGGAC------------------------------------------------------------------------

Linnaea_borealis_subsp_borealis_BOP022789 CCTGAGCCAAATCCAGTTTTACGAAAACAAACAAGGGTTCAGAAAGCTAAAATC-AAAAAGGATAGGTGCAGAGACTCAATGGAAGCTGTTCTAACAAATGGAGTTGACTGTGTTGTGTTGGTAGAAAGAATCCTTCCATAGAAACTTCAGAAAGGATAAACGTATAAACATAGATATACGCATTGAAATACTAT-ATACTCTACCAAATGATTAATGACGACCCGAATCTGTATT--TA-----TATATATCAAAATGGGAGAATGGTTGTGAAGTGATTCCATATTGAAGAAAGAATCGAATATTCATTGATCAAATCATTCACTCCATAGTCTGATAGATC-TTTTGAAGAACTGATTAATTGGACGAGAATAAAGATAGAGTCCCATTCTACATGTCAATACCGGCAACAATGAAATTTATAGTAAGAGGAAAATCCGTCGACTTTAGAAATCGTGAGGGTTCAAGTCCCTCTATCCCCAAAAAACCCATATGGACTCCCTAATTATTTATCCTCTCCTT-TTATCC-TTTTTTGTTAGCGGTTAAAAATTCGTT-TCGTTATCTTTCTCATTCACCCTACTCTTTTACAAAGAGATCCGAGCGGAAATGTTTTTCTCTTATCACAAACAAGTCTTGTGATCTAAG----ATAA-TACGTGTACAAATGAACATCTTTGAGTAAGGAATCCCCATTTGAATGATTCATGGTCAA------TGTCATTATTCATACTGAAACTTACAAAGTCTTCCTT-TTGAAGATCCAAGAAATTCCAGGACCTGGATAAGACTTTGTAAGACCCTTTCAATTGACA-TAGACCCGAGTTATCTAGCCAAAAATGAGGATGCAGCGGTATTTATGTTAATGCACTTCCCAATGATACGTAAACAAGGCATTTCTGGTCCTTTA--------TAGAGAAGATCTATCATAGATATTTGTAATCTATCATTTATCGCTTGGGGGAGGAACAATAGTATTTCATTGCTACAAGTATGGATTATTGAGAATAATAAGACATGTATTTGGACATTTCCCTTGAACTCCGCAATCTTTTTTATTTGACATGGATAGTTGAAGGGAATTTTCCGAAGAAAAAATGGATTAGATTATGGGAGTGTGTGACTTGAACTATTGATTGGTCTGTGCAGATATATGCCTTTCTCTATCTGCCACATTGGAATTCACAACCAAATGTGTCTTTGTTCCAACCATTGCGTAAGCCC----------CATACAGAGGATAGGCTGGTTCACTTGAAGAGAATCTTTTCTATGATCAGATCCGAATCATGTCGTACATGAGCAGGTTCCGTAAGATCCAGTAGAATAAAATAAGTG-AACTAGATAACCCATAATCGAGATTATGGGTTATCTAG-TTCACTTACATACGATTGAATAGTATGGAAATGCATTCATTTCCTATGCATTGACACGATCTATGATACTATCGGAGTGAAACAGGGGATCTAAGGAAGAACAGAGGCTAGGCTATATTAGTAACAAGTAAACCC-----TTTGTGTGTCTGTCAAAAGTCTCCAAGTATTTTGGGGATAAACACCGATCCTAAGGTCTGAGACGACCCAGAAAGCATT--TGATCCTATCATGATCCACCTTGTAAGCCTACTTGGGTATTGAGTATTTACTTGTAAGAACCAAATTTTTTGCGCTGGATAGTTGCAACTCCGGAAAAAGAAATCCAGTCA-------------AGTCAAATTTTTC------TTTGAAT--------AAAATCATTCATATTATCATATATGTTATGTGTGGATCTAGATAACATATAGATTTTATATGGATTCCTTATGGTTCTTTTTCTTTTTGCTCGAGCCGTATGATGAAAAATTATCATGTCCGGTTCCTTCGGGGGATGGAT-----CTATAAAAATTCACCTATCCCAATAACAAAAAAACCTGACCTGAATGATCCTGTATTAAGAGCTAAATTGGCTAAAGGTATGGGTCATAATTATTACGGAGAGCCCGCATGGCCCAATGATCTTTTATATATTTTTCCAGTAGAGATTG-----------------------AAG-----AAATCAATGCAATATTTTTTAGGAAAGATAAAACTGGATGAATTCAAATTCGTTCAAATGGG-----------------------ATTGGAAGGTTACTTATTTTCATTTAGGATGTTCGTTTTATTTCTTTCCTTAGGACTTTGGTGTAGTTTCTGCTCTCTCTCCTGGAATCG-AATTGTTGT-AACTGGACGCTTCTATCCT-CTAGCTAGTA--GGGATAG----------AAAAAATATTTTCATTTTTTTTTTTTAATGAATTCTTTCTCATTTATCCGATTTATCAAATTTGAAACAAAAAGATACATTTTTTTAATGAACACA----------AAAAAATCCTAAAGTT---------ATACTATACAGAAGGTTGTCAAAATGGAATCAATTAGTTTCCCCAATTCCTTA-----------------------ATTTTTACTAATGATCTTACA----TACGCCC-------TTCTATAGATAT------------------------ATAGAGAAACCC----------------------------ATTTTTCT-------TATTATA-----GATAAATAGGTTGATGGGGAAAATAAGACCCCGC-------CCTCGAAATGATAAAATCTACTAAAAAGAAAGGTAAAACCTTGTATCTTGTCTTTATTC---------------TTTTTTCAAAAACA--------TT-TTTTTCGAATTTAGAAATTTAGTAAACAGAAGCATTTTTATTCTACTTCCATTCCCTATTGTTTTCGGCC-------AATGAATAGGGAATGGGAATTATTCATTTGATTTTTAGATTAACAATGAAATCAGAC---------AGTCAAATCAATTGGGATTATTCCAACGTTTTATGA--------CTTATTTGTTTGTCGTACAAAAAAACCTTTTACATTCCCGGTAGGTCGTACAAAAAAAC-TTTTTGAATTCCCGGT-AGAAAG------------------------------------------------------------------------C--TTGCCTTTCTCTTTCTTTATGATATAG-------ATATGTACAACTTTGACCAGCAATTTCATTTAGATC-TAAGTAAGGGCTCGAAAGAT-CCAATAGACAAAT-------------ATAAAGAAAAATAAAGAAGACCC-----CTTTGATTTTGTTCCCTTTATTCCCACGGCCTGGCCTGGTCAATACCTAGCCGGGCC----TTTTTTTGTTCCAACAAATCCTAGCTAAAAGAATTTAGCTG-------------CTTTGAACACAAAAATGCTTGCTATTAAAATGCTTGCTATTAAAGCAGC-------------------AATAAAAAGATGAGGGGTTATTTCCATTCTTACTTATTATTCCAT------------------TCTTACTTATTATATA--TATTATTTTTAA--------------------TATATATATAAATTATATAAAATCAAAGTATCCTTTCTTATT-----ATTCCTTCTTCCCTTTTGAGTTACTTGACGACCTTACGGGAAT--ATAAAATGAAACTG-----TGGGTTCTTA---AATAATAATGAATGCATTTTTCTGTTATGATTTCAGTGGTTTTAGTGAGCCATATCTATCAAAATCCCCCCAGCAAAAGAAAAAATAGAACTT--GTTATTTC----------------ATTTAGTTATTTTAAAGAGCCC-----TCCTTTCCGGAATCTCATTAAATTGAAATCCCCCGCGAA-AAACGT-CGACACTCTCATTTTCATGA-----TTATGA----------------------------------------------------------------GTATTAG-TATATCTAATACCTTACCCAGCCCATTTGGAAATTTTGGTTCAAACTCTTCGCTACTGGGTAAAAGATGCCCCCTCTTTGCATTTTTTACGATTCTTTCTTCACGACTATCGGCATTGGACTAGTCTTTTTATTCCAAAGAAAGCCAGTTCTTTTTTTTCAAAACGAAAG------------------CAAAGATTATTCTTCTTCCTATATAATTCTCATGTATGTGAATACGAATCCATCTTCGTCTTTCTCCGTAACCAATCTTCTCATTTACAATCAATATCTTCTGGAGCCCTTCTTGAACGAATATATTTCTATGAAAAAATAGAACATCTTGTCGAAGTCTTTGCTAAA-GATTTTCAAGGCAATCTATGGTTGTTCAAGGATCCTTTCATGCATTATGTTAGGTATCAAGGAAAGTCAATTCTCGCTTCAAAGGAGAACTTTCTTTTGATGCGTAAATGGAAATATTACTTTGTACGTTTCTGGCAATGTCATTTTTACCAGTGGTTTCAACCAGGAAGGATCTATATAAACCAATTATCCAAACATTCCCTCGACCTTCTGGGCTATCTTTCAAGTGTGCGGCTAAACCCTTTAACGATACGCAGTCAAATGCTAGAAAATTCATTTCTAATCGATAATGCTGTTAAGAAGTTCGATACCATTGTTCCAATTATTCCTTTGATTGGATCATTGGTTAAAGCGAAATTTTGTAACGTATTAGGGCATCCTGTTAGTAAGGTAGTTTGGGCAGATTTATCAGATTTAAGACAT-AAAATTTACCCTACTTCTTTATCTTTC-TATGAAGGGCCTTTCATGAGTTGAATAGAGATTTTCA-TTTTTTATTCATCATTCGGGTTGATGAACTAAA-CCAGATAGTTATATGAGTGAAAGAAACAGCTTATAAGTTTGCAGTAAAAAGATTGAGTCTCATTTT-CTATGTAC-AAGAG-TTAAGTGAAAGTAACCATAAACATTA-GAAACGGTTTACCCCAAGATTGGTTAATTAGTGATCATGGCTTGAAGCGGGTGC-AAAAGATCAACTGTATGGGG-TTTTTACTATCTATTACCATACATGTATTACCCTAACGGGCGATTAGCAAAAAGAGGTGGATAGTTAGGAACACCAAGGTACACAAAGGATTCGTAATAGAGATTATGTAAGTTATTCAACAGAATTTTTCTGTGCATAA-AAGGAATTCTGATTGGGACTTTAAGTTGGTAGAAATGATGAAGAAGTACTCCCCCTGATTCCGATCCAGAGTATACTCCTATCCACCGATTAAGTAAATAACTATCAAGAACGAAGTAATCCTTTAACTTT------GTTTAAAGTCCCTTTTTCTGAGAAAGGAGAATAGGAACGAAAAAAATA-------AAATAG-AAAGA-ATAGAATTGCACTAG-----AAAGAAAGAGATCTTTTTTTATTCTTTCTTTCC-------------TCTATTTATAGAGATAGAATTCTTGTCATCATTCGTGAACTAATGCGATGCCTAATTGTTTTTCGTAATCGAAAATGCTAGGTTG--------AAATATCTATGAATA-TTGCTACAAGAAAGA-TTTTATTGAAAG-CTTAAGTTATCACTCAAC-AAAGAAAAATAAAAA-TTATTAAAAGAAAAGATAAGATCAA-TTCCG-AAGCGCTTTA-TTTTCAATATAGCAGACAGAATTCCATTGTCTAATTCGGG--ACTTTACGGTAGATTTAGATTCTATCTATCCTACGAATATAT-------------------CAAGATAAATAATAGCGAACTGGTCCTTAGA-TTTATTTGTGA-CCTTTGAGGAGCC-GTATGAGATGAAAATCTCATGTACG-TATTTAGATTA---------------------------------------------------------------------------------------------------------------------------------------------------------------------------------ATCAA------AATATTAACGATTTTTCTTT-AA-----------AGAAA--ATATGTAAGTCAA--------------------------------------------------------------------------------TGTGAAAT-AAAAAAGGAGCAATAGCTCCCT-CTTGATAGAACAACAAGGGGATTATTGCTCCTTTTTT-ATTTCTTTCAAATACTCGTATACACTAAGGCCAGGTCTTATCCATTTATAGATGGAGCTTCAAGAGCAGCTAGGTCTAGAGGGAAGTTATGAGCATTACGTTCATGCATAACTTCCATACCAAGGTTAGCGCGGTTAATGATATCAGCCCAAGTATTAATTACACGACCTTGACTATCAACTACAGATTGGTTGAAATTAAAACCATTTAGGTTGAAAGCCATAGTGCTGATACCTAAAGCAGTGAACCAGATACCTACTACCGGCCAAGCAGCTAGGAAGAAATGTAAAGAACGAGAGTTGTTGAAACTAGCATATTGGAAGATCAATCGGCCAAAATAACCATGAGCAGCTACAATATTATAAGTTTCTTCTTCTTGA-CCGAATC---TGTAACCTTCATTAGCAATAAATGCAAGAATATTTAC-TTCCATAATCTCATCGTTTTTTTACTTCAAAATAACT-CGGGATTTAATCCCATAGAGATAATAAATCTTTCGCCTGTCAATTCA----ATGAATTACCTCTCGATGATC-TTGAAATCGGATCAATATCATGAATAACAATATCTGAGCTATCAAATCAATTCGTCGTCGAGAATTGAATAGTATAACATAGAAAGATCTTT-TATCCATACCG------AATCCAA------------------------AATTTCTTTATTTATCAATCATTCTTTTCTGTTCTTTCTTTATCTACAACCTATCTTAGGTCCTCCTTGTACAATCATCGGATAAAGTATCGTTTGACCGCCCGTCCGTTTCCATTAGTCACAAACGCCCAACAAACAATAGAAGCGAAGTGGAAAAAGAAATAAGTTACGTTCTAAACTCCG----TTTTTTTAATGATCTAGTTTTCTTGGAAGACAAAGAAGTGTGATAAAGAGGAGTTCCGGGATAAAGGATGTAATATTCCATCAAACTAACTATTTGAGTTTGGGTTTTGTTCGTTCTTCGACGGG--CCCT--------AAAAAAAAATAG--AAAAAAATAGGAAGGAAAAAT-GATTTATTCCCCTGCTACTTGCTAAGCTAAAAAAGGGGTGGGATCTTTGATTGATCTTTATTTTTCTTTTA-CCCCCCCTTCCTT---------------------------------------------------------------------------------------------------------------------------------------------------------------------------------------------------------------------------------------------------------------------------------------------------------------AACGCTCTCAATAATTGTACTATTCTACATATGTCTTTCTCCTACCAATCAGTATTATTTGAAATAATGAAAATTCCCCTATTTGTTTGATGAGAAGTGC----GAAATGCCAAAGGAAA--GAAAAAAGAACCCCCTT-GGGAATG-AAATTCTGCTCCCCGTGCCCCCTTTAACAGAAAAGGG------------------------------------------------------------------------GTAACTCCTCAACCTGGAGTTCCACCTGAAGAAGCAGGGGCCGCGGTAGCTGCCGAATCTTCAACTGGTACATGGACAACTGTGTGGACCGATGGACTTACCAGCCTTGATCGTTACAAAGGGCGATGCTACCACATCGAGCCCGTTGCTGGAGAAGAAACTCAATTTATTGCTTATGTAGCTTACCCATTAGACCTTTTTGAAGAAGGTTCTGTTACTAACATGTTTACTTCTATTGTGGGTAATGTATTTGGGTTCAAAGCCCTGCGCGCTCTACGTCTGGAAGATCTGCGAATCCCTGTCGCTTATGTTAAAACTTTCCAAGGCCCACCTCATGGCATCCAAGTTGAGAGAGATAAATTGAACAAGTATGGTCGCCCCCTGTTGGGATGTACTATTAAACCTAAATTGGGGTTATCTGCTAAAAACTATGGTAGAGCGGTTTATGAATGTCTACGTGGTGGACTTGATTTTACCAAAGATGATGAGAAACAGT--GAACTCCCAACCATTTATGC--------------------

Linnaea_borealis_subsp_longiflora_BOP022790 CCTGAGCCAAATCCAGTTTTACGAAAACAAACAAGGGTTCAGAAAGCTAAAATC-AAAAAGGATAGGTGCAGAGACTCAATGGAAGCTGTTCTAACAAATGGAGTTGACTGTGTTGTGTTGGTAGAAAGAATCCTTCCATAGAAACTTCAGAAAGGATAAACGTATAAACATAGATATACGCATTGAAATACTAT-ATACTCTACCAAATGATTAATGACGACCCGAATCTGTATT--TA-----TATATATCAAAATGGGAGAATGGTTGTGAAGTGATTCCATATTGAAGAAAGAATCGAATATTCATTGATCAAATCATTCACTCCATAGTCTGATAGATC-TTTTGAAGAACTGATTAATTGGACGAGAATAAAGATAGAGTCCCATTCTACATGTCAATACCGGCAACAATGAAATTTATAGTAAGAGGAAAATCCGTCGACTTTAGAAATCGTGAGGGTTCAAGTCCCTCTATCCCCAAAAAACCCATATGGACTCCCTAATTATTTATCCTCTCCTT-TTATCC-TTTTTTGTTAGCGGTTAAAAATTCGTT-TCGTTATCTTTCTCATTCACCCTACTCTTTTACAAAGAGATCCGAGCGGAAATGTTTTTCTCTTATCACAAACAAGTCTTGTGATCTAAG----ATAA-TACGTGTACAAATGAACATCTTTGAGTAAGGAATCCCCATTTGAATGATTCATGGTCAA------TGTCATTATTCATACTGAAACTTACAAAGTCTTCCTT-TTGAAGATCCAAGAAATTCCAGGACCTGGATAAGACTTTGTAAGACCCTTTCAATTGACA-TAGACCCGAGTTATCTAGCCAAAAATGAGGATGCAGCGGTATTTATGTTAATGCACTTCCCAATGATACGTAAACAAGGCATTTCTGGTCCTTTA--------TAGAGAAGATCTATCATAGATATTTGTAATCTATCATTTATCGCTTGGGGGAGGAACAATAGTATTTCATTGCTACAAGTATGGATTATTGAGAATAATAAGACATGTATTTGGACATTTCCCTTGAACTCCGCAATCTTTTTTATTTGACATGGATAGTTGAAGGGAATTTTCCGAAGAAAAAATGGATTAGATTATGGGAGTGTGTGACTTGAACTATTGATTGGTCTGTGCAGATATATGCCTTTCTCTATCTGCCACATTGGAATTCACAACCAAATGTGTCTTTGTTCCAACCATTGCGTAAGCCC----------CATACAGAGGATAGGCTGGTTCACTTGAAGAGAATCTTTTCTATGATCAGATCCGAATCATGTCGTACATGAGCAGGTTCCGTAAGATCCAGTAGAATAAAATAAGTG-AACTAGATAACCCATAATCTCGATTATGGGTTATCTAG-TTCACTTACATACGATTGAATAGTATGGAAATGCATTCATTTCCTATGCATTGACACGATCTATGATACTATCGGAGTGAAACAGGGGATCTAAGGAAGAACAGAGGCTAGGCTATATTAGTAACAAGTAAACCC-----TTTGTGTGTCTGTCAAAAGTCTCCAAGTATTTTGGGGATAAACACCGATCCTAAGGTCTGAGACGACCCAGAAAGCATT--TGATCCTATCATGATCCACCTTGTAAGCCTACTTGGGTATTGAGTATTTACTTGTAAGAACCAAATTTTTTGCGCTGGATAGTTGCAACTCCGGAAAAAGAAATCCAGTCA-------------AGTCAAATTTTTC------TTTGAAT--------AAAATCATTCATATTATCATATATGTTATGTGTGGATCTAGATAACATATAGATTTTATATGGATTCCTTATGGTTCTTTTTCTTTTTGCTCGAGCCGTATGATGAAAAATTATCATGTCCGGTTCCTTCGGGGGATGGAT-----CTATAAAAATTCACCTATCCCAATAACAAAAAAACCTGACCTGAATGATCCTGTATTAAGAGCTAAATTGGCTAAAGGTATGGGTCATAATTATTACGGAGAGCCCGCATGGCCCAATGATCTTTTATATATTTTTCCAGTAGAGATTG-----------------------AAG-----AAATCAATGCAATATTTTTTAGGAAAGATAAAACTGGATGAATTCAAATTCGTTCAAATGGG-----------------------ATTGGAAGGTTACTTATTTTCATTTAGGATGTTCGTTTTATTTCTTTCCTTAGGACTTTGGTGTAGTTTCTGCTCTCTCTCCTGGAATCG-AATTGTTGT-AACTGGACGCTTCTATCCT-CTAGCTAGTA--GGGATAG----------AAAAAATATTTTCATTTTTTTTTTTTAATGAATTCTTTCTCATTTATCCGATTTATCAAATTTGAAACAAAAAGATACATTTTTTTAATGAACACA----------AAAAAATCCTAAAGTT---------ATACTATACAGAAGGTTGTCAAAATGGAATCAATTAGTTTCCCCAATTCCTTA-----------------------ATTTTTACTAATGATCTTACA----TACGCCC-------TTCTATAGATAT------------------------ATAGAGAAACCC----------------------------ATTTTTCT-------TATTATA-----GATAAATAGGTTGATGGGGAAAATAAGACCCCGC-------CCTCGAAATGATAAAATCTACTAAAAAGAAAGGTAAAACCTTGTATCTTGTCTTTATTC---------------TTTTTTCAAAAACA--------TT-TTTTTCGAATTTAGAAATTTAGTAAACAGAAGCATTTTTATTCTACTTCCATTCCCTATTGTTTTCGGCC-------AATGAATAGGGAATGGGAATTATTCATTTGATTTTTAGATTAACAATGAAATCAGAC---------AGTCAAATCAATTGGGATTATTCCAACGTTTTATGA--------CTTATTTGTTTGTCGTACAAAAAAACCTTTTACATTCCCGGTAGGTCGTACAAAAAAAC-TTTTTGAATTCCCGGT-AGAAAG----------------------------------------------------------------------GTC-TTGG-CTTTCTCTTTCTTTATGATATAG-------ATATGTACAACTTTGACCAGCAATTTCATTTAGATC-TAAGTAAGGGCTCGAAAGAT-CCAATAGACAAAT-------------ATAAAGAAAAATAAAGAAGACCC-----CTTTGATTTTGTTCCCTTTATTCCCACGGCCTGGCCTGGTCAATACCTAGCCGGGCC----TTTTTTTGTTCCAACAAATCCTAGCTAAAAGAATTTAGCTG-------------CTTTGAACACAAAAATGCTTGCTATTAAAATGCTTGCTATTAAAGCAGC-------------------AATAAAAAGATGAGGGGTTATTTCCATTCTTACTTATTATTCCAT------------------TCTTACTTATTATATA--TATTATTTTTAA--------------------TATATATATAAATTATATAAAATCAAAGTATCCTTTCTTATT-----ATTCCTTCTTCCCTTTTGAGTTACTTGACGACCTTACGGGAAT--ATAAAATGAAACTG-----TGGGTTCTTA---AATAATAATGAATGCATTTTTCTGTTATGATTTCAGTGGTTTTAGTGAGCCATATCTATCAAAATCCCCCCAGCAAAAGAAAAAATAGAACTT--GTTATTTC----------------ATTTAGTTATTTTAAAGAGCCC-----TCCTTTCCGGAATCTCATTAAATTGAAATCCCCCGCGAA-AAACGT-CGACACTCTCATTTTCATGA-----TTATGATCACTAGGT-CTTTATTACGCTCAATTCCT----------------------------------GTATTAG-TATATCTAATACCTTACCCAGCCCATTTGGAAATTTTGGTTCAAACTCTTCGCTACTGGGTAAAAGATGCCCCCTCTTTGCATTTTTTACGATTCTTTCTTCACGACTATCGGCATTGGACTAGTCTTTTTATTCCAAAGAAAGCCAGTTCTTTTTTTTCAAAACGAAAG------------------CAAAGATTATTCTTCTTCCTATATAATTCTCATGTATGTGAATACGAATCCATCTTCGTCTTTCTCCGTAACCAATCTTCTCATTTACAATCAACATCTTCTGGAGCCCTTCTTGAACGAATATATTTCTATGAAAAAATAGAACATCTTGTCGAAGTCTTTGCTAAA-GATTTTCAAGGCAATCTATGGTTGTTCAAGGATCCTTTCATGCATTATGTTAGGTATCAAGGAAAGTCAATTCTCGCTTCAAAGGAGAACTTTCTTTTGATGCGTAAATGGAAATATTACTTTGTACGTTTCTGGCAATGTCATTTTTACCAGTGGTTTCAACCAGGAAGGATCTATATAAACCAATTATCCAAACATTCCCTCGACCTTCTGGGCTATCTTTCAAGTGTGCGGCTAAACCCTTTAACGATACGCAGTCAAATGCTAGAAAATTCATTTCTAATCGATAATGCTGTTAAGAAGTTCGATACCATTGTTCCAATTATTCCTTTGATTGGATCATTGGTTAAAGCGAAATTTTGTAACGTATTAGGGCATCCTGTTAGTAAGGTAGTTTGGGCAGA----------TTAAGACATAAAAATTTACCCTACTTCTTTATCTTTCTTATGAAGGGCCTTTCATGAGTTGAATAGAGATTTTCA-TTTTTTATTCATCATTCGGGTTGATGAACTAAA-CCAGATAGTTATATGAGTGAAAGAAACAGCTTATAAGTTTGCAGTAAAAAGATTGAGTCTCATTTT-CTATGTAC-AAGAG-TTAAGTGAAAGTAACCATAAACATTA-GAAACGGTTTACCCCAAGATTGGTTAATTAGTGATCATGGCTTGAAGCGGGTGC-AAAAGATCAACTGTATGGGG-TTTTTACTATCTATTACCATACATGTATTACCCTAACGGGCGATTAGCAAAAAGAGGTGGATAGTTAGGAACACCAAGGTACACAAAGGATTCGTAATAGAGATTATGTAAGTTATTCAACAGAATTTTTCTGTGCATAA-AAGGAATTCTGATTGGGACTTTAAGTTGGTAGAAATGATGAAGAAGTACTCCCCCTGATTCCGATCCAGAGTATACTCCTATCCACCGATTAAGTAAATAACTATCAAGAACGAAGTAATCCTTTAACTTT------GTTTAAAGTCCCTTTTTCTGAGAAAGGAGAATAGGAACGAAAAAAATA-------AAATAG-AAAGA-ATAGAATTGCACTAG-----AAAGAAAGAGATCTTTTTTTATTCTTTCTTTCCTCTATTTAGAGAGTCTATTTAGAGAGATAGAATTCTTGTCATCATTCGTGAACTAATGCGATGCCTAATTGTTTTTCGTAATCGAAAATGCTAGGTTG--------AAATATCTATGAATA-TTGCTACAAGAAAGA-TTTTATTGAAAG-CTTAAGTTATCACTCAAC-AAAGAAAAATAAAAA-TTATTAAAAGAAAAGATAAGATCAA-TTCCG-AAGCGCTTTA-TTTTCAATATAGCAGACAGAATTCCATTGTCTAATTCGGG--ACTTTACGGTAGATTTAGATTCTATCTATCCTACGAATATAT-------------------CAAGATAAATAATAGCGAACTGGTCCTTAGA-TTTATTTGTGA-CCTTTGAGGAGCC-GTATGAGATGAAAATCTCATGTACGGTATTTAGATTA---------------------------------------------------------------------------------------------------------------------------------------------------------------------------------ATCAA------AATATTTACGATTTTTCTTT-AA-----------AGAAA--ATATGTAAGTCAA--------------------------------------------------------------------------------TGTGAAAT-AAAAAAGGAGCAATAGCTCCCT-CTTGATAGAACAACAAGGGGATTATTGCTCCTTTTTT-ATTTCTTTCAAATACTCGTATACACTAAGGCCAGGTCTTATCCATTTATAGATGGAGCTTCAAGAGCAGCTAGGTCTAGAGGGAAGTTATGAGCATTACGTTCATGCATAACTTCCATACCAAGGTTAGCGCGGTTAATGATATCAGCCCAAGTATTAATTACACGACCTTGACTATCAACTACAGATTGGTTGAAATTAAAACCATTTAGGTTGAAAGCCATAGTGCTGATACCTAAAGCAGTGAACCAGATACCTACTACCGGCCAAGCAGCTAGGAAGAAATGTAAAGAACGAGAGTTGTTGAAACTAGCATATTGGAAGATCAATCGGCCAAAATAACCATGAGCAGCTACAATATTATAAGTTTCTTCTTCTTGA-CCGAATC---TGTAACCTTCATTAGCAATAAATGCAAGAATATTTAC-TTCCATAATCTCATCGTTTTTTTACTTCAAAATAACT-CGGGATTTAATCCCATAGAGATAATAAATCTTTCGCCTGTCAATTCA----ATGAATTACCTCTCGATGATC-TTGAAATCGGATCAATATCATGAATAACAATATCTGAGCTATCAAATCAATTCGTCGTCGAGAATTGAATAGTATAACATAGAAAGATCTTT-TATCCATACCG------AATCCAA------------------------AATTTCTTTATTTATCAATCATTCTTTTCTGTTCTTTCTTTATCTACAACCTATCTTAGGTCCTCCTTGTACAATCATCGGATAAAGTATCGTTTGACCGCCCGTCCGTTTCCATTAGTCACAAACGCCCAACAAACAATAGAAGCGAAGTGGAAAAAGAAATAAGTTACGTTCTAAACTCCG----TTTTTTTAATGATCTAGTTTTCTTGGAAGACAAAGAAGTGTGATAAAGAGGAGTTCCGGGATAAAGGATCTAATATTCCATCAAACTAACTATTTGAGTTTGGGTTTTGTTCGTTCTTCGACGGG--CCCT--------AAAAAAAAATAG--AAAAAAATAGGAAGGAAAAAT-GATTTATTCCCCTGCTACTTGCTAAGCTAAAAAAGGGGTGGGATCTTTGATTGATCTTTATTTTTCTTTTA-CCCCCCCTTCCTT---------------------------------------------------------------------------------------------------------------------------------------------------------------------------------------------------------------------------------------------------------------------------------------------------------------AACGCTCTCAATAATTGTACTATTCTACATATGTCTTTCTCCTACCAATCAGTATTATTTGAAATAATGAAAATTCCCCTATTTGTTTGATGAGAAGTGC----GAAATGCCAAAGGAAA--GAAAAAAGAACCCCCTT-GGGAATG-AAATTCTGCTCCCCGTGCCCCCTTTAACAGAAAAGGG-----------------------------------------------------------TCAGCATTCCCGAGTAACTCCTCAACCTGGAGTTCCACCTGAAGAAGCAGGGGCCGCGGTAGCTGCCGAATCTTCAACTGGTACATGGACAACTGTGTGGACCGATGGACTTACCAGCCTTGATCGTTACAAAGGGCGATGCTACCACATCGAGCCCGTTGCTGGAGAAGAAACTCAATTTATTGCTTATGTAGCTTACCCATTAGACCTTTTTGAAGAAGGTTCTGTTACTAACATGTTTACTTCTATTGTGGGTAATGTATTTGGGTTCAAAGCCCTGCGCGCTCTACGTCTGGAAGATCTGCGAATCCCTGTCGCTTATGTTAAAACTTTCCAAGGCCCACCTCATGGCATCCAAGTTGAGAGAGATAAATTGAACAAGTATGGTCGCCCCCTGTTGGGATGTACTATTAAACCTAAATTGGGGTTATCTGCTAAAAACTATGGTAGAGCGGTTTATGAATGTCTACGTGGTGGACTTGATTTTACCAAAGATGATGAGAA--CGT--GAACTCCCAACCATTTAAATGCTGGGGGAGAGATA-----

Lonicera_involucrata ??????????????????????????????????????????????????????????????????????????????????????????????????????????????????????????????????????????????????????????????????????????????????????????????????????????????????????????????????????????????????????????????????????????????????????????????????????????????????????????????????????????????????????????????????????????????????????????????????????????????????????????????????????????????????????????????????????????????????????????????????????????????????????????????????????????????????????????????????????????????????????????????????????????????????????????????????????????????????????????????????????????????????????????????????????????????????????????????????????????????????????????????????????????????????????????????????????????????????????????????????????????????????????????????????????????????????????????????????????????????????????????????????????????????????????????????????????????????????????????????????????????????????????????????????????????????????????????????????????????????????????????????????????????????????????????????????????????????????????????????????????????????????????????????????????????????????????????????????????????????????????????????????????????????????????????????????????????????????????????????????????????????????????????????????????????????????????????????????????????????????????????????????????????????????????????????????????????????????????????????????????????????????????????????????????????????????????????????????????????????????????????????????????????????????????????????????????????????????????????????????????????????????????????????????????????????????????????????????????????????????????????????????????????????????????????????????????????????????????????????????????????????????????????????????????????????????????????????????????????????????????????????????????????????????????????????????????????????????????????????????????????????????????????????????????????????????????????????????????????????????????????????????????????????????????????????????????????????????????????????????????????????????????????????????????????????????????????????????????????????????????????????????????????????????????????????????????????????????????????????????????????????????????????????????????????????????????????????????????????????????????????????????????????????????????????????????????????????????????????????????????????????????????????????????????????????????????????????????????????????????????????????????????????????????????????????????????????????????????????????????????????????????????????????????????????????????????????????????????????????????????????????????????????????????????????????????????????????????????????????????????????????????????????????????????????????????????????????????????????????????????????????????????????????????????????????????????????????????????????????????????????????????????????????????????????????????????????????????????????????????????????????????????????????????????????????????????????????????????????????????????????????????????????????????????????????????????--------------------------------ACTATGTTACATTACACATGAAGTAAGGATTGAAAAAAGTC--TTT-CTTTCTCTTTCTTTATTATATAG-------ATATGTACAACTTTTACCAGCAATTTCATTTAGATA-TAAGTAAGGGCTCGAAAGAT-CCAATAGACAAAT-------------CTAAAGAAAAATAAAGAAGACCCCGTTGCTTTGATTTTGTTCCTTTTATTCCCATAGCCTGGCCCGGTCAATACCTAGCCGGGCC-----TTTTTTGTTCCAACGAATCCTAGCTAAAAGGATTTAGCTG-------ATTTGAATTTGAAAACA---------------AAAATGCTTGCTATTAAAGCAGC-------------------AATAAAAAGACGCGGGGCTATTTCCATTCTT-TTTAT---------------------------------------ATA----------------------------------AATGGATATAAATTATATA-----AAAGTCGCATTTCTTATTTTATAATTCCTTCTCCCTTTTTGAGTTACTTGACGACCTTACGGGAAT-AAAAAAATGAAACTA-----TGGGTTGTTA---AATAATAATGAATGCATTTTTCTGTTATGATTTCGGTGGTTTTAGCGAGCCATATCTATTAAAACCCCTCCAGCAAAAGAAAAGGTAGAGCTT--GTT---------------------ATTTAGTT---------AGCCC-CCCTTTCTTTCCGGAATCTCATTAAATTGAAACCCCCCGTGAA-AAACAT-CGACACTCGCATTTTCATGATTCTTTTATGATC-CTA--T-CTTTATTACGCCCAATTCC-----------------------------------GTATTAGATATA-CTAATACCTTACTCAGCCCATTTGGAAATTCTGGTTCAAACTCTTCGCTACTGGGTAAAAGATGCCCCCTCTTTGCATTTATTACGATTCTTTCTCCACGAGTATCCTAATTTGACTAGTCTTATTATTCCAAAGAAAGCTGGTTCTTCTTTTTCAAAACGAAAT------------------CAAAGATTATTTTTCTTCCTATATAATTCTCATGTATGTGAATACGAATTCATCTTCGTCTTTCTCCGTAACCAATCTTCTCATTTACGATCAACATCTTCTGGAGCCTTTCTTGAACGAATATATTTCTATGAAAAAATAGAACATCTTGTAGAAGTCTTTGCTAAG-GATTTTCAAGCCAATTTATGGTTGTTCAAGGATCCTTTCATGCATTATGTTAGGTATCAAGGAAAGTCAATTCTCGCTTCAAAGGGGACCTTTCTTTTGATGAATAAATGGAAATATTACTTTGTACGTTTCTGGCAATGGCATTTTT--------------------------------------------------------------------------------------------------------------------------------------------------------------------------------------------------------------------------------------------------------------------------???????????????????????????????????????????????????????????????????????????????????????????????????????????????????????????????????????????????????????????????????????????????????????????????????????????????????????????????????????????????????????????????????????????????????????????????????????????????????????????????????????????????????????????????????????????????????????????????????????????????????????????????????????????????????????????????????????????????????????????????????????????????????????????????????????????????????????????????????????????????????????????????????????????????????????????????????????????????????????????????????????????????????????????????????????????????????????????????????????????????????????????????????????????????????????????????????????????????????????????????????????????????????????????????????????????????????????????????????????????????????????????????????????????????????????????????????????????????????????????????????????????????????????????????????????????????????????????????????????????????????????????????????????????????????????????????????????????????????????????????????????????????????????????????????????????????????????????????????????????????????????????????????????????????????????????????????????????????????????????????????????????????????????????????????????????????????????????????????????????????????????????????????????????????????????????????????????????????????????????????????????????????????????????????????????????????????????????????????????????????????????????????????????????????????????????????????????????????????????????????????????????????????????????????????????????????????????????????????????????????????????????????????????????????????????????????????????????????????????????????????????????????????????????????????????????????????????????????????????????????????????????????????????????????????????????????????????????????????????????????????????????????????????????????????????????????????????????????????????????????????????????????????????????????????????????????????????????????????????????????????????????????????????????????????????????????????????????????????????????????????????????????????????????????????????????????????????????????????????????????????????????????????????????????????????????????????????????????????????????????????????????????????????????????????????????????????????????????????????????????????????????????????????????????????????????????????????????????????????????????????????????????????????????????????????????????????????????????????????????????????????????????????????????????????????????????????????????????????????????????????????????????????????????????????????????????????????????????????????????????????????????????????????????????????????????????????????????????????????????????????????????????????????????????????????????????????????????????????????????????????????????????????????????????????????????????????????????????????????????????????????????????????????????????????????????????????????????????????????????????????????????????????????????????????????????????????????????????????????????????????????????????????????????????????????????????????????????????????????????????????????????????????????????????????????????????????????????????????????????????????????????????????????????????????????????????????????????????????????????????????????????????????????????????????????????????????????????????????????????????????????????????????????????????????????????????????????????????????????????????????????????????????????????????????????????????????????????????????????????????????????????????????????????????????????????????????????????????????????????????????????????????????????????????????????????????????????????

Morina_longifolia CCTGAGCCAAATCCAGTTTTCCGAAAACAAACAAGGGTTCAGAAAGCTAAAATC-AAAAAGGATAGGTGCAGAGACTCAATGGAAGCTGTTCTAACAAATGGAGTTGAC-----TGTGTTGGTAGAAAGAATCCTTCCATAGAAACTTCAGAAAGGATAAACCTATAAACATAGATATATGCATTGAAATACTAT-ATATTCTACCAAATGATTAATGACAACCCGAATCTTTATA--TATATATTATATATCAAAATGGGAGA-----TGTGAAGTGATTCCATATTGAAGAAAGAATCGAATATTCATTGATCAAATCATTCACTCCATAGTCTGATAGATCTTTTTAAAGAACTGATTAATCGGACGAGAATAAAGATAGAGTCCCATTCTACATGTCAATACCGGCAACAATGAAATTTATAGTAAGAGGAAAATCCG-CGACTTTAGGAATCGTGAGGGTTCAAGTCCCTCTATCCCCAAAAAACCCATATGAGCTCTCTAATTCTTTATTCTCTCCTT-TTATGC-TTTTTTGTTAGCGGTTCCAAATTCGTTATCTTTATCTTTCTCATTCACCCTACTCTTTTACAAAGAGATCTGAGCGGAAATGTTTTTCTCTTATC----ACAAGTCTTGGGATCTAAG----ATAA-TCCGTGTACAAATGAACATCTTTGAGTAAGTAATCCCCATTTGAATGATTCATGATCAA------TATCATTATTCATACTGAAACTTACAAAGTCTTCCTTATTGAAGATCCAAGAAATTCCAGGACCTGGATAAGACTTTGTAATA-CCTTTCAATTGACA-TAGACCCGAGTTATCTAGC--AAAATGAGGATGCA?????????????????????????????????????????????????????????????????????????????????????????????????????????????????????????????????????????????????????????????????????????????????????????????????????????????????????????????????????????????????????????????????????????????????????????????????????????????????????????????????????????????????????????????????????????????????????????????????????????????????????????????????????????????????????????????????????????????????????????????????????????????????????????????????????????????????????????????????????????????????????????????????????????????????????????????????????????????????????????????????????????????????????????????????????????????????????????????????????????????????????????????????????????????????????????????????????????????????????????????????????????????????????????????????????????????????????????????????????????????????????????????????????????????????????????????????????????????????????????????????????????????????????????????????????????????????????????????????????????????????????????????????????????????????????????????????????????????????????????????????????????????????????????????????????????????????????????????????????????????????????????????????????????????????????????????????????????????????????????????????????????????????????????????????????????????????????????????????????????????????????????????????????????????????????????????????????????????????????????????????????????????????????????????????????????????????????????????????????????????????????????????????????????????????????????????????????????????????????????????????????????????????????????????????????????????????????????????????????????????????????????????????????????????????????????????????????????????????????????????????????????????????????????????????????????????????????????????????????????????????????????????????????????????????????????????????????????????????????????????????????????????????????????????????????????????????????????????????????????????????????????????????????????????????????????????????????????????????????????????????????????????????????????????????????????????????????????????????????????????????????????????????????????????????????????????????????????????????????????????????????????????????????????????????????????????????????????????????????????????????????????????????????????????????????????????????????????????????????????????????????????????????????????????????????????????????????????????????????????????????????????????????????????????????????????????????????????????????????????????????????????????????????????????????????????????????????????????????????????????????????????????????????????????????????????????????????????????????????????????????????????????????????????????????????????????????????????????????????????????????????????????????????????????????????????????????????????????????????????????????????????????????????????????????????????????????????????????????????????????????????????????????????????????????????????????????????????????????????????????????????????????????????????????????????????????????????????????????????????????????????????????????????????????????????????????GTATTAG-TATACCTAATACCTTACCCAGCCCATTTGGAAATTTTGGTTCAAACTCTTCGCTACTGGGTAAAAGATGCCCCTTCTTTGCATTTATTACGATTCTTTCTCCACGACTATCGGCATTGGACTAGGCTTTATATTCCAAAGAAAGCCAGTTCTTTTTTTTCAAAACGAAAG------------------CAAAGATTATTCTTCTTACTATATAATTCTCATGTATGTGAATACGAATCCATCTTTGTCTTTCTCCGTAACCAATCTTCTCATTTACAATCAACATCTTCGGGAGCCCTTCTTGAACGAATATATTTCTATGAAAAAATAGAACACCTTGTAGAAGTCTTTGCTAAA-GATTTTCAAGGAAATCTATGGTTGTGCAAGGATCCTTTCATTCATTATGTTAGGTATCAAGGAAAGTCAATTCTCGCTTCAAAGGGGAACTTTCTTTTGATGCATAAATGTAAATATTACTTTGTACATTTCTGGCAATGTCATTTTTACCAGTGGTTTCAACCAGGAAGGATCCATATAAACCAATTATCCAAACATTCCCTCGACCTTCTGGGCTATCTTTCAAGTGTGCGGCTAAACCCTTTAACGGTACGCAGTCAAATGCTAGCAAATTCATTTCTAATCGATAATGCTGTTAAGAAGTTCGATACCATTGTTCCAATTATTCCTCTGATTGGATCATTGGTTAAAGCGAAATTTTGTAACGTATTAGGGCATCCTCTTAGTAAGGTAGTTTGGGCAGATTTATCAGAT?????????????????????????????????????????????????????????????????????????????????????????????????????????????????????????????????????????????????????????????????????????????????????????????????????????????????????????????????????????????????????????????????????????????????????????????????????????????????????????????????????????????????????????????????????????????????????????????????????????????????????????????????????????????????????????????????????????????????????????????????????????????????????????????????????????????????????????????????????????????????????????????????????????????????????????????????????????????????????????????????????????????????????????????????????????????????????????????????????????????????????????????????????????????????????????????????????????????????????????????????????????????????????????????????????????????????????????????????????????????????????????????????????????????????????????????????????????????????????????????????????????????????????????????????????????????????????????????????????????????????????????????????????????????????????????????????????????????????????????????????????????????????????????????????????????????????????????????????????????????????????????????????????????????????????????????????????????????????????????????????????????????????????????????????????????????????????????????????????????????????????????????????????????????????????????????????????????????????????????????????????????????????????????????????????????????????????????????????????????????????????????????????????????????????????????????????????????????????????????????????????????????????????????????????????????????????????????????????????????????????????????????????????????????????????????????????????????????????????????????????????????????????????????????????????????????????????????????????????????????????????????????????????????????????????????????????????????????????????????????????????????????????????????????????????????????????????????????????????????????????????????????????????????????????????????????????????????????????????????????????????????????????????????????????????????????????????????????????????????????????????????????????????????????????????????????????????????????????????????????????????????????????????????????????????????????????????????????????????????????????????????????????????????????????????????????????????????????????????????????????????????????????????????????????????????????????????????????????????????????????????????????????????????????????????????????????????????????????????????????????????????????????????????????????????????????????????????????????????????????????????????????????????????????????????????????????????????????????????????????????????????????????????????????????????????????????????????????????????????????????????????????????????????????????????????????????????????????????????????????????????????????????????????????????????????????????????????????????????????????????????????????????????????????????????????????????????????????????????????????????????????????????????????????????????????????????????????????????????????????????????????AAGATTACAAATTGACTTATTATACTCCTGACTATGAAACCAAAGATACTGATATCTTGGCAGCATT-CCGAGTAACTCCTCAACCTGGAGTTCCACCTGAAGAAGCAGGGGCCGCGGTAGCTGCCGAATCTTCAACTGGTACATGGACAACTGTGTGGACCGATGGACTTACCAGCCTTGATCGTTACAAAGGGCGATGCTACCACATCGAGCCCGTTCCTGGAGAAGAAAATCAATTTATTGCTTATGTAGCTTACCCATTAGACCTTTTTGAAGAAGGTTCTGTTACTAACATGTTTACTTCTATTGTGGGTAATGTATTTGGGTTCAAAGCCCTGCGCGCTCTACGTCTGGAAGATCTGCGAATCCCTGTCGCTTATGTTAAAACTTTCCAAGGCCCGCCTCATGGCATCCAAGTTGAGAGAGATAAATTGAACAAGTATGGTCGCCCCTTGTTGGGATGTACTATTAAACCTAAATTGGGGTTATCTGCTAAAAACTATGGTAGAGCGGTTTATGAATGTCTACGTGGTGGACTTGATTTTACCAAAGATGATGAGAC--CGT--GAACTCCCAACCATTTATGCGTT---GGAGAGATCGTTTC

Symphoricarpos_sinensis_BOP012300 CCTGAGCCAAATCCAGTTTTCCGAAAACAAACAAGGGTTCAGAAAGCAAAAATC-AAAAAGGATAGGTGCAGAGACTCAATGGAAGCTGTTCTAACAAACGGAGTTGACTGTCTTGTGTTGGTAGAAAGAATCCTTCCATAGAAACTTCAGAAAGGATAAACCTATAAACATAGATATACGTATTGAAATGCTATGATACTATAGCAAATGATTAATGACGATCCAAATCCGTATCTATATTTTATATATATCAAAATGGAAGAATTGTTGTGAAGTGATTCCATATTGAAGAAAGAATCGAATATTCATTGATCAAATCATTCACTCCATAGTCTGATCGATC-TTTTGAAGAACTGATTAATCGGACGAGAATAAAGATAGAGTCCCATTCTACATGTCAAGACCGGCAACAATGAAATTTATAGTAAGAGGAAAATCCGTCGACTTTAGAAATCGTGAGGGTTCAAGTCCCTCTATCCCCAAAAAACCCATATTGACTCCCTAATGATTTATCCTCTCCTTTTTATCCTTTTTTTGTTAGCGGTTCAAAATTCG------TTCTATTTATCATCCACCCTACTCTTTTACAAAGAGATCTGAGCGGAAATGTTTTTCTCTTATC----ACAAGTCTTGTGATCTAAG----ATAA-TACGTGTACAAATGAACATCTTTGAGTAAGGAATCCCCATTTGAATGATTCACGGCCAA------TATCATTATTCATACTGAAACTTACAAAGTCTTCCTT-TTGAAGATCCAAGAAATTCCAGGACCTGGATAAGACTTTGTAATACCCTTTCAATTGACA-TAGACCCGAGTTAGCTAGT--AAAATGAGGATGCA????????????????????????????????????????????????????????????????????????????????????????????????????????????????????????????????????????????????????????????????????????????????????????????????????????????????????????????????????????????????????????????????????????????????????????????????????????????????????????????????????????????????????????????????????????????????????????????????????????????????????????????????????????????????????????????????????????????????????????????????????????????????????????????????????????????????????????????????????????????????????????????????????????????????????????????????????????????????????????????????????????????????????????????????????????????????????????????????????????????????????????????????????????????????????????????????????????????????????????????????????????????????????????????????????????????????????????????????????????????????????????????????????????????????????????????????????????????????????????????????????????????????????????????????????????????????????????????????????????????????????????????????????????????????????????????????????????????????????????????????????????????????????????????????????????????????????????????????????????????????????GAGATTGAAAA-------------------AAA-----AAATCAATGCAATATTTTTTAGGAAAGATAAAAATGAATGAATAAAAATTCGTTCAAATGGG-----------------------ATTGAAAGGTTCATTTTTTTCGTTTAGGGTGTTCATTTTATTTCTTCCCTTAGGACTTTGGTGTAGTTTATGCTCTC----CTGGAATCG-AACTGTTGT-AACTGGACGCTTCTATCCT-CTAGCTA-----GGGGTAGAACGAAAAAAAAAAAAAATTTTCA------TTTTTTAATGAATTCTTTCTCATTTATCCGATTTA--AAATTTGAAGCAAAGGGATCCATTTTTTCAATCAACACAA---------AAGAAATCTGAAAGTT--------------ATACAAAAGGTTATCAAAATGGAATCTATTCGTTTCACCAATTCCTTA-----------------------ATTTTAACTAAAGATCTTACA----TAGGCCC-------TTCTATAGATAGATATAGAGAAAAAATTTTAGATATATAGAGAAAA------------------------------ATTTTTCT-------TATTAGAATTGTGACAAATAGGTTGATGGGGAAAATAAGATCCCGCTCTCAAATCTCAAAATGAGAAAATCTACTAAAAAGAAAGGGAAACCCTTGTATTTTGTCTTTCTTC---------------TTTTTTCAAAAGTC--------TTCTTTTC---------AAATTGAGTAAACAGAAGCATTTTT-TTCTACTTCCATTCCCTATTGTTTTCGGCT-------AATCAATAGGAAATGGAAGTTATTCATTTTATTTTTAGATTAACAATGAAATCAGACATTTTTTCGATTCAAATTAATTCGGATTATTCCAATGTTTTATGA--------CTTATTTGTTTGTCGCACAAAATAACTTTTTGAATTCCCTGTAG-------AAAGAGAT---------TCCCCAA--------GCCATCTCTCCTAATTGAAAAAGGTAATAATTACTATGTTACATTACACATGAAGTAAGGATTGAAAAAAGTA--TTT-CTTTCTCTTTCTTTATTATATAG-------ATATGTACAACTTTTACCAGCAATTTCATTTAGATATTAAGTAAGGGCTCGAAAGAT-CCAATAGACAAAT-------------CTAAAGAAACATAAAGAAGACCCCGTTGCTTTGATTTTATTCCTTTTATTCCCACAGCCCGGCCCGGTCAATACCTAGCCGGGCC-----TTTTTTGTTCCAACGAATCCTAGCTAAAAGAATTTCGCTG-------ATTTGAATTTGAAAACA---------------AAAATGCTTGCTATTAAAGCAGC-------------------AATAAAAAGACGCGGGGCTATTTCCATTCTT-TTTAT--------------------------------ATATTATATA----------------------------------AATTGATATAAATTATATA-----AAAGTCGCATTTATTATTTTATAATTCCTTCTCCCTTTTTGAGTTACTTGACGACCTTACGGGAAT-AAAAAAATGAAACTA-----TGGATTCTTA---ACTAATAATGAATCCATTTTTCTGTTAGGATTTCAGTGGTTTTAGCGAGCCATATCTATTAAAACCCCTCCAGCAAAAGAAAAGGTAGAGCTT--GTT---------------------ATTTAGTTATTTAAAAGAGCCC--CCTTTCTTTCCGTAATCTCATTAAATTGAAATCCCCCGCGAA-AAACGT-CGACACTCGCATTTTCATGATTCTTTTAGGATC-CTA--T-CTTTATTACGCCCAATTCCTCTGTT-CGACAAAAAGTTCATTTGTATATAATATGTATTAGATATA-CTAATACCTTACTCAGCCCATTTGGAAATTCTGGTTCAAACTCTTCGCTACTGGGTAAAAGATGCCCCCTCTTTGCATTTATTACGATTCTTTCTCCACGAGTATCCTAATTTGACTAGTCTTATTATTCCAAAGAAAGCCGGTTCTTCTTTTTCAAAAGGAAAT------------------CAAAGATTCTTTTTCTTCCTATATAATTCTCATGTATGTGAATACGAATTCATCTTCGTCTTTCTCCGTAACCAATCTTCTCATTTACGATCAACATCTTCTGGAGCCCTTCTTGAACGAATCTATTTTTATGAAAAAATAGAACATCTTGTAGAAGTCTTTGCTAAAGGATTTTCAAGCCAATCTATGGTTGTTCAAGGATCCTTTCATGCATTATGTTAGGTATCAAGGAAAGTCAATTCTCGCTTCAAAGGGGACCTTTCTTTTGATGAATAAATGGAAATATTACTTTGTACGTTTCTGGCAATGTCATTTTTACCAGTGGTTTCAACCAGGAAGGATCTCTATAAACCAATTATCCAAACATTCGCTCGACCTTCTGGGTTATCTTTCAAGTGTGCGGCTAAACCCCTTAACGGTGCGCAGTCAAATGCTAGAAAATGCATTTCTAATCGATAATGCTGGTAAGAAGTTCGATACCATTGTTCCAATTAGTCCTCTGATTAGATCATTGGCTAAAGCGAAATTTTGTAACGTATTAGGGCATCCTGTTAGTAAGGTGCTTTGGGCCAATTTATCAGATTTGAGACAT-AAAATTAACCCTACTTCTTT-TCTTTC-TAGGAAGGGCCTTTGATGAGTTGAATATATATTTTTC-TTTTTGATTCATCATTCGGGTTGATGAACTAAA-CCAGATAGTTATATGAGTGAAAGAAACAGCTTCTAAATTTGCAGTAAAAAGATTGAATCTCATTTT-CTATGTAC-AAGAG-TGAAGAGAAAGTAAACATAAACATTA-GAAACTGTTTACCCCAAGATTGGTTAATTAGTGATCATGGCTTGAAGCGGGTGC-AAAAGATCAACTATATGGGG-TTTTTCCTATCTATTACCATACATGTATTATCTTAACGGCGGATTCGCA-AAAGAGATGGATAGCTAGGAACACCAAGGTACACAAAGGATTCGTAATAGAGATTATGTAAGTTATTCAACAGGATTTTTCTGTGCATAATAAGGAATTCTAATTGGAACTTTAAGTTGGTAGAAATGATGAAGAAGTACTCCCCCCGATTCCGATCCAGAGTATCCTCCTATCCACCGATTAAGTAAATAACTATCAAGAACGAAGTAATCCTTT-ACTTTGTTTAAGTTTAAAGTCCCTTTTTCTGAGAAAGGAGAATAGGAACGAAAAAAAGA-------AACTAG-AAAGA-ATATAATTGCACTAG-----AAAGAAAGAGATCTTTTTTTATTCTTTCC-----------------TCTATTTAGAGAAATAGAATTCTTGTCATGATTCATGAACTAATGTGATACCTAATTGTTTTTCGTAATCGAAAATGCTAGGTTG--------AAATATCTATTGATA-TTGCTACAAGAAAGA-TTTTATTGAAAG-CTTAAGTTATTCCTCAAC-AAAGAAAAATGAAAA-TTCTT-----AAAAGATAAGATCAA-TTCCG-AAGCACTTTA-TTTTAAATATAGCAGACAGAATTCCATTGTCTAATTCGGGACACCTTACGGTATATTTTGACTCTACCTATCCTACGAATATAT-------------------CAAGATAAATAATAGCGAACCGGTCCTTAGA-TTTATTTGTGA-CCTTTGAGGAGCC-GTATGAGATGAAAATCTCATGTACGGTTCCTAGTTCA-------------------GCCATTCATGATTTTTCTTTCTTTTTTCTCAAGTACAATTATAGATAAGCCAGCAAAAAAATTCCAACAGTTTCTCTTTT------ATTTTAGAGGATATAAAT-TTTTTCATACAGAAAAAAAAATGAAAGCCCAAAACACAATCATGACCCAACCCCTAAAAATAAGAAGATTTACGATTTTTCTTG-AA-----------AGAAA--ATAGGTAAGTCAA----------------------------------------------------------------------------ACACTGTTAAAT-AAAAAAGGAGCAATGACGCCCT-CTTGATAAAACAAGAGGGGGATTATTGCTCCTTTTTTCAT----TTCAAAAACTCGTATACACTAAGACTGGGTCTTATCCATTTGTAGATGGAGCTTCAAGAGCGGCTAGGTCTAGAGGGAAATTATGAGCATTACGTTCATGCATAACTTCCATACCGAGGTTAGCACGATTAATGATATCAGCCCAAGTATTAATTACACGGCCTTGACTATCAACTACAGATTGGTTGAAATTAAAACCATTTAGGTTGAAAGCCATAGTGCTGATACCTAAAGCAGTGAACCAGATACCTACTACAGGCCAAGCGGCTAGGAAGAAATGTAAAGAACGAGAGTTGTTGAAACTAGCATATTGGAAGATCAATCGGCCAAAATAACCATGAGCGGCTACAATATTATAAGTTTCTTCTTCTTGA-CCGAATC---TGTAACCTTCATTAGCAATAAATGCAAGAATATTTAC-TTCCATAATCTCATCGTTTTTTTACTTCAAAATAACT-CGGGATTTAATCCCATAGAGATAATAAATCTTTCTCCTGTCAATTCAATGAATGAATTCCCTCTCGATGATC-TTGAAATCAGATCAATATCATGAATAACAATATCTGAGCTATCAAATCAATTCGTCGTCAAGAATTGAATAGTATAACATAGGAAGATCTTT-TATCCATACCG------AATCCAA------------------------AATTTCTTTATTT----CTCATTCTTTTCTG----TTCTTTATCTATAACCTACCTTCCGTCCTCCTTGTACAATCATAGGATAAAGTATCGTCTGACCACCCGTCCGTTTCCATTAGTCACAAACCCCCAACAAACAATCGAAGCGAAGTGGAAAAAGAAAGGAGTTACGTTCTAAACTCCGTTTTTTTTTTTAATGATCTAGTTTTCTTGGAAGACAAAGAAGTGTGATAAAGAGGAGTCCCGGGATAAAGGATGTAATATTCCATCAAACTAACTATTTTAGTTTGGGGTTTGCTCGTTCTTCGACGAG--CCCT--------CTAAAAAAATAT----CAAAATAGGAAGGAAAAATGGATTTATTCCCCTGCTACTTGCTAAGCTAATTAAGGGGTGGGATCTTTGATTGATCTTTATTTTTCTTTTA--CCCTCCTTCCTTAGGTTATTCGTACTGGGATACCTATACCAAAAGCTCAGCGTACAATTTGAATGAAATAGATTTTTCAACTTCACATTAGTAATTGCGATTACACAAACAAAACCGAAATCAGAAGGGGGATTTTTGAATCTGGAAAAGTATTCTATCAGGGAAATTCTGTTAATGTGAAATTCATTTTGTATTGTACAAGAAATGAATTCCATTTTGGTCTGTGCGCCCGAGAAACATATAGTCCTCTATTCCATTCCATGAATTGGGAACAATCGAAAAAGCAGACTCAGTATCTCATGGAATCCGGACTAGAATGCTCTCAATAATTGTA-----CTAAATATGTCTTTCTCCTACCAATCTGTAGGAGTTGAAATAATGAAAATCCCCCTATTTATTTGATGAGAAATGCGAAAGAAATGCCAAAGGAAA--GAAAAAAGAACCCCCTT-GGGAATA-AAATTCTGCCCACTGTGCCCC-TTTCACAGAAAAGGG------------------------------------------------------------------T-CCGAGTAACTCCTCAACCTGGAGTTCCACCTGAAGAAGCAGGGGCCGCGGTAGCTGCTGAATCTTCAACTGGTACATGGACAACCGTGTGGACCGATGGACTTACCAGCCTTGATCGTTACAAAGGGCGATGCTACCACATCGAGCCCGTTGCTGGAGAAGAAACTCAATTTATTGCTTATGTAGCTTACCCATTAGACCTTTTTGAAGAAGGTTCTGTTACTAACATGTTTACTTCTATTGTGGGTAATGTATTTGGGTTCAAAGCCCTGCGCGCTCTACGTCTGGAAGATCTGCGAATCCCTACCTCTTATGTTAAAACTTTCCAAGGCCCGCCTCATGGTATCCAAGTTGAGAGAGATAAATTGAACAAGTACGGTCGCCCCCTGTTGGGATGTACTATTAAACCTAAATTGGGGTTATCTGCTAAAAACTACGGTAGGGCGGTTTATGAATGTCTACGCGGTGGACTTGATTTTACCAAAGATGATGAGAA--CGT--GAACTCCCAACCATTTATGCGTT---GGAGAGATCGTTTC

Triosteum_perfoliatum CCTGAGCCAAATCCAGTTTTCCGAAAACAAACAAGGGTTCAGAAAGCAAAAATC-AAAAAGGATAGGTGCAGAGACTCAATGGAAGCTGTTCTAACAAACGGAGTTGACTGTCTTGTGTTGGTAGAAAGAATCCTTCCATAGAAACTTCAGAAAGGATAAACCTATAAACATAGATATACGTATTGAAATGCTATGATACTATATCAAATGATTAATGACGATCCAAATCTTTATTTGTATTTTATATATATCAAA---------TTGTTGTGAAGTGATTCCATATTGAAGAAAGAATCGAATATTCATTGATCAAATCATTCACTCCATAGTCTGATCGATC-TTTTGAAGAACTGATTAATCGGACGAGAATAAAGATAGAGTCCCATTCTACATGTCAAGACCGGCAACAATGAAATTTATAGTAAGAGGAAAATCCGTCGACTTTAGAAATCGTGAGGGTTCAAGTCCCTCTATCCCCAAAAAACCCATATTGACTCCCTAATTATTTATCCTCTCCTTTTTATCC-TTTTTTGTTAGCGGTTCAAAATTCG------TTATATTTCTCATCCATCCTACTCTTTTACAAAGAGATTTGAGCGGAAATGTTTTTCGCTTATC----ACAAGTCTTGTGATCTAAG----ATAA-TACGTGTACAAATGAACATCTTTGAGTAAGGAATCCCCATTTGAATGATTCACGGCCAA------TATCATTATTCATACTGAAACTTACAAAGTCTTACTT-TTGAAGATCCAAGAAATTCCAGGACCTGGATAAGACTTTGTAATACCCTTTCAATTGACA-TAGACCCGAGTTATCTAGT--AAAATGAGGATGCA????????????????????????????????????????????????????????????????????????????????????????????????????????????????????????????????????????????????????????????????????????????????????????????????????????????????????????????????????????????????????????????????????????????????????????????????????????????????????????????????????????????????????????????????????????????????????????????????????????????????????????????????????????????????????????????????????????????????????????????????????????????????????????????????????????????????????????????????????????????????????????????????????????????????????????????????????????????????????????????????????????????????????????????????????????????????????????????????????????????????????????????????????????????????????????????????????????????????????????????????????????????????????????????????????????????????????????????????????????????????????????????????????????????????????????????????????????????????????????????????????????????????????????????????????????????????????????????????????????????????????????????????????????????????????????????????????????????????????????????????????????????????????????????????????????????????????????????????????????????????????????????????????????????????????????????????????????????????????????????????????????????????????????????????????????????????????????????????????????????????????????????????????????????????????????????????????????????????????????????????????????????????????????????????????????????????????????????????????????????????????????????????????????????????????????????????????????????????????????????????????????????????????????????????????????????????????????????????????????????????????????????????????????????????????????????????????????????????????????????????????????????????????????????????????????????????????????????????????????????????????????????????????????????????????????????????????????????????????????????????????????????????????????????????????????????????????????????????????????????????????????????????????????????????????????????????????????????????????????????????????????????????????????????????????????????????????????????????????????????????????????????????????????????????????????????????????????????????????????????----------------------------------------------------------------------GTC--TTT-CTTTCTCTTTCTTTATTATATAA-------ATATGTACAACTTTTACCAGCAATTTCATTTAGATA-TAAGTAAGGGCTCGAAAGAT-CCAATAGACAAATCTAAAGAAAAATAAAAAAGAAAAATAAAGAAGACCCCGGTGCTTTGATTTTGTTCCTTTTATTCCCACAGCCTGGCCCGGTCAATACCTAGCCGGGCC----TTTTTTTGTTCCAACGAATCCTAGCTAAAAGAATTTAGCTG-------ATTTGAATTTGAAAACA---------------AAAATGCTTGCTATTAAAGCAGC-------------------AATAAAAAGACGCGGGGCTATTTCCATTCTT-TTTAT---------------------------------------ATA----------------------------------AATTGATATAAATTATATA-----AAAGTCGCATTTCTTATTTTATAATTCTTTCTCCCTTTTTGAGTTACTTGACGACCTTACGGGAATAAAAAAAATGAAACTA-----TGGATTCTTA---AATAATAATGAATGCATTTTTCTGTTATGATTTCAGTGGTTTTAGCGAGCCATATCTATTAAAACCCCTCCAGCAAAAGAAAAGGTAGAGCTT--GTT---------------------ATTTAGTTATTTAAAAGAGCCC--CCTTTCTTTCCGGAATCTCATTAAATTGAAATCCCTCGCGAA-AAACGT-CGACACTCGCATTTTCATGATTCTTTTATGATC-CTA--T-CTTTATTACGCCC-----------------------------------------GTATTAGATATA---------------AGCCCATCGGGAAATT-TGGTTCAAATTCTTCGCTACTGGGTAAAAGAGGCCCCCTCTCCGCATTTATTACGATTCTTTCTCCACGAGTATCCGAATTTGACTGGTCTTATTATTCCAAAGAAAGCCGGTACTTCTTTTTCAAAACGAAAT------------------CAAAGATTCTTGTTCTTCCCATATAATTCTCATGTATGTGAATACGAATTAATGTTTGTCTTTCTCCGTAACCAATCTTCTCATTTACGATCAACATATTMAGGAGCCCTTCTTGAACGAATATATTTCTATGAAAAAATAGAACATCTGGTAGAAGTCTTTGCTAAG-GATTTTCAAGCCAATCTATGGTTGTTCAAGGATCCTTTCATGCATTATGTTAGGTATCAAGGAAAGTCAATTCTCGCTTCAAAGGGGACCTTTCTTTTGATGAATAAATGGAAATATTACTTTGTACGTTTCT-----------------------------------------------------------------------------------------------------------------------------------------------------------------------------------------------------------------------------------------------------------------------------------------?????????????????????????????????????????????????????????????????????????????????????????????????????????????????????????????????????????????????????????????????????????????????????????????????????????????????????????????????????????????????????????????????????????????????????????????????????????????????????????????????????????????????????????????????????????????????????????????????????????????????????????????????????????????????????????????????????????????????????????????????????????????????????????????????????????????????????????????????????????????????????????????????????????????????????????????????????????????????????????????????????????????????????????????????????????????????????????????????????????????????????????????????????????????????????????????????????????????????????????????????????????????????????????????????????????????????????????????????????????????????????????????????????????????????????????????????????????????????????????????????????????????????????????????????????????????????????????????????????????????????????????????????????????????????????????????????????????????????????????????????????????????????????????????????????????????????????????????????????????????????????????????????????????????????????????????????????????????????????????????????????????????????????????????????????????????????????????????????????????????????????????????????????????????????????????????????????????????????????????????????????????????????????????????????????????????????????????????????????????????????????????????????????????????????????????????????????????????????????????????????????????????????????????????????????????????????????????????????????????????????????????????????????????????????????????????????????????????????????????????????????????????????????????????????????????????????????????????????????????????????????????????????????????????????????????????????????????????????????????????????????????????????????????????????????????????????????????????????????????????????????????????????????????????????????????????????????????????????????????????????????????????????????????????????????????????????????????????????????????????????????????????????????????????????????????????????????????????????????????????????????????????????????????????????????????????????????????????????????????????????????????????????????????????????????????????????????????????????????????????????????????????????????????????????????????????????????????????????????????????????????????????????????????????????????????????????????????????????????????????????????????????????????????????????????????????????????????????????????????????????????????????????????????????????????????????????????????????????????????????????????????????????????????????????????????????????????????????????????????????????????????????????????????????????????????????????????????????????????????????????????????????????????????????????????????????????????????????????????????????????????????????????????????????????????????????????????????????????????????????????????????????????????????????????????????????????????????????????????????????????????????AAGATTACAAATTGACTTATTATACTCCTGACTATGAAACCAAAGATACTGATATCTTGGCAGCATT-CCGAGTAACTCCTCAACCTGGAGTTCCACCTGAAGAAGCAGGGGCCGCGGTAGCTGCTGAATCTTCAACTGGTACATGGACAACTGTGTGGACCGATGGACTTACCAGCCTTGATCGTTACAAAGGGCGATGCTACCACATCGAGCCCGTTGCTGGAGAAGAAAGTCAATTTATTGCTTATGTAGCTTACCCATTAGACCTTTTTGAAGAAGGTTCTGTTACTAACATGTTTACTTCTATTGTGGGTAATGTGTTTGGGTTCAAAGCCCTGCGCGCTCTACGTCTGGAAGATCTGCGAATCCCTGTCTCTTATGTTAAAACTTTCCAAGGCCCGCCTCATGGTATCCAAGTTGAGAGAGATAAATTGAACAAGTACGGTCGCCCTCTGTTGGGATGTACTATTAAACCTAAATTGGGGTTATCTGCTAAAAACTACGGTAGGGCGGTTTATGAATGTCTACGTGGTGGACTTGATTTTACCAAAGATGATGAGAA--CGT--GAACTCCCAACCATTTATGCGTT---GGAGAGACCGTTTC

Vesalea_coriacea_var_coriacea_BOP022785 CCTGAGCCAAATCCAGTTTTACGAAAACAAACAAGGGTTCAGAAAGCTAAAATC-AAAAAGGATAGGTGCAGAGACTCAATGGAAGCTGTTCTAACAAATGGAGTTGAC-----TGTGTTGGTAGAAAGAATCCTTCCATAGAAACTTCAGAAAGGATAAACGTATAAACATAGATATACGCATTGAAATACTAT-ATACTCTACCAAATGATTAATGACGACCCGAATCTGTATT---------TATATATCAAAATGGGAGAATGGTTGTGAAGTGATTCCATATTGAAAAAAGAATCGAATATTCATTGATCAAATCATTCACTCCATAGTCTGATAGATC-TTTTGAAGAACTGATTAATTGGACGAGAATAAAGATAGAGTCCCATTCTACATGTCAATACCGGCAACAATGAAATTTATAGTAAGAGGAAAATCCGTCGACTTTAGAAATCGTGAGGGTTCAAGTCCCTCTATCCCCAAAAAACCCATATGGACTCCCTAATTATTTATCCTCTCCTT-TTATCC-TTTTTTGTTAGCGGTTAAAAATTCG------TTATCTTTCTCATTCACCCTACTCTTTTACAAAGAGATCTGAGCGGAAATGTTTTTCTCTTATC----ACAAGTCTTGTGATCTAAG----ATAA-TACGTGTACAAATGAACATCTTTGAGTAAGGAATCCCCATTTGAATGATTCATGGTCAA------TGTCATTATTCATACTGAAACTTACAAAGTCTTCCTT-TTGAAGATCCAAGAAATTCCAGGACCTGGATAAGACTTTGTAAGACCCTTTCAATTGACA-TAGACCCGAGTTATCTAGC--AAAATGAGGATGCAGCGGTATTTATGTTAATGCACTTCCCAATGATACGTAAACAAGGCATTTCTGGTCCTTTA--------TAGAGAAGATCTATCATAGATATTTGTAATCTATCATTTATCACTTGGGGGAGGAACAATAGTATTTCATTGCTACAAGTATGGATTATTGAGAATAATAAGACATGTATTTGGATATTTCCCTTGAACTCCGCAATCTTTTTTATTTGACATGGATAGTTGAAGGGAATTTTCCGAAGAAAAAATGGATTAGATTATGGGAGTGTGTGACTTGAACTATTGATTGGTCTGTGCAGATATATGCCTTTCTCTATCTGCCACATTGGAATTCACAACCAAATGTGTCTTTGTTCCAACCATTGCGTAAGCCC----------CATACAGAGGATAGGCTGGTTCACTTGAAGAGAATCTTTTCTATGATCAGATCCGAATCATGTCGTACATGAGCAGGTTCCGTAAGATCCAGTAG-----AATAAGTG-AACTAGATAACCCATAATCTCGATTATGGGTTATCTAG-TTCACTTACATACGATTGAATAGTATGGAAATGCATTCATTTCCTATGCATTGACACGATCTATGATACTATCGGAGTGAAACAGGGGATCTAAGGAAGAACAGAGGCTAGGCTATATTAGTAACAAGTAAACCC-----TTTGTGTGTCTGTCAAAAGTCTCCAAGTATTTTGGGGATAAACACCGATCCTAAGGTCTGAGACGACCCAGAAAGCATT--TGATCCTATCATGATCCACCTTGTAAGCCTACTTGGGTATTGAGTATTTACTTGTAAGAACCAAATTTTTTGCGCTGGATAGTTGCAACTCCGGAAAAAGAAATCC------------------AGTCAAATTTTT-------TTTTAAT--------AAAATCATTCATATTATCATA-----TATGTGTGGATCTAGATAACATATAGATTTTATATGGATTCCTTATGGTTCTTTTTCTTTTTGCTCGAGCCGTATGATGAAAAATTATCATGTCCGGTTCCTTCGGGGGATGGAT-----CTATAAAAATTCACCTATCCCAATAACAAAAAAACCTGACCTGAATGATCCTGTATTAAGAGCTAAATTGGCTAAAGGTATGGGTCATAATTATTACGGAGAGCCCGCATGGCCCAATGATCTTTTATATATTTTTCCAGTAGAGATTG-----------------------AAG-----AAATCAATGCAATA-TTTTTAGGAAAGATAAAACTGGATGAATTCAAATTCGTTCAAATGGG-----------------------ATTGGAAGGTTCCTTATTTTCATTTAGGGTGTTCGTTTTATTTCTTCCCTTAGGACTTTGGTCTAGTTTAGGCTCTCTCTCCTGGAATCG-AATTGTTGT-AACTGGACGCTTCCATCCT-CTAACTAGTA--GGGATAGAAC------AAAAAAATATTTTCA------TTTTTTAATGAATTCTTTCTCATTTATCCGATTTATCAAATTTGAAACAAAAAGATACATTTTTTCAATGAACACA----------AAAAAATCCTAAAGTT---ATAGTTATACTATACAAAAGGTTGTCAAAATGGAATCAATTAGTTTCACCAATTCCTTA-----------------------ATTTTTACTAATGATCTTACA----TATGCCC-------TTCTATAGATAT----AGAG----------------ATAGAGAAACCC----------------------------ATTTTTCT-------TATTATA-----GATAAATAGGTTGATGGGGAAAATAAGACCCCGC-------CCTCGAAATGATAAAATCTACTAAAAAGAAAGGTAAAACCTTGTATCTTGTCTTTATTC---------------TTTTTTCAAAAGCA--------TTCTTTTTCTAATTTAGAAATTTAGTAAACAGAAGCATTTTTATTCTACTTCCATTCCCTATTCATT--GGCCGA-----AAACAATAGGGAATGGGAATTATTCATTTTATTTTTAAATTAACAATGAAATCAGAC---------AGTCAAATCAATTGGGATTATTCCAACGTTTTATGA--------CTTATTTGTTTGTCGTACAAAAAAACCTTTTACATTCCCCGTAGGTCGTACAAAAAAAC-TTTTTGAATTCCCGGT-AGAAAG------------------------------------------CATACACATGAAGTAA-----GAAAAAAGTC--TTG-CTTTCTCTTTCTTTATGATATAGATATGTAATATGTACAACTTTGACCAGCAATTTCATTTAGATC-TAAGTAAGGGCTCGAAAGAT-CCAATAGACAAAT-------------ATAAAGAAAAATAAAGAAGACCC-----CTTTGATTTTGTTCCCTTTATTCCCACGGCCTGGCCTGGTCAATACCTAGCCGGGCC----TTTTTTTGTTCCAACAAATCCTAGCTAAAAGAATTTAGCTG-------------CTTTGAACACA---------------AAAATGCTTGCTATTAAAGCAGC-------------------AATAAAAAGATGAGGGGTTATTTCCATTCTTACTTAT--------------------------------------TATATATATTATT-------------------------TATATATATAAATTATATAAAATCAAAGTATCCTTTCTTATT-----ATTCCTTCTTCCCTTTTGAGTTACTTGACGACCTTACGGGAAT--ATAAAATGAAACTG-----TGGGTTCTTA---AATAATAATGAATGCATTTTTCTGTTATGATTTCAGTGGTTTTAGTGAGCCATATCTATCAAAATCCCCCCAGCAAAAGAAAAAATAGAACTT--GTTATTTC----------------ATTTAGTTATTTAAAAGAGCCC-----TCCTTTCCAGAATCTCATTAAATTGAAATCCCCCGCAAA-AAACGT-CGACACTCTCATTTTCATGA-----TTATGATC-CTA--T-CTTTATTACGCTCAATTCCTCTT-------------------------------GTATTAG-TATATCTAATACCTTACCCAGCCCATTTGGAAATTTTGGTTCAAACTCTTCGCTACTGGGTAAAAGATGCCCCCTCTTTGCATTTATTACGATTCTTTCTTCACGACTATCGGCATTGGACTAGTCTTTTTATTCCAAAGAAAGCCAGTTCTTTTTTTTCAAAACGAAAG------------------CAAAGATTATTCTTCTTCCTATATAATTCTCATGTATGTGAATACGAATCCATCTTCGTCTTTCTCCGTAACCAATCTTCTCATTTACAATCAACATCTTCTGGAGCCCTTCTTGAACGAATATATTTCTATGAAAAAATAGAACATCTTGTCGAAGTCTTTGCTAAA-GATTTTCAAGGCAATCTATGGTTGTTCAAGGATCCTTTCATGCATTATGTTAGGTATCAAGGAAAGTCAATTCTCGCTTCAAAGGAGAACTTTCTTTTGATGCGTAAATGGAAATATTACTTTGTACGTTTCTGGCAATGTCATTTTTACCAGTGGTTTCAACCAGGAAGGATCTATATAAACCAATTATCCAAACATTCCCTCGACCTTCTGGGCTATCTTTCAAGTGTGCGGCTAAACCCTTTAACGATACGCAGTCAAATGTTAGAAAATTCATTTCTAATCGATAATGCTGTTAAGAAGTTCGATACCATTGTTCCAATTATTCCTCTGATTGGATCATTGGTTAAAGCGAAATTTTGTAACGTATTAGGGCATCCTGTTAGTAAGGTAGTTTGGGCAGATT--------??????????????????????????????????????????????????????????????????????????????????????????????????????????????????????????????????????????????????????????????????????????????????????????????????????????????????????????????????????????????????????????????????????????????????????????????????????????????????????????????????????????????????????????????????????????????????????????????????????????????????????????????????????????????????????????????????????????????????????????????????????????????????????????????????????????????????????????????????????????????????????????????????????????????????????????????????????????????????????????????????????????????????????????????????????????????????????????????????????????????????????????????????????????????????????????????????????????????????????????????????????????????????????????????????????????????????????????????????????????????????????????????????????????????????????????????????????????????????????????????????????????????????????????????????????????????????????????????????????????????????????????????????????????????????????????????????????????????????????TATTTAGATTA---------------------------------------------------------------------------------------------------------------------------------------------------------------------------------ATAAC------AATATTTATGATTTTTCTTT-AA-----------AAAAA--ATATGTAAGTCAA--------------------------------------------------------------------------------TGTGAAAT-AAAAAAGGAGCAATAATCCCCTTGTTGTTCTATCAAG-AGGGCGCTATTGCTCCTTTTTT-AT----TTCAAATACTCGTATACACTAAGGCCGGGTCTTATCCATTTATAGATGGAGCTTCAAGAGCAGCTAGGTCTAGAGGGAAGTTATGAGCATTACGTTCATGCATAACTTCCATACCAAGGTTAGCGCGGTTAATGATATCAGCCCAAGTATTAATTACACGACCTTGACTATCAACTACAGATTGGTTGAAATTAAAACCATTTAGGTTGAAAGCCATAGTGCTAATACCTAAAGCAGTGAACCAGATACCTACTACAGGCCAAGCAGCTAGGAAGAAATGTAAAGAACGAGAGTTGTTGAAACTAGCATATTGGAAGATCAATCGGCCAAAATAACCATGAGCAGCTACAATATTATAAGTTTCTTCTTCTTGA-CCGAATC---TGTAACCTTCATTAGCAATAAATGCAAGAATATTTAC-TTCCATAATCTCATCGTTTTTTTACTTCAAAATAACT-CGGGATTTAATCCCATAGAGATAATAAATCTTTCGCCTGTCAATTCA----ATGAATTACCTCTCGATGATC-TTGAAATCGGATCAATATCATGAATAACAATATCTGAGCTATCAAATCAATTCGTCGTCGAGAATTGAATAGTATAACATAGAAAGATCTTT-TATCCATACCG------AATCCAA------------------------AATTTCTTTATTTATCAATCATTCTTTTCTGTTCTTTCTTTATCTACAACCTATCTTAGGTCCTCCTTGTACAATCATCGGATAAAGTATCGTCTGACCGCCCGTCCGTTTCCATTAGTCACAAACGCCCAACAAACAATAGAAGCGAAGTGGAAAAAGAAATAAGTTACGTTCTAAACTCCG----TTTTTTTAATGATCTAGTTTTCTTGGAAGACAAAGAAGTGTGATAAAGAGGAGTTCCGGGATAAAGGATGTAATATTCCATCAAACTAACTATTTGAGTTTGGGTTTTGTTTGTTCTTCGACGGG--CCCT---------AAAAAAAATAG-AAAAAAAATAGGAAGGAAAAAT-GATTTATTCCCCTGCTACTTGCTAAGCTAAAAAAGGGGTGGGATCTTTGATTGATCTTTATTTTTCTTTTA--CCCCCCTTCCTT---------------------------------------------------------------------------------------------------------------------------------------------------------------------------------------------------------------------------------------------------------------------------------------------------------------AACGCTCTCAATAATTGTACTATTCTACATATGTCTTTCTCCTACCAATCCGTATTATTTGAAATAATGAAAATTCCCCTATTTGTTTGATGAGAAGTGC----GAAATGCCAAAGGAAA--GAAAAAAGAACCCCCTT-GGGAATG-AAATTCTGCTCCCCGTGCCCCCTTTAACAGAAAAGGG------------------------------------------------------------------------GGAACTCCTCAACCTGGAGTTCCACCTGAAGAAGCAGGGGCCGCGGTAGCTGCCGAATCTTCAACTGGTACATGGACAACTGTGTGGACCGATGGACTTACCAGCCTTGATCGTTACAAAGGGCGATGCTACCACATCGAGCCCGTTGCTGGAGAAGAAAATCAATTTATTGCTTATGTAGCTTACCCATTAGACCTTTTTGAAGAAGGTTCTGTTACTAACATGTTTACTTCTATTGTGGGTAATGTATTTGGGTTCAAAGCCCTGCGCGCTCTACGTCTGGAAGATCTGCGAATCCCTGCCGCTTATGCTAAAACTTTCCAAGGCCCGCCTCATGGCATCCAAGTTGAGAGAGATAAATTGAACAAGTATGGTCGCCCCCTGTTGGGATGTACTATTAAACCTAAATTGGGGTTATCTGCTAAAAACTATGGTAGAGCGGTTTATGAATGTCTACGTGGTGGACTTGATTTTACCAAAGATGATGAGAA--CGT--GAACTCCCAACCATTTATGCG-------------------

Vesalea_coriacea_var_subcoriacea_BOP022788 CCTGAGCCAAATCCAGTTTTACGAAAACAAACAAGGGTTCAGAAAGCTAAAATC-AAAAAGGATAGGTGCAGAGACTCAATGGAAGCTGTTCTAACAAATGGAGTTGAC-----TGTGTTGGTAGAAAGAATCCTTCCATAGAAACTTCAGAAAGGATAAACGTATAAACATAGATATACGCATTGAAATACTAT-ATACTCTACCAAATGATTAATGACGACCCGAATCTGTATT--TA-----TATATATCAAAATGGGAGAATGGTTGTGAAGTGATTCCATATTGAAAAAAGAATCGAATATTCATTGATCAAATCATTCACTCCATAGTCTGATAGATC-TTTTGAAGAACTGATTAATTGGACGAGAATAAAGATAGAGTCCCATTCTACATGTCAATACCGGCAACAATGAAATTTATAGTAAGAGGAAAATCCGTCGACTTTAGAAATCGTGAGGGTTCAAGTCCCTCTATCCCCAAAAAACCCATATGGACTCCCTAATTATTTATCCTCTCCTT-TTATCC-TTTTTTGTTAGCGGTTAAAAATTCG------TTATCTTTCTCATTCACCCTACTCTTTTACAAAGAGATCTGAGCGGAAATGTTTTTCTCTTATC----ACAAGTCTTGTGATCTAAG----ATAA-TACGTGTACAAATGAACATCTTTGAGTAAGGAATCCCCATTTGAATGATTCATGGTCAA------TGTCATTATTCATACTGAAACTTACAAAGTCTTCCTT-TTGAAGATCCAAGAAATTCCAGGACCTGGATAAGACTTTGTAAGACCCTTTCAATTGACA-TAGACCCGAGTTATCTAGC--AAAATGAGGATGCAGCGGTATTTATGTTAATGCACTTCCCAATGATACGTAAACAAGGCATTTCTGGTCCTTTA--------TAGAGAAGATCTATCATAGATATTTGTAATCTATCATTTATCACTTGGGGGAGGAACAATAGTATTTCATTGCTACAAGTATGGATTATTGAGAATAATAAGACATGTATTTGGATATTTCCCTTGAACTCCGCAATCTTTTTTATTTGACATGGATAGTTGAAGGGAATTTTCCGAAGAAAAAATGGATTAGATTATGGGAGTGTGTGACTTGAACTATTGATTGGTCTGTGCAGATATATGCCTTTCTCTATCTGCCACATTGGAATTCACAACCAAATGTGTCTTTGTTCCAACCATTGCGTAAGCCC----------CATACAGAGGATAGGCTGGTTCACTTGAAGAGAATCTTTTCTATGATCAGATCCGAATCATGTCGTACATGAGCAGGTTCCGTAAGATCCAGTAG-----AATAAGTG-AACTAGATAACCCATAATCTCGATTATGGGTTATCTAG-TTCACTTACATACGATTGAATAGTATGGAAATGCATTCATTTCCTATGCATTGACACGATCTATGATACTATCGGAGTGAAACAGGGGATCTAAGGAAGAACAGAGGCTAGGCTATATTAGTAACAAGAAAACCC-----TTTGTGTGTCTGTCAAAAGTCTCCAAGTATTTTGGGGATAAACACCGATCCTAAGGTCTGAGACGACCCAGAAAGCATT--TGATCCTATCATGATCCACCTTGTAAGCCTACTTGGGTATTGAGTATTTACTTGTAAGAACCAAATTTTTTGCGCTGGATAGTTGCAACTCCGGAAAAAGAAATCC------------------AGTCAAATTTTT-------TTTTAAT--------AAAATCATTCATATTATCATA-----TATGTGTGGATCTAGATAACATATAGATTTTATATGGATTCCTTATGGTTCTTTTTCTTTTTGCTCGAGCCGTATGATGAAAAATTATCATGTCCGGTTCCTTCGGGGGATGGAT-----CTATAAAAATTCACCTATCCCAATAACAAAAAAACCTGACCTGAATGATCCTGTATTAAGAGCTAAATTGGCTAAAGGTATGGGTCATAATTATTACGGAGAGCCCGCATGGCCCAATGATCTTTTATATATTTTTCCAGTAGAGATTG-----------------------AAG-----AAATCAATGCAATA-TTTTTAGGAAAGATAAAACTGGATGAATTCAAATTCGTTCAAATGGG-----------------------ATTGGAAGGTTCCTTATTTTCATTTAGGGTGTTCGTTTTATTTCTTCCCTTAGGACTTTGGTCTAGTTTAGGCTCTCTCTCCTGGAATCG-AATTGTTGA-AACTGGACGCTTCCATCCT-CTAGCTAGTA--GGGATAGAAC------AAAAAAATATTTTCA------TTTTTTAATGAATTCTTTCTCATTTATCCGATTTATCAAATTTGAAACAAAAAGATACATTTTTTCAATGAACACA----------AAAAAATCCTAAAGTT---------ATACTATACAAAAGGTTGTCAAAATGGAATCAATTAGTTTCACCAATTCCTTA-----------------------ATTTTGACTAATGATCTTACA----TATGCCC-------TTCTATAGATAT----AGAG----------------ATAGAGAAACCC----------------------------ATTTTTCT-------TATTATA-----GATAAATAGGTTGATGGGGAAAATAAGACCCCGC-------CCTCGAAATGATAAAATCTACTAAAAAGAAAGGTAAAACCTTGTATCTTGTCTTTATTC---------------TTTTTTCAAAAGCA--------TTCTTTTTCTAATTTAGAAATTTAGTAAACAGAAGCATTTTTATTCTACTTCCATTCCCTATTGTTTTCGGCC-------AATGAATAGGGAATGGGAATTATTCATTTTATTTTTAAATTAACAATGAAATCAGAC---------AGTCAAATCAATTGGGATTATTCCAACGTTTTATGA--------CTTATTTGTTTGTCGTACAAAAAAACCTTTTACATTCCCCGTAGGTCGTACAAAAAAAC-TTTTTGAATTCCCGGT-AGAAAG------------------------------------------CATACACATGAAGTAA-----GAAAAAAGTC--TTG-CTTTCTCTTTCTTTATGATATAGATATGTAATATGTACAACTTTGACCAGCAATTTCATTTAGATC-TAAGTAAGGGCTCGAAAGAT-CCAATAGACAAAT-------------ATAAAGAAAAATAAAGAAGACCC-----CTTTGATTTTGTTCCCTTTATTCCCACGGCCTGGCCTGGTCAATACCTAGCCGGGCC----TTTTTTTGTTCCAACAAATCCTAGCTAAAAGAATTTAGCTG-------------CTTTGAACACA---------------AAAATGCTTGCTATTAAAGCAGC-------------------AATAAAAAGATGAGGGGTTATTTCCATTCTTACTTAT--------------------------------------TATA--TATTATATATATTATT----------------TATATATATAAATTATATAAAATCAAAGTATCCTTTCTTATT-----ATTCCTTCTTCCCTTTTGAGTTACTTGACGACCTTACGGGAAT--ATAAAATGAAACTG-----TGGGTTCTTA---AATAATAATGAATGCATTTTTCTGTTATGATTTCAGTGGTTTTAGTGAGCCATATCTATCAAAATCCCCCCAGCAAAAGAAAAAATAGAACTT--GTTATTTC----------------ATTTAGTTATTTAAAAGAGCCC-----TCCTTTCCAAAATCTCATTAAATTGAAATCCCCCGCAAA-AAACGT-CGACACTCTCATTTTCATGA-----TTATGATC-CTA--T-CTTTATTACGC-------------------------------------------GTATTAG-TATATCTAATACCTTACCCAGCCCATTTGGAAATTTTGGTTCAAACTCTTCGCTACTGGGTAAAAGATGCCCCCTCTTTGCATTTATTACGATTCTTTCTTCACGACTATCGGCATTGGACTAGTCTTTTTATTCCAAAGAAAGCCAGTTCTTTTTTTTCAAAACGAAAG------------------CAAAGATTATTCTTCTTCCTATATAATTCTCATGTATGTGAATACGAATCCATCTTCGTCTTTCTCCGTAACCAATCTTCTCATTTACAATCAACATCTTCTGGAGCCCTTCTTGAACGAATATATTTCTATGAAAAAATAGAACATCTTGTCGAAGTCTTTGCTAAA-GATTTTCAAGGCAATCTATGGTTGTTCAAGGATCCTTTCATGCATTATGTTAGGTATCAAGGAAAGTCAATTCTCGCTTCAAAGGAGAACTTTCTTTTGATGCGTAAATGGAAATATTACTTTGTACGTTTCTGGCAATGTCATTTTTACCAGTGGTTTCAACCAGGAAGGATCTATATAAACCAATTATCCAAACATTCCCTCGACCTTCTGGGCTATCTTTCAAGTGTGCGGCTAAACCCTTTAACGATACGCAGTCAAATGTTAGAAAATTCATTTCTAATCGATAATGCTGTTAAGAAGTTCGATACCATTGTTCCAATTATTCCTCTGATTGGATCATTGGTTAAAGCGAAATTTTGTAACGTATTAGGGCATCCTGTTAGTAAGGTAGTTTGGGCAG-----------??????????????????????????????????????????????????????????????????????????????????????????????????????????????????????????????????????????????????????????????????????????????????????????????????????????????????????????????????????????????????????????????????????????????????????????????????????????????????????????????????????????????????????????????????????????????????????????????????????????????????????????????????????????????????????????????????????????????????????????????????????????????????????????????????????????????????????????????????????????????????????????????????????????????????????????????????????????????????????????????????????????????????????????????????????????????????????????????????????????????????????????????????????????????????????????????????????????????????????????????????????????????????????????????????????????????????????????????????????????????????????????????????????????????????????????????????????????????????????????????????????????????????????????????????????????????????????????????????????????????????????????????????????????????????????????????????????????????????????TATTTAGATTA---------------------------------------------------------------------------------------------------------------------------------------------------------------------------------ATAAA------AATATTTACGATTTTTCTTT-AA-----------AAAAA--ATATGTAAGTCAA--------------------------------------------------------------------------------TGTGAAAT-AAAAAAGGAGCAATAATCCCCTTGTTATTCTATCAAG-AGGGCGCTATTGCTCCTTTTTT-AT----TTCAAATACTCGTATACACTAAGGCCGGGTCTTATCCATTTATAGATGGAGCTTCAAGAGCAGCTAGGTCTAGAGGGAAGTTATGAGCATTACGTTCATGCATAACTTCCATACCAAGGTTAGCGCGGTTAATGATATCAGCCCAAGTATTAATTACACGACCTTGACTATCAACTACAGATTGGTTGAAATTAAAACCATTTAGGTTGAAAGCCATAGTGCTAATACCTAAAGCAGTGAACCAGATACCTACTACAGGCCAAGCAGCTAGGAAGAAATGTAAAGAACGAGAGTTGTTGAAACTAGCATATTGGAAGATCAATCGGCCAAAATAACCATGAGCAGCTACAATATTATAAGTTTCTTCTTCTTGA-CCGAATC---TGTAACCTTCATTAGCAATAAATGCAAGAATATTTAC-TTCCATAATCTCATCGTTTTTTTACTTCAAAATAACT-CGGGATTTAATCCCATAGAGATAATAAATCTTTCGCCTGTCAATTCA----ATGAATTACCTCTCGATGATC-TTGAAATCGGATCAATATCATGAATAACAATATCTGAGCTATCAAATCAATTCGTCGTCGAGAATTGAATAGTATAACATAGAAAGATCTTT-TATCCATACCG------AATCCAA------------------------AATTTCTTTATTTATCAATCATTCTTTTCTGTTCTTTCTTTATCTACAACCTATCTTAGGTCCTCCTTGTACAATCATCGGATAAAGTATCGTCTGACCGCCCGTCCGTTTCCATTAGTCACAAACGCCCAACAAACAATAGAAGCGAAGTGGAAAAAGAAATAAGTTACGTTCTAAACTCCG----TTTTTTTAATGATCTAGTTTTCTTGGAAGACAAAGAAGTGTGATAAAGAGGAGTTCCGGGATAAAGGATGTAATATTCCATCAAACTAACTATTTGAGTTTGGGTTTTGTTTGTTCTTCGACGGG--CCCT--------AAAAAAAAATAG-AAAAAAAATAGGAAGGAAAAAT-GATTTATTCCCCTGCTACTTGCTAAGCTAAAAAAGGGGTGGGATCTTTGATTGATCTTTATTTTTCTTTTA-CCCCCCCTTCCTT---------------------------------------------------------------------------------------------------------------------------------------------------------------------------------------------------------------------------------------------------------------------------------------------------------------AACGCTCTCAATAATTGTACTATTCTACATATGTCTTTCTCCTACCAATCCGTATTATTTGAAATAATGAAAATTCCCCTATTTGTTTGATGAGAAGTGC----GAAATGCCAAAGGAAA--GAAAAAAGAACCCCCTT-GGGAATG-AAATTCTGCTCCCCGTGCCCCCTTTAACAGAAAAGGG-------------------------------------------------------------------------TAACTCCTCAACCTGGAGTTCCACCTGAAGAAGCAGGGGCCGCGGTAGCTGCCGAATCTTCAACTGGTACATGGACAACTGTGTGGACCGATGGACTTACCAGCCTTGATCGTTACAAAGGGCGATGCTACCACATCGAGCCCGTTGCTGGAGAAGAAACTCAATTTATTGCTTATGTAGCTTACCCATTAGACCTTTTTGAAGAAGGTTCTGTTACTAACATGTTTACTTCTATTGTGGGTAATGTATTTGGGTTCAAAGCCCTGCGCGCTCTACGTCTGGAAGATCTGCGAATCCCTGCCGCTTATGCTAAAACTTTCCAAGGCCCGCCTCATGGCATCCAAGTTGAGAGAGATAAATTGAACAAGTATGGTCGCCCCCTGTTGGGATGTACTATTAAACCTAAATTGGGGTTATCTGCTAAAAACTATGGTAGAGCGGTTTATGAATGTCTACGTGGTGGACTTGATTTTACCAAAGATGATGAGAA--CGT--GAACTCTCAACCATTTATGC--------------------

Vesalea_floribunda_BOP022783 CCTGAGCCAAATCCAGTTTTACGAAAACAAACAAGGGTTCAGAAAGCTAAAATC-AAAAAGGATAGGTGCAGAGACTCAATGGAAGCTGTTCTAACAAATGGAGTTGAC-----TGTGTTGGTAGAAAGAATCCTTCCATAGAAACTTCAGAAAGGATAAACGTATAAACATAGATATACGCATTGAAATACTAT-ATACTCTACCAAATGATTAATGACGACCCGAATCTGTATT--TA-----TATATATCAAAATGGGAGAATGGTTGTGAAGTGATTCCATATTGAAAAAAGAATCGAATATTCATTGATCAAATCATTCACTCCATAGTCTGATAGATC-TTTTGAAGAACTGATTAATTGGACGAGAATAAAGATAGAGTCCCATTCTACATGTCAATACCGGCAACAATGAAATTTATAGTAAGAGGAAAATCCGTCGACTTTAGAAATCGTGAGGGTTCAAGTCCCTCTATCCCCAAAAAACCCATATGGACTCCCTAATTATTTATCCTCTCCTT-TTATCC-TTTTTTGTTAGCGGTTAAAAATTCG------TTATCTTTCTCATTCACCCTACTCTTTTACAAAGAGATCTGAGCGGAAATGTTTTTCTCTTATC----ACAAGTCTTGTGATCTAAG----ATAA-TACGTGTACAAATGAACATCTTTGAGTAAGGAATCCCCATTTGAATGATTCATGGTCAA------TGTCATTATTCATACTGAAACTTACAAAGTCTTCCTT-TTGAAGATCCAAGAAATTCCAGGACCTGGATAAGACTTTGTAAGACCCTTTCAATTGACA-TAGACCCGAGTTATCTAGC--AAAATGAGGATGCAGCGGTATTTATGTTAATGCACTTCCCAATGATACGTAAACAAGGCATTTCTGGTCCTTTA--------TAGAGAAGATCTATCATAGATATTTGTAATCTATCATTTATCACTTGGGGGAGGAACAATAGTATTTCATTGCTACAAGTATGGATTATTGAGAATAATAAGACATGTATTTGGATATTTCCCTTGAACTCCGCAATCTTTTTTATTTGACATGGATAGTTGAAGGGAATTTTCCGAAGAAAAAATGGATTAGATTATGGGAGTGTGTGACTTGAACTATTGATTGGTCTGTGCAGATATATGCCTTTCTCTATCTGCCACATTGGAATTCACAACCAAATGTGTCTTTGTTCCAACCATTGCGTAAGCCC----------CATACAGAGGATAGGCTGGTTCACTTGAAGAGAATCTTTTCTATGATCAGATCCGAATCATGTCGTACATGAGCAGGTTCCGTAAGATCCAGTAG-----AATAAGTG-AACTAGATAACCCATAATCTCGATTATGGGTTATCTAG-TTCACTTACATACGATTGAATAGTATGGAAATGCATTCATTTCCTATGCATTGACACGATCTATGATACTATCGGAGTGAAACAGGGGATCTAAGGAAGAACAGAGGCTAGGCTATATTAGTAACAAGTAAACCC-----TTTGTGTGTCTGTCAAAAGTCTCCAAGTATTTTGGGGATAAACACCGATCCTAAGGTCTGAGACGACCCAGAAAGCATT--TGATCCTATCATGATCCACCTTGTAAGCCTACTTGGGTATTGAGTATTTACTTGTAAGAACCAAATTTTTTGCGCTGGATAGTTGCAACTCCGGAAAAAGAAATCC------------------AGTCAAATTTTT-------TTTTTAT--------AAAATCATTCATATTATCATA-----TATGTGTGGATCTAGATAACATATAGATTTTATATGGATTCCTTATGGTTCTTTTTCTTTTTGCTCGAGCCGTATGATGAAAAATTATCATGTCCGGTTCCTTCGGGGGATGGAT-----CTATAAAAATTCACCTATCCCAATAACAAAAAAACCTGACCTGAATGATCCTGTATTAAGAGCTAAATTGGCTAAAGGTATGGGTCATAATTATTACGGAGAGCCCGCATGGCCCAATGATCTTTTATATATTTTTCCAGTAGAGATTG-----------------------AAG-----AAATCAATGCAATA-TTTTTAGGAAAGATAAAACTGGATGAATTCAAATTCGTTCAAATGGG-----------------------ATTGGAAGGTTCCTTATTTTCATTTAGGGTGTTCGTTTTATTTCTTCCCTTAGGACTTTGGTCTAGTTTAGGCTCTCTCTCCTGGAATCG-AATTGTTGT-AACTGGACGCTTCCATCCT-CTAACTAGTA--GGGATAGAAC------AAAAAAATATTTTCA------TTTTTTAATGAATTCTTTCTCATTTATCCGATTTATCAAATTTGAAACAAAAAGATACATTTTTTCAATGAACACA----------AAAAAATCCTAAAGTT---ATAGTTATACTATACAAAAGGTTGTCAAAATGGAATCAATTAGTTTCACCAATTCCTTA-----------------------ATTTTTACTAATGATCTTACA----TATGCCC-------TTCTATAGATAT----AGAG----------------ATAGAGAAACCC----------------------------ATTTTTCT-------TATTATA-----GATAAATAGGTTGATGGGGAAAATAAGACCCCGC-------CCTCGAAATGATAAAATCTACTAAAAAGAAAGGTAAAACCTTGTATCTTGTCTTTATTC---------------TTTTTTCAAAAGCA--------TTCTTTTTCTAATTTAGAAATTTAGTAAACAGAAGCATTTTTATTCTACTTCCATTCCCTATTCATT--GGCCGA-----AAACAATAGGGAATGGGAATTATTCATTTTATTTTTAAATTAACAATGAAATCAGAC---------AGTCAAATCAATTGGGATTATTCCAACGTTTTATGA--------CTTATTTGTTTGTCGTACAAAAAAACCTTTTACATTCCCCGTAGGTCGTACAAAAAAAC-TTTTTGAATTCCCGGT-AGAAAGTCTATGT-------------------------------TTACATTACACATGAAGTAA-----GAAAAAAGTC--TTG-CTTTCTCTTTCTTTATGATATAGATATGTAATATGTACAACTTTGACCAGCAATTTCATTTAGATC-TAAGTAAGGGCTCGAAAGAT-CCAATAGACAAAT-------------ATAAAGAAAAATAAAGAAGACCC-----CTTTGATTTTGTTCCCTTTATTCCCACGGCCTGGCCTGGTCAATACCTAGCCGGGCC----TTTTTTTGTTCCAACAAATCCTAGCTAAAAGAATTTAGCTG-------------CTTTGAACACA---------------AAAATGCTTGCTATTAAAGCAGC-------------------AATAAAAAGATGAGGGGTTATTTCCATTCTTACTTAT--------------------------------------TATATATATTATTTATATA-------------------TATATATATAAATTATATAAAATCAAAGTATCCTTTCTTATT-----ATTCCTTCTTCCCTTTTGAGTTACTTGACGACCTTACGGGAAT--ATAAAATGAAACTG-----TGGGTTCTTA---AATAATAATGAATGCATTTTTCTGTTATGATTTCAGTGGTTTTAGTGAGCCATATCTATCAAAATCCCCCCAGCAAAAGAAAAAATAGAACTT--GTTATTTC----------------ATTTAGTTATTTAAAAGAGCCC-----TCCTTTCCAGAATCTCATTAAATTGAAATCCCCCGCAAA-AAACGT-CGACACTCTCATTTTCATGA-----TTATGATC-CTA--T-CTTTATTACGCTCAATTCCTCTT-------------------------------GTATTAG-TATATCTAATACCTTACCCAGCCCATTTGGAAATTTTGGTTCAAACTCTTCGCTACTGGGTAAAAGATGCCCCCTCTTTGCATTTATTACGATTCTTTCTTCACGACTATCGGCATTGGACTAGTCTTTTTATTCCAAAGAAAGCCAGTTCTTTTTTTTCAAAACGAAAG------------------CAAAGATTATTCTTCTTCCTATATAATTCTCATGTATGTGAATACGAATCCATCTTCTTCTTTCTCCGTAACCAATCTTCTCATTTACAATCAACATCTTCTGGAGCCCTTCTTGAACGAATATATTTCTATGAAAAAATAGAACATCTTGTCGAAGTCTTTGCTAAA-GATTTTCAAGGCAATCTATGGTTGTTCAAGGATCCTTTCATGCATTATGTTAGGTATCAAGGAAAGTCAATTCTCGCTTCAAAGGAGAACTTTCTTTTGATGCGTAAATGGAAATATTACTTTGTACGTTTCTGGCAATGTCATTTTTACCAGTGGTTTCAACCAGGAAGGATCTATATAAACCAATTATCCAAACATTCCCTCGACCTTCTGGGCTATCTTTCAAGTGTGCGGCTAAACCCTTTAACGATACGCAGTCAAATGTTAGAAAATTCATTTCTAATCGATAATGCTGTTAAGAAGTTCGATACCATTGTTCCAATTATTCCTCTGATTGGATCATTGGTTAAAGCGAAATTTTGTAACGTATTAGGGCATCCTGTTAGTACGGTAGTTTGGGCAGATTTATCAGAT??????????????????????????????????????????????????????????????????????????????????????????????????????????????????????????????????????????????????????????????????????????????????????????????????????????????????????????????????????????????????????????????????????????????????????????????????????????????????????????????????????????????????????????????????????????????????????????????????????????????????????????????????????????????????????????????????????????????????????????????????????????????????????????????????????????????????????????????????????????????????????????????????????????????????????????????????????????????????????????????????????????????????????????????????????????????????????????????????????????????????????????????????????????????????????????????????????????????????????????????????????????????????????????????????????????????????????????????????????????????????????????????????????????????????????????????????????????????????????????????????????????????????????????????????????????????????????????????????????????????????????????????????????????????????????????????????????????????????????TATTTAGATTA---------------------------------------------------------------------------------------------------------------------------------------------------------------------------------ATAAC------AATATTTATGATTTTTCTTT-AA----------AAAAAA--ATATGTAAGTCAA--------------------------------------------------------------------------------TGTGAAAT-AAAAAAGGAGCAATAATCCCCTTGTTGTTCTATCAAG-AGGGCGCTATTGCTCCTTTTTT-AT----TTCAAATACTCGTATACACTAAGGCCGGGTCTTATCCATTTATAGATGGAGCTTCAAGAGCAGCTAGGTCTAGAGGGAAGTTATGAGCATTACGTTCATGCATAACTTCCATACCAAGGTTAGCGCGGTTAATGATATCAGCCCAAGTATTAATTACACGACCTTGACTATCAACTACAGATTGGTTGAAATTAAAACCATTTAGGTTGAAAGCCATAGTGCTAATACCTAAAGCAGTGAACCAGATACCTACTACAGGCCAAGCAGCTAGGAAGAAATGTAAAGAACGAGAGTTGTTGAAACTAGCATATTGGAAGATCAATCGGCCAAAATAACCATGAGCAGCTACAATATTATAAGTTTCTTCTTCTTGA-CCGAATC---TGTAACCTTCATTAGCAATAAATGCAAGAATATTTAC-TTCCATAATCTCATCGTTTTTTTACTTCAAAATAACT-CGGGATTTAATCCCATAGAGATAATAAATCTTTCGCCTGTCAATTCA----ATGAATTACCTCTCGATGATC-TTGAAATCGGATCAATATCATGAATAACAATATCTGAGCTATCAAATCAATTCGTCGTCGAGAATTGAATAGTATAACATAGAAAGATCTTT-TATCCATACCG------AATCCAA------------------------AATTTCTTTATTTATCAATCATTCTTTTCTGTTCTTTCTTTATCTACAACCTATCTTAGGTCCTCCTTGTACAATCATCGGATAAAGTATCGTCTGACCGCCCGTCCGTTTCCATTAGTCACAAACGCCCAACAAACAATAGAAGCGAAGTGGAAAAAGAAATAAGTTACGTTCTAAACTCCG----TTTTTTTAATGATCTAGTTTTCTTGGAAGACAAAGAAGTGTGATAAAGAGGAGTTCCGGGATAAAGGATGTAATATTCCATCAAACTAACTATTTGAGTTTGGGTTTTGTTTGTTCTTCGACGGG--CCCT---------AAAAAAAATAG-AAAAAAAATAGGAAGGAAAAAT-GATTTATTCCCCTGCTACTTGCTAAGCTAAAAAAGGGGTGGGATCTTTGATTGATCTTTATTTTTCTTTTA--CCCCCCTTCCTT---------------------------------------------------------------------------------------------------------------------------------------------------------------------------------------------------------------------------------------------------------------------------------------------------------------AACGCTCTCAATAATTGTACTATTCTACATATGTCTTTCTCCTACCAATCCGTATTATTTGAAATAATGAAAATTCCCCTATTTGTTTGATGAGAAGTGC----GAAATGCCAAAGGAAA--GAAAAAAGAACCCCCTT-GGGAATG-AAATTCTGCTCCCCGTGCCCCCTTTAACAGAAAAGGG--------------------------------------------------------------------------GACTCCTCAACCTGGAGTTCCACCTGAAGAAGCAGGGGCCGCGGTAGCTGCCGAATCTTCAACTGGTACATGGACAACTGTGTGGACCGATGGACTTACCAGCCTTGATCGTTACAAAGGGCGATGCTACCACATCGAGCCCGTTGCTGGAGAAGAAAATCAATTTATTGCTTATGTAGCTTACCCATTAGACCTTTTTGAAGAAGGTTCTGTTACTAACATGTTTACTTCTATTGTGGGTAATGTATTTGGGTTCAAAGCCCTGCGCGCTCTACGTCTGGAAGATCTGCGAATCCCTGCCGCTTATGCTAAAACTTTCCAAGGCCCGCCTCATGGCATCCAAGTTGAGAGAGATAAATTGAACAAGTATGGTCGCCCCCTGTTGGGATGTACTATTAAACCTAAATTGGGGTTATCTGCTAAAAACTATGGTAGAGCGGTTTATGAATGTCTACGTGGTGGACTTGATTTTACCAAAGATGATGAGAA--CGT--GAACTCCCAACCATTTATGC--------------------

Vesalea_grandifolia_BOP022782 CCTGAGCCAAATCCAGTTTTACGAAAACAAACAAGGGTTCAGAAAGCTAAAATC-AAAAAGGATAGGTGCAGAGACTCAATGGAAGCTGTTCTAACAAATGGAGTTGAC-----TGTGTTGGTAGAAAGAATCCTTCCATAGAAACTTCAGAAAGGATAAACGTATAAACATAGATATACGCATTGAAATACTAT-ATACTCTACCAAATGATTAATGACGACCCGAATCTGTATT--TA-----TATATATCAAAATGGGAGAATGGTTGTGAAGTGATTCCATATTGAAAAAAGAATCGAATATTCATTGATCAAATCATTCACTCCATAGTCTGATAGATC-TTTTGAAGAACTGATTAATTGGACGAGAATAAAGATAGAGTCCCATTCTACATGTCAATACCGGCAACAATGAAATTTATAGTAAGAGGAAAATCCGTCGACTTTAGAAATCGTGAGGGTTCAAGTCCCTCTATCCCCAAAAAACCCATATGGACTCCCTAATTATTTATCCTCTCCTT-TTATCC-TTTTTTGTTAGCGGTTAAAAATTCG------TTATCTTTCTCATTCACCCTACTCTTTTACAAAGAGATCTGAGCGGAAATGTTTTTCTCTTATC----ACAAGTCTTGTGATCTAAG----ATAA-TACGTGTACAAATGAACATCTTTGAGTAAGGAATCCCCATTTGAATGATTCATGGTCAA------TGTCATTATTCATACTGAAACTTACAAAGTCTTCCTT-TTGAAGATCCAAGAAATTCCAGGACCTGGATAAGACTTTGTAAGACCCTTTCAATTGACA-TAGACCCGAGTTATCTAGC--AAAATGAGGATGCAGCGGTATTTATGTTAATGCACTTCCCAATGATACGTAAACAAGGCATTTCTGGTCCTTTA--------TAGAGAAGATCTATCATAGATATTTGTAATCTATCATTTATCACTTGGGGGAGGAACAATAGTATTTCATTGCTACAAGTATGGATTATTGAGAATAATAAGACATGTATTTGGATATTTCCCTTGAACTCCGCAATCTTTTTTATTTGACATGGATAGTTGAAGGGAATTTTCCGAAGAAAAAATGGATTAGATTATGGGAGTGTGTGACTTGAACTATTGATTGGTCTGTGCAGATATATGCCTTTCTCTATCTGCCACATTGGAATTCACAACCAAATGTGTCTTTGTTCCAACCATTGCGTAAGCCC----------CATACAGAGGATAGGCTGGTTCACTTGAAGAGAATCTTTTCTATGATCAGATCCGAATCATGTCGTACATGAGCAGGTTCCGTAAGATCCAGTAG-----AATAAGTG-AACTAGATAACCCATAATCTCGATTATGGGTTATCTAG-TTCACTTACATACGATTGAATAGTATGGAAATGCATTCATTTCCTATGCATTGACACGATCTATGATACTATCGGAGTGAAACAGGGGATCTAAGGAAGAACAGAGGCTAGGCTATATTAGTAACAAGTAAACCC-----TTTGTGTGTCTGTCAAAAGTCTCCAAGTATTTTGGGGATAAACACCGATCCTAAGGTCTGAGACGACCCAGAAAGCATT--TGATCCTATCATGATCCACCTTGTAAGCCTACTTGGGTATTGAGTATTTACTTGTAAGAACCAAATTTTTTGCGCTGGATAGTTGCAACTCCGGAAAAAGAAATCC------------------AGTCAAATTTTT-------TTTTAAT--------AAAATCATTCATATTATCATA-----TATGTGTGGATCTAGATAACATATAGATTTTATATGGATTCCTTATGGTTCTTTTTCTTTTTGCTCGAGCCGTATGATGAAAAATTATCATGTCCGGTTCCTTCGGGGGATGGAT-----CTATAAAAATTCACCTATCCCAATAACAAAAAAACCTGACCTGAATGATCCTGTATTAAGAGCTAAATTGGCTAAAGGTATGGGTCATAATTATTACGGAGAGCCCGCATGGCCCAATGATCTTTTATATATTTTTCCAGTAGAGATTG-----------------------AAG-----AAATCAATGCAATA-TTTTTAGGAAAGATAAAACTGGATGAATTCAAATTCGTTCAAATGGG-----------------------ATTGGAAGGTTCCTTATTTTCATTTAGGGTGTTCGTTTTATTTCTTCCCTTAGGACTTTGGTCTAGTTTAGGCTCTCTCTCCTGGAATCG-AATTGTTGT-AACTGGACGCTTCCATCCT-CTAACTAGTA--GGGATAGAAC------AAAAAAATATTTTCA------TTTTTTAATGAATTCTTTCTCATTTATCCGATTTATCAAATTTGAAACAAAAAGATACATTTTTTCAATGAACACA----------AAAAAATCCTAAAGTT---ATAGTTATACTATACAAAAGGTTGTCAAAATGGAATCAATTAGTTTCACCAATTCCTTA-----------------------ATTTTTACTAATGATCTTACA----TATGCCC-------TTCTATAGATAT----AGAG----------------ATAGAGAAACCC----------------------------ATTTTTCT-------TATTATA-----GATAAATAGGTTGATGGGGAAAATAAGACCCCGC-------CCTCGAAATGATAAAATCTACTAAAAAGAAAGGTAAAACCTTGTATCTTGTCTTTATTC---------------TTTTTTCAAAAGCA--------TTCTTTTTCTAATTTAGAAATTTAGTAAACAGAAGCATTTTTATTCTACTTCCATTCCCTATTCATT--GGCCGA-----AAACAATAGGGAATGGGAATTATTCATTTTATTTTTAAATTAACAATGAAATCAGAC---------AGTCAAATCAATTGGGATTATTCCAACGTTTTATGA--------CTTATTTGTTTGTCGTACAAAAAAACCTTTTACATTCCCCGTAGGTCGTACAAAAAAAC-TTTTTGAATTCCCGGT-AGAAAG------------------------------------------CATACACATGAAGTAA-----GAAAAAAGTC--TTG-CTTTCTCTTTCTTTATGATATAGATATGTAATATGTACAACTTTGACCAGCAATTTCATTTAGATC-TAAGTAAGGGCTCGAAAGAT-CCAATAGACAAAT-------------ATAAAGAAAAATAAAGAAGACCC-----CTTTGATTTTGTTCCCTTTATTCCCACGGCCTGGCCTGGTCAATACCTAGCCGGGCC----TTTTTTTGTTCCAACAAATCCTAGCTAAAAGAATTTAGCTG-------------CTTTGAACACA---------------AAAATGCTTGCTATTAAAGCAGC-------------------AATAAAAAGATGAGGGGTTATTTCCATTCTTACTTAT--------------------------------------TATATATATTATT-------------------------TATATATATAAATTATATAAAATCAAAGTATCCTTTCTTATT-----ATTCCTTCTTCCCTTTTGAGTTACTTGACGACCTTACGGGAAT--ATAAAATGAAACTG-----TGGGTTCTTA---AATAATAATGAATGCATTTTTCTGTTATGATTTCAGTGGTTTTAGTGAGCCATATCTATCAAAATCCCCCCAGCAAAAGAAAAAATAGAACTT--GTTATTTC----------------ATTTAGTTATTTAAAAGAGCCC-----TCCTTTCCAGAATCTCATTAAATTGAAATCCCCCGCAAA-AAACGT-CGACACTCTCATTTTCATGA-----TTATGATC-CTA--T-CTTTATTACGCTCAATTCCTCT--------------------------------GTATTAG-TATATCTAATACCTTACCCAGCCCATTTGGAAATTTTGGTTCAAACTCTTCGCTACTGGGTAAAAGATGCCCCCTCTTTGCATTTATTACGATTCTTTCTTCACGACTATCGGCATTGGACTAGTCTTTTTATTCCAAAGAAAGCCAGTTCTTTTTTTTCAAAACGAAAG------------------CAAAGATTATTCTTCTTCCTATATAATTCTCATGTATGTGAATACGAATCCATCTTCGTCTTTCTCCGTAACCAATCTTCTCATTTACAATCAACATCTTCTGGAGCCCTTCTTGAACGAATATATTTCTATGAAAAAATAGAACATCTTGTCGAAGTCTTTGCTAAA-GATTTTCAAGGCAATCTATGGTTGTTCAAGGATCCTTTCATGCATTATGTTAGGTATCAAGGAAAGTCAATTCTCGCTTCAAAGGAGAACTTTCTTTTGATGCGTAAATGGAAATATTACTTTGTACGTTTCTGGCAATGTCATTTTTACCAGTGGTTTCAACCAGGAAGGATCTATATAAACCAATTATCCAAACATTCCCTCGACCTTCTGGGCTATCTTTCAAGTGTGCGGCTAAACCCTTTAACGATACGCAGTCAAATGTTAGAAAATTCATTTCTAATCGATAATGCTGTTAAGAAGTTCGATACCATTGTTCCAATTATTCCTCTGATTGGATCATTGGTTAAAGCGAAATTTTGTAACGTATTAGGGCATCCTGTTAGTAAGGTAGTTTGGGCAGATT-ATCAGAT-------------------------------------------------------------------------------------CATCATTCGGGTTGATGAACTAAM-CCAGATAGTTATATGAGTGAAAGAAACAGCTTATAAAWTTTCAGTAAAAAGATTGAGTCTCATTTT-CTATGTAC-AAGAGTTAAAGTGAAAGTAACCATAAACATTA-GAAACGGTTTACCCCAAGATTGGTTAATTAGTGATCATGGCTTGAAGCGGGTGC-AAAAGATTAACTGTATGGGG-TTTTTACTATCTATTACCATACATGTATTACCCTAACGGGCGATTAGCAAAAAGAGGTGGATAGTTAGGAACACCAAGGTACACAAAGGATTCGTAATAGAGATTATGTAAGTTATTCAACAGAATTTTTCTGTGCATAA-AAGGAATTCTGATTGGGACTTTAAGTTGGTAGAAATGATGAAGAAGTACTCCCCCTGATTCCGATCCAGAGTATACTCCTATCCACCGATTAAGTAAATAACTATCAAGAACGAAGTAATCCTTT-ACTTT------GTTTAAAGTCCCTTTTTCTGAGAAAGGAGAATAGGAACGAAAAAAATC-------AAATAG-AAAGA-ATAGAATTGCACTAG-----AAAGAAAGAGATCTTTTTT-ATTCTTTCTTTCC-------------TCTATTTAGAGAGATAGAATTCTTGTCATCATTCGTGAACTAATGCGATGCCTAATAGTTTTTCGTAATCGAAAATGCTAGGTTG--------AAATATCTATGAATATTTGCTACAAGAAAGA-TTTTATTGAAAG-CTTAAGTTATCACTCAAC-AAAGAAAAATAAAAA-TTATT-----AAAAGATAAGATCAATTTCCG-AAGCGSWWTATTTTTCAATATAGCAGACAGAATTCCATTGTCTAATTC------------------------------------------------------------------------------------------------------------------------------------------------------TATTTAGATTA---------------------------------------------------------------------------------------------------------------------------------------------------------------------------------ATAAC------AATATTTATGATTTTTCTTT-AA-----------AAAAA--ATATGTAAGTCAA--------------------------------------------------------------------------------TGTGAAAT-AAAAAAGGAGCAATAATCCCCTTGTTGTTCTATCAAG-AGGGCGCTATTGCTCCTTTTTT-AT----TTCAAATACTCGTATACACTAAGGCCGGGTCTTATCCATTTATAGATGGAGCTTCAAGAGCAGCTAGGTCTAGAGGGAAGTTATGAGCATTACGTTCATGCATAACTTCCATACCAAGGTTAGCGCGGTTAATGATATCAGCCCAAGTATTAATTACACGACCTTGACTATCAACTACAGATTGGTTGAAATTAAAACCATTTAGGTTGAAAGCCATAGTGCTAATACCTAAAGCAGTGAACCARATACCTACTACAGGCCAAGCAGCTAGGAARAAATGTAAAGAACGAGAGTTGTTGAAACTAGCATATTGGAARATCAATCGGCCAAAATAACCATGAGCAGCTACAATATTATAAGTTTCTTCTTCTTGA-CCGAATC---TGTAACCTTCAT--------------------TTTAC-TTCCATAATCTCATCGTTTTTTTACTTCAAAATAACT-CGGGATTTAATCCCATAGAGATAATAAATCTTTCGCCTGTCAATTCA----ATGAATTACCTCTCGATGATC-TTGAAATCGGATCAATATCATGAATAACAATATCTGAGCTATCAAATCAATTCGTCGTCGAGAATTGAATAGTATAACATAGAAAGATCTTT-TATCCATACCG------AATCCAA------------------------AATTTCTTTATTTATCAATCATTCTTTTCTGTTCTTTCTTTATCTACAACCTATCTTAGGTCCTCCTTGTACAATCATCGGATAAAGTATCGTCTGACCGCCCGTCCGTTTCCATTAGTCACAAACGCCCAACAAACAATAGAAGCGAAGTGGAAAAAGAAATAAGTTACGTTCTAAACTCCG----TTTTTTTAATGATCTAGTTTTCTTGGAAGACAAAGAAGTGTGATAAAGAGGAGTTCCGGGATAAAGGATGTAATATTCCATCAAACTAACTATTTGAGTTTGGGTTTTGTTTGTTCTTCGACGGG--CCCT---------AAAAAAAATAG-AAAAAAAATAGGAAGGAAAAAT-GATTTATTCCCCTGCTACTTGCTAAGCTAAAAAAGGGGTGGGATCTTTGATTGATCTTTATTTTTCTTTTA--CCCCCCTTCCTT---------------------------------------------------------------------------------------------------------------------------------------------------------------------------------------------------------------------------------------------------------------------------------------------------------------AACGCTCTCAATAATTGTACTATTCTACATATGTCTTTCTCCTACCAATCCGTATTATTTGAAATAATGAAAATTCCCCTATTTGTTTGATGAGAAGTGC----GAAATGCCAAAGGAAA--GAAAAAAGAACCCCCTT-GGGAATG-AAATTCTGCTCCCCGTGCCCCCTTTAACAGAAAAGGG-----------------------------------------------------------------------CGGAACTCCTCAACCTGGAGTTCCACCTGAAGAAGCAGGGGCCGCGGTAGCTGCCGAATCTTCAACTGGTACATGGACAACTGTGTGGACCGATGGACTTACCAGCCTTGATCGTTACAAAGGGCGATGCTACCACATCGAGCCCGTTGCTGGAGAAGAAAATCAATTTATTGCTTATGTAGCTTACCCATTAGACCTTTTTGAAGAAGGTTCTGTTACTAACATGTTTACTTCTATTGTGGGTAATGTATTTGGGTTCAAAGCCCTGCGCGCTCTACGTCTGGAAGATCTGCGAATCCCTGCCGCTTATGCTAAAACTTTCCAAGGCCCGCCTCATGGCATCCAAGTTGAGAGAGATAAATTGAACAAGTATGGTCGCCCCCTGTTGGGATGTACTATTAAACCTAAATTGGGGTTATCTGCTAAAAACTATGGTAGAGCGGTTTATGAATGTCTACGTGGTGGACTTGATTTTACCAAAGATGATGAGAA--CGT--GAACTCCCAACCATTTATGCG-------------------

Vesalea_mexicana_BOP022787 CCTGAGCCAAATCCAGTTTTACGAAAACAAACAAGGGTTCAGAAAGCTAAAATC-AAAAAGGATAGGTGCAGAGACTCAATGGAAGCTGTTCTAACAAATGGAGTTGAC-----TGTGTTGGTAGAAAGAATCCTTCCATAGAAACTTCAGAAAGGATAAACGTATAAACATAGATATACGCATTGAAATACTAT-ATACTCTACCAAATGATTAATGACGACCCGAATCTGTATT--TA-----TATATATCAAAATGGGAGAATGGTTGTGAAGTGATTCCATATTGAAAAAAGAATCGAATATTCATTGATCAAATCATTCACTCCATAGTCTGATAGATC-TTTTGAAGAACTGATTAATTGGACGAGAATAAAGATAGAGTCCCATTCTACATGTCAATACCGGCAACAATGAAATTTATAGTAAGAGGAAAATCCGTCGACTTTAGAAATCGTGAGGGTTCAAGTCCCTCTATCCCCAAAAAACCCATATGGACTCCCTAATTATTTATCCTCTCCTT-TTATCC-TTTTTTGTTAGCGGTTAAAAATTCG------TTATCTTTCTCATTCACCCTACTCTTTTACAAAGAGATCTGAGCGGAAATGTTTTTCTCTTATC----ACAAGTCTTGTGATCTAAG----ATAA-TACGTGTWCAAATGAACATCTTTGAGTAAGGAATCCCCATTTGAATGATTCATGGTCAA------TGTCATTATTCATACTGAAACTTACAAAGTCTTCCTT-TTGAAGATCCAAGAAATTCCAGGACCTGGATAAGACTTTGTAAGACCCTTTCAATTGACA-TAGACCCGAGTTATCTAGC--AAAATGAGGATGCAGCGGTATTTATGTTAATGCACTTCCCAATGATACGTAAACAAGGCATTTCTGGTCCTTTA--------TAGAGAAGATCTATCATAGATATTTGTAATCTATCATTTATCACTTGGGGGAGGAACAATAGTATTTCATTGCTACAAGTATGGATTATTGAGAATAATAAGACATGTATTTGGATATTTCCCTTGAACTCCGCAATCTTTTTTATTTGACATGGATAGTTGAAGGGAATTTTCCGAAGAAAAAATGGATTAGATTATGGGAGTGTGTGACTTGAACTATTGATTGGTCTGTGCAGATATATGCCTTTCTCTATCTGCCACATTGGAATTCACAACCAAATGTGTCTTTGTTCCAACCATTGCGTAAGCCC----------CATACAGAGGATAGGCTGGTTCACTTGAAGAGAATCTTTTCTATGATCAGATCCGAATCATGTCGTACATGAGCAGGTTCCGTAAGATCCAGTAG-----AATAAGTGAAACTAGATAACCCATAATCGAGATTATGGGTTATCTAGTTTCACTTACATACGATTGAATAGTATGGAAATGCATTCATTTCCTATGCATTGACACGATCTATGATACTATCGGAGTGAAACAGGGGATCTAAGGAAGAACAGAGGCTAGGCTATATTAGTAACAAGTAAACCC-----TTTGTGTGTCTGTCAAAAGTCTCCAAGTATTTTGGGGATAAACACCGATCCTAAGGTCTGAGACGACCCAGAAAGCATT--TGATCCTATCATGATCCACCTTGAAAGCCTACTTGGGTATTGAGTATTTACTTGTAAGAACCAAATTTTTTGCGCTGGATAGTTGCAACTCCGGAAAAAGAAATCC------------------AGTCAAATTTTTT------TTTTAAT--------AAAATCATTCATATTATCATA-----TATGTGTGGATCTAGATAACATATAGATTTTATATGGATTCCTTATGGTTCTTTTTCTTTTTGCTCGAGCCGTATGATGAAAAATTATCATGTCCGGTTCCTTCGGGGGATGGAT-----CTATAAAAATTCACCTATCCCAATAACAAAAAAACCTGACCTGAATGATCCTGTATTAAGAGCTAAATTGGCTAAAGGTATGGGTCATAATTATTACGGAGAGCCCGCATGGCCCAATGATCTTTTATATATTTTTCCAGTAGAGATTG-----------------------AAT-----AAATCAATGCAATA-TTTTTAGGAAAGATAAAACTGGATGAATTCAAATTCGTTCAAATGGG-----------------------ATTGGAAGGTTCCTTATTTTCATTTAGGGTGTTCGTTTTATTTCTTCCCTTAGGACTTTGGTCTAGTTTAGGCTCTCTCTCCTGGAATCG-AATTGTTGT-AACTGGACGCTTCCATCCT-CTAGCTAGTA--GGGATAGAAC------AAAAAAATATTTTCA------TTTTTTAATGAATTCTTTCTCATTTATCCGATTTATCAAATTTGAAACAAAAAGATACATTTTTTCAATGAACAC-----------AAAAAATCCTAAAGTT---------ATACTATACAAAAGGTTGTCAAAATGGAATCAATTAGTTTCACCAATTCCTTA-----------------------ATTTTTACTAATGATCTTACA----TATGCCC-------TTCTATAGATAT----AGAG----------------ATAGAGAAACCC----------------------------ATTTTTCT-------TATTATA-----GATAAATAGGTTGATGGGGAAAATAAGACCCCGC-------CCTCGAAATGATAAAATCTACTAAAAAGAAAGGTAAAACCTTGTATCTTGTCTTTATTC---------------TTTTTTCAAAAGCA--------TTCTTTTTCTAATTTAGAAATTTAGTAAACAGAAGCATTTTTATTCTACTTCCATTCCCTATTGTTTTCGGCC-------AATGAATAGGGAATGGGAATTATTCATTTTATTTTTAAATTAACAATGAAATCAGAC---------AGTCAAATCAATTGGGATTATTCCAACGTTTTATGA--------CTTATTTGTTTGTCGTACAAAAAAACCTTTTACATTCCCCGTAGGTCGTACAAAAAAAC-TTTTTGAATTCCCGGT-AGAAAG----------------------------------------------CACATGAAGTAA-----GAAAAAAGTC--TTG-CTTTCTCTTTCTTTATGATATAGATATGTAATATGTACAACTTTGACCAGCAATTTCATTTAGATC-TAAGTAAGGGCTCGAAAGAT-CCAATAGACAAAT-------------ATAAAGAAAAATAAAGAAGACCC-----CTTTGATTTTGTTCCCTTTATTCCCACGGCCTGGCCTGGTCAATACCTAGCCGGGCC----TTTTTTTGTTCCAACAAATCCTAGCTAAAAGAATTTAGCTG-------------CTTTGAACACA---------------AAAATGCTTGCTATTAAAGCAGC-------------------AATAAAAAGATGAGGGGTTATTTCCATTCTTACTTAT--------------------------------------TATATATATTATT-------------------------TATATATATAAATTATATAAAATCAAAGTATCCTTTCTTATT-----ATTCCTTCTTCCCTTTTGAGTTACTTGACGACCTTACGGGAAT--ATAAAATGAAACTG-----TGGGTTCTTA---AATAATAATGAATGCATTTTTCTGTTATGATTTCAGTGGTTTTAGTGAGCCATATCTATCAAAATCCCCCCAGCAAAAGAAAAAATAGAACTT--GTTATTTC----------------ATTTAGTTATTTAAAAGAGCCC-----TCCTTTCCAGAATCTCATTAAATTGAAATCCCCCGCAAA-AAACGT-CGACACTCTCATTTTCATGA-----TTATGATC-CTA--T-CTTTATTACGCTCAATTCCTT---------------------------------GTATTAG-TATATCTAATACCTTACCCAGCCCATTTGGAAATTTTGGTTCAAACTCTTCGCTACTGGGTAAAAGATGCCCCCTCTTTGCATTTATTACGATTCTTTCTTCACGACTATCGGCATTGGACTAGTCTTTTTATTCCAAAGAAAGCCAGTTCTTTTTTTTCAAAACGAAAG------------------CAAAGATTATTCTTCTTCCTATATAATTCTCATGTATGTGAATACGAATCCATCTTCGTCTTTCTCCGTAACCAATCTTCTCATTTACAATCAACATCTTCTGGAGCCCTTCTTGAACGAATATATTTCTATGAAAAAATAGAACATCTTGTCGAAGTCTTTGCTAAA-GATTTTCAAGGCAATCTATGGTTGTTCAAGGATCCTTTCATGCATTATGTTAGGTATCAAGGAAAGTCAATTCTCGCTTCAAAGGAGAACTTTCTTTTGATGCGTAAATGGAAATATTACTTTGTACGTTTCTGGCAATGTCATTTTTACCAGTGGTTTCAACCAGGAAGGATCTATATAAACCAATTATCCAAACATTCCCTCGACCTTCTGGGCTATCTTTCAAGTGTGCGGCTAAACCCTTTAACGATACGCAGTCAAATGTTAGAAAATTCATTTCTAATCGATAATGCTGTTAAGAAGTTCGATACCATTGTTCCAATTATTCCTCTGATTGGATCATTGGTTAAAGCGAAATTTTGTAACGTATTAGGGCATCCTGTTAGTAAGGTAGTTTGGGCAGATTTATCAGA-TTAAGACAT-AAAATTTACCCTACTTCTTTATCTTTC-TAGGAAGGGCCTTTCATGAGTTGAATAGAGATTTTCA-TTTTTTATTCATCATTCGGGTTGATGAACTAAA-CCAGATAGTTATATGAGTGAAAGAAACAGCTTATAAATTTTCAGTAAAAAGATTGAGTCTCATTTT-CTATGTAC-AAGAG-TTAAGTGAAAGTAACCATAAACATTA-GAAACGGTTTACCCCAAGATTGGTTAATTAGTGATCATGGCTTGAAGCGGGTGC-AAAAGATTAACTGTATGGGG-TTTTTACTATCTATTACCATACATGTATTACCCTAACGGGCGATT-----AAAGAGGTGGATAGTTAGGAACACCAAGGTACACAAAGGATTCGTAATAGAGATTATGTAAGTTATTCAACAGAATTTTTCTGTGCATAA-AAGGAATTCTGATTGGGACTTTAAGTTGGTAGAAATGATGAAGAAGTACTCCCCCTGATTCCGATCCAGAGTATACTCCTATCCACCGATTAAGTAAATAACTATCAAGAACGAAGTAATCCTTT-ACTTT------GTTTAAAGTCCCTTTTTCTGAGAAAGGAGAATAGGAACGAAAAAAATA-------AAATAG-AAAGA-ATAGAATTGCACTAG-----AAAGAAAGAGATCTTTTTTA-----TTCTTTCC-------------TCTATTTAGAGAGATATAATTCTTGTCATCATTCGTTAACTAATGCGATGCCTAATAGTTTTTCGTAATCGAAAATGCTAGGTTG--------AAATATCTATGAATA-TTGCTACAAGAAAGA-TTTTATTGAAAG-CTTAAGTTATCACTCAAC-AAAGAAAAATAAAAA-TTATT-----AAAAGATAAGATCAA-TTCCG-AAGCGCTTTA-TTTTCAATATAGCAGACAGAATTCCATTGTCTAATTCGGG--ACTTTACGGTAGATTTTGATTCTATCTATCCTACGAATATAT-------------------CAAGATAAATAATAGCGAACTGGTCCTTAGA-TTTATTTGTGA-CCTTTGAGGAGCC-GTATGAGATGAAAATCTCATGTACGG??????????????????????????????????????????????????????????????????????????????????????????????????????????????????????????????????????????????????????????????????????????????????????????????????????????????????????????????????????????????????????????????????????????????????????????????????????????????????????????????????????????????????????????????????????????????????????????????????????????????????????????????????????????????????????????????????????????????????????????????????????????????????????????????????????????????????????????????????????????????????????????????????????????????????????????????????????????????????????????????????????????????????????????????????????????????????????????????????????????????????????????????????????????????????????????????????????????????????????????????????????TAGCAATAAATGCAAGAATATTTAC-TTCCATAATCTCATCGTTTTTTTACTTCAAAATAACT-CGGGATTTAATCCCATAGAGATAATAAATCTTTCGCCTGTCAATTCA----ATGAATTACCTCTCGATGATC-TTGAAATCGGATCAATATCATGAATAACAATATCTGAGCTATCAAATCAATTCGTCGTCGAGAATTGAATAGTATAACATAGAAAGATCTTT-TATCCATACCG------AATCCAA------------------------AATTTCTTTATTTATCAATCATTCTTTTCTGTTCTTTCTTTATCTACAACCTATCTTAGGTCCTCCTTGTACAATCATCGGATAAAGTATCGTCTGACCGCCCGTCCGTTTCCATTAGTCACAAACGCCCAACAAACAATAGAAGCGAAGTGGAAAAAGAAATAAGTTACGTTCTAAACTCCG----TTTTTTTAATGATCTAGTTTTCTTGGAAGACAAAGAAGTGTGATAAAGAGGAGTTCCGGGATAAAGGATGTAATATTCCATCAAACTAACTATTTGAGTTTGGGTTTTGTTTGTTCTTCGACGGG--CCCT--------AAAAAAAAATAG-AAAAAAAATAGGAAGGAAAAAT-AATTTATTCCCCTGCTACTTGCTAAGCTAAAAAAGGGGTGGGATCTTTGATTGATCTTTATTTTTCTTTTA-CCCCCCCTTCCTT---------------------------------------------------------------------------------------------------------------------------------------------------------------------------------------------------------------------------------------------------------------------------------------------------------------AACGCTCTCAATAATTGTACTATTCTACATATGTCTTTCTCCTACCAATCCGTATTATTTGAAATAATGAAAATTCCCCTATTTGTTTGATGAGAAGTGC----GAAATGCCAAAGGAAA--GAAAAAAGAACCCCCTT-GGGAATG-AAATTCTGCTCCCCGTGCCCCCTTTAACAGAAAAGGG--------------------------------------------------------------------------AACTCCTCAACCTGGAGTTCCACCTGAAGAAGCAGGGGCCGCGGTAGCTGCCGAATCTTCAACTGGTACATGGACAACTGTGTGGACCGATGGACTTACCAGCCTTGATCGTTACAAAGGGCGATGCTACCACATCGAGCCCGTTGCTGGAGAAGAAAATCAATTTATTGCTTATGTAGCTTACCCATTAGACCTTTTTGAAGAAGGTTCTGTTACTAACATGTTTACTTCTATTGTGGGTAATGTATTTGGGTTCAAAGCCCTGCGCGCTCTACGTCTGGAAGATCTGCGAATCCCTGCCGCTTATGCTAAAACTTTCCAAGGCCCGCCTCATGGCATCCAAGTTGAGAGAGATAAATTGAACAAGTATGGTCGCCCCCTGTTGGGATGTACTATTAAACCTAAATTGGGGTTATCTGCTAAAAACTATGGTAGAGCGGTTTATGAATGTCTACGTGGTGGACTTGATTTTACCAAAGATGATGAGAA--CGT--GAACTCCCAACCATTTATGCATA-----------------

Vesalea_occidentalis_BOP022786 CCTGAGCCAAATCCAGTTTTACGAAAACAAACAAGGGTTCAGAAAGCTAAAATCAAAAAAGGATAGGTGCAGAGACTCAATGGAAGCTGTTCTAACAAATGGAGTTGAC-----TGTGTTGGTAGAAAGAATCCTTCCATAGAAACTTCAGAAAGGATAAACGTATAAACATAGATATACGCATTGAAATACTAT-ATACTCTACCAAATGATTAATGACGACCCGAATCTGTATT--TA-----TATATATCAAAATGGGAGAATGGTTGTGAAGTGATTCCATATTGAAAAAAGAATCGAATATTCATTGATCAAATCATTCACTCCATAGTCTGATAGATC-TTTTGAAGAACTGATTAATTGGACGAGAATAAAGATAGAGTCCCATTCTACATGTCAATACCGGCAACAATGAAATTTATAGTAAGAGGAAAATCCGTCGACTTTAGAAATCGTGAGGGTTCAAGTCCCTCTATCCCCAAAAAACCCATATGGACTCCCTAATTATTTATCCTCTCCTT-TTATCC-TTTTTTGTTAGCGGTTAAAAATTCG------TTATCTTTCTCATTCACCCTACTCTTTTACAAAGAGATCTGAGCGGAAATGTTTTTCTCTTATC----ACAAGTCTTGTGATCTAAG----ATAA-TACGTGTACAAATGAACATCTTTGAGTAAGGAATCCCCATTTGAATGATTCATGGTCAA------TGTCATTATTCATACTGAAACTTACAAAGTCTTCCTT-TTGAAGATCCAAGAAATTCCAGGACCTGGATAAGACTTTGTAAGACCCTTTCAATTGACA-TAGACCCGAGTTATCTAGC--AAAATGAGGATGCAGCGGTATTTATGTTAATGCACTTCCCAATGATACGTAAACAAGGCATTTCTGGTCCTTTA--------TAGAGAAGATCTATCATAGATATTTGTAATCTATCATTTATCACTTGGGGGAGGAACAATAGTATTTCATTGCTACAAGTATGGATTATTGAGAATAATAAGACATGTATTTGGATATTTCCCTTGAACTCCGCAATCTTTTTTATTTGACATGGATAGTTGAAGGGAATTTTCCGAAGAAAAAATGGATTAGATTATGGGAGTGTGTGACTTGAACTATTGATTGGTCTGTGCAGATATATGCCTTTCTCTATCTGCCACATTGGAATTCACAACCAAATGTGTCTTTGTTCCAACCATTGCGTAAGCCC----------CATACAGAGGATAGGCTGGTTCACTTGAAGAGAATCTTTTCTATGATCAGATCCGAATCATGTCGTACATGAGCAGGTTCCGTAAGATCCAGTAG-----AATAAGTG-AACTAGATAACCCATAATCTCGATTATGGGTTATCTAG-TTCACTTACATACGATTGAATAGTATGGAAATGCATTCATTTCCTATGCATTGACACGATCTATGATACTATCGGAGTGAAACAGGGGATCTAAGGAAGAACAGAGGCTAGGCTATATTAGTAACAAGAAAACCC-----TTTGTGTGTCTGTCAAAAGTCTCCAAGTATTTTGGGGATAAACACCGATCCTAAGGTCTGAGACGACCCAGAAAGCATT--TGATCCTATCATGATCCACCTTGTAAGCCTACTTGGGTATTGAGTATTTACTTGTAAGAACCAAATTTTTTGCGCTGGATAGTTGCAACTCCGGAAAAAGAAATCC------------------AGTCAAATTTTTT------TTTTAAT--------AAAATCATTCATATTATCATA-----TATGTGTGGATCTAGATAACATATAGATTTTATATGGATTCCTTATGGTTCTTTTTCTTTTTGCTCGAGCCGTATGATGAAAAATTATCATGTCCGGTTCCTTCGGGGGATGGAT-----CTATAAAAATTCACCTATCCCAATAACAAAAAAACCTGACCTGAATGATCCTGTATTAAGAGCTAAATTGGCTAAAGGTATGGGTCATAATTATTACGGAGAGCCCGCATGGCCCAATGATCTTTTATATATTTTTCCAGTA-----------------------------------------------CAATA-TTTTTAGGAAAGATAAAACTGGATGAATTCAAATTCGTTCAAATGGG-----------------------ATTGGAAGGTTCCTTATTTTCATTTAGGGTGTTCGTTTTATTTCTTCCCTTAGGACTTTGGTCTAGTTTAGGCTCTCTCTCCTGGAATCG-AATTGTTGA-AACTGGACGCTTCCATCCT-CTAGCTAGTA--GGGATAGAAC------AAAAAAATATTTTCA------TTTTTTAATGAATTCTTTCTCATTTATCCGATTTATCAAATTTGAAACAAAAAGATACATTTTTTCAATGAACACA----------AAAAAATCCTAAAGTT---------ATACTATACAAAAGGTTGTCAAAATGGAATCAATTAGTTTCACCAATTCCTTA-----------------------ATTTTGACTAATGATCTTACA----TATGCCC-------TTCTATAGATAT----AGAG----------------ATAGAGAAACCC----------------------------ATTTTTCT-------TATTATA-----GATAAATAGGTTGATGGGGAAAATAAGACCCCGC-------CCTCGAAATGATAAAATCTACTAAAAAGAAAGGTAAAACCTTGTATCTTGTCTTTATTC---------------TTTTTTCAAAAGCA--------TTCTTTTTCTAATTTAGAAATTTAGTAAACAGAAGCATTTTTATTCTACTTCCATTCCCTATTGTTTTCGGCC-------AATGAATAGGGAATGGGAATTATTCATTTTATTTTTAAATTAACAATGAAATCAGAC---------AGTCAAATCAATTGGGATTATTCCAACGTTTTATGA--------CTTATTTGTTTGTCGTACAAAAAAACCTTTTACATTCCCCGTAGGTCGTACAAAAAAAC-TTTTTGAATTCCCGGT-AGAAAG-------------------------------------------ATACACATGAAGTAA-----GAAAAAAGTC--TTG-CTTTCTCTTTCTTTATGATATAGATATGTAATATGTACAACTTTGACCAGCAATTTCATTTAGATC-TAAGTAAGGGCTCGAAAGAT-CCAATAGACAAAT-------------ATAAAGAAAAATAAAGAAGACCC-----CTTTGATTTTGTTCCCTTTATTCCCACGGCCTGGCCTGGTCAATACCTAGCCGGGCC----TTTTTTTGTTCCAACAAATCCTAGCTAAAAGAATTTAGCTG-------------CTTTGAACACA---------------AAAATGCTTGCTATTAAAGCAGC-------------------AATAAAAAGATGAGGGGTTATTTCCATTCTTACTTAT--------------------------------------TATA--TATTATATATATTATT----------------TATATATATAAATTATATAAAATCAAAGTATCCTTTCTTATT-----ATTCCTTCTTCCCTTTTGAGTTACTTGACGACCTTACGGGAAT--ATAAAATGAAACTG-----TGGGTTCTTA---AATAATAATGAATGCATTTTTCTGTTATGATTTCAGTGGTTTTAGTGAGCCATATCTATCAAAATCCCCCCAGCAAAAGAAAAAATAGAACTT--GTTATTTC----------------ATTTAGTTATTTAAAAGAGCCC-----TCCTTTCCAAAATCTCATTAAATTGAAATCCCCCGCAAA-AAACGT-CGACACTCTCATTTTCATGA-----TTATGATC-CTA--T-CTTTATTACGCTCAATTCCTTT--------------------------------GTATTAG-TATATCTAATACCTTACCCAGCCCATTTGGAAATTTTGGTTCAAACTCTTCGCTACTGGGTAAAAGATGCCCCCTCTTTGCATTTATTACGATTCTTTCTTCACGACTATCGGCATTGGACTAGTCTTTTTATTCCAAAGAAAGCCAGTTCTTTTTTTTCAAAACGAAAG------------------CAAAGATTATTCTTCTTCCTATATAATTCTCATGTATGTGAATACGAATCCATCTTCGTCTTTCTCCGTAACCAATCTTCTCATTTACAATCAACATCTTCTGGAGCCCTTCTTGAACGAATATATTTCTATGAAAAAATAGAACATCTTGTCGAAGTCTTTGCTAAA-GATTTTCAAGGCAATCTATGGTTGTTCAAGGATCCTTTCATGCATTATGTTAGGTATCAAGGAAAGTCAATTCTCGCTTCAAAGGAGAACTTTCTTTTGATGCGTAAATGGAAATATTACTTTGTACGTTTCTGGCAATGTCATTTTTACCAGTGGTTTCAACCAGGAAGGATCTATATAAACCAATTATCCAAACATTCCCTCGACCTTCTGGGCTATCTTTCAAGTGTGCGGCTAAACCCTTTAACGATACGCAGTCAAATGTTAGAAAATTCATTTCTAATCGATAATGCTGTTAAGAAGTTCGATACCATTGTTCCAATTATTCCTCTGATTGGATCATTGGTTAAAGCGAAATTTTGTAACGTATTAGGGCATCCTGTTAGTAAGGTAGTTTGGGCAGATTTATCAGATTTAAGACAT-AAAATTTACCCTACTTCTTTATCTTTC-TAGGAAGGGCCTTTCATGAGTTGAATAGAGATTTTCA-TTTTTTATTCATCATTCGGGTTGATGAACTAAA-CCAGATAGTTATATGAGTGAAAGAAACAGCTTATAAATTTTCAGTAAAAAGATTGAGTCTCATTTT-CTATGTAC-AAGAG-TTAAGTGAAAGTAACCATAAACATTA-GAAACGGTTTACCCCAAGATTGGTTAATTAGTGATCATGGCTTGAAGCGGGTGC-AAAAGATTAACTGTATGGGG-TTTTTACTATCTATTACCATACATGTATTACCCTAACGGGCGATTAGCAAAAAGAGGTGGATAGTTAGGAACACCAAAGTACACAAAGGATTCGTAATAGAGATTATGTAAGTTATTCAACAGAATTTTTCTGTGCATAA-AAGGAATTCTGATTGGGACTTTAAGTTGGTAGAAATGATGAAGAAGTACTCCCCCTGATTCCGATCCAGAGTATACTCCTATCCACCGATTAAGTAAATAACTATCAAGAACGAAGTAATCCTTT-ACTTT------GTTTAAAGTCCCTTTTTCTGAGAAAGGAGAATAGGAACGAAAAAAATC-------AAATAG-AAAGA-ATAGAATTGCACTAG-----AAAGAAAGAGATCTTTTTTA-----TTCTTTCC-------------TCTATTTAGAGAGATAGAATTCTTGTCATCATTCGTGAACTAATGCGATGCCTAATAGTTTTTCGTAATCGAAAATGCTAGGTTG--------AAATATCTATGAATA-TTGCTACAAGAAAGA-TTTTATTGAAAG-CTTAAGTTATCACTCAACAAAAGAAAAATAAAAA-TTATT-----AAAAGATAAGATCAATTTCCGAAAGCGCTTTA-TTTTCAATATAGCAGACAGAATTCCATTGTCTAATTCGGG--ACTTTACGGTAGATTTTGATTCTATCTATCCTACGAATATAT-------------------CAAGATAAATAATAGCGAACTGGTCCTTAGATTTTATTTGTGACCCTTTGAGGAGCC-GTATGAGATGAAAATCTCATGTACGGTATTTAGATTA---------------------------------------------------------------------------------------------------------------------------------------------------------------------------------ATAAA------AATATTTACGATTTTTCTTT-AA-----------AAAAA--ATATGTAAGTCAA--------------------------------------------------------------------------------TGTGAAAT-AAAAAAGGAGCAATAATCCCCTTGTTATTCTATCAAG-AGGGCGCTATTGCTCCTTTTTT-AT----TTCAAATACTCGTATACACTAAGGCCGGGTCTTATCCATTTATAGATGGAGCTTCAAGAGCAGCTAGGTCTAGAGGGAAGTTATGAGCATTACGTTCATGCATAACTTCCATACCAAGGTTAGCGCGGTTAATGATATCAGCCCAAGTATTAATTACACGACCTTGACTATCAACTACAGATTGGTTGAAATTAAAACCATTTAGGTTGAAAGCCATAGTGCTAATACCTAAAGCAGTGAACCAGATACCTACTACAGGCCAAGCAGCTAGGAAGAAATGTAAAGAACGAGAGTTGTTGAAACTAGCATATTGGAAGATCAATCGGCCAAAATAACCATGAGCAGCTACAATATTATAAGTTTCTTCTTCTTGA-CCGAATC---TGTAACSTTCATTAGCAATAAATGCAAGAATATTTAC-TTCCATAATCTCATCGTTTTTTTACTTCAAAATAACT-CGGGATTTAATCCCATAGAGATAATAAATCTTTCGCCTGTCAATTCA----ATGAATTACCTCTCGATGATC-TTGAAATCGGATCAATATCATGAATAACAATATCTGAGCTATCAAATCAATTCGTCGTCGAGAATTGAATAGTATAACATAGAAAGATCTTT-TATCCATACCG------AATCCAA------------------------AATTTCTTTATTTATCAATCATTCTTTTCTGTTCTTTCTTTATCTACAACCTATCTTAGGTCCTCCTTGTACAATCATCGGATAAAGTATCGTCTGACCGCCCGTCCGTTTCCATTAGTCACAAACGCCCAACAAACAATAGAAGCGAAGTGGAAAAAGAAATAAGTTACGTTCTAAACTCCG----TTTTTTTAATGATCTAGTTTTCTTGGAAGACAAAGAAGTGTGATAAAGAGGAGTTCCGGGATAAAGGATGTAATATTCCATCAAACTAACTATTTGAGTTTGGGTTTTGTTTGTTCTTCGACGGG--CCCT--------AAAAAAAAATAG-AAAAAAAATAGGAAGGAAAAAT-GATTTATTCCCCTGCTACTTGCTAAGCTAAAAAAGGGGTGGGATCTTTGATTGATCTTTATTTTTCTTTTA-CCCCCCCTTCCTT---------------------------------------------------------------------------------------------------------------------------------------------------------------------------------------------------------------------------------------------------------------------------------------------------------------AACGCTCTCAATAATTGTACTATTCTACATATGTCTTTCTCCTACCAATCCGTATTATTTGAAATAATGAAAATTCCCCTATTTGTTTGATGAGAAGTGC----GAAATGCCAAAGGAAA--GAAAAAAGAACCCCCTTGGGGAATG-AAATTCTGCTCCCCGTGCCCCCTTTAACAGAAAAGGG-------------------------------------------------------------------------TAACTCCTCAACCTGGAGTTCCACCTGAAGAAGCAGGGGCCGCGGTAGCTGCCGAATCTTCAACTGGTACATGGACAACTGTGTGGACCGATGGACTTACCAGCCTTGATCGTTACAAAGGGCGATGCTACCACATCGAGCCCGTTGCTGGAGAAGAAAATCAATTTATTGCTTATGTAGCTTACCCATTAGACCTTTTTGAAGAAGGTTCTGTTACTAACATGTTTACTTCTATTGTGGGTAATGTATTTGGGTTCAAAGCCCTGCGCGCTCTACGTCTGGAAGATCTGCGAATCCCTGCCGCTTATGCTAAAACTTTCCAAGGCCCGCCTCATGGCATCCAAGTTGAGAGAGATAAATTGAACAAGTATGGTCGCCCCCTGTTGGGATGTACTATTAAACCTAAATTGGGGTTATCTGCTAAAAACTATGGTAGAGCGGTTTATGAATGTCTACGTGGTGGACTTGATTTTACCAAAGATGATGAGAA--CGT--GAACTCCCAACCATTTATGC--------------------

Weigela_florida_BOP012296 CCTGAGCCAAATCCAGTTTTCCGAAAACAAACAAGGGTTCAGAAAGCAAAAATC-AAAAAGGATAGGTGCAGAGACTCAATGGAAGCTGTTCTAACAAATGGAGTTGACTGTGTTGTGTTGGTAGAAAGAATCCTTCCATAGAAACTTCAGAAAGGATAAAGCTATAAACATAGATATACGTATTGAAATACTAT--------ATCAAATGATTAATGATGACCCGAATCTGTATCTTTATTTTATATATATCAAAATGGAAGAATTGTTGTGAAGTGATTCCATATTGAAGAAAGAATCGAATATTCATTGATCAAATCTATCACTCCATAGTCTGATAGATC-TTGTGAAGAACTGATTAATCGGACGAGAATAAAGATAGAGTCCCATTCTACATGTCAATACCGGCAACAATGAAATTTATAGTAAGAGGAAAATCCGTCGACTTTAGAAATCGTGAGGGTTCAAGTCCCTCTATCCCCAAAAAACCCATATTGACTCCCTAATTATTTATCCTCTCCTT--------TTTTTTGTTAGCGGTTCAAAATTCG------TTATCTTTCTCATTCACCCTACT-TTTTACAAAGAGATCTGAGCGGAAATGTTTTTCTCTTATC----ACAAGTCTTGTGATCTAAG----ATAA-TACGTGTACAAATGAACATCTTTGAGCAAGGAATACCCATTTGAATGATTCACGGTCAA------TATCATTATTCATACTAAAACTTACAAAGTCTTCCTT-TTGAGGATCCAAGAAATTCCAGGACCTGGATAAGACTTTGTAATACCCTTTCAATTGACA-TAGACCCGAATTATCTAGT--AAAATGAGGATGCAGCCGTATTTATGTTAATGCACTTCCCAATGATACGTAAACAAGGCATTTCTGGTCCTTTA--------TAGAGAGGATCTATCATAGATATTTGTAATCTATCATTTATCGCTTGGGGGAGGAACAATAGTATTTCATTGCTACAAGTATGGATTATTGAGAATAATAAGACATGTATTTGGATATTTCCCTTGAACTCTGCAATATTTTTTATTTGACATGAATAGTTGAAGGGAATTTTCCGAAG-AAAAATGGATTAGATTATGGGAGTGTGTGACTTGAACTATTGATTGGTCTGTGCAGATATATGCCTTTCTCTATCTGCCACATTGGAATTCACAACCAAATGTGTCTTTGTTCCAACCACCGCGTAAGCCC----------CATACAGAGGATAGGCTGGTTTGCTTGAAGAGAATCTTTTCTATGATCAGATCCGAATCATGTCGTACATGAGCAGGCTCCGTAAGATCCAGTAG-----AATAAGTG-AACTAGATAACCCATAATCTTGATTATGGGTTATCTAG-TTCACTTACTTACGATTGAATAGTATGGAAATGCATTCATTTCCTATGCATTGACACGATCTATGATACTATCGGAGTGAAACAGGAGATCTAAGGAAGAACAGAGGCTAGGCTATATTAGTAACAAGTAAACCC-----TTTGTGTGTCTGTCAAAAGTCTCCAAGTATTTGGGGGATAAACACCGATCGTAAGGTCTGAGACGACCCAGAAAGCACT--TGATCCTATCACGATCCACTTTGTAAGCCTACTTGGGTATTGAGTATTTACTTGTAAGAACCAAATTCTTTGCACTGGATAGTTGC---------AAAAGAAATCC------------------AGTTAAATTTTTC------TTTTAAA--------AAAATCATTCATATTATCATA-----TATGTGTGGATCTAGATAACATATAGATTTTATATGGATTCCTT-TGGTTCTTTTTATTTTTGCTCGAGCCGTATGATGAAAAATTATCATGTCCGGTTCCTTCGGAGGATGGAT-----CTATAAAAATTCACCTATCCCAATAACAAAAAAACCTGACCTGAATGATCCTGTATTAAGAGCTAAATTGGCTAAAGGTATGGGTCATAATTATTACGGAGAGCCCGCATGGCCCAATGATCTTTTATATATTT--------GAGATTG-----------------------AAGAAATAAAATCAATGCAATATTTTTTAGGAAAGATAAAAATGGATGAATCAAAATTCGTTCAAATGGG-----------------------ATTGGAAGGTTCATTATTTTCGTTTAGGGTGTTCGTTTTATTTCTTCCCTTAGGACTTTGGTGTAGTTTATGCTCTC----CTGGAATCG-AACTGTTGT-AACTGGACGCTTCTATCCT-CTAGCTA-----GGGATAGAAC---GAAAAAAAAATATTGTCA------TTTTTTACTGAATTCTTTCTCATTGATCCGATTTTCAAAATTTGAAACAAAAAGATACATTTTTTCAATGAACACAA---------AATAAATCCGAAAGTT--------------ATACAAAAGGTTGTCAAAATTGAATCAATTAGTTTCAACAATTCCTTA-----------------------ATTTTAACTAAAGATCTTACA----TATGCCC-------TTCTCTAGATAT------------------------AGAGAAAAA-------------------------------ACTTTTCT-------TATTGTAATTGTGACAGATAGGTTGATGGGGAAAATAAGACCTCGC-------CTTCAAAATGATAAAATCTACTAAAAAGAAAGGTAAAACCTTATATCTTGTCTTT-------------------------CAAAAGTA--------TTCTTTTTC--------AAATTTAGTAAACAGAAGCATTTTTATTCTACTTCCGTTCCCTATTGTTTTCGGCT-------AATCAATAGGGAATGGAAGTTATTCATTTT-TTTTTAGATTTACAATGAAATCAGACAATTTTTCGAGTCAAATAAATTCGGATTATTCCAATGTTTTATGA--------CTTATTTGTTTGTTGCACAAAAAAACTTTTTGAATTTCCGGTAG-------AAAGAGAT--------TTCCCCAATGACAAA-GCCATCTCTCCCAATTGAAAAAGATAATAATTACTATCTTACATTACAGATGAAGTAAGGATTGAAAAAAGTC--TTT-CTTTCTCTTTCTTTATTATATAG-------ATATGTACAACTTTTACCAGCAATTTCATTTAGATA--AAGTAAGGGCTCGAAAGAT-CCAATAGAAAAAT-------------AGAAAGAAAAATAAAGAAGACCCCGTTGCTTTGATTTTGTTCCTTTTATTCCCACGGCCTGGCCTGGTCAATACCTAGCCGGGCC----TTTTTTTGTTCCAACGAATCCTAGCTACAAGAATTTATCTG-------------ATTTGAAAACA---------------AAAATGCTTGCTATTAAAGCAACAACAAAAAGATAAAGCAACAACAAAAAGACGAGGGGCTATTTCCATTCTTA------------------------------------------------------------------------------------TTATATAAATTATATAAAATCAAAGTCTCATTTCTTATT-----ATTCCTTCTTCCTTTTTGATTTACTTGACGACCTTACGGGAAT-AAAAAAATGAAACTATGGGTTGGATTCTTAAATAATAATAATGAATGCACTTTTCTGTTATGATTTCAGCGGTTTTAGCGAGCCATATCTATCAAAATCCCTCCAGCAAAAGAAAAGATAGAACTT--GTT---------------------ATTTAGTTATTTAAAAGAGCCC-----TCCTTTCCGGAATCTCATTAAATTGAAATCCCCCGCGAA-AAACGT-CGACACTCTCATTTTCATGATTCTTTTATGATC-CTA--T-CTTTATTACGCCTAATTCCTCTCTT-CGACAAAAAGTGCATTTGTATATAATAAGTATTAGATATA-CTAATACCTTACCCAGTCCATTTGGAAATTCTGGTTCAAATTCTTCGCTACTGGGTAAAAGATGCCCCCTCTTTGCATTTATTACGATTCTTTCTCCACGAGTATCGTAATTGGACTAGTCTTATTATTCCAAAGAAAGCCGGTTCTTCTTTTTCAAAACGAAAT------------------CAAAGATTATTCTTCTTCCTATATAATTCTCATGTATGTGAATACGAATCCATCTTCGTCTTTCTCCGTAACCAATCTTCTCATTTACGATCAACATCTTCTGGAGCCCTTCTTGAACGAATATATTTCTATGAAAAAATAGAACATCTTGTAGAAGTCTTTGCTAAG-GATTTTCAGGCCAATCTATGGTTGTTCAAGGATCCTTTCATGCATTATCTTAGGTATCAAGGAAAGTCAATTCTCGCTTCAAAGGGGACCTTTCTTTTGATGAATAAATGGAAATATTATTTTGTACGTTTCTGGCAATGTCATTTTTACCAGTGGTTTCAACCAGGAAGGATCTATATAAACCAATTATCCAAACATTCCCTCGACCTTCTGAGCTATCTTTCAAGTGTGCGGCTAAACCCTTTAATGGTACGCAGTCAAATGCTAGAAAATGCATTTCTAATCGATAATGCTGTTAAGAAGTTCGATACCGTTGTTCCAATTATTCCTCTGCTTGGATCATTGGCTAAAGGGAAATTTTGTAACGTATTAGGGCATCCTGTTAGTAAGGTGGTTTGGACCGATTTATCAGATTTGAGACAT-AAAATTTACCCTACTTCTTT-TCTTTT-TAGGAAGGGCCTTTCATGAGTTGAATATATATTTTCA-TTTTTTATTCATCATTCGGGTTGATGAACTAAA-CCAGATAGTTATATGAGTGAAAGAAACAGCTTATAAATTTGCAGTAAAAAGATTGAGTCTCATTTT-CTATGTAC-AAGAG-TTAAGTGAAAGTAAACATAAACATTA-GAAACTGTTTACCCCAAGATTGGTTAATTAGTGATCATGGCTTGAAGCGGGTGC-AAAAGATCAACTGTATGGGG-TTTTTACTATCTATTACCATACATGTATTATCCTAACGGGGGATTAGCAAAAAGAGGTGGATAGTTAGGAACACCAAGGTACACAAAGGATTCGTAATAGAGATTATGTAAGTTATTCAACAGGATTTTTCTGTGCATAA-AAGGAATTCTAATTGGGACTTTAAGTTGGTAGAAATTATGAAGAAGTACTCCCCCTGATTCCGATCCAGAGTATACTCCTATCCACCGATTAAGTAAATAACTATCAAGAACGAAGTAATCCTTT-ACTTT------GTTTAAAGTCCC-TTTTCTGAGAAAGGAGAATAGGAACGAAAAAWATAAAAATAGAAATAG-AAAGA-ATATAATTGCACTAG-----AAAGAAAGAGATCTTTTTTTATTCTTTCTTTCY-------------TCTATTTAGAGAGATAGAATTCTTGTCACGATTCGTGAACTAATGTGATGTCTAATTGTTTTTCGTAATCGAAAATGCTAGGTTG--------AAATATCTATTGATA-TTGCTACAAGAAAGA-TTTTATTGAAAG-CTTAAGTTATTACTCAAC-AAAGAAAAATAAAAA-TTCTT-----AAAAGATAAGATCAA-TTCTG-AAGCGCTTTA-TTTTAAATATAGCAGACAGAATTCCATTGTCTAATTCGGG--ACCTTAAGGTAGATTTTGACTCTACCTATCCTACGAATATAT-------------------CAAGATAAATAATAGTGAACCGGTCCTTAGA-TTTATTTGTGA-CCTTCGAGGAGCC-GTATGAGGTGAAAATCTCATGTACGGTATTTAGATTACTAAAAAAATGAAATTTCTCAACATTTTTCTTTATTTTTTCTGAGATACAATTAATATTATAGATAAATAAGCAAAAAAATGCCAACATTTTATCTTTT------ATTTTAAAGGATATAAAA-------ATTTTGAATACAGAATGAAAGCCCAAAAAACAATCATGAACCAAGCCATAAAAATAAAAAGATTTACGATTTTTCTTT-AA-----------AGAAA--ATATGTAAGTCAA----------------------------------------------------------------------------ATACTGTTAAATAAAAAAAGGAGCAATAATCCCTCTTT---TCTATCAAG-AGGG-ATTATTGCTCCTTTTTTTATTAAAAAAAAAAACTCGTATACACTAATACCGGGTCTTATCCATTTGTAGATGGAGCTTCAAGAGCGGCTAGGTCTAGAGGGAAGTTATGAGCATTACGTTCATGCATAACTTCCATACCAAGGTTAGCACGGTTAATGATATCAGCCCAAGTATTAATTACACGGCCTTGACTATCAACTACGGATTGGTTGAAATTAAAACCATTTAGGTTGAAAGCCATAGTGCTGATACCTAAAGCAGTGAACCAGATGCCTACTACAGGCCAAGCAGCTAGGAAGAAATGTAAAGAACGAGAGTTGTTGAAACTAGCATATTGGAATATCAATCGGCCAAAATAACCATGAGCGGCTACGATATTATAAGTTTCTTCTTCTTGA-CCGAATC---TGTAACCTTCAT?????????????????????????????????????????????????????????????????????????????????????????????????????????????????????????????????????????????????????????????????????????????????????????????????????????????????????????????????????????????????????????????????????????????????????????????????????????????????????????????????????????????????????????????????????????????????????????????????????????????????????????????????????????????????????????????????????????????????????????????????????????????????????????????????????????????????????????????????????????????????????????????????????????????????????????????????????????????????????????????????????????????????????????????????????????????????????????????????????????????????????????????????????????????????????????????????????????????????????????????????????????????????????????????????????????????????????????????????????????????????????????????????????????????????????????????????????????????????????????????????????????????????????????????????????????????????????????????????????????????????????????????????????????????????????????????????????????????????????????????????????????????????????????????????????????????????????????????????????????????????????????????AAGATTACAAATTGACTTATTATACTCCTGACTATGAAACCAAAGATACTGATATCTTGGCAGCATT-CCGAGTAACTCCTCAACCTGGAGTTCCACCTGAAGAAGCAGGGGCCGCGGTAGCTGCCGAATCTTCAACTGGTACATGGACAACTGTGTGGACCGATGGACTTACCAGCCTTGATCGTTACAAAGGGCGATGCTACCACATCGAGCCCGTTGCTGGAGAAGAAAATCAATATATTGCTTATGTAGCTTACCCATTAGACCTTTTTGAAGAAGGTTCTGTTACTAACATGTTTACTTCTATTGTGGGTAATGTATTTGGGTTCAAAGCCCTGCGCGCTCTACGTCTGGAAGATCTGCGAATCCCTGCCGCTTATGCTAAAACTTTCCAAGGCCCGCCTCATGGCATCCAAGTTGAGAGAGATAAATTGAACAAGTATGGTCGCCCCCTGTTGGGATGTACTATTAAACCTAAATTGGGGTTATCTGCTAAAAACTACGGTAGAGCGGTTTATGAATGTCTACGTGGTGGACTTGATTTTACCAAAGATGATGAGAA--CGT--GAACTCGCAACCATT-------------------------

Zabelia_buddleioides_BOP012222 CCTGAGCCAAATCCAGTTTTCCGAAAACAAACAAGGGTTCAGAAAGCTAAAATC-AAAAAGGATAGGTGCAGAGACTCAATGGAAGCTGTTCTAACAAATGGAGTTGACTGTGTTGTGTTGGTAGAAAGAATCCTTCCATAGAAACTTCAGAAAGGATAAACCTATAAACATAGATATACGCATTGAAATACTAT-ATACTCTACCAAATGATTAATGACGACCCGAATCTGTATT--TA-----TATATATCAAAATGGGAGAATGGTTGTGAAGTGATTCCATATTGAAGAAAGAATCGAATATTCATTGATCAAATCATTCACTCCATAGTCTGATAGATC-TTTTGAAGAACTGATTAATCGGACGAGAATAAAGATAGAGTCCCATTCTACATGTCAATACCGGCAACAATGAAATTTATAGTAAGAGGAAAATCCGTCGACTTTAGAAATCGTGAGGGTTCAAGTCCCTCTATCCCCAAAAAACCTATATGAACTCCCTAATTATTTATCCTCTCCTT-TTATCC-TTTTTTGTTAGCGGTTCAAAATTCG------TTATCTTTCTCATTCACCCTACTCTTTTACAAAGAGATCTGAGCGGAAATGTTTTTCTCTTATC----ACAAGTCTTGTGATCTAAG----ATAA-TACGTGTACAAATGAACATCTTTGAGTAAGGAATCCCCATTTGAATGATTCATGGTC-----------CATTATTTATACTGAAACTTACAAAGTCTTCCTT-TTAAAGATCCAAGGAATTCCAGGACCTGGATAAGACTTTGTAATACCCTTTCAATTGACA-TAGACCCGAGTTATCTAGC--AAAATGAGGATGCGGCGGTATTTATGTTAATGCACTTCCCAATGATACGTAAACAAGGCATTTCTGGTCCTTTA--------TAGAGAAGATCTATCATAGATATTTGTAATCTATCATTTATCGCTTGGGGGAGGAACAATAGTATTTCATTGCTACAAGTATGGATTATTGAGAATAATAAGACATGTATTTGGATATCTCCCTTGAACTCCGCAATCTTTTTTATTTGACATGGAGAGTTGAAGGGAATTTTCCGAAGAAAAAATGGATTAGATTATGGGAGTGTGTGACTTGAACTATTGATTGGTCTGTGCAGATATATGCCTTTCTCTATCTGCCACATTGGAATTCACAACCAAATGTGTCTTTGTTCCAACCACCGCGTAAGCCC----------CATACAGAGGATAGGCTGGTTCACTTGAAGAGAATCTTTTCTATGATCAGATCCGAATCATGTCGTACATGAGCAGGCTCCGTAAGATCCAGTAG-----AATAAGTG-AACTAGATAACCCATAATCTCGATTATGGGTTATCTAG-TTCACTTACATACGATTGAATAGTATGGAAATGCATTCATTTCCTATGCATTGACACGATCTATGATACTATCGGAGTGAAACAGGGGATCTAAGGAAGAACAGAGGCTAGGCTATATTAGTAACAAGTAAACCC-----TTTGTGTGTCTGTCAAAAGTCTCCAAGTATTTTAGGGATAAACACCGATCATAAGGTCTGAGACGACCCAGAAAGCATT--TGATCCTATCATGATCCACCTTGTAAGCCTACTTGGGTATTGAGTATTTACTTGTAAGAACCGAATTTTTTGCGCTGGATAGTTGCAACTCCGGAAAAAGAAATCC------------------AGTCAAATTTTTC------TTTTAAT--------AAAATCATTCATATTATCATA-----TATGTGTGGATCTAGATAACATATAGATTTTATATGGATTCCTTATGGTTCTTTTTATTTTTGCTCGAGCCGTATGATGAAAAATTATCATGTCCGGTTCCTTCGGGGGATGGATCTATACTATAAAAATTCACCTATCCCAATAACAAAAAAACCTGACCTGAATGATCCTGTATTAAGAGCTAAATTGGCTAAAGGTATGGGTCATAATTATTACGGAGAGCCCGCATGGCCCAATGATCTTTTATATATTTTTCCAGTAGAGATTG-----------------------AAG-----AAATCCATGCAATATTTTTTAGGAAAGATAAAACTGGATGAATTCAAATTCGTTCAAATGGG-----------------------ATTGGAAGGTTCCTTATTTTCATTTAGGGTGTTCGTTTTATTTCTTCCCTTAGGACTTTGGTGTAGTTTATGCTCTCTCTCCTGGAATCGAATTCTTTGT-AACTGGACGCTTCTATCCT-CTAGCTAGTA--GGGATAGAAC---AAAAAAAAAAGATTTTCA------TTTTTTAATGAATTTTTTATCATTTATCCGATTTATCAAATTTGAAACAAAAAGATACATTTTTTCAATGAACACAA---------AATAAATCCTAAAGTT---------ATACTATACAAAAGGTTGTCAAAATGGCATCAATTAGTTTCATCAATTCCTTA-----------------------ATTTTAACTAACGATCTTACA----TATGCCC--------TCTATAGATATATATAGAT----------------ATAGAGAAAGCC----------------------------ATTTTTCT-------TATTATA-----GACAAATAGGTTGATGGGGAAAATAAGACTCCGC-------CCTCGAAATGATAAAATCTACTAAAAAGAAAGGTAAAACCTTGTATCTTGTCTTTATTC---------------TTTTTTCAAAAACA--------TTCTTTTTCT--------AATTTAGTAAACAGAGGCATTTTTATTCTACTTCCATTCCCTATTGTTTTTGGCC-------AATGAATAGGGAATGGAAATTCTTCATTTTATTTTTAGATTAACAATGAAATCAGACAATTTTTTGAGTCAAATCAATTCGGATTATTCCAATGTTTTATGA--------CTTATTTGTTTGTCGTACAAAAAAACTTTTTGAATTCCCGGTAG-------AAAGAGAT---------TCCCCAATGACAAAGGCCATCTCTCCCAATTGAACAAGGGAATAATGACTATGTTACATTACACATGAAGTAA-----GAAAAAAGTC--TTG-CTTTCTCTTTCTTTATTATATAG-------ATATGTACAACTTTGACCAGCAATTTCATTTAGATA-TAAGTAAGGGCTCGAAAGAT-CCAATAGACAAAT-------------AGAAAGAAAAATAAAGAAGACCC-----CTTTGATTTTGTTCCCTTTATTCCCACGGCCTGGCCTGGTCAATACCTAGCCGGGCCTTTTTTTTTTTGTTCCAACAAATCCTAGCTAAAAGAATTTATCTG-------------GTTTAAACACA---------------AAAATGCTTGCTATTAAAGCAGCAAT--------------ATAATAAAAAGATAAGGGGTTATTTCCATTCTTACTTAT--------------------------------------TATA--TATTA---------------------------TAATTATAT---TTATATAAAATCAAAGTATCCTTTCTTATT-----ATTCCTTCTTCCTTTTTGAGTTACTTGACGACCTTACGGGAAT--ATAAAATGAAACTA-----TGGATTCTTA---AATAATAATGAATGCATTTTTCTGTTATGATTTCGGTGGTTTTAGTGAGCCATATCTATCAAACCCCCCCCAGCAAAAGAAAA-ATGGAACTT--GTTATTTA----------------ATTTAGTTATTTAAAAGAGTCC-----TCCTTTCCGGAATCTCATTAAATTGAAATCCC-CGCGAA-AAACGT-CGACACTCTCATTTTCATGA-----TTATGATC-CTA--T-CTTTATTACGCCTAATTCCTCTGTT-CGACAAAAAGTCCATTTGTATATAATAAGTATTAG-TATATCTAATACCTTACCCAGCCCATTTGGAAATTTTGGTTCAAACTCTTCGCTACTGGGTAAAAGATGCCCCCTCTTTGCATTTATTACGATTCTTTCTCCACGACTATCGGCATTGGACTAGTCTTTTTATTCCAAAGAAAGCCAGTTCTTTTTTTTCAAAACGAAAG------------------CAAAGATTATTCTTCTTCCTATATAATTCTCATGTATGTGAATACGAATCCATCTTCGTCTTTCTCCGTAACCAATCTTCTCATTTACAATCAACATCTTCTGGAGCCCTTCTTGAACGAATATATTTCTATGAAAAAATAGAACATCTTGTAGAGGTCTTTGCTAAA-GATTTTCAAGGCAATCTATGGTTGTTCAAGGACCCTTTCATGCATTATGTTAGGTATCAAGGAAAGTCAATTCTCGCTTCAAAGGGGAACTTTCTTTTGATGCATAAATGGAAATATTACTTTGTACGTTTCTGGCAATGTCATTTTTACCAGTGGTTTCAACCAGGAAGGATCTATATAAACCAATTATCCAAACATTCCCTCGACCTTCTGGGCTATCTTTCAAGTGTGCGGCTAAACCCTTTAACGATACGCAGTCAAATGCTAGAAAATGCATTTCTAATCGATAATGCTGGTAAGAAGTTCGATACCGTTGTTCCAATTATTCCTCTGATTGGATCATTGGTTAAAGCGAAGTTTTGTAACGTATTAGGGCATCCTGTTAGTAAGGTGGTTTGGGCAGATTTATCAGAT??????????????????????????????????????????????????????????????????????????????????????????????????????????????????????????????????????????????????????????????????????????????????????????????????????????????????????????????????????????????????????????????????????????????????????????????????????????????????????????????????????????????????????????????????????????????????????????????????????????????????????????????????????????????????????????????????????????????????????????????????????????????????????????????????????????????????????????????????????????????????????????????????????????????????????????????????????????????????????????????????????????????????????????????????????????????????????????????????????????????????????????????????????????????????????????????????????????????????????????????????????????????????????????????????????????????????????????????????????????????????????????????????????????????????????????????????????????????????????????????????????????????????????????????????????????????????????????????????????????????????????????????????????????????????????????????????????????????????????TATTTAGATTA---------------------------------------------------------------------------------------------------------------------------------------------------------------------------------ATAAA------AAAATTTCTGATTTTTCTTTAAATTTTTCTTTAAAGAAA--ATATGTAAGTCACATACTGTTAAATAAATCAAATACTGTTAAATAAATCAAATACTGTTAAATAAATCAAATACTGTTAAATAAATCAAATACTGTTAAAT-AAAAAAGGAGCAATAATCCCTTTCTTGTTCTAGCAAG-AAGGCGCTATTGCTCCTTTTTT-ATTTATTTCAAAAACTCGTATACACTAAGGCCGGGTCTTATCCATTTGTAGATGGAGCTTCAAGAGCAGCTAGGTCTAGAGGGAAGTTATGAGCATTACGTTCATGCATAACTTCCATACCAAGGTTAGCACGGTTGATGATATCAGCCCAAGTATTAATTACACGACCCTGACTATCAACTACAGATTGGTTGAAATTAAAACCATTTAGGTTGAAAGCCATAGTGCTGATACCTAAAGCAGTGAACCAGATACCTACTACAGGCCAAGCAGCTAGGAAGAAATGTAAAGAACGAGAGTTGTTGAAACTAGCATATTGGAAGATCAATCGGCCAAAATAACCATGAGCAGCTACGATATTATAAGTTTCTTCTTCTTGA-CCGAATC---TGTAACCTTCATTAGCAATAAATGCAAGAATATTTAC-TTCCATAATCTCATCG--TTTTTACTTCAAAATAACT-CGGGATTTAATCCCATAGAGATAATAAATCTTTCGCCTGTCAATTCA----ATGAATTACCTCTCGATGATC-TTGAAATCGGATCAATATCATGAATAACAATATCTGAGCTATCAAATCAATTCGTCGTCGAGAATTGAATAGTATAACATAGAAAGATCTTT-TATCCATACCG------AATCAAA------------------------AAATTCTTTATTT----ATCATTCTTTTCTGTTCTTTCTTTATCTATAACCTAT---------TCCTTGTACAATCATCGGATAAAGTATCGTCTGACCGCCCGTCCGTTTCCATTAGTCACAAACGCCCAACAAACAATAGAAGCGAAGTGGAAAAAGAAATGAGTTACGTTCTAAACTCCG----TTTTTTTAATGATCTAGTTTTCTTGGAAGGCAAAGAAGTGTGATAAAGAGGAGTTCCGGGATAAAGGATGTAATATTCCATCAAACTAACTATTTGAGTTTGGGGTTTGTTCGTTCTTCGACGGG--CCCT-----CTAAAAAAAAAATAG----AAAAATAGGAAGGAAAAAT-GATTTATTCCCCTGCTACTTGCTAAGCTAAAAAAGGGGTGGGATCTTTGATTGATCTTTATTTTTCTTTTA-CCCCCCCTTCCTT---------------------------------------------------------------------------------------------------------------------------------------------------------------------------------------------------------------------------------------------------------------------------------------------------------------AACGCTCTCAAAAATTGTACTATTCTACATATGTCTTTCTCCTACCAATCAGTATTAGTTGAAATACTGAAAATTCCCCTATTTGTTTGATGAGAAGTGC----GAAATGCCAAAGGAAA--GAAAAAAGAACCCCCTT-GGGAATG-AAATTCTGCTCCCCGTGCCCCCTTTCACAGAAAAGGGAAGATTACAAATTGACTTATTATACTCCTGACTATGAAACCAAAGATACTGATATCTTGGCAGCATT-CCGAGTAACTCCTCAACCTGGAGTTCCGCCTGAAGAAGCAGGGGCCGCGGTAGCTGCCGAATCTTCAACTGGTACATGGACAACTGTGTGGACCGATGGGCTTACCAGCCTTGATCGTTACAAAGGGCGATGCTACCACATCGAGCCCGTTGCTGGAGAAGAAAATCAATATATTGCTTATGTAGCTTACCCATTAGACCTTTTTGAAGAAGGTTCTGTTACTAACATGTTTACTTCTATTGTGGGTAATGTATTTGGGTTCAAAGCCCTGCGCGCTCTACGTCTGGAAGATCTGCGAATCCCTACCGCTTATGTTAAAACTTTCCAAGGCCCGCCTCATGGCATCCAAGTTGAGAGAGATAAATTGAACAAGTATGGTCGCCCCCTGTTGGGATGTACTATTAAACCTAAATTGGGGTTATCTGCTAAAAACTATGGTAGAGCGGTTTATGAATGTCTACGTGGTGGACTTGATTTTACCAAAGATGATGAGAA--CGT--GAACTCCCAACCATTTATGCGTT---GGAGAGATCGATTC

Zabelia_dielsii_BOP012228 CCTGAGCCAAATCCAGTTTTCCGAAAACAAACAAGGGTTCAGAAAGCTAAAATC-AAAAAGGATAGGTGCAGAGACTCAATGGAAGCTGTTCTAACAAATGGAGTTGACTGTGTTGTGTTGGTAGAAAGAATCCTTCCATAGAAACTTCAGAAAGGATAAACCTATAAACATAGATATACGCATTGAAATACTAT-ATACTCTACCAAATGATTAATGACGACCCGAATCTGTATT--TA-----TATATATCAAAATGGGAGAATGGTTGTGAAGTGATTCCATATTGAAGAAAGAATCGAATATTCATTGATCAAATCATTCACTCCATAGTCTGATAGATC-TTTTGAAGAACTGATTAATCGGACGAGAATAAAGATAGAGTCCCATTCTACATGTCAATACCGGCAACAATGAAATTTATAGTAAGAGGAAAATCCGTCGACTTTAGAAATCGTGAGGGTTCAAGTCCCTCTATCCCCAAAAAACCTATATGAACTCCCTAATTATTTATCCTCTCCTT-TTATCC-TTTTTTGTTAGCGGTTCAAAATTCG------TTATCTTTCTCATTCACCCTACTCTTTTACAAAGAGATCTGAGCGGAAATGTTTTTCTCTTATC----ACAAGTCTTGTGATCTAAG----ATAA-TACGTGTACAAATGAACATCTTTGAGTAAGGAATCCCCATTTGAATGATTCATGGTC-----------CATTATTTATACTGAAACTTACAAAGTCTTCCTT-TTCAAGATCCAAGAAATTCCAGGACCTGGATAAGACTTTGTAATACCCTTTCAATTGACA-TAGACCCGAGTTATCTAGC--AAAATGAGGATGCGGCGGTATTTATGTTAATGCACTTCCCAATGATACGTAAACAAGGCATTTCTGGTCCTTTA--------TAGAGAAGATCTATCATAGATATTTGTAATCTATCATTTATCGCTTGGGGGAGGAACAATAGTATTTCATTGCTACAAGTATGGATTATTGAGAATAATAAGACATGTATTTGGATATCTCCCTTGAACTCCGCAATCTTTTTTATTTGACATGGAGAGTTGAAGGGAATTTTCCGAAGAAAAAATGGATTAGATTATGGGAGTGTGTGACTTGAACTATTGATTGGTCTGTGCAGATATATGCCTTTCTCTATCTGCCACATTGGAATTCACAACCAAATGTGTCTTTGTTCCAACCACCGCGTAAGCCC----------CATACAGAGGATAGGCTGGTTCACTTGAAGAGAATCTTTTCTATGATCAGATCCGAATCATGTCGTACATGAGCAGGCTCCGTAAGATCCAGTAG-----AATAAGTG-AACTAGATAACCCATAATCGAGATTATGGGTTATCTAG-TTCACTTACATACGATTGAATAGTATGGAAATGCATTCATTTCCTATGCATTGACACGATCTATGATACTATCGGAGTGAAACAGGGGATCTAAGGAAGAACAGAGGCTAGGCTATATTAGTAACAAGTAAACCC-----TTTGTGTGTCTGTCAAAAGTCTCCAAGTATTTTAGGGATAAACACCGATCATAAGGTCTGAGACGACCCAGAAAGCATT--TGATCCTATCATGATCCACCTTGTAAGCCTACTTGGGTATTGAGTATTTACTTGTAAGAACCGAATTTTTTGCGCTGGATAGTTGCAACTCCGGAAAAAGAAATCC------------------AGTCAAATTTTTC------TTTTAAT--------AAAATCATTCATATTATCATA-----TATGTGTGGATCTAGATAACATATAGATTTTATATGGATTCCTTATGGTTCTTTTTATTTTTGCTCGAGCCGTATGATGAAAAATTATCATGTCCGGTTCCTTCGGGGGATGGATCTATACTATAAAAATTCACCTATCCCAATAACAAAAAAACCTGACCTGAATGATCCTGTATTAAGAGCTAAATTGGCTAAAGGTATGGGTCATAATTATTACGGAGAGCCCGCATGGCCCAATGATCTTTTATATATTTTTCCAGTAGAGATTG-----------------------AAG-----AAATCCATGCAATATTTTTTAGGAAAGATAAAACTGGATGAATTCAAATTCGTTCAAATGGG-----------------------ATTGGAAGGTTCCTTATTTTCATTTAGGGTGTTCGTTTTATTTCTTCCCTTAGGACTTTGGTGTAGTTTATGCTCTCTCTCCTGGAATCGAATTCTTTGT-AACTGGACGCTTCTATCCT-CTAGCTAGTA--GGGATAGAAC----AAAAAAAAAGATTTTCA------TTTTTTAATGTATTTTTTATCATTTATCCGATTTATCAAATTTGAAACAAAAAGATACATTTTTTCAATGAACACAA---------AATAAATCCTAAAGTT---------ATACTATACAAAAGGTTGTCAAAATGGCATCAATTAGTTTCATCAATTCCTTA-----------------------ATTTTAACTAACGATCTTACA----TATGCCC--------TCTATAGATATATATAGAT----------------ATAGAGAAAGCC----------------------------ATTTTTCT-------TATTATA-----GACAAATAGGTTGATGGGGAAAATAAGACTCCGC-------CCTCGAAATGATAAAATCTACTAAAAAGAAAGGTAAAACCTTGTATCTTGTCTTTATTC---------------TTTTTTCAAAAACA--------TTCTTTTTCT--------AATTTAGTAAACAGAGGCATTTTTATTCTACTTCCATTCCCTATTGTTTTTGGCC-------AATGAATAGGGAATGGAAATTCTTCATTTTATTTTTAGATTAACAATGAAATCAGACAATTTTTTGAGTCAAATCAATTCGGATTATTCCAATGTTTTATGA--------CTTATTTGTTTGTCGTACAAAAAAACTTTTTGAATTCCCGGTAG-------AAAGAGAT---------TCCCCAATGACAAAGGCCATCTCTCCCAATTGAACAAGGGAATAATGACTATGTTACATTACACATGAAGTAA-----GAAAAAAGTC--TTG-CTTTCTCTTTCTTTATTATATAG-------ATATGTACAACTTTGACCAGCAATTTCATTTAGATA-TAAGTAAGGGCTCGAAAGAT-CCAATAGACAAAT-------------AGAAAGAAAAATAAAGAAGACCC-----CTTTGATTTTGTTCCCTTTATTCCCACGGCCTGGCCTGGTCAATACCTAGCCGGGCC----TTTTTTTGTTCCAACAAATCCTAGCTAAAAGAATTTATCTG-------------GTTTAAACACA---------------AAAATGCTTGCTATTAAAGCAGCAAT--------------ATAATAAAAAGATAAGGGGTTATTTCCATTCTTACTTAT--------------------------------------TATA--TATTC---------------------------TAATTATAT---TTATATAAAATCAAAGTATCCTTTCTTATT-----ATTCCTTCTTCCTTTTTGAGTTACTTGACGACCTTACGGGAAT--ATAAAATGAAACTA-----TGGATTCTTA---AATAATAATGAATGCATTTTTCTGTTATGATTTCGGTGGTTTTAGTGAGCCATATCTATCAAAACCCCCCCAGCAAAAGAAAA-ATGGAACTT--GTTATTTA----------------ATTTAGTTATTTAAAAGAGTCC-----TCCTTTCCGGAATCTCATTAAATTGAAATCCC-CGCGAA-AAACGT-CGACACTCTCATTTTCATGA-----TTATGATC-CTA--T-CTTTATTACGCCTAATTCCTCTGTT-CGACAAAAAGTCCATTTG----------GTATTAG-TATATCTAATACCTTACCCAGCCCATTTGGAAATTTTGGTTCAAACTCTTCGCTACTGGGTAAAAGATGCCCCCTCTTTGCATTTATTACGATTCTTTCTCCACGACTATCGGCATTGGACTAGTCTTTTTATTCCAAAGAAAGCCAGTTCTTTTTTTTCAAAACGAAAG------------------CAAAGATTATTCTTCTTCCTACATAATTCTCATGTATGTGAATACGAATCCATCTTCGTCTTTCTCCGTAACCAATCTTCTCATTTACAATCAACATCTTCTGGAGCCCTTCTTGAACGAATATATTTCTATGAAAAAATAGAACATCTTGTAGAGGTCTTTGCTAAA-GATTTTCAAGGCAATCTATGGTTGTTCAAGGACCCTTTCATGCATTATGTTAGGTATCAAGGAAAGTCAATTCTCGCTTCAAAGGGGAACTTTCTTTTGATGCATAAATGGAAATATTACTTTGTACGTTTCTGGCAATGTCATTTTTACCAGTGGTTTCAACCAGGAAGGATCTATATAAACCAATTATCCAAACATTCCCTCGACCTTCTGGGCTATCTTTCAAGTGTGCGGCTAAATCCTTTAACGATACGCAGTCAAATGCTAGAAAATGCATTTCTAATCGATAATGCTGGTAAGAAGTTCGATACCGTTGTTCCAATTATTCCTCTGATTGGATCATTGGTTAAAGCGAAGTTTTGTAACGTATTAGGGCATCCTGTTAGTAAGGCGGTTTGGGCAGATTTATCAGATTTAAGACAT-AAAATTGACCCTACTTCTTTATCTTTC-TAGGAAGGGCCTTTCGTGAGTTGAATAGATATTTTCA-TTTTTTATTCATCATTCGGGTTGATGAACTAAA-CCAGATAGTTATATGAGTGAAAGAAACAGCTTATAAATTTGCAGTAAAAAGATTGAGTCTCATTTTCCTATGTAC-AAGAG-TTAAGTCAAAGTAACCATAAACATTA-GAAACTGTTTACCCCAAGATTGGTTAATTAGTAATCATGGCTTGAAGCGGGTGC-AAAAGATCAACTGTATGGGG-TTTTTACTATGTATTACCATACATGTATTACTCTAACGGGCGATTAGCAAAAAGAGGTGGATAGTTAGGAACACTAAGGTACACAAAGGATTCGTAATAGAGATTATGTAAGTTATTCAACAGGATTTTTCTGTGCATAA-AAGGAATTCTGATTGGGACTTTAAGTTGGTAGAAATGATGAAGAAGTACT-CCCCTGATTCCGATCCAGAGTATACTCCTATCCACCGATTAAGTAAATAACTATCAAGAACGAAGTAATCCTTT-ACTTT------GTTTAAAGTCCCTTTTTCTGAGAAAGGAGAATAGGAACGAAAAA-ATC-------AAATAG-AAAGA-ATATAATTGTACTAG-----AAAGAAAGAGATCTTTTTTTATTCTTTCTTTCC-------------TCTATTTAGAGAGATAGAATTCTTGTCATGATTCGTGAACTAATGCGATGCCTAATTGTTTTTCGTAATCGAAAATGCTAGGTTG--------AAATATCTATGAATA-TTGCTACAAGAAAGA-TTTTATTGAAAG-CTTAAGTTATCACTCAAC-AAAGAAAAATTAAAA-TTCTT-----AAAAGATAAGATCAA-TTCCG-AAGCGCTTTA-TTTTCAATATAGCAGACAGAATTCCATTGTCTAATTCGGG--ACTTTACAGTAGATTTTGATTCTACCTATCCTAGGAATATAT-------------------CAAGATAAATAATAGCGAACTGGTCCTTAGA-TTTATTTGTGA-CCTTTGAGGAGCC-GTATGAGATGAAAATCTCATGTACGGTATTTAGATTA---------------------------------------------------------------------------------------------------------------------------------------------------------------------------------ATAAA------AAAATTTCTGATTTTTCTTTAAATTTTTCTTTAAAGAAA--ATATGTAAGTCAC---------------------------------------------------------ATACTGTTAAATAAATCAAATACTGTTAAAT-AAAAAAGGAGCAATAATCCCTTTCTTGTTCTAGCAAG-AGGGCGCTATTGCTCCTTTTTT-ATTTATTTCAAAAACTCGTATACACTAAGGCCGGGTCTTATCCATTTGTAGATGGAGCTTCAAGAGCAGCTAGGTCTAGAGGGAAGTTATGAGCATTACGTTCATGCATAACTTCCATACCAAGGTTAGCACGGTTGATGATATCAGCCCAAGTATTAATTACACGACCCTGACTATCAACTACAGATTGGTTGAAATTAAAACCATTTAGGTTGAAAGCCATAGTGCTGATACCTAAAGCAGTGAACCAGATACCTACTACAGGCCAAGCAGCTAAGAAGAAATGTAAAGAACGAGAGTTGTTGAAACTAGCATATTGGAAGATCAATCGGCCAAAATAACCATGAGCAGCTACGATATTATAAGTTTCTTCTTCTTGA-CCGAATC---TGTAACCTTCATTAGCAATAAATGCAAGAATATTTAC-TTCCATAATCTCATCG--TTTTTACTTCAAAATAACT-CGGGATTTAATCCCATAGAGATAATAAATCTTTCGCCTGTCAATTCA----ATGAATTACCTCTCGATGATC-TTGAAATCGGATCAATATCATGAATAACAATATCTGAGCTATCAAATCAATTCGTCGTCGAGAATTGAATAGTATAACATAGAAAGATCTTT-TATCCATACCG------AATCAAA------------------------AAATTCTTTATTT----ATCATTCTTTTCTGTTCTTTCTTTATCTATAACCTAT---------TCCTTGTACAATCATCGGATAAAGTATCGTCTGACCGCCCGTCCGTTTCCATTAGTCACAAACGCCCAACAAACAATAGAAGCGAAGTGGAAAAAGAAATGAGTTACGTTYTAAACTCCG----TTTTTTTAATGATCTAGTTTTCTTGGAAGGCAAAGAAGTGTGATAAAGAGGAGTTCCGGGATAAAGGATGGAATATTCCATCAAACTAACTATTTGAGTTTGGGGTTTGTTCGTTCTTCGACGGG--CCCT-----CTAAAAAAAAAATAG----AAAAATAGGAAGGAAAAAT-GATTTATTCCCCTGCTACTTGCTAAGCTAAAAAAGGGGTGGGATCTTTGATTGATCTTTATTTTTCTTTTACCCCCCCCTTCCTT---------------------------------------------------------------------------------------------------------------------------------------------------------------------------------------------------------------------------------------------------------------------------------------------------------------AACGCTCTCAAAAATTGTACTATTCTACATATGTCTTTCTCCTACCAATCAGTATTAGTTGAAATAATGAAAATTCCCCTATTTGTTTGATGAGAAGTGC----GAAATGCCAAAGGAAA--GAAAAAAGAACCCCCTT-GGGAATG-AAATTCTGCTCCCCGTGCCCCCTTTCACAGAAAAGGGAAGATTACAAATTGACTTATTATACTCCTGACTATGAAACCAAAGATACTGATATCTTGGCAGCATT-CCGAGTAACTCCTCAACCTGGAGTTCCGCCTGAAGAAGCAGGGGCCGCGGTAGCTGCCGAATCTTCAACTGGTACATGGACAACTGTGTGGACCGATGGGCTTACCAGCCTTGATCGTTACAAAGGGCGATGCTACCACATCGAGCCCGTTGCTGGAGAAGAAAATCAATTTATTGCTTATGTAGCTTACCCATTAGACCTTTTTGAAGAAGGTTCTGTTACTAACATGTTTACTTCTATTGTGGGTAATGTATTTGGGTTCAAAGCCCTGCGCGCTCTACGTCTGGAAGATCTGCGAATCCCTACCGCTTATGTTAAAACTTTCCAAGGCCCGCCTCATGGCATCCAAGTTGAGAGAGATAAATTGAACAAGTATGGTCGCCCCCTGTTGGGATGTACTATTAAACCTAAATTGGGGTTATCTGCTAAAAACTATGGTAGAGCGGTTTATGAATGTCTACGTGGTGGACTTGATTTTACCAAAGATGATGAGAA--CGT--GAACTCCCAACCATTTATGCGTT---GGAGAGATCGATTC

;

END;

BEGIN MRBAYES;

CHARSET LF = 1-858;

CHARSET PETB = 859-2062;

CHARSET RPL = 2063-3094;

CHARSET SG = 3095-4023;

CHARSET matk = 4024-4802;

CHARSET ndhA = 4803-5912;

CHARSET psbA = 5913-6718;

CHARSET psbM = 6719-7931;

CHARSET rbcL = 7932-8541;

partition parts = 9:LF,PETB,RPL,SG,matK,ndhA,psbA,psbM,rbcL;

set partition=parts;

lset applyto = (1,3,5,6,8) nst=6 rates=gamma;

lset applyto = (4) nst=2 rates=invgamma;

lset applyto = (2,7,9) nst=2 rates=gamma;

unlink shape=(all) pinvar=(all) statefreq=(all) revmat=(all);

mcmc ngen=1000000 printfreq=1000 samplefreq=100 nchains=4 savebrlens=yes checkpoint=yes checkfreq=10000;

sump relburnin=yes burninfrac=0.25;

sumt relburnin=yes burninfrac=0.25;

END;
