# Supplementary figures and images for: Molecular Phylogeny and Biogeographic Diversification of Linnaeoideae (Caprifoliaceae s. l.) Disjunctly Distributed in Eurasia, North America and Mexico
Source: PLoS One. 2015 Mar 10;10(3):e0116485. doi: 10.1371/journal.pone.0116485 (PMC4355296; doi:10.1371/journal.pone.0116485)

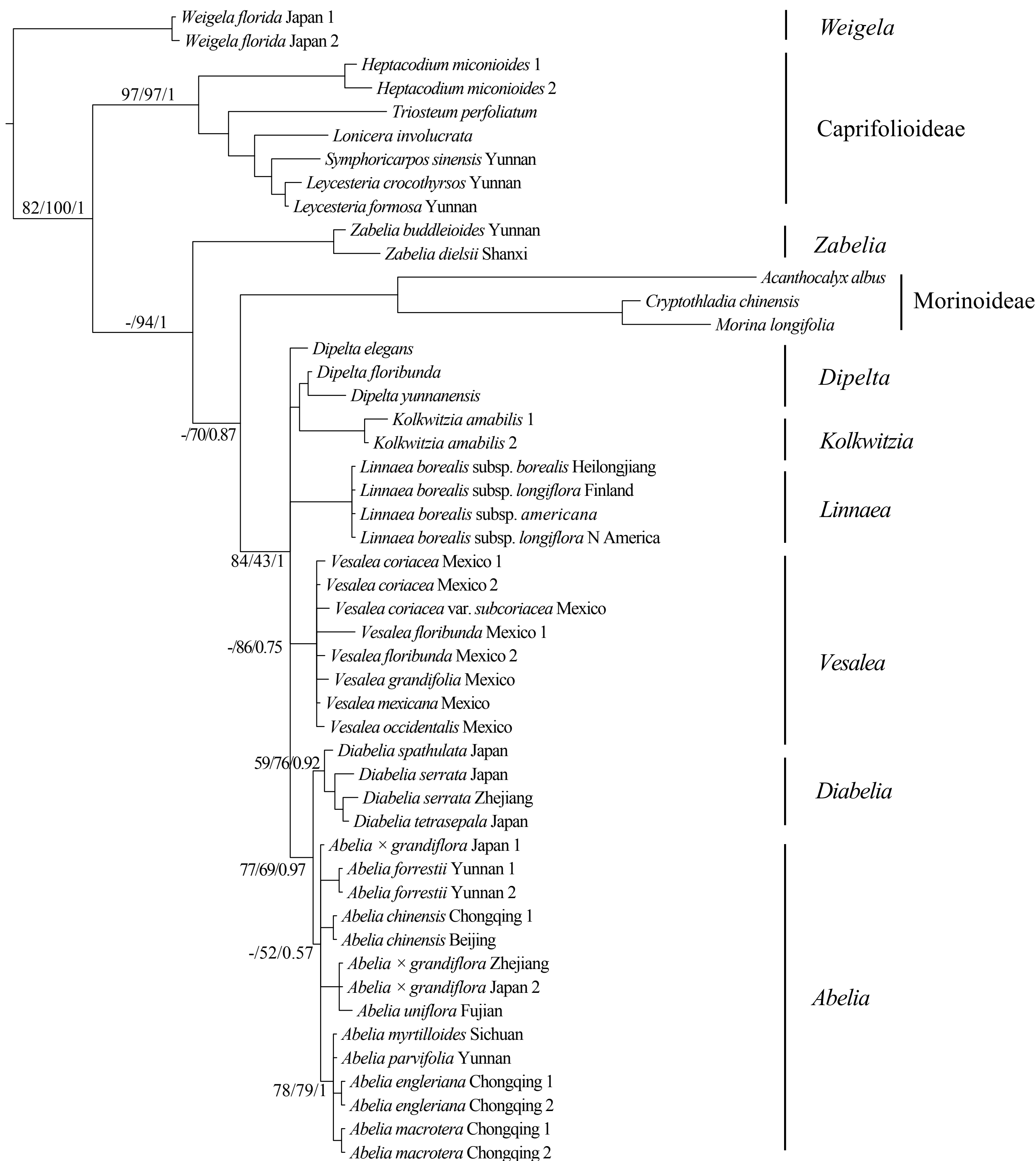

Supplement: S1 Fig — (PDF) [file pone.0116485.s002.pdf]

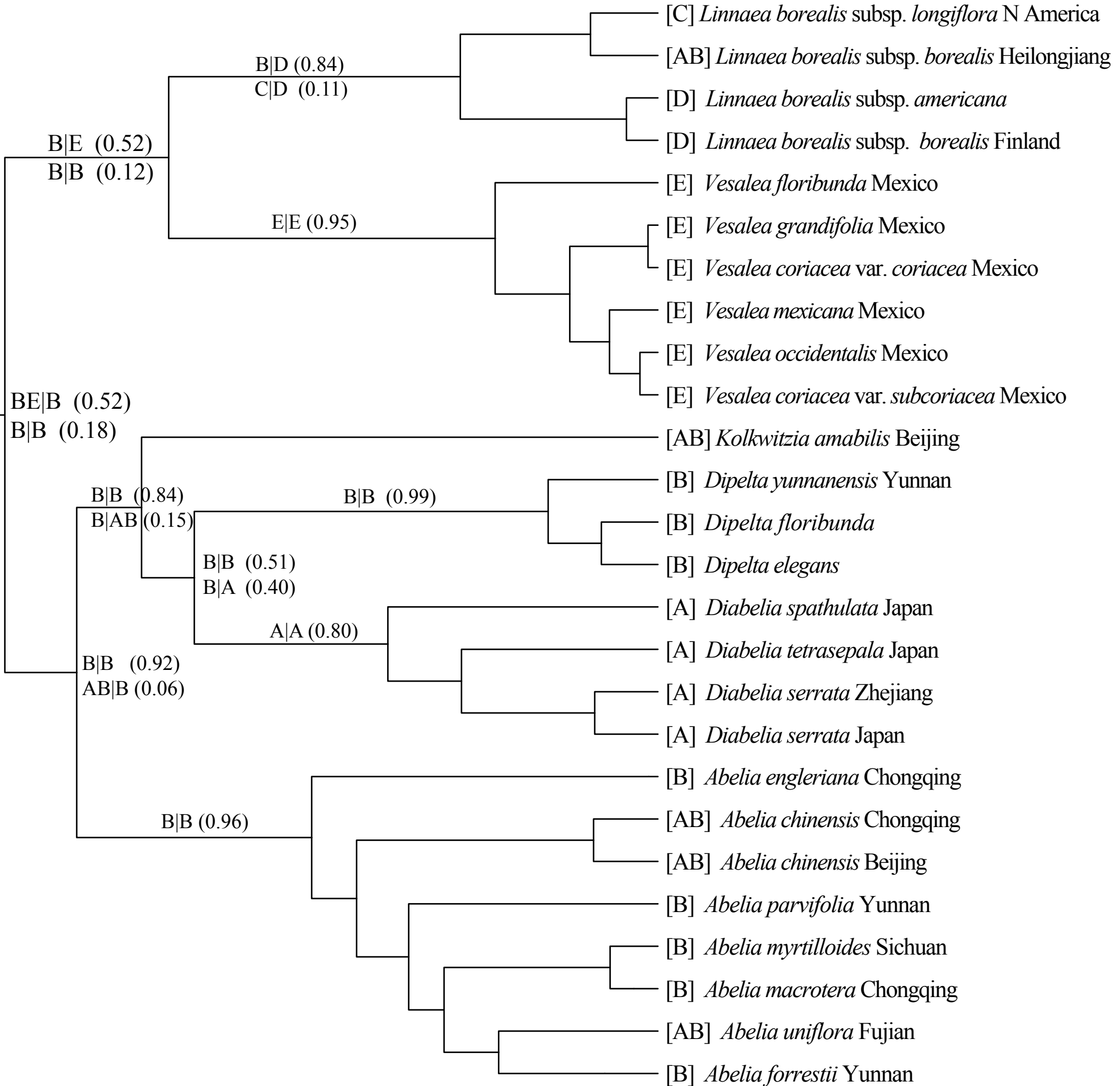

Supplement: S2 Fig — (PDF) [file pone.0116485.s003.pdf]

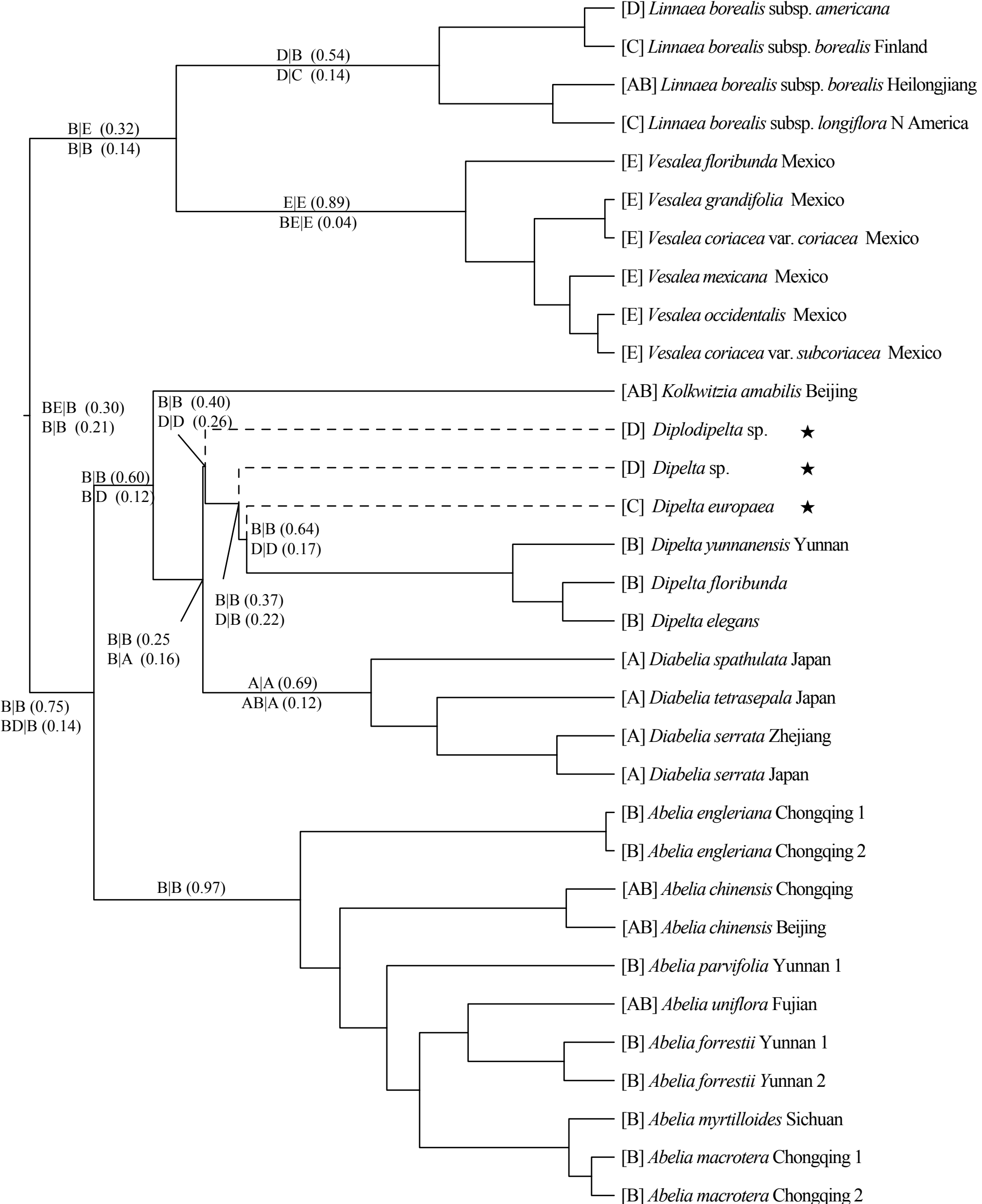

Supplement: S3 Fig — (PDF) [file pone.0116485.s004.pdf]

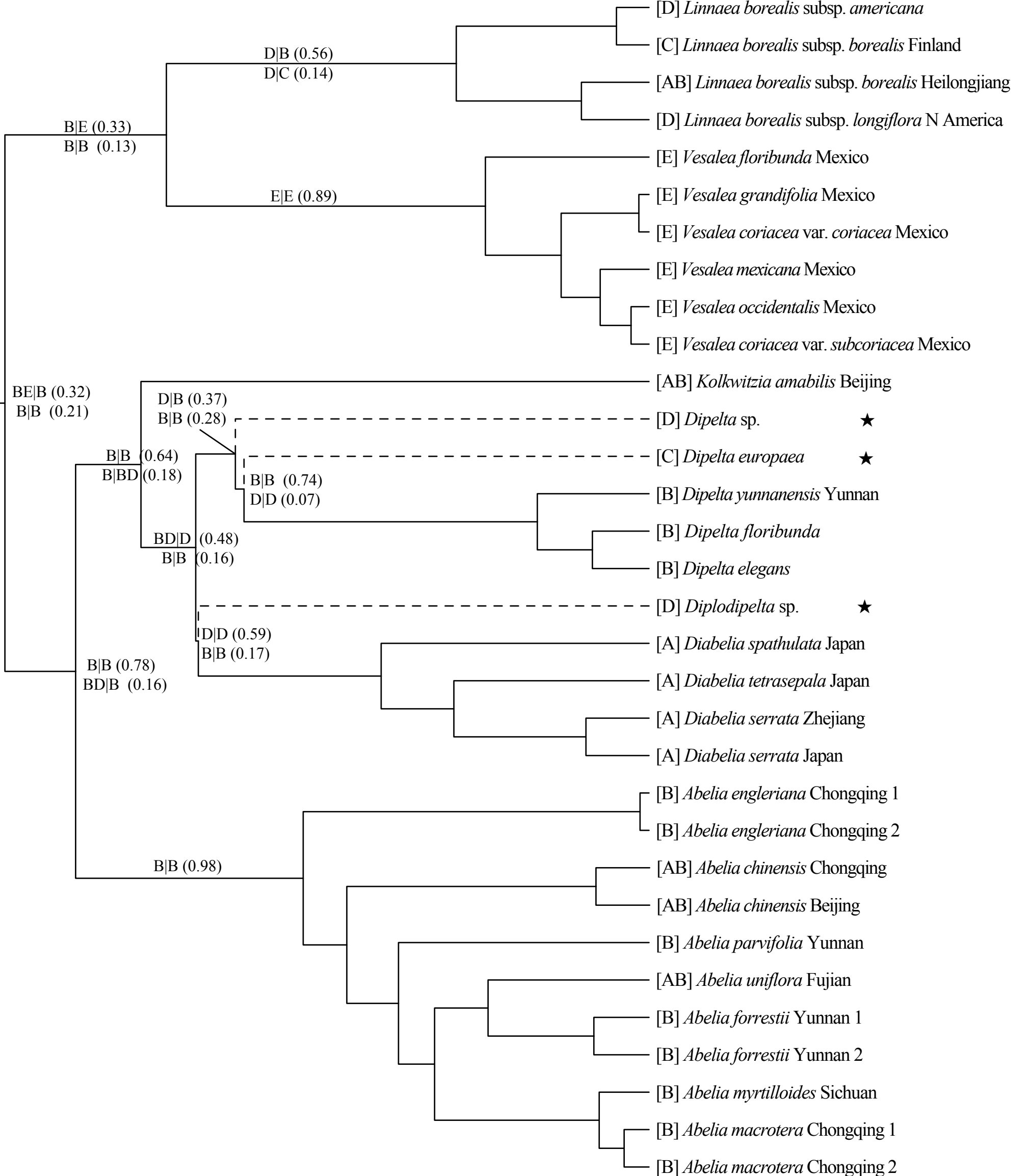

6.0

Supplement: S4 Fig — (PDF) [file pone.0116485.s005.pdf]
